# Identification and Comparative Analysis of microRNA in Wheat (*Triticum aestivum* L.) Callus Derived from Mature and Immature Embryos during *In vitro* Culture

**DOI:** 10.3389/fpls.2016.01302

**Published:** 2016-08-30

**Authors:** Zongli Chu, Junying Chen, Haixia Xu, Zhongdong Dong, Feng Chen, Dangqun Cui

**Affiliations:** National Key Laboratory of Wheat and Maize Science, Collaborative Innovation Center of Henan Grain Crops, Agronomy College, Henan Agricultural UniversityZhengzhou, China

**Keywords:** wheat (*Triticum aestivum* L.), embryogenic callus, immature embryo, mature embryo, microRNA

## Abstract

Feasible and efficient tissue culture plays an important role in plant genetic engineering. Wheat (*Triticum aestivum* L.) immature embryos (IMEs) are preferred for tissue culture to mature embryos (MEs) because IMEs easily generate embryogenic callus, producing large number of plants. The molecular mechanisms of regulation and the biological pathways involved in embryogenic callus formation in wheat remain unclear. Here, microRNAs (miRNAs) potentially involved in embryogenic callus formation and somatic embryogenesis were identified through deep sequencing of small RNAs (sRNAs) and analyzed with bioinformatics tools. Six sRNA libraries derived from calli of IMEs and MEs after 3, 6, or 15 d of culture (DC) were constructed and sequenced. A total of 85 known miRNAs were identified, of which 30, 33, and 18 were differentially expressed (*P* < 0.05) between the IME and ME libraries at 3, 6, and 15 DC, respectively. Additionally, 171 novel and 41 candidate miRNAs were also identified, of the novel miRNA, 69, 67, and 37 were differentially expressed (*P* < 0.05) between the two types of libraries at 3, 6, and 15 DC, respectively. The expression patterns of eight known and eight novel miRNAs were validated using quantitative real-time polymerase chain reaction. Gene ontology annotation of differentially expressed miRNA targets provided information regarding the underlying molecular functions, biological processes, and cellular components involved in embryogenic callus development. Functional miRNAs, such as miR156, miR164, miR1432, miR398, and miR397, differentially expressed in IMEs and MEs might be related to embryogenic callus formation and somatic embryogenesis. This study suggests that miRNA plays an important role in embryogenic callus formation and somatic embryogenesis in wheat, and our data provide a useful resource for further research.

## Introduction

Feasible and efficient tissue culture plays an important role in plant genetic engineering (Wu et al., 2003). Common wheat (*Triticum aestivum* L.; 2n = 6x = 42; AABBDD) is an important staple crop cultivated worldwide. The success of wheat genetic engineering has significantly expanded current resources and improved breeding efficiency. Immature embryos (IMEs) are the most widely used explants to initiate *in vitro* culture in wheat. These are often used in transformation research because of their ability to generate embryogenic callus and produce a large number of plants compared to mature embryos (MEs) (Tao et al., 2011). The major challenges in the use of IMEs are their temporal availability and production requirements, while MEs are easily stored and readily available. Extensive efforts have been made to improve the culture efficiency of MEs (Delporte et al., 2001, 2014; Zale et al., 2004; Filippov et al., 2006), but it remains relatively low. Embryogenic callus is derived from diverse vigorous tissues or organs by *in vitro* culture, and it contains a mass of pro-embryogenic cells that give rise to somatic embryos (Zimmerman, 1993). Thus, embryogenic callus and somatic embryos serve as a flexible *in vitro* model system for developing efficient tissue culture protocols in plants. Somatic embryogenesis is a complex biological process involving well-coordinated molecular signaling pathways (Singla et al., 2007; Yang and Zhang, 2010).

Small interfering RNAs (siRNAs) and microRNAs (miRNAs) are small RNAs (sRNAs) that control cellular metabolism and differentiation, combat viruses and mobile genetic elements in eukaryotes (Papp et al., 2003). In animals and plants, miRNAs are a type of endogenous with 20–24 nt in length RNAs that play important regulatory roles in gene expression through perfect or near-perfect complementarity with target mRNAs, facilitating mRNA cleavage or inhibiting mRNA translation (Bartel, 2004; Jones-Rhoades et al., 2006; Zhang et al., 2007; De Felippes, 2013). miRNAs are produced from miRNA genes, which are transcribed by RNA polymerase II to form primary miRNA transcripts (pri-miRNA). In higher plants, pri-miRNAs are cleaved twice by DICER-LIKE1 (an RNase III enzyme) to generate a sense-antisense miRNA (miRNA/miRNA^*^) duplex (Kurihara and Watanabe, 2004; Lee et al., 2004). The mature miRNA strand is incorporated into an RNA-induced silencing complex and then binds to its mRNA target (Chen, 2005).

In recent years, high-throughput sequencing technology has provided an alternative way to identify miRNAs and numerous miRNAs have been identified in many plant, especially in model plants and main crops such as Arabidopsis (Fahlgren et al., 2007), rice (*Oryza sativa* L.) (Jeong et al., 2011), maize (*Zea mays* L.) (Liu et al., 2014), and soybean (*Glycine max* L.) (Song et al., 2011). More evidences have shown that miRNAs are involved in the regulation of numerous biological and metabolic processes, including plant development, changes in plant vegetative phase, and resistance to biotic and abiotic stresses (Xin et al., 2010; Jeong et al., 2011).

Mounting evidences suggests that miRNAs are involved in the control of cell proliferation, meristem division and differentiation, and embryogenesis in plants (Wong et al., 2011; Lin et al., 2015). For example, four abiotic stress-induced miRNA families, miR159, miR169, miR171, and miR172, are all target transcription factors involved in the regulation of gene function in cell differentiation and development in larch (Zhang et al., 2010).

As large sections of the wheat genome are undefined, the prediction of miRNAs remains challenging. However, miRNAs have been identified from seedlings, developing grains, germ extract, leaves, roots, spikes, and mixed tissues and used to study development and stress response in wheat (Yao et al., 2007; Xin et al., 2010; Meng et al., 2013; Han et al., 2014; Sun et al., 2014; Ma et al., 2015). For example, 605 conserved (from other plant species without secondary structure prediction in wheat) and 268 novel miRNAs were identified from wheat grains (Meng et al., 2013). Recently, a whole-genome shotgun strategy was used to produce draft genomes for common wheat (Brenchley et al., 2012) and its A-genome (*Triticum urartu* Thumanjan ex Gandilyan; 2n = 14; AA) (Ling et al., 2013) and D-genome (*Aegilops tauschii* Coss; *2n* = 14; DD) progenitors (Jia et al., 2013). Moreover, 323 novel miRNAs were explored in 11 wheat tissues, and 191 wheat-specific miRNAs were screened (Sun et al., 2014). A systematic identification of the miRNA precursors and mature miRNAs in wheat group 7 chromosomes has been performed by computational analysis in isolated chromosome sequences (Deng et al., 2014). Next-generation sequencing data of individually flow-sorted chromosome arms are now partially available from the International Wheat Genome Sequencing Consortium (http://www.wheatgenome.org), while recent studies have predicted miRNAs on chromosomes 1AL, 5D, and 6B (Lucas and Budak, 2012; Kurtoglu et al., 2013; Tanaka et al., 2014).

A better understanding of the molecular mechanisms controlling embryogenic callus formation and somatic embryogenesis is crucial for biological research and its applications. MiRNAs involved in embryogenic callus formation and somatic embryogenesis have been studied in several plant species. A unique set of miRNAs expressed or differentially expressed in embryogenic callus were identified in rice (Luo et al., 2006; Chen et al., 2014); miRNAs and their target genes were analyzed in cotton (*Gossypium hirsutum* L.) revealing their regulation role during somatic embryogenesis (Yang et al., 2013), miRNA expression during somatic embryogenesis in citrus (*Citrus sinensis* L.) shows that miR156, miR168, and miR171 as well as miR159, miR164, miR390, and miR397 are related to somatic embryo induction or formation (Wu et al., 2011). Stage-specific expression of miRNAs and their targets in larch (*Larix leptolepis* Gordon) indicate that miR171a/b might exert function on proembryogenic masses, miR171c acts in the induction process; miR162 and miR168 exert their regulatory function during total somatic embryogenesis process (Zhang et al., 2012). However, the identification and possible modulation of miRNA in embryogenic callus formation in wheat has rarely been studied. There are obvious differences in callus development between MEs and IMEs during *in vitro* culture. IME induces embryogenic callus and embryogenesis within 15 d of culture (DC), whereas MEs fail to produce embryogenic callus under the same experimental conditions. To some extent, miRNA activity may be associated with embryogenic callus formation and somatic embryogenesis. The aim of this study was to analyze the differences in miRNA expression between IMEs and MEs and determine the function of their targets. The results presented here provide new insight into the embryogenic callus formation in wheat.

## Materials and methods

### Plant material and sample preparation

Wheat cultivar Zhoumai 18 was used in this experiment. Plants were grown in an experimental field of Henan Agricultural University, Zhengzhou, China, and the selfing-pollination had performed for three consecutive years. During the winter of 2011–2012 and headed May and June 2012, mature seeds from the same spike were harvested and labeled for the next year planting. Plants derived from a single spike were grown in the same experimental field in a line during the winter of 2012, and in December 2012, those with substantial tillers from the same line were selected and transplanted into a greenhouse under conditions of 75% relative humidity, 26/20°C day/night temperature, 12-h light/dark photoperiod, and light intensity of 10,000 lx. Anthesis was recorded in February 2013, when 50% of the plants reached the flowering stage. Immature seeds from an individual plant were collected 14 d after anthesis from the spikes of the main section and were used for culture. Well-developed mature seeds from the same plant were collected from the spike of the main section in April and soaked in running tap water for 4 h. Immature and mature seeds from the same plant were surface-sterilized for 30 s in 75% (v/v) ethanol, followed by immersion for 6 min in 0.1% (m/v) mercuric chloride solution with agitation, and then rinsed four times with sterilized distilled water. Immature and mature embryos were extracted from sterilized seeds on a clean bench, placed with the scutellum upwards in sterile petri dishes containing solid Murashige and Skoog (MS) medium (Murashige and Skoog, 1962) (MS basal salts, B5 vitamins, 30 g L^−1^ sucrose, 5.5 g L^−1^ agar, and 2 mg L^−1^ 2,4-dichlorophenoxyacetic acid with pH 5.8). They were then grown in a culture room at 22–24°C in the dark. MEs and IMEs were cultured in three biological replicates, each replicate consisting of 15 plates and each plate with 10 embryos. The embryos were harvested at 3, 6, and 15 DC from 5 plates in each of the three replicates, snap-frozen in liquid nitrogen, and stored at −80°C until RNA extraction.

### sRNA library construction and RNA sequencing

Total RNA was extracted from IME and ME callus in three biological replicates using TRIzol reagent (Invitrogen, Carlsbad, CA, USA) according to the manufacturer's instructions. The integrity of RNA samples was checked by 1% agarose gel electrophoresis. RNA samples of the three biological replicates were mixed in equal amount and used for the construction of libraries. Thus, for each treatment, a sample of 700-μg RNA from the three replicates with an equal amount of total RNA were generate and was fractionated on a 15% denaturing polyacrylamide gel. sRNA regions corresponding to 18–30 nt were excised and recovered. These sRNAs were then 5′ and 3′ RNA adapter-ligated using T4 RNA ligase (Takara, Dalian, China). Ligated products were purified using an Oligotex mRNA mini kit (Qiagen, Hilden, Germany) and subsequently transcribed into cDNAs via a SuperScript II RT (Invitrogen, USA). PCR amplifications were performed with primers that annealed to the ends of the adapters. The final quality of the cDNA library was ensured using an Agilent2100 Bioanalyzer (Agilent Technologies, Santa Clara, CA, USA) and examining its size, purity, and concentration. PCR products were sequenced using a HiSeq 2000 Sequencing System (Illumina, San Diego, CA, USA) according to the manufacturer's instructions.

### Bioinformatics analysis and miRNA identification

Sequence tags from deep sequencing were processed by Phred and Crossmatch (http://www.phrap.org/phredphrapconsed.html), filtering out low quality tags and eliminating contamination of adaptor sequences not ligated to any other sequences. The remaining high-quality sRNA reads were trimmed from their adapter sequences. Reads with a length of 18–30 nt were compared against Rfam (http://rfam.xfam.org) (Gardner et al., 2009), GenBank, and RepBase (http://www.girinst.org/repbase/) (Kapitonov and Jurka, 2008) databases to annotate RNA categories including tRNA, rRNA, small cytoplasmic RNA (scRNA), small nuclear RNA (snRNA), and small nucleolar RNA (snoRNA) from each database. sRNA reads with sequence similarity more than 90% to these sequences were removed. Putative origins for the remaining sequences were identified by BLASTN search against the wheat expressed sequence tags (ESTs) database from NCBI or the contig sequences from WGS assembly. The protein-coding EST sequences were removed, and the remaining non-coding sequences were retained for miRNAs identification. To identify previously known miRNAs, these sRNA sequences were aligned to known pre-miRNAs in miRBase 21.0 (http://www.mirbase.org) using the criterion of perfect match. The putative pre-miRNAs in wheat genome and the processing patterns (e.g., miRNA/miRNA^*^) of known (non-wheat reference) miRNAs from other plant species found in miRBase were further detected in the same way as novel miRNAs (as described in the following section). The remaining sRNA sequences were retained to predicate novel miRNAs. Putative pre-miRNAs were searched, excluding the EST sequences from NCBI or the contig sequences from WGS assembly, and their potential to form a hairpin secondary structure was predicted using RNAfold (http://rna.tbi.univie.ac.at/cgi-bin/RNAfold.cgi). miRNAs were predicted using MIREAP (http://sourceforge.net/projects/mireap/) by exploring DCL1 cleavage sites and the minimum free energy (maximal free energy allowed for a miRNA precursor is −18 kcal/mol). Candidates of miRNA with more than 20 reads in one library were used for the following analysis. The main miRNA sequence tag must cover at least 70% of all reads surrounding the miRNA start site, from 20 nt upstream to 20 nt downstream of the flank sequence site of miRNA precursor. The following key criteria were used for miRNA prediction: (1) miRNAs and miRNAs^*^ were derived from opposite stem-arms, such that they formed a duplex with two-nucleotide 3′ overhangs; (2) the base-pairing between miRNA and the other arm of the hairpin, which included miRNA^*^, was extensive, typically including four or fewer mismatched miRNA bases; five mismatched bases were allowed, if an miRNA^*^ was detected, (3) no asymmetric bulges larger than 2 nt and no more than two asymmetric bulges were present within the miRNA/miRNA^*^ duplex (Meyers et al., 2008; Yue et al., 2012); and (4) the reads number was greater than 5 in either library (Sun et al., 2014). Sequences that met these criteria and with miRNA^*^ detected were considered as novel miRNAs, whereas that met the criteria but no miRNA^*^s were detected were considered as candidate miRNAs (Han et al., 2014). All novel miRNA identified in this research were aligned to other reported wheat microRNAs using the criterion of perfect match.

### Differential expression analysis of miRNAs

The frequency of each identified miRNA read count was normalized according to the expression of transcripts per million (TPM) to the total clean reads of miRNA in each sample. For each sample, TPM was calculated as actual miRNA count/total count of clean reads × 1,000,000. The fold-change of miRNA expression between the IMEs and MEs libraries at each DC was calculated as log_2_ (IMEs/MEs). The Bayesian method was then applied to evaluate statistical significance. If the *P*-value was = 0.05 and with the normalized sequence count log_2_ (IMEs/MEs) >1 or <−1, the specific miRNA was considered to be differentially expressed.

### miRNA target prediction and annotation

Putative known and novel miRNA sequences were aligned against available wheat genome sequences (including the wheat NCBI EST database or the WGS contig sequences or wheat A-genome progenitor *T. urartu* [2*n* = 14; AA] and D-genome progenitor *A. tauschii* [2*n* = 14; DD]) and using Target Finder (https://github.com/carringtonlab/TargetFinder?) predicted putative miRNA targets. Default parameters and a prediction cut off value of 3 were used. BLASTN hits possessing less than four mismatches were chosen as candidate targets; for the non-target predicted miRNA, the psRNA Target software (http://plantgrn.noble.org/psRNATarget/) (version 12) was used to predict the targets in wheat transcripts with: prediction score cutoff value = 3.0, length for complementarity scoring = 20, and target accessibility = 25. To obtain their functions, the putative target genes of the miRNAs were BLASTX and subjected to GO analysis.

### miRNA quantification by real-time PCR

Reverse transcription (RT) reactions were performed in three independent biological replicates with RNA that was extracted from three independent biological samples of six types of callus individually, which were performed in a final volume of 20 μL using a One Step PrimeScript® miRNA cDNA Synthesis Kit (Takara, China). Reactions were performed at 37°C for 60 min, 85°C for 5 s, and then kept at 4°C, following the manufacturer's instructions. Real-Time PCR was performed using a Bio-Rad IQ5 Real-Time PCR Detection System (BIO-RAD, Hercules, CA, USA) with SYBR® Premix Ex Taq II™ (Takara, China). Each reaction included 2.0 μL of diluted RT reaction product, 1.0 μL of each primer (forward and reverse), 12.5 μL of SYBR® Premix Ex Taq™ (Perfect Real Time; Takara, China), and 8.5 μL of nuclease-free water. All qRT-PCR reactions were incubated in a 96-well plate at 95°C for 30 s, followed by 40 cycles at 95°C for 5 s, 61°C for 30 s, and 72°C for 30 s. All reactions were run in triplicate.

Wheat U6 [GenBank: X63066] snRNA was used as an endogenous control. The specificity of each primer pair was verified by agarose gel electrophoresis analysis and melting curve analysis. The relative abundance of each miRNA was calculated by a comparative C_T_ method (ΔΔC_T_) using the formula 2^−ΔΔC_T_^ (Livak and Schmittgen, 2001). The miRNA sample in the ME library with the C_T_ value was selected as the calibrator, in which the expression level was set to 1.0. The relative expression levels of the same miRNA in the corresponding IME library were then normalized by comparison. All primers used in this study are presented in Table S7.

### Target validation of miRNAs by 5′ RLM-RACE

The cleavage sites of the predicted targets for the two novel miRNAs were determined by 5′RLM-RACE. An M-MuLV First Strand cDNA Synthesis Kit (Sangon, Shanghai, China) was used for the first stand cDNA synthesis from total mRNA. The reaction mixture, which included 10 μL of total RNA, 1.0 μL of 3′ adaptor was incubated for 5 min at 70°C and then chilled for 2 min on ice. Thereafter, 4.0 μL of 5 × First-Strand Buffer, 2 μL of 10 mmol dNTP, 1 μL of RNase inhibitor, and 2 μL of reverse transcriptase were added to make total volume of 20 μL. The reaction mixtures were incubated for 60 min at 42°C, followed by 10 min at 72°C. After cDNA synthesis, removal of RNA template in the cDNA:RNA hybrid, purification of the first strand product (TaKaRaLATaq), TdT tailing of cDNA, and PCR of dC-tailed cDNA (RT1, 6 × DNA Loading Dye) were conducted according to the Invitrogen 5′ RACE system manual (Invitrogen, USA), and followed by nested PCR. Gene-specific reverse primers and 5′ RACE adapter specific primers were used for amplification of target genes. For the first PCR, each reaction included 12.5 μL of 2 × GC Buffer I, 1 μL of cDNA solution, 0.5 μL of forward (AAP) and reverse (R1) primer (10 μM), 4 μL of dNTP mix (2.5 mM), 0.2 μL of Taq (5 U/μL), and 6.3 μL of RNase Free water. The reactions at 95°C for 3min were followed by 33 cycles at 94°C for 30 s, 68°C for 30 s, 72°C for 60 s and a final extension at 72°C for 7 min. For the second PCR, the total reaction volume was 50 μL and it included 1 μL of the PCR product from the first PCR, 12.5 μL of RNase Free water, forward (AUAP) and reverse (R2) primer (10 μM), and the remaining three components were added in double amounts of those in the first PCR. The PCR protocol was identical to the first PCR with the exception of the step at 68°C, which was set to 68 s. The PCR products were eluted, cloned in a pGEMT Easy vector (Promega, USA) and sequenced to map the cleavage site. The primers used are provided in Table S8.

### Targets quantification by real-time PCR

A total of 14 target genes, of which miRNAs have been validated by qRT-PCR or were involved in embryogenic callus formation (including tae-miR164, zma-miR156k-5p, ata-miR396b-5p, sbi-miR1432, novel-m0837_5p, novel-m0411_5p, novel-m0713_3p, novel-m0130_3p and novel-m0143_5p), were selected for expression profile validation by qRT-PCR. A PrimeScript® RT reagent Kit with gDNA Eraser (Perfect Real Time; Takara, China) was used for the RT reactions. First genomic DNA elimination reaction was conducted in a final volume of 10 μL including 2.0 μL of 5 × gDNA Eraser Buffer, 1.0 μL of gDNA Eraser, 2.0 μL of total RNA, and 5 μL of RNase Free dH_2_O. Reactions were incubated at 42°C for 2 min, and then kept at 4°C. The RT reactions (10.0 μL) were then used for SYBR® Green qPCR assay in a 20-μL reaction mixture that included 4.0 μL of 5 × PrimeScript® Buffer 2 (for real time), 1.0 μL of PrimeScript® RT Enzyme Mix I, 1.0 μL of RT Primer Mix, and 4.0 μL of RNase Free dH_2_O. The reactions were incubated at 37°C for 15 min, followed by 85°C for 5 s, and then kept at 4°C. Real-time PCR was conducted on a Bio-Rad CFX96TM Real-Time System with SYBR® Premix Ex Taq II™ (Takara, China). Each reaction included 2 μL of product from the diluted RT reactions (cDNA solution), 1.0 μL of forward and reverse primer (10 μM), 12.5 μL of SYBR® Premix Ex Taq™ II (2 ×), and 8.5 μL of RNase Free water. The reactions were incubated in a 96-well plate at 95°C for 30 s, followed by 40 cycles at 95°C for 5 s, 60°C for 30 s, and 72°C for 30s. The actin gene (GenBank: AB181991) was used as the endogenous control. All reactions were run in triplicate. The specificity of each primer pair was verified by agarose gel electrophoresis and melting curve analysis. The relative expression levels of target genes were calculated by a comparative C_T_ method (ΔΔC_T_) using the formula 2^−ΔΔC_T_^ (Livak and Schmittgen, 2001). The target sample in the ME library with the C_T_ value was selected as the calibrator, in which the expression level was set as 1.0. The relative expression levels of the same target in the corresponding IME library were then normalized by comparison. All primers used in this study are presented in Table S7.

## Results

### Phenotypic analysis of IME and ME cultures

**Figure 1:**
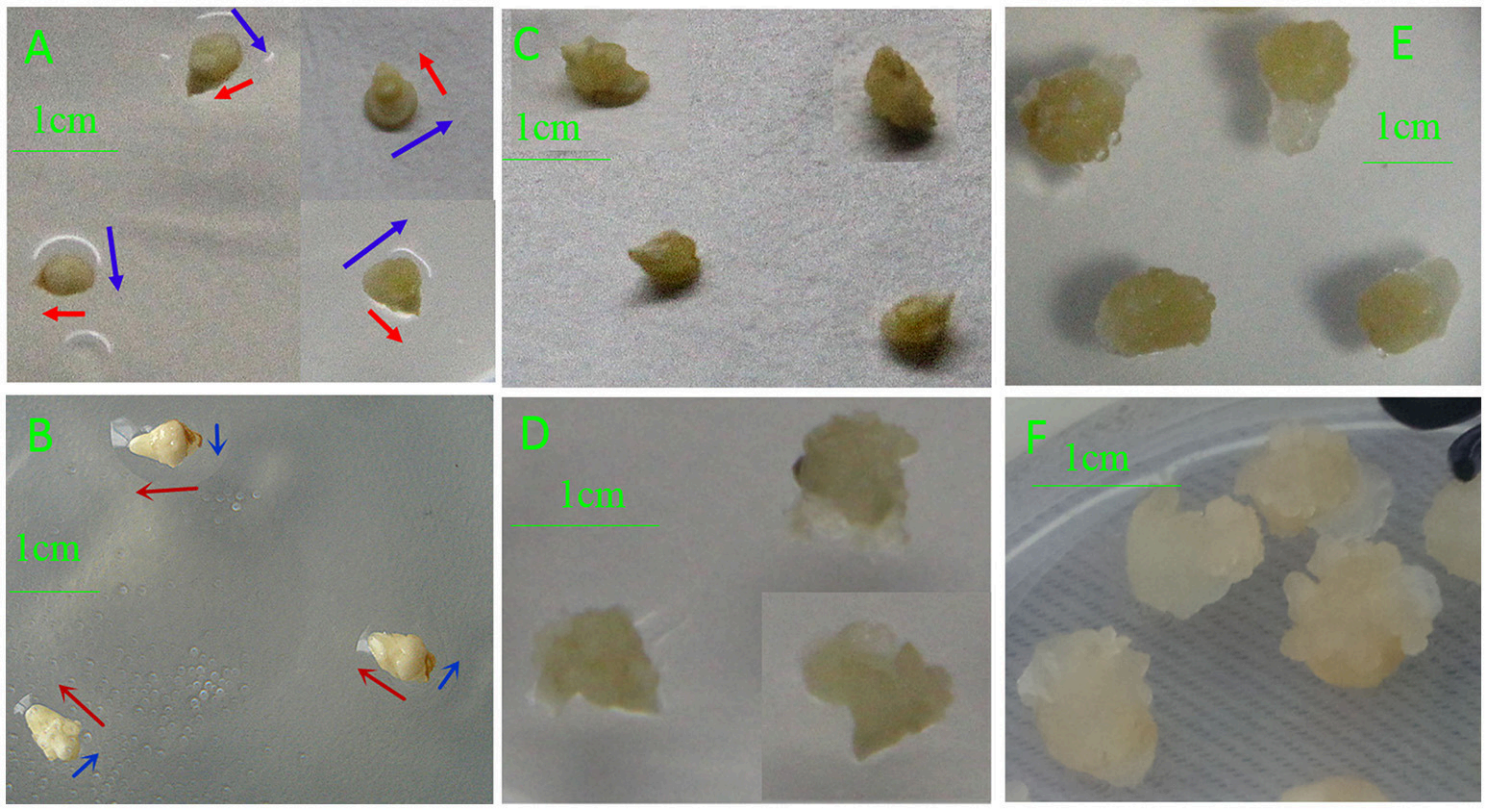
**Phenotypic differences in developing callus derived from immature and mature embryos under ***in-vitro*** culture conditions**. **(A)** IME3; **(B)** ME3; **(C)** IME6; **(D)** ME6; **(E)** IME15; **(F)** ME15. IME3, IME6, and IME15 are immature embryos at 3, 6, and 15 d of culture, respectively. ME3, ME6, and ME15 are mature embryos at 3, 6, and 15 d of culture, respectively. Blue arrows: development in horizontal direction; Red arrows: development in vertical direction.

Different phenotypes were observed between IMEs and MEs at 3, 5, and 15 DC (Figure 1). IMEs at 3 DC (IME3) mainly developed from the scutellum (IMEs widened, which phase we labeled horizontal development; Figure 1A), whereas ME3 mainly developed from germ layers (MEs elongate, which process was labeled vertical development; Figure 1B). At 6 DC, granular embryogenic callus was induced from IMEs (IME6; Figure 1C), while the callus of ME6 enlarged sequentially and turned transparent (Figure 1D). Somatic embryogenesis was visible in IMEs at 15 DC (IME15; Figure 1E), while there was no embryogenic callus in ME15 (Figure 1F). Therefore, embryogenic callus and somatic embryos were obtained from IMEs but not from MEs at 15 DC. These results allowed us to compare the expression of miRNAs between the two types of embryo-derived callus at the formation stage.

### Deep-sequencing of sRNAs in embryo culture

**Figure 2:**
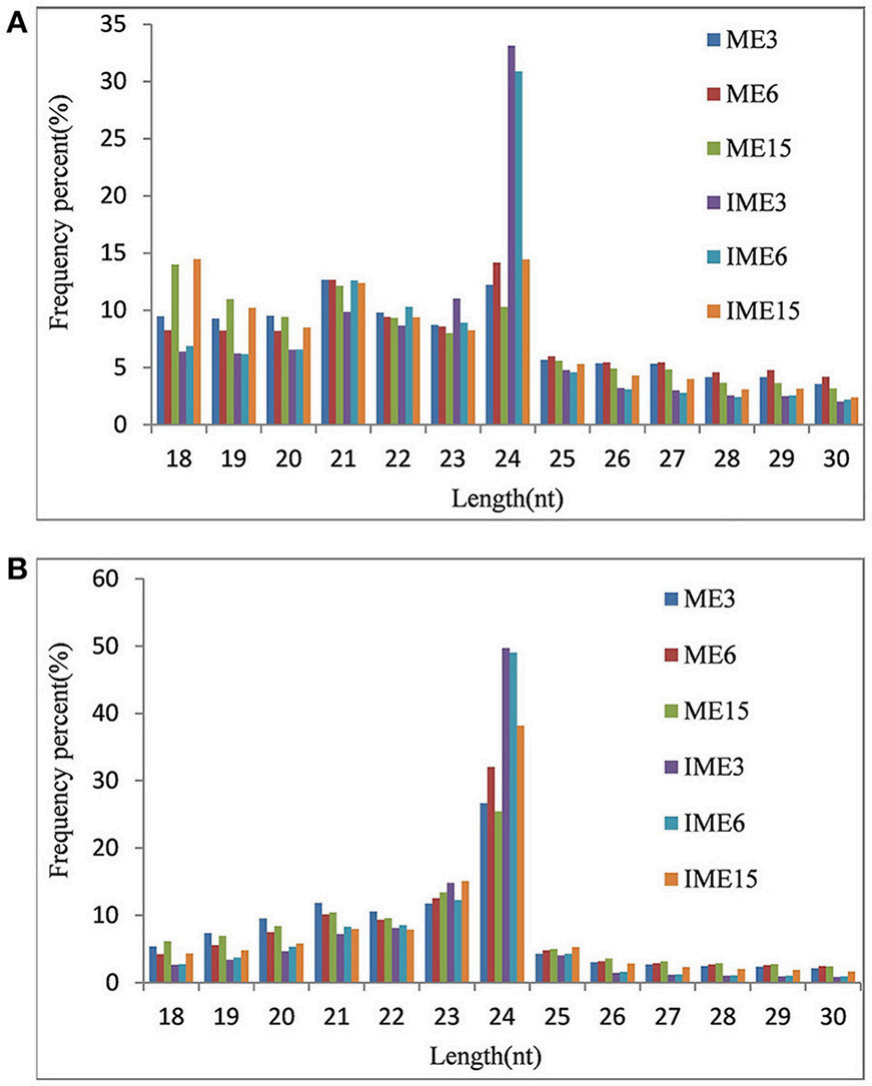
**Length distributions of small RNAs (sRNAs) in developing callus derived from immature and mature embryos under ***in-vitro*** culture conditions**. IME3, IME6, and IME15 are immature embryos at 3, 6, and 15 d of culture, respectively. ME3, ME6, and ME15 are mature embryos at 3, 6, and 15 d of culture, respectively. **(A)** Redundant sRNAs; **(B)** Unique sRNAs.

To study the role of miRNAs in embryogenic callus and somatic embryogenesis, callus from IMEs and MEs at 3, 6, and 15 DC was used to construct six sRNA libraries. All libraries were sequenced and 16–24 million raw reads were produced from each library. After the removal of adapter sequences, sequences less than 18 nt and more than 30 nt in length, and low quality reads, 10.8 million (mean; range 7.5–13.1 million) clean sRNAs were obtained from each library, and 3.5 million (mean; range 1.5–6.3 million) of them were unique (Table 1). The number of unique reads in the IME3 and IME6 libraries was more than twice the number of reads in the ME3 and ME6 libraries. Likewise, the number of unique reads in the IME15 library was higher than that in the ME15 library (Table 1). The length of sRNAs in the six libraries ranged from 18 to 30 nt; the majority of sRNAs in each library was 21–24 nt in length (Figures 2A,B), with 24 nt being the most common length of unique sequences, followed by 23 nt.

**Table 1:** **Sequencing data of six small RNA libraries derived from callus of mature and immature embryos at 3, 6, and 15 d of culture**.

**Type**	**Number of reads Percentage of reads (%)**					
	**ME3**	**ME6**	**ME15**	**IME3**	**IME6**	**IME15**
Raw reads	24,358,302 **100**	19,864,585 **100**	16,785,212 **100**	21,635,426 **100**	20,882,605 **100**	22,343,378 **100**
Low quality reads	1,027,168 **4.22**	918,725 **4.62**	0 **0.00**	821,116 **3.80**	748,056 **3.58**	1,315,631 **5.89**
Having-'N' reads	23,812 **0.10**	19,297 **0.10**	19,306 **0.12**	21,612 **0.10**	21,075 **0.10**	16,089 **0.07**
Length <18 nt	7,986,857 **32.79**	4,580,458 **23.06**	7,181,669 **42.79**	5,507,749 **25.46**	5,348,951 **25.61**	8,774,178 **39.27**
Length >30 nt	3,420,719 **14.04**	3,990,426 **20.09**	2,080,054 **12.39**	2,166,250 **10.01**	2,857,509 **13.68**	1,927,863 **8.63**
Clean reads	11,899,746 **48.85**	10,355,679 **52.13**	7,504,183 **44.71**	13,118,699 **60.64**	11,907,014 **57.02**	10,309,617 **46.14**
Unique reads	2,920,515 **11.99**	2,590,246 **13.04**	1,538,356 **9.16**	6,351,457 **29.36**	5,470,746 **26.20**	2,272,913 **10.17**

*ME3, ME6, and ME15 are mature embryos at 3, 6, and 15 d of culture, respectively*.

*IME3, IME6, and IME15 are immature embryos at 3, 6, and 15 d of culture, respectively*.

sRNA categories were determined and the annotated RNAs were removed, the remaining unannotated sequences accounted for 47% (mean; range 35–67%) of redundant reads and 91% (mean; range 85–95%) of unique reads (Table S1). Of these unannotated sequences, 62% of the redundant reads and 41.93% of the unique reads were matched perfectly to those from the whole genome shotgun (WGS) assembly (http://mips.helmholtz-muenchen.de/plant/wheat/uk454survey/download/index.jsp) and the National Center for Biotechnology Information (NCBI) database (Table 2).

**Table 2:** **Redundant and unique reads mapped in six small RNA libraries derived from callus of mature and immature embryos at 3, 6, and 15 d of culture**.

**Library**	**Number of redundant reads (%)**		**Number of unique reads (%)**	
	**Unannotated**	**Mapped**	**Unannotated**	**Mapped**
ME3	4,294,177	2,062,137 (48.02)	2,615,380	995,149 (38.05)
ME6	4,225,193	2,221,093 (52.53)	2,321,771	939,389 (40.46)
ME15	2,626,660	1,392,313 (53.01)	1,321,879	549,983 (41.60)
IME3	8,437,101	4,180,808 (49.55)	6,092,413	2,726,624 (44.75)
IME6	7,995,909	4,154,382 (51.96)	5,242,235	2,271,101 (43.32)
IME15	4,070,679	2,226,314 (54.69)	2,076,096	901,093 (43.40)

*ME3, ME6, and ME15 are mature embryos at 3, 6, and 15 d of culture, respectively*.

*IME3, IME6, and IME15 are immature embryos at 3, 6, and 15 d of culture, respectively*.

### Identification of known miRNAs

Unannotated unique sRNA sequences perfectly matched to the WGS assembly were used to identify known miRNAs. A total of 85 known miRNAs were identified in the six sRNA libraries, including 68 miRNAs from wheat found in miRBase/*Triticum aestivum* (Table S2-1) and 17 miRNAs from other plant species that were identified for the first time in wheat herein. Among these 17 miRNAs from other plant species that were identified for the first time in wheat, 13 pre-miRNAs were predicted to generate miRNAs^*^ with an abundance of more than five reads in the six libraries (Table 3; Table S2-2). The secondary structures of these miRNAs are shown in **Figure 4A** and Image S1.

**Table 3:** **Seventeen known sense microRNAs (miRNAs) and their anti-sense miRNAs**.

**miRNA**	**Raw reads of sense miRNA**						**Raw reads of antisense miRNA**					
	**ME3**	**ME6**	**ME15**	**IME3**	**IME6**	**IME15**	**ME3**	**ME6**	**ME15**	**IME3**	**IME6**	**IME15**
vvi-miR171e	6508	10,616	3521	7996	3426	3674	6	1	4	17	11	6
ata-miR9863a-3p	31,628	45,630	38,856	34,801	60,015	30,691	293	154	226	187	260	293
sbi-miR1432	63	42	25	182	118	42	2	0	3	3	4	2
ata-miR396b-5p	120,667	124,882	31,183	43,892	71,351	47,918	0	0	5	3	0	0
mdm-miR171i	161	205	227	7	86	168	7	7	12	1	7	7
gma-miR393j	84	61	40	152	106	18	10	8	6	11	11	10
rco-miR156c	2503	3746	2932	6566	9142	2923	12	9	47	55	27	12
hvu-miR1120	154	231	103	77	175	114						
ata-miR319-3p	68	85	63	38	111	110	7	4	12	11	24	7
aly-miR166c-3p	4217	3011	847	4889	3597	607						
zma-miR156k-5p	33	55	37	114	174	35						
bdi-miR1878-3p	130	340	376	198	477	927	5	5	10	8	14	5
bdi-miR167g	1711	1886	1410	2661	3436	1833	28	16	50	40	30	28
ata-miR9674b-3p	4511	3660	2487	3106	4288	2154	61	23	16	100	42	61
mtr-miR164c	366	538	211	999	844	299						
ata-miR168-5p	592	573	746	649	990	891	514	367	950	507	533	514
lja-miR396	18,013	14,553	11,450	16,823	31,540	24,391	296	307	253	540	463	296

*ME3, ME6, and ME15 are mature embryos at 3, 6, and 15 d of culture, respectively*.

*IME3, IME6, and IME15 are immature embryos at 3, 6, and 15 d of culture, respectively*.

**Figure 3:**
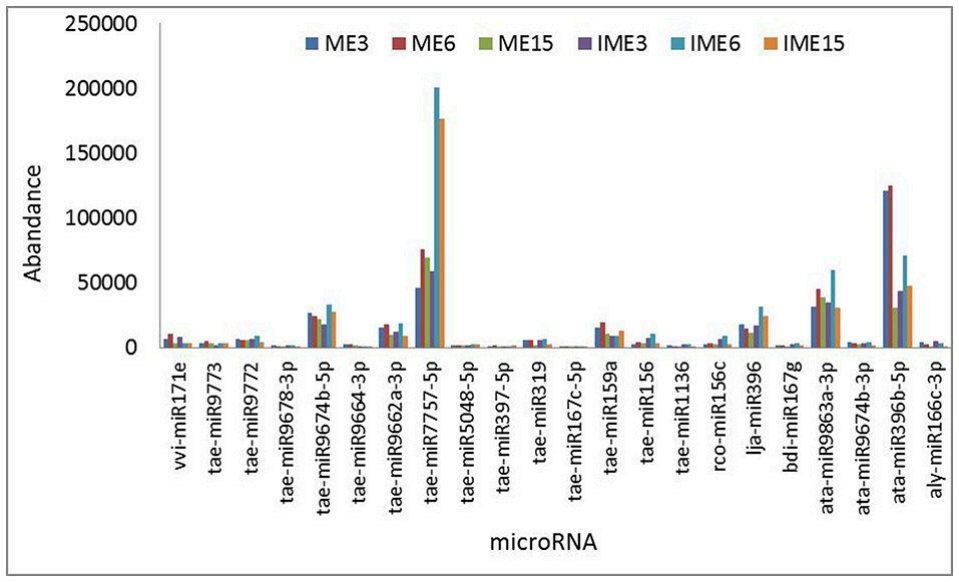
**Most abundantly expressed known microRNAs (miRNAs) involved in callus development**. Reads of the most abundant miRNAs among all miRNAs are given.

As shown in Figure 3, the miR7757 and miR396 families were the most abundantly expressed, while the miR9863, miR9674, and miR9662 families were moderately abundant. The majority of abundantly expressed miRNAs were known miRNAs from the miRBase of wheat or they were deeply conserved families such as miR156, miR159, and miR171. Conserved miRNAs were expressed at relatively higher levels than non-conserved miRNAs (Table S2), similarly to other species (Li et al., 2011; Yanik et al., 2013).

### Predicted novel mIRNAs

Unannotated sequences with no similarity to known miRNAs were used to predict novel miRNAs. A total of 171 novel miRNAs were identified, of which 131 had the total reads of miRNAs^*^ more than five in all the six libraries (Table S3-1). Forty-one sequences without miRNAs^*^ were identified as candidate miRNAs (Table S3-2)

Several studies have reported the identification of miRNAs in wheat (around 1598 miRNA sequences have been identified), but very few of them have been submitted to the miRbase. To confirm the novelty of miRNAs, we searched the miRNAs identified in this research against all previously published wheat miRNA (Xin et al., 2010; Meng et al., 2013; Han et al., 2014; Sun et al., 2014; Ma et al., 2015) (Table S4). A total of 21 miRNAs in our novel miRNA dataset (of the 171 miRNAs) shared identical sequences with the previously reported novel miRNAs (Table S3-1, in yellow), and 3 miRNAs in our candidate miRNA dataset (of the 41 candidate miRNAs) shared identical sequences with the previously reported novel miRNAs (Table S3-2, in yellow).

**Figure 4:**
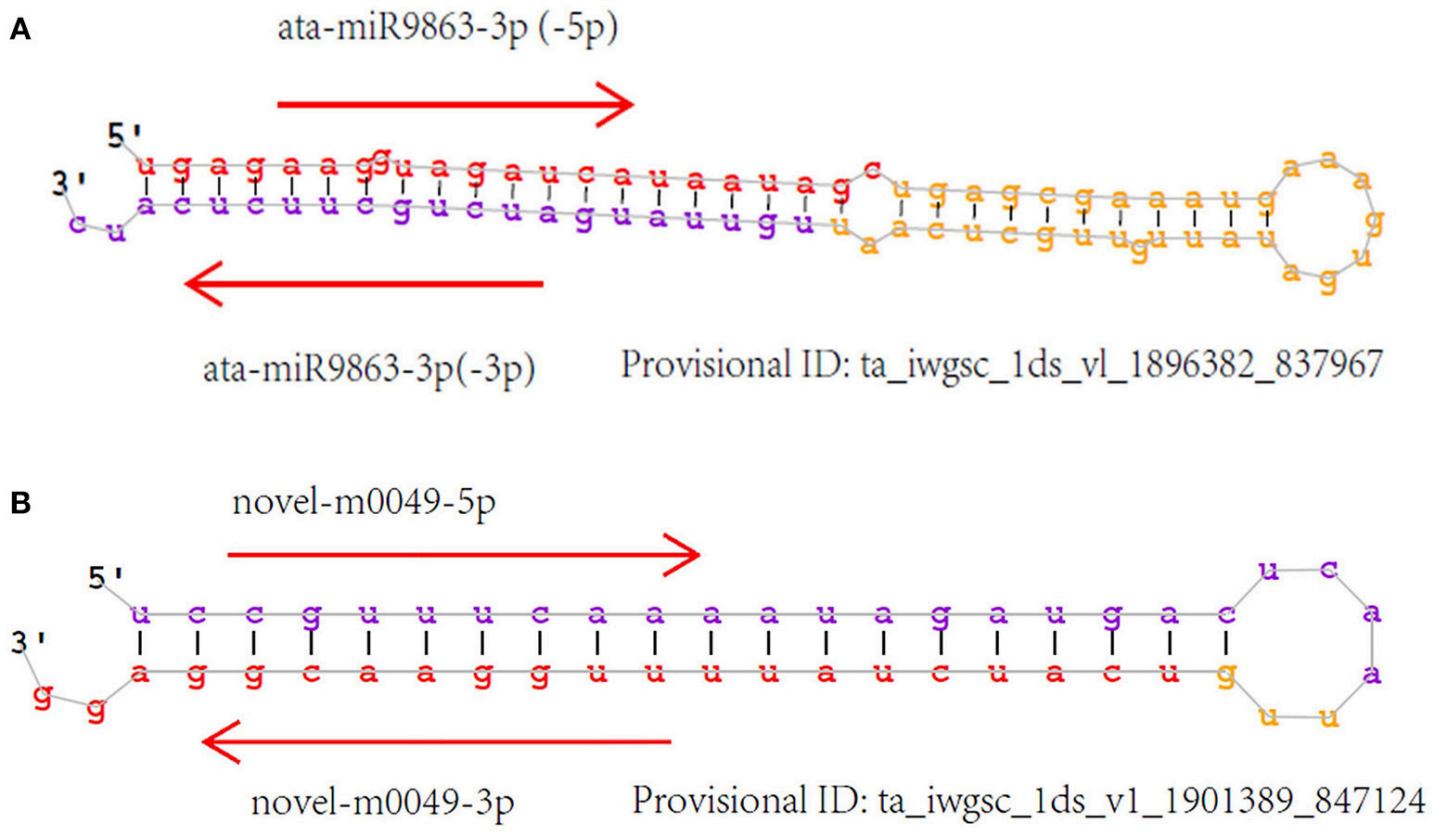
**Secondary structure of identified miRNAs**. Sense miRNAs (in red) and antisense miRNAs (in purple). **(A)** Known miRNAs, first found in wheat; **(B)** Novel miRNAs.

The minimum free energy of pre-miRNAs ranged from −93.80 kcal mol^−1^ to −29.60 kcal mol^−1^ (Table S3-1), with an average of about −48.60 kcal mol^−1^, indicating the high stability of hairpin structures. Detailed information of all the novel miRNAs is provided in Table S3-1, and the secondary structure of several novel pre-miRNAs is shown in Figure 4B and Image S2.

The expression levels of novel miRNAs ranged broadly. Most of them had a relatively low expression level (39 novel miRNAs with a total of less than 120 raw reads in all six libraries), whereas 11 novel (including 3 reported novel) miRNAs had a total of more than 1000 reads. The most abundant novel miRNAs were novel-m0064_5p, novel-m0492_5p, and novel-m0661_5p with a total of more than 5000 reads (Table S3-1).

### Differentially expressed mIRNAs between calli from IMEs and MEs

**Figure 5:**
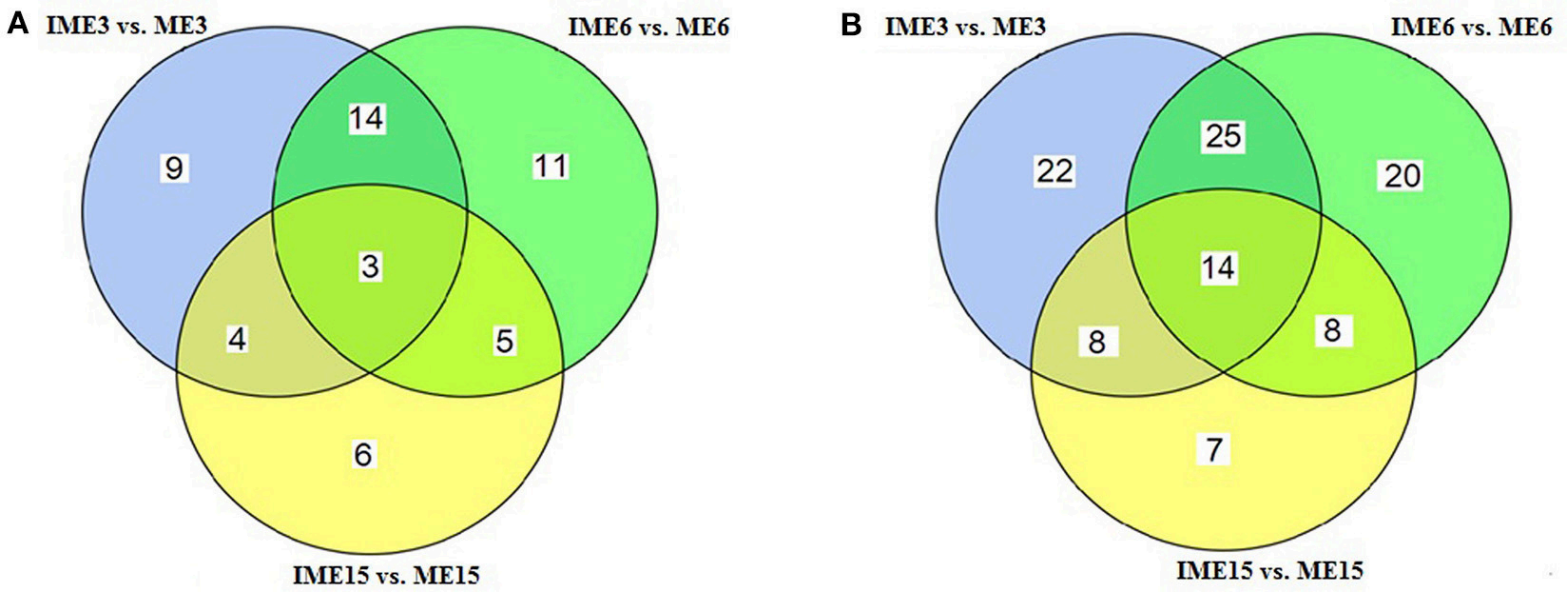
**Venn diagram of the differentially expressed miRNAs at different stages of callus development. (A)** Known miRNAs; **(B)** Novel miRNAs.

To identify miRNAs associated with embryogenic callus formation, the differentially expressed miRNAs in the IME and corresponding ME libraries were compared using a statistical method developed by Audic and Claverie (1997). A total of 52 known and 104 novel (including 9 reported novel) miRNAs were differentially expressed (*P* < 0.05) between the IME and ME libraries at all DC (Figure 5). For known miRNAs, 30 (17 up-regulated), 33 (24 up-regulated), and 18 (3 up-regulated) miRNAs were differentially expressed between the IME and ME libraries at 3, 6, and 15 DC, respectively (**Tables 5–7**; Table S5-1). For novel miRNAs, 69 (62 up-regulated), 67 (59 up-regulated), and 37 (21 up-regulated) miRNAs were differentially expressed between the IME and ME libraries at 3, 6, and 15 DC, respectively (Table S5-2).

Of all the differentially expressed miRNAs between the IME and ME libraries, 3 known and 14 novel (2 candidate) miRNAs had the same differential expression at all DC (Figure 5; Tables S5-1–S5-3). For IME3 vs. ME3 and IME6 vs. ME6, 17 known and 39 novel miRNAs showed same differential expression, while for IME6 vs. ME6 and IME15 vs. ME15, eight known and 22 novel miRNAs shared the same differential expression (Figure 5).

The comparison between the developmental stages of IME and ME identified 62 known and 111 novel miRNAs that were differentially expressed for IME6 vs. IME3, IME15 vs. IME6, ME6 vs. ME3, and ME15 vs. ME6 (Tables 5–8; Tables S5-1, S5-2). In total, there were 68 known and 132 novel miRNAs differentially expressed between IME and ME and between the developmental stages of IME and ME (Tables S5-1, S5-2), as well as there were 46 known and 83 novel miRNAs commonly differentially expressed in two types of comparisons. For IME6 vs. IME3, IME15 vs. IME6, ME6 vs. ME3, and ME15 vs. ME6, the ratio of up-regulated miRNA was 21/26, 6/37, 10/12, and 8/20 for known miRNA, respectively and 16/17, 2/83, 22/27, and 7/27 for novel miRNA, respectively (Tables S5-1, S5-2). The number of all differentially expressed and up-regulated miRNA in each comparison are listed in Table 4.

**Table 4:** **The number of differentially expressed and up-regulated miRNAs**.

	**Known**		**Novel**		**Candidate**	
	**Total**	**Up**	**Total**	**Up**	**Total**	**Up**
IME3 vs. ME3	30	17	69	62	19	13
IME6 vs. ME6	33	24	67	59	13	11
IME15 vs. ME15	18	3	37	21	8	6
IME6 vs. IME3	26	21	17	16	10	10
IME15 vs. IME6	37	6	83	2	20	2
ME6 vs. ME3	12	10	27	22	7	5
ME15 vs. ME6	20	8	27	7	10	1

*ME3, ME6, and ME15 are mature embryos at 3, 6, and 15 d of culture, respectively*.

*IME3, IME6, and IME15 are immature embryos at 3, 6, and 15 d of culture, respectively*.

**Table 5:** **The up-regulated known miRNAs in IME vs. ME and their target function**.

**miRNA**	**Transcripts per million (TPM)**						**Fold-change**							**Target function**
							**IME vs. ME**			**IME vs. IME**		**ME vs. ME**		
	**ME3**	**ME6**	**ME15**	**IME3**	**IME6**	**IME15**	**3dc**	**6dc**	**15dc**	**6/3**	**15/6**	**6/3**	**15/6**	
tae-miR9657b-5p	0.25	0.19	0.80	1.91	1.76	0.87	2.92	3.19			−1.01		2.05	CDPK adapter protein 2-like
tae-miR9775	0.08	0.29	1.47	1.37	1.68	0.97	4.03	2.54				1.79	2.34	Resistance protein T10rga2-1A
tae-miR444a	0.08	0.10	0.13	0.46	0.42	0.19	2.44	2.12			−1.11			MADS-box transcription factor
tae-miR6201	5.63	4.06	2.00	12.43	14.61	1.65	1.14	1.85			−3.15		−1.02	
tae-miR9782	1.51	1.55	2.00	4.19	5.37	2.04	1.47	1.80			−1.40			
zma-miR156k-5p	2.77	5.31	4.93	8.69	14.61	3.39	1.65	1.46			−2.11			SBP-like protein
tae-miR171b	0.42	0.48	1.87	1.22	1.26	2.33	1.54	1.38					1.95	6,7-dimethyl-8-ribityllumazine synthase
sbi-miR1432	5.29	4.05	3.60	13.86	9.91	4.07	1.39	1.29			−1.28			Calcium-transporting ATPase
tae-miR1137a	1.76	1.74	1.47	3.66	4.20	1.07	1.05	1.27			−1.98			Expansion EXPB8
tae-miR399	0.50	1.45	0.53	5.11	3.44	0.78	3.34	1.25			−2.15	1.52	−1.44	Disease resistance RPP13-like protein
rco-miR156c	210.34	361.73	390.72	500.51	767.78	283.52	1.25	1.09			−1.44			SBP-like protein
tae-miR156	246.56	423.25	482.53	579.55	877.63	354.52	1.23	1.05			−1.31			SBP-like protein
tae-miR9657a-3p	0.25	0.48	0.40	0.69	0.84	0.68	1.44							Glycosyl transferase
tae-miR395a	0.08	0.10	0.27	0.23	0.08	0.10	1.44		−1.46	−1.45			1.46	Ribosomal protein L19
tae-miR164	30.17	51.28	28.52	76.07	71.81	29.78	1.33				−1.27			NAC transcription factor
tae-miR1139	2.18	2.51	1.87	4.80	4.87	3.49	1.14							Transcription factor Myb1
tae-miR9774	0.42	1.35	2.00	1.45	0.76	0.29	1.79		−2.78		−1.38	1.69		Resistance protein T10rga2-1A

*ME3, ME6, and ME15 are mature embryos at 3, 6, and 15 d of culture (dc), respectively*.

*IME3, IME6, and IME15 are immature embryos at 3, 6, and 15 d of culture, respectively*.

**Table 6:** **The down-regulated known miRNAs in IME vs. ME and their target function**.

	**Transcripts per million (TPM)**						**Fold-change**							**Target function**
							**IME vs. ME**			**IME vs. IME**		**ME vs. ME**		
	**ME3**	**ME6**	**ME15**	**IME3**	**IME6**	**IME15**	**3 dc**	**6 dc**	**15 d**	**6/3**	**15/6**	**6/3**	**15/6**	
mdm-miR171i	13.5	19.8	30.2	0.5	7.2	16.3	−4.7	−1.5		3.8	1.2			TRIUR3_13261
tae-miR9664-3p	254.0	278.3	275.7	22.1	81.2	57.3	−3.5	−1.8	−2.3	1.9				Alternative splicing regulator
tae-miR531	3.0	3.2	5.9	0.5	1.7	2.4	−2.5		−1.3	1.7				Wpk4 protein kinase
tae-miR398	2.9	3.0	4.8	0.5	3.6	6.8	−2.5			2.8				Superoxide dismutase
tae-miR397-5p	46.8	157.4	53.6	9.0	60.0	151.2	−2.4	−1.4	1.5	2.7	1.3	1.7	−1.6	
tae-miR408	1.95	3.99	4.13	0.97	2.35	5.53	−1.0			1.3	1.2	1.0		Resistance protein RGA1R
tae-miR6197-5p	0.3	0.5	0.1	0.1	0.3	0.2	−1.7			1.7			−1.9	Cell Division Protein
ata-miR396b-5p	10140.3	12059.3	4155.4	3345.8	5992.4	4647.9	−1.6	−1.0					−1.5	Growth-regulating factor
tae-miR9653b	5.1	5.6	7.2	1.9	5.5	4.4	−1.4			1.5				
tae-miR9773	323.3	464.3	471.6	131.6	315.4	362.1	−1.3			1.3				
tae-miR9778	12.3	8.2	8.3	5.3	5.1	1.1	−1.2		−3.0		−2.3			Resistance protein
hvu-miR1120	12.9	22.3	13.7	5.9	14.7	11.1	−1.1			1.3				Resistance-related RLK
tae-miR169	0.3	0.2	0.3	0.2	0.5	0.1	−1.1	1.4	−1.5	1.7	−2.4			
tae-miR1120b-3p	4.5	10.2	4.8	3.1	5.0	3.2		−1.0				1.2	−1.1	MADS-box TF
vvi-miR171e	546.9	1025.1	469.2	609.5	287.7	356.4		−1.8		−1.1			−1.1	
tae-miR159a	1325.5	1886.7	1430.0	723.2	788.4	1241.9		−1.3						Myb3 TF
tae-miR5049-3p	0.3	1.4	1.6	0.3	0.7	0.4		−1.1	−2.0	1.1		2.1		Photosystem 1 subunit 5
tae-miR9776	6.4	12.8	7.6	8.7	11.8	3.4			−1.2		−1.8	1.0		Transcriptional factor B3-like
tae-miR9772	579.2	602.4	841.5	492.4	741.7	388.5			−1.1					ATPase-like
tae-miR9661-5p	2.8	4.3	8.8	2.3	3.6	1.0			−3.2		−1.9		1.02	Metallopeptidase family M24
gma-miR393j	7.1	5.9	5.3	11.6	8.9	1.7			−1.6		−2.4			Hypothetical protein
tae-miR1137b-5p	2.4	2.9	3.7	2.1	3.8	1.8			−1.0		−1.0			
tae-miR9670-3p	9.1	8.7	6.0	8.9	3.2	1.7		−1.4	−1.8	−1.5				Non-specific LTP precursor

*ME3, ME6, and ME15 are mature embryos at 3, 6, and 15 d of culture (dc), respectively*.

*IME3, IME6, and IME15 are immature embryos at 3, 6, and 15 d of culture, respectively*.

**Table 7:** **Up-regulated and non-differentially expressed known miRNAs at 3 days of culture and their target function**.

**miRNA**	**Transcripts per million (TPM)**						**Fold-change**							**Target function**
							**IME vs. ME**			**IME vs. IME**		**ME6 vs. ME**		
	**ME3**	**ME6**	**ME15**	**IME3**	**IME6**	**IME15**	**3 dc**	**6 dc**	**15 dc**	**I6/3**	**15/6**	**6/3**	**15/6**	
tae-miR9654b-3p	40.2	52.3	28.7	31.9	50.9	77.0	−		1.4					PHD-finger
tae-miR1127b-3p	2.6	2.2	1.5	2.1	4.9	3.8	−	1.1	1.4	1.2				Non-specific LTP precursor
tae-miR9652-3p	2.7	1.4	2.5	2.1	2.8	1.1	−	1.0	−1.2		−1.4			Cyclic nucleotide gated channel
tae-miR171a	2.7	2.1	5.1	5.0	4.7	1.9	−	1.1	−1.4		−1.3		1.3	Cysteine proteinase-like protein
tae-miR9672b	7.5	6.7	16.1	8.8	15.4	8.9	−	1.2					1.3	Resistance protein
tae-miR7757-5p	3911.2	7299.3	9247.8	4527.2	16852.8	17156.7	−	1.2		1.9				AAA-type ATPase-like
tae-miR9663-5p	1.5	0.8	0.7	1.8	2.4	1.3	−	1.6						Transposase
tae-miR9658-3p	3.9	2.8	2.5	4.9	8.7	3.2	−	1.6			−1.4			Resistance protein
tae-miR9660-5p	0.6	0.5	0.4	0.5	1.6	0.3	−	1.7		1.8	−2.5			Heat shock protein
tae-miR1117	1.7	0.8	1.3	2.4	3.5	0.9	−	2.2			−2.0	−1.1		
tae-miR167b	1.4	0.7	1.3	1.7	1.7	1.1	−	1.3				−1.1		Histone H3.2
tae-miR1136	166.6	119.6	82.6	221.3	238.8	85.4	−	1.0			−1.5			Mitochondrial 2-oxoglutarate/ malate translocator

*ME3, ME6, and ME15 are mature embryos at 3, 6, and 15 d of culture (dc), respectively*.

*IME3, IME6, and IME15 are immature embryos at 3, 6, and 15 d of culture, respectively*.

**Table 8:** **Differentially expressed known miRNAs in stages and their target function**.

**miRNA**	**Transcripts per million (TPM)**						**Fold-change**					**Target function**
							**IME vs. IME**	**IME vs. IME**		**ME6 vs. ME15**		
	**ME3**	**ME6**	**ME15**	**IME3**	**IME6**	**IME15**		**6/3**	**16/6**	**6/3**	**15/6**	
ata-miR319-3p	5.7	8.2	8.4	2.9	9.3	10.7		1.7				Dihydrodipicolinate reductase1
tae-miR319	527.4	599.2	209.7	422.5	565.4	294.3					−1.5	
bdi-miR1878-3p	10.9	32.8	50.1	15.1	40.1	89.9		1.4	1.2	1.6		NBS-LRR resistance protein-like
tae-miR1121	4.1	3.5	4.8	3.0	6.2	2.5		1.0	−1.3			
tae-miR9679-5p	4.9	8.2	6.1	6.4	14.9	8.0		1.2				Elongation factor 1-gamma 2
tae-miR9678-3p	139.7	139.3	43.0	149.9	190.0	24.2			−3.0		−1.7	F-box protein-like
tae-miR9675-3p	10.3	5.2	2.3	9.1	3.9	1.4		−1.2	−1.5		−1.2	
tae-miR9674b-5p	2227.4	2379.7	2974.1	1363.2	2798.3	2710.8		1.0				
tae-miR9673-5p	7.3	8.8	7.6	9.5	12.7	6.3			−1.0			
tae-miR9669-5p	16.8	22.7	11.9	21.7	39.8	9.4			−2.1			
tae-miR9779	4.9	3.7	8.3	3.7	3.9	10.6			1.5		1.2	
tae-miR9777	22.1	34.3	46.9	14.3	20.0	40.5			1.0			
aly-miR166c-3p	354.4	290.8	112.9	372.7	302.1	58.9			−2.4		−1.4	HD-zipper protein Hox9
tae-miR395b	0.0	0.2	0.3	1.0	0.3	0.2		−1.6				Ribosomal protein L19
tae-miR1130b-3p	1.9	2.9	1.1	3.3	2.5	1.0			−1.4		−1.4	TaGA3ox2-3;
tae-miR1120a	0.3	0.9	0.9	0.4	1.1	0.6		1.5		1.4		Proteinase inhibitor Rgpi9

*ME3, ME6, and ME15 are mature embryos at 3, 6, and 15 d of culture (dc), respectively*.

*IME3, IME6, and IME15 are immature embryos at 3, 6, and 15 d of culture, respectively*.

**Figure 6:**
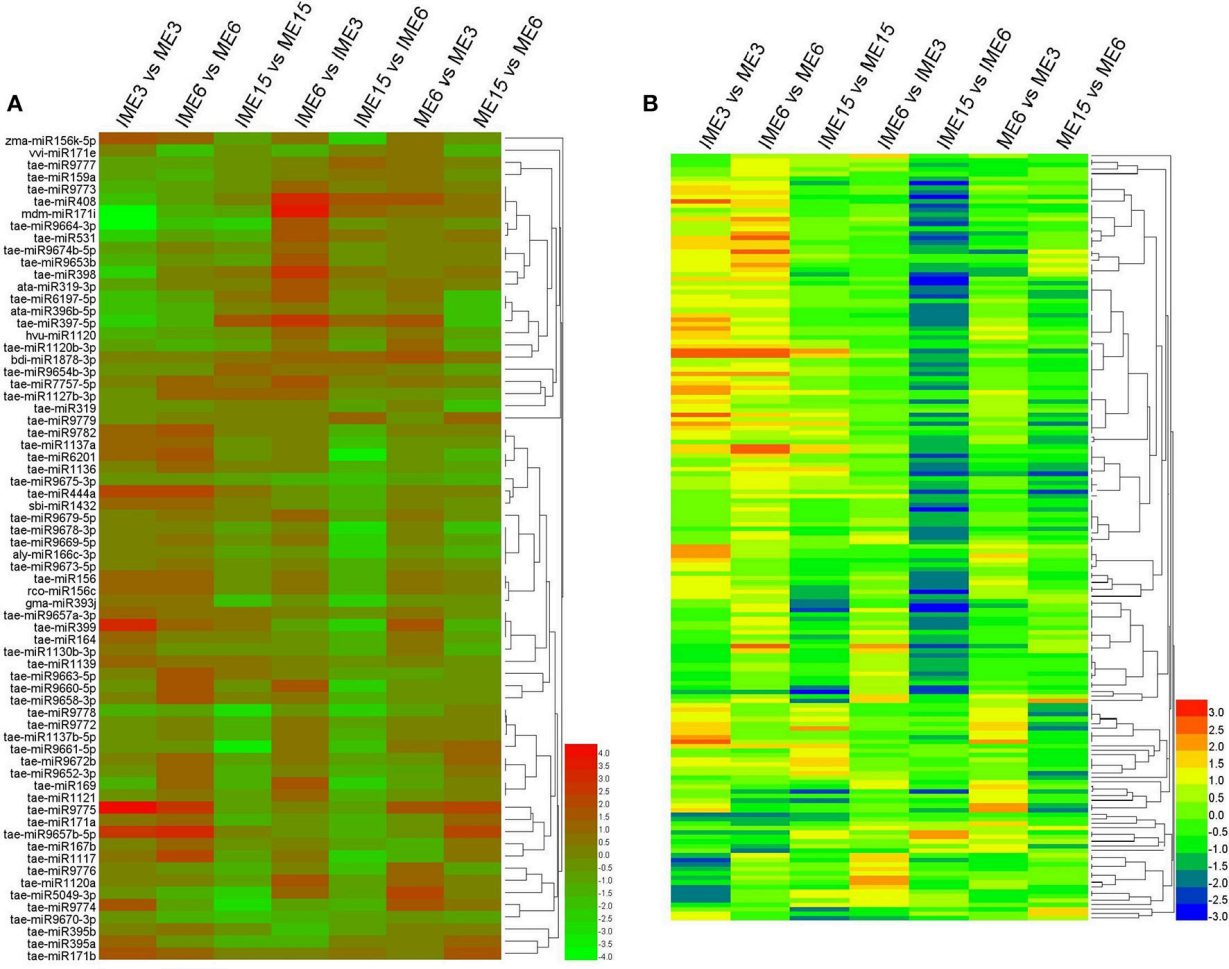
**Expression patterns of the differentially expressed known and novel miRNAs in developing callus based on deep-sequencing datasets**. Clustering was performed based on fold-changes between IME and ME at 3, 6, and 15 DC and between different development stages in IME and ME. **(A)** Known miRNAs; **(B)** Novel miRNAs. The bars represent the scale of the relative expression levels of miRNAs (MEAN centered).

To find common expression patterns of the differentially expressed miRNAs, we performed hierarchical clustering based on fold-changes between IME and ME and between the developmental stages of IME and ME. All the differentially expressed miRNAs in our comparison were used for clusters analysis including 68 known and 132 novel (including 11 reported novel) miRNAs (Figure 6).

**Figure 7:**
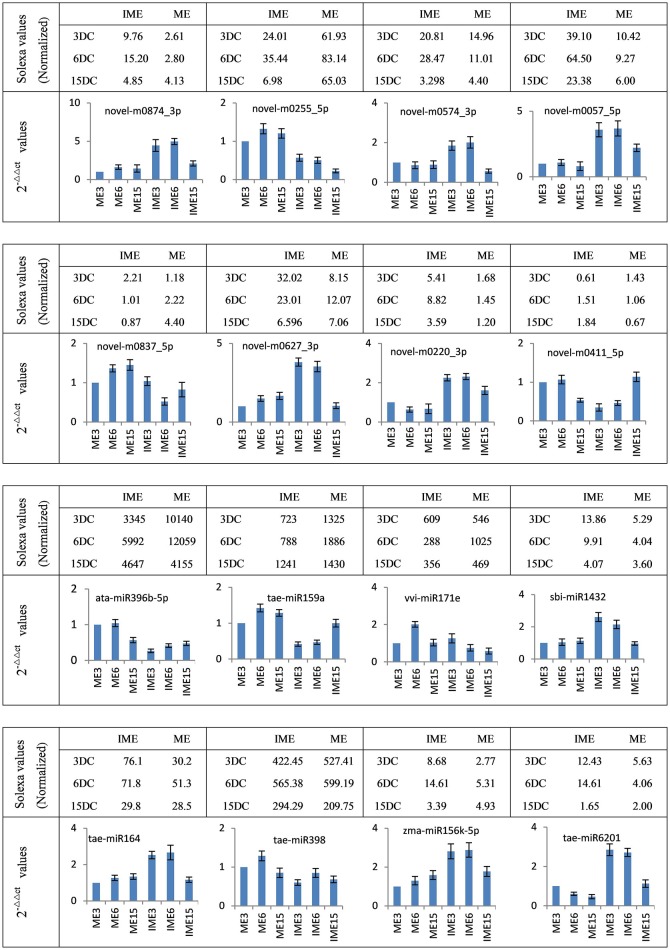
**Confirmation of the expression patterns of 16 (eight known and eight novel) miRNAs in developing callus**. miRNA expression data were normalized to wheat U6 small nuclear RNA gene [GenBank: X63066]. Experiments were performed in three biological replicates. Error bars represent one standard deviation (SD). Individual miRNA value is presented as fold-change (mean ± SD) compared to ME3 value set to 1.0.

To confirm miRNA expression at 3, 6, and 15 DC and validate the deep-sequencing results, eight known and eight novel (including one reported novel) miRNAs were selected randomly for quantitative real-time polymerase chain reaction (qRT-PCR). The expression patterns of these miRNAs were similar to those obtained from deep-sequencing, indicating that sRNA sequencing was reliable (Figure 7).

### Predicted and validated miRNA targets

Targets were predicted for the differentially expressed miRNA (Table S6), functional annotations of those predicted target genes performed by BLAST analysis found that these targets included mRNA coding for zinc finger protein, MYB protein, and SQUAMOSA promoter-binding protein-like (SPL); some miRNAs were also found to target transcription factors, including NAC, MADS-box, and auxin response factors (ARFs) (Table S6), some of which are important in regulation of plant development and embryogenic callus formation (Yang and Zhang, 2010). Some targets involved in biological processes such as meristem development (GO: 0048507), embryo development (GO: 0009790), embryo-sac development (GO: 0009553), auxin biosynthetic process (GO: 0009851), regulation of cell differentiation (GO: 0045595; GO: 0045597) and cell division (GO: 0051302). These results implied that miRNAs are involved in embryogenic callus formation. GO analysis of the differentially expressed miRNAs between the IME and ME libraries at 3, 6, and 15 DC classified the target genes according to their cellular component, molecular function, and biological processes. A total of 11 molecular functions were identified, of which binding, catalytic activity, and nucleic acid binding were the three most frequent functions (Image S3). For biological processes, 19 categories were identified, of which metabolic processes, cellular processes, and biological regulation were the three most frequent processes (Image S3).

**Figure 8:**
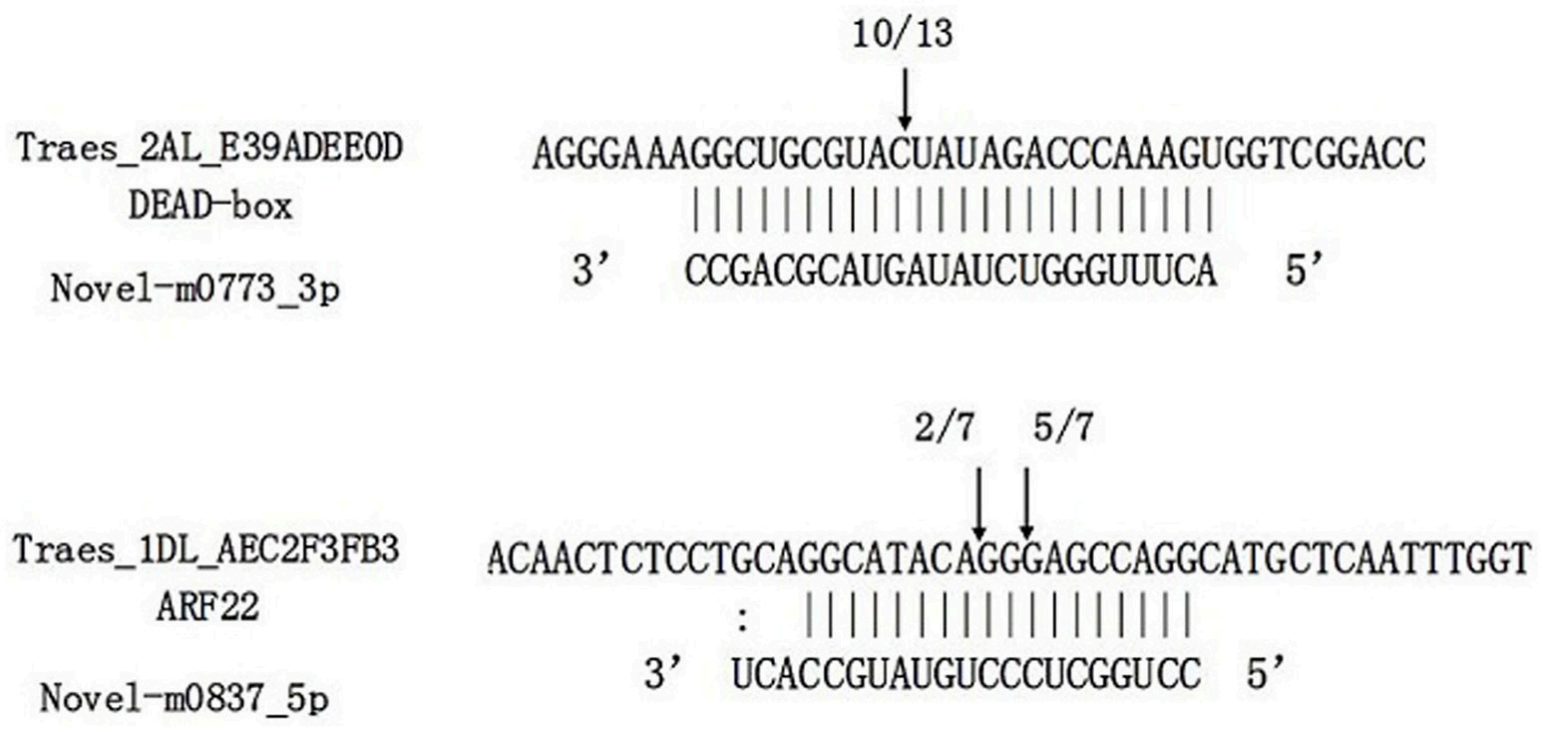
**Target validation of two novel miRNAs through RNA ligase-mediated rapid amplification of 5'complementary DNA ends (5′RLM-RACE)**. miRNA:mRNA alignment along with the cleavage frequencies of two novel miRNAs targets detected using 5′RACE. The arrow indicates the cleavage site and the number indicates the frequency of cloned RACE products. Vertical lines indicate matched base pairs and “**:**” symbolizes mismatches.

RNA ligation-mediated (RLM) rapid amplification of 5′ cDNA ends (RACE) was performed to determine the cleavage sites of the predicted targets for two target genes of novel miRNA, including an ARF gene for novel-m0837-5p and a gene for novel-m0773-3p. The results confirmed the predicted cleavage sites of these two genes (Figure 8), thus providing 5′RACE evidence for miRNAs regulating the predicted targets.

**Figure 9:**
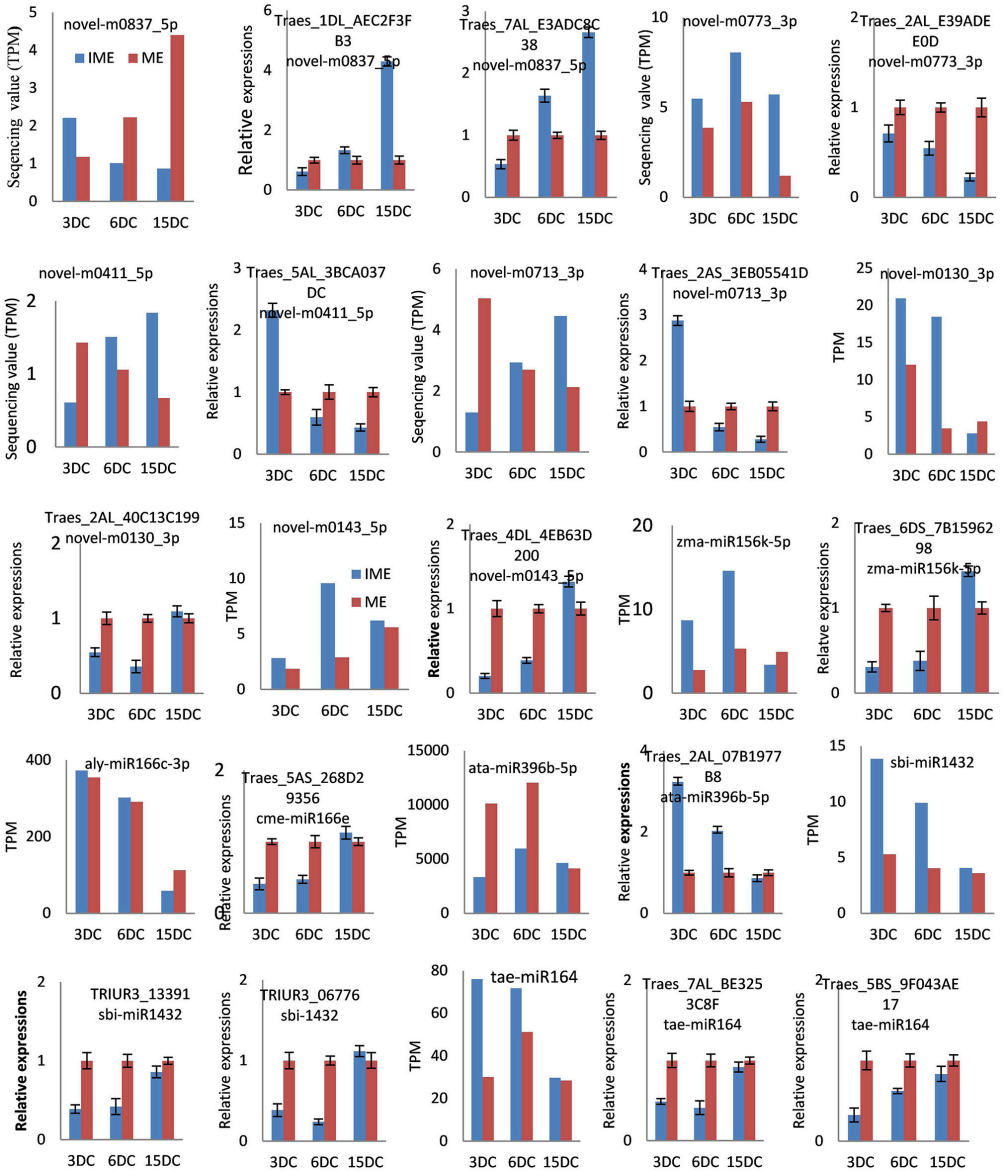
**Confirmation of the expression patterns of 14 miRNA targets in wheat developing callus**. Targets expression data were normalized to the actin gene (GenBank: AB181991). Experiments were repeated in triplicate. Error bars represent one standard deviation (SD). Individual targets value is presented as fold-change (mean ± SD) compared according to ME of 1.0.

Furthermore, to validate the expression levels of potential targets, 14 target genes of 11 miRNAs (five known and six novel) were selected for gene specific qRT-PCR. The expression analysis demonstrated that the target genes were negatively correlated to the expression of their corresponding miRNAs (Figure 9). These results implied that miRNAs are involved in the developmental processes of embryogenic callus formation in wheat by regulating the target genes expression.

## Discussion

Effective *in vitro* culture, which greatly depends on the induction of embryogenic callus (Zimmerman, 1993), plays an important role in plant genetic transformation. Therefore, low efficiency of tissue culture is a major limitation for gene function research and genetic improvement using transgenic technology in some crops. It is known that miRNAs take part in various developmental processes. Previous studies have identified miRNAs associated with development and stress response in bread wheat by sequencing sRNA populations or through computational strategies (Jin et al., 2008; Kantar et al., 2012). Recent studies have indicated that miRNAs were associated with embryogenic callus formation in many plants (Luo et al., 2006; Wu et al., 2011; Zhang et al., 2012; Yang et al., 2013). Furthermore, we investigated the differential expression of miRNAs associated with embryogenic callus formation in IMEs and MEs and analyzed the function of their targets in bread wheat.

In this study, we identified a set of miRNAs that were significantly up-regulated in IME vs. ME at 3 DC and/or 6 DC but down-regulated in IME15 vs. IME6, including three members of miR156 family, miR164, miR1432, tae-miR9657b-5p and several novel miRNAs. These miRNAs might be involved in embryogenic callus formation. Research in citrus somatic embryogenesis showed that expressions of the miR156 and miR164 were significantly higher in embryogenic callus than in non-embryogenic callus (Wu et al., 2011), suggesting that these two miRNAs were involved in embryogenic callus formation. Study in larch showed that expression of the miR156 reached it minor and major peak level at early cotyledonary embryo and late cotyledonary embryo stage, respectively, and was in low level at middle cotyledonary embryo and before the late single embryo stages (Zhang et al., 2012).

Previous studies indicated that the miR156 directly repressed the expression of SBP-box transcription factors that played an important role in juvenile-to-adult transition in Arabidopsis, maize, rice, and wheat (Liu et al., 2008; Qin et al., 2008; Wang, 2014). Furthermore, miR156 played a vital role in controlling leaf development, apical dominance, and floral transition and development by targeting several members of SPL (Cardon et al., 1997; Xie et al., 2006). Research in Arabidopsis showed that high expression of miR156 in young plants prevented precocious flowering (Wang et al., 2009). Furthermore, miR156 was found to be intimately associated with embryogenic callus formation in rice (Luo et al., 2006), citrus (Wu et al., 2011), and larch (Zhang et al., 2012). In this study, miR156 expressed significantly higher in IME3 and IME6 than in MEs and IME15, 3 DC and 6 DC were the important time for embryogenic callus formation in IME, indicating that miRNA156 was possibly related to embryogenic callus formation in bread wheat.

It was reported that miR164 targeting NAC transcription factor family was a key regulator in diverse developmental processes, including embryonic, vegetative, floral, and lateral root development (Mallory et al., 2004). It also revealed that miR164 targeted transcription factor that regulated a group of genes important for cell differentiation and development in Larixleptolepis (Zhang et al., 2010). miR1432, which was predicted to target calcium-transporting ATPase 9, and tae-miR9657b-5p, which was predicted to target calcium-dependent protein kinase (CDPK), Ca^2+^ was suggested to play an intermediary role during plant embryogenesis (Anil and Rao, 2000; Kiselev et al., 2008; Yang and Zhang, 2010; Kiselev et al., 2012). In this study, miR164, miR1432, and tae-miR9657b-5p were all significantly up-regulated in IMEs at 3 DC and/or 6 DC compared to MEs but down-regulated in IME15 vs. IME6. Additionally, novel-m0868_3p was predicted to target the ethylene-responsive transcription factor that was involved in embryogenic callus formation in plants (Mantiri et al., 2008). novel-m0874_3p and novel-m0220_3p were predicted to target zinc finger CCCH domain-containing protein that was involved in somatic embryogenic callus formation (Li and Thomas, 1998). It suggested that miR164, miR1432, tae-miR9657b-5p as well as novel-m0868_3p, novel-m0874_3p and novel-m0220_3p might be involved in embryogenic callus formation in wheat.

In addition, a different expression pattern was shown to be down-regulated in IMEs vs. MEs at 3 DC and/or 6 DC but up-regulated in IME15 vs. IME6 for miR398, miR397 and miR159 (Table 6) and several novel miRNAs including novel-m0411_5p and novel-m0713_3p. Wu et al. (2011) showed that expressions of the miR398, miR397 and miR159 were lower in embryogenic callus than in non-embryogenic callus, and miR397 and miR159 expressed their peak level at GE (Globular-shaped somatic embryo), and miR398 at CE (Cotyledon-shaped somatic embryo). In larch, miR398, miR397 and miR159 reached their peak level at fully mature embryos stage (Zhang et al., 2012). Therefore, miR398, miR397 and miR159 were thought to be possibly involved in somatic embryogenesis.

miR398, which was predicted to target superoxide dismutase, was induced during oxidative stress (Sunkar et al., 2006). The transcript profiles of the oxidative stress response were associated with somatic embryos in soybean, the development of which depended on a balance between cell proliferation and cell death (Thibaud-Nissen et al., 2003). In this study, the miR398 was down-regulated in the IME vs. ME but up-regulated in the development stages. Therefore, low expression of miR398 at 3 DC and 6 DC in IME might increase super oxide dismutase expression as stress response to contribute to the embryogenic callus formation.

Laccases, a group of polyphenol oxidases that targeted by miR397 (Jones-Rhoades and Bartel, 2004; Sunkar and Zhu, 2004), were associated with lignification and thickening of the cell wall in secondary cell growth (Constabel et al., 2000). Previous studies showed that expression level of the miR397 was very low in mature organs such as leaf, flower and stem (Jones-Rhoades and Bartel, 2004; Sunkar and Zhu, 2004), and was found to strongly and almost specifically express in undifferentiated embryogenic calli when compared with other organs (sprout, young panicle and young seed) (Luo et al., 2006), indicating that the miR397 might play an important role in meristematic tissues. In this study, expression of the miR397 was significantly lower in IME than in ME, which was consistent with results of Wu et al. (2011). It suggested that high expression of the miR397 in ME contributed to degradation of laccase genes, which might result in thin-wall cell and the transparent callus in ME. On the other hand, low expression of miR397 in IME at 3 and 6 DC contributed to accumulation of laccases, leading to the thicken of cell walls in IME. Due to completion of somatic embryogenesis, expression of miR397 increased at 15DC and thus its target reduced.

MYB-like transcription factor as the target of miR159 was involved in gibberellin signaling and plant anther development (Allen et al., 2007). The down-regulated miR159 families in embryogenic callus were regulated by abscisic acid (ABA) revealing the underlying mechanism controlling the transformation of embryogenic competence (Zhang et al., 2010). In this study, miR159 were down-regulated in IME when compared to ME at 6 DC. Ma et al. (2015) showed that miR159 was also down-regulated in dehydration stress in bread wheat. Therefore, low expression of the miR159 in IME3 and IME6 possibly increased MYB-like transcription factor expression as stress response to contribute to the embryogenic callus formation. Additionally, miR1139 was up-regulated at 3 DC and was predicted to target the transcription factor Myb1, perhaps through a complex regulation of the embryogenic callus formation. miR169, which targets the CCAAT-box transcription factor, might enhance dehydration stress tolerance by influencing ABA-responsive transcription in wheat (Ma et al., 2015). Embryogenic callus formation was regulated by ABA and physiological responses that were directly controlled by ABA stresses (Kikuchi et al., 2006). Growth regulator factor was considered as an important actor for the embryogenic callus initiation (Quiroz-Figueroa et al., 2006). In this study, miR396b-5p was predicted to target growth regulator factor and might be associated with embryogenic callus formation.

Auxin has a highly significant effect on callus and plantlet production. ARFs are transcription factors involved in auxin signal transduction during many stages of plant growth development, and they are considered to be critical for initiating cell division and differentiation of already differentiated cells before they can express embryogenic competence (Su and Zhang, 2009; Zhao et al., 2010), and ARF comprises a class of genes targeted by the miR167 families. ARF8 was regulated by the miR167 in cultured rice cells (Yang et al., 2006). It was shown that when cells grew in auxin-free medium, miR167 level decreased and when grew in the medium containing auxin showed a reversed trend (Yang et al., 2006). This proposed miRNA-auxin signal transduction pathway could be in conjunction with other miRNA-mediated auxin signals for responding to exogeneous auxin and determining the cellular auxin level to guide auxin responses. In this study, the miR167 differentially expressed in low abundance. In addition, two novel miRNAs (novel-m0837_5p and novel-m0143_5p) were predicted to be associated with auxin, and both of them were significantly down-regulated during embryogenic callus development in IME. Furthermore, novel-m0837_5p was down-regulated in IME vs. ME and novel-m0143_5p was up-regulated in IME vs. ME. It suggested that miR167, novel-m0837_5p and novel-m0143_5p might be involved in embryogenic callus development.

Some miRNAs, such as miR166, tae-miR1130b-3p, tae-miR1120a, and ata-miR319-3p, were only differentially expressed in comparisons between stages of IME and/or ME. Previous studies showed that miR166 targeted homeobox-leucine zipper III transcription factor genes involved in meristem formation, lateral organ polarity, and cell differentiation (Du et al., 2011; Lakhotia et al., 2014). In Arabidopsis, miR166 was thought to play a critical role in the development and maintenance of embryonic shoot apical meristem and the polarity of leaves (Liu et al., 2009; Zhou et al., 2015). In our study, miR166 was down-regulated between the development stages of IME and ME, and therefore it may be involved in regulation of callus development.

The results also showed that miR399 targeted putative disease resistance RPP13-like protein, a glycine-rich RNA-binding protein involved in embryo sac development. Some miRNAs that were predicted to target resistance proteins such as tae-miR9774 and tae-miR9775 were up-regulated in IME vs. ME, whereas others such as hvu-miR1120, tae-miR9778, and tae-miR408 were down-regulated. Stress was the important factor in embryogenic callus formation and somatic embryogenesis (Jin et al., 2014).

## Conclusions

We retrieved embryogenic and non-embryogenic callus from IMEs and MEs, respectively, using *in vitro* culture. Sequencing identified 85 known and 171 novel miRNAs in bread wheat. Based on differentially expressed miRNAs and target prediction, putative target genes for 30 (17 up-regulated), 33 (24 up-regulated), and 18 (3 up-regulated) differentially expressed known miRNAs and 69 (62 up-regulated), 67 (59 up-regulated), and 37 (21 up-regulated) differentially expressed novel miRNAs were predicted at 3, 6, and 15 DC, respectively. Targets function analysis indicated that some miRNA families, such as miR156, miR164, miR1432, miR398, miR397 and some novel miRNAs, play important roles in callus formation. Our data provide a useful resource for further study on embryogenic callus formation in wheat.

## Author contributions

DC designed and executed the study. ZC and ZD cultivated the wheat plantlets and callus derived from IMEs and MEs. ZC and FC extracted the RNA, preformed the experiments, carried out bioinformatics analysis, and wrote the draft of the manuscript. JC and HX supervised the project, supported experimental design and analysis, and critically reviewed the manuscript. All authors read, revised, and approved the final manuscript.

### Conflict of interest statement

The authors declare that the research was conducted in the absence of any commercial or financial relationships that could be construed as a potential conflict of interest. The reviewer JT declared a shared affiliation, though no other collaboration with the authors to the handling Editor, who ensured that the process nevertheless met the standards of a fair and objective review.

## Supplementary material

The data set, including the raw sequencing data of six small RNA libraries supporting the results presented herein, is available in SNBI SRA database under accession numbers: (accession: SRP068965).

Image S1 **Secondary structure of a part of known miRNA**. Detailed information includes locus sequences, abundance, precursor miRNA sequences, and structure of novel miRNAs.

Image S2 **Secondary structure of a set of novel miRNAs**.

Image S3 **Gene ontology of predicted targets for all differentially expressed miRNAs**. Categorization of miRNA-target genes was performed according to cellular components, molecular functions, and biological processes.

Table S1 **Annotation and distribution of small RNAs (sRNAs) in six libraries derived from callus of mature and immature embryos at 3, 6, or 15 d of culture**. Classifications for all sRNAs detected in six libraries. 3, 6, and 15 DC are 3, 6, and 15 d of culture, respectively.

Table S2 **Known miRNAs in six small RNA libraries derived from callus of mature and immature embryos at 3, 6, or 15 d of culture. S2-1**: known miRNAs; **S2-2**: detailed locus information, precursor miRNA sequences, and structure of 17 known miRNAs.

Table S3 **Detailed information of novel miRNAs in six small RNA libraries derived from callus of mature and immature embryos at 3, 6, or 15 d of culture**. Detailed information includes locus sequences, abundance, precursor miRNA sequences, and structure of novel miRNAs.

Table S4 **Other reported miRNAs in wheat**.

Table S5 **Detailed information of the differentially expressed known and novel miRNAs during callus development**. Detailed information includes sequences, abundance, and fold change of differentially expressed miRNAs.

Table S6 **Target annotation of the differentially expressed known and novel miRNAs**. Categorization of miRNA-target genes was performed according to cellular components, molecular functions, and biological processes.

Table S7 **Primers used in this study for qRT-PCR**.

Table S8 **Primers used in this study for 5′ RLM-RACE**.

## References

[B1] Anil V. S. Rao K. S. (2000). Calcium-mediated signaling during sandalwood somatic embryogenesis. role for exogenous calcium as second messenger. Plant Physiol. 123, 1301–1311. 10.1104/pp.123.4.1301 10938349 PMC59089

[B2] Allen R. S. Li J. Stahle M. I. Dubroué A. Gubler F. Millar A. A. (2007). Genetic analysis reveals functional redundancy and the major target genes of the Arabidopsis miR159 family. Proc. Natl. Acad. Sci. U.S.A. 104, 16371–16376. 10.1073/pnas.0707653104 17916625 PMC2042213

[B3] Audic S. Claverie J. M. (1997). The significance of digital gene expression profiles. Genome Res. 7, 986–995. 9331369 10.1101/gr.7.10.986

[B4] Bartel D. P. (2004). MicroRNAs: genomics, biogenesis, mechanism, and function. Cell 116, 281–297. 10.1016/S0092-8674(04)00045-5 14744438

[B5] Brenchley R. Spannagl M. Pfeifer M. Barker G. L. D'amore R. Allen A. M.. (2012). Analysis of the bread wheat genome using whole-genome shotgun sequencing. Nature 491, 705–710. 10.1038/nature11650 23192148 PMC3510651

[B6] Cardon G. H. Höhmann S. Nettesheim K. Saedler H. Huijser P. (1997). Functional analysis of the Arabidopsis thaliana SBP-box gene SPL3: a novel gene involved in the floral transition. Plant J. 12, 367–377. 10.1046/j.1365-313X.1997.12020367.x 9301089

[B7] Chen C.-J. Liu Q. Zhang Y.-C. Qu L.-H. Chen Y.-Q. Gautheret D. (2014). Genome-wide discovery and analysis of microRNAs and other small RNAs from rice embryogenic callus. RNA Biol. 8, 538–547. 10.4161/rna.8.3.15199 21525786

[B8] Chen X. (2005). MicroRNA biogenesis and function in plants. FEBS Lett. 579, 5923–5931. 10.1016/j.febslet.2005.07.071 16144699 PMC5127707

[B9] Constabel C. P. Yip L. Patton J. J. Christopher M. E. (2000). Polyphenol oxidase from hybrid poplar. Cloning and expression in response to wounding and herbivory. Plant Physiol. 124, 285–295. 10.1104/pp.124.1.285 10982443 PMC59143

[B10] De Felippes F. F. (2013). Downregulation of plant genes with miRNA-induced gene silencing. Methods Mol. Biol. 942, 379–387. 10.1007/978-1-62703-119-6_20 23027062

[B11] Delporte F. Mostade O. Jacquemin J. M. (2001). Plant regeneration through callus initiation from thin mature embryo fragments of wheat. Plant Cell Tissue Organ Cult. 67, 73–80. 10.1023/A:1011697316212

[B12] Delporte F. Pretova A. Du Jardin P. Watillon B. (2014). Morpho-histology and genotype dependence of *in vitro* morphogenesis in mature embryo cultures of wheat. Protoplasma 251, 1455–1470. 10.1007/s00709-014-0647-7 24763701 PMC4209243

[B13] Deng P. Nie X. Wang L. Cui L. Liu P. Tong W. (2014). Computational identification and comparative analysis of miRNAs in wheat group 7 chromosomes. Plant Mol. Biol. Rep. 32, 487–500. 10.1007/s11105-013-0669-x

[B14] Du J. Miura E. Robischon M. Martinez C. Groover A. (2011). The Populus Class III HD ZIP transcription factor POPCORONA affects cell differentiation during secondary growth of woody stems. PLoS ONE 6:e17458. 10.1371/journal.pone.0017458 21386988 PMC3046250

[B15] Fahlgren N. Howell M. D. Kasschau K. D. Chapman E. J. Sullivan C. M. Cumbie J. S.. (2007). High-throughput sequencing of Arabidopsis microRNAs: evidence for frequent birth and death of MIRNA genes. PLoS ONE 2:e219. 10.1371/journal.pone.0000219 17299599 PMC1790633

[B16] Filippov M. Miroshnichenko D. Vernikovskaya D. Dolgov S. (2006). The effect of auxins, time exposure to auxin and genotypes on somatic embryogenesis from mature embryos of wheat. Plant Cell Tissue Organ Cult. 84, 213–222. 10.1007/s11240-005-9026-6

[B17] Gardner P. P. Daub J. Tate J. G. Nawrocki E. P. Kolbe D. L. Lindgreen S.. (2009). Rfam: updates to the RNA families database. Nucleic Acids Res. 37, D136–D140. 10.1093/nar/gkn766 18953034 PMC2686503

[B18] Han R. Jian C. Lv J. Yan Y. Chi Q. Li Z.. (2014). Identification and characterization of microRNAs in the flag leaf and developing seed of wheat (*Triticum aestivum* L.). BMC Genomics 15:289. 10.1186/1471-2164-15-289 24734873 PMC4029127

[B19] Jeong D. H. Park S. Zhai J. Gurazada S. G. De Paoli E. Meyers B. C.. (2011). Massive analysis of rice small RNAs: mechanistic implications of regulated microRNAs and variants for differential target RNA cleavage. Plant Cell 23, 4185–4207. 10.1105/tpc.111.089045 22158467 PMC3269859

[B20] Jia J. Zhao S. Kong X. Li Y. Zhao G. He W.. (2013). Aegilops tauschii draft genome sequence reveals a gene repertoire for wheat adaptation. Nature 496, 91–95. 10.1038/nature12028 23535592

[B21] Jin F. Hu L. Yuan D. Xu J. Gao W. He L.. (2014). Comparative transcriptome analysis between somatic embryos (SEs) and zygotic embryos in cotton: evidence for stress response functions in SE development. Plant Biotechnol. J. 12, 161–173. 10.1111/pbi.12123 24112122

[B22] Jin W. Li N. Zhang B. Wu F. Li W. Guo A.. (2008). Identification and verification of microRNA in wheat (*Triticum aestivum*). J. Plant Res. 121, 351–355. 10.1007/s10265-007-0139-3 18357413

[B23] Jones-Rhoades M. W. Bartel D. P. (2004). Computational identification of plant microRNAs and their targets, including a stress-induced miRNA. Mol. Cell 14, 787–799. 10.1016/j.molcel.2004.05.027 15200956

[B24] Jones-Rhoades M. W. Bartel D. P. Bartel B. (2006). MicroRNAS and their regulatory roles in plants. Annu. Rev. Plant Biol. 57, 19–53. 10.1146/annurev.arplant.57.032905.105218 16669754

[B25] Kantar M. Akpinar B. A. Valarik M. Lucas S. J. Doležel J. Hernandez P.. (2012). Subgenomic analysis of microRNAs in polyploid wheat. Funct. Integr. Genomics 12, 465–479. 10.1007/s10142-012-0285-0 22592659

[B26] Kapitonov V. V. Jurka J. (2008). A universal classification of eukaryotic transposable elements implemented in Repbase. Nat. Rev. Genet. 9, 411–412. author reply: 414. 10.1038/nrg2165-c1 18421312

[B27] Kikuchi A. Sanuki N. Higashi K. Koshiba T. Kamada H. (2006). Abscisic acid and stress treatment are essential for the acquisition of embryogenic competence by carrot somatic cells. Planta 223, 637–645. 10.1007/s00425-005-0114-y 16160844

[B28] Kiselev K. V. Gorpenchenko T. Y. Tchernoded G. K. Dubrovina A. S. Grishchenko O. V. Bulgakov V. P.. (2008). Calcium-dependent mechanism of somatic embryogenesis in Panax ginseng cell cultures expressing the rolC oncogene. Mol. Biol. 42, 243–252. 10.1134/S0026893308020106 18610836

[B29] Kiselev K. V. Shumakova O. A. Manyakhin A. Y. Mazeika A. N. (2012). Influence of calcium influx induced by the calcium ionophore, A23187, on resveratrol content and the expression of CDPK and STS genes in the cell cultures of Vitis amurensis. Plant Growth Regul. 68, 371–381. 10.1007/s10725-012-9725-z

[B30] Kurihara Y. Watanabe Y. (2004). Arabidopsis micro-RNA biogenesis through Dicer-like 1 protein functions. Proc. Natl. Acad. Sci. U.S.A. 101, 12753–12758. 10.1073/pnas.0403115101 15314213 PMC515125

[B31] Kurtoglu K. Y. Kantar M. Lucas S. J. Budak H. (2013). Unique and conserved microRNAs in wheat chromosome 5D revealed by next-generation sequencing. PLoS ONE 8:e69801. 10.1371/journal.pone.0069801 23936103 PMC3720673

[B32] Lakhotia N. Joshi G. Bhardwaj A. R. Katiyar-Agarwal S. Agarwal M. Jagannath A.. (2014). Identification and characterization of miRNAome in root, stem, leaf and tuber developmental stages of potato (*Solanum tuberosum* L.) by high-throughput sequencing. BMC Plant Biol. 14:6. 10.1186/1471-2229-14-6 24397411 PMC3913621

[B33] Lee Y. Kim M. Han J. Yeom K. H. Lee S. Baek S. H.. (2004). MicroRNA genes are transcribed by RNA polymerase II. EMBO J. 23, 4051–4060. 10.1038/sj.emboj.7600385 15372072 PMC524334

[B34] Li H. Dong Y. Yin H. Wang N. Yang J. Liu X.. (2011). Characterization of the stress associated microRNAs in Glycine max by deep sequencing. BMC Plant Biol. 11:170. 10.1186/1471-2229-11-170 22112171 PMC3267681

[B35] Li Z. Thomas T. L. (1998). PEI1, an embryo-specific zinc finger protein gene required for heart-stage embryo formation in Arabidopsis. Plant Cell 10, 383–398. 10.2307/3870596 9501112 PMC143998

[B36] Lin Y. Lin L. Lai R. Liu W. Chen Y. Zhang Z.. (2015). MicroRNA390-directed TAS3 cleavage leads to the production of tasiRNA-ARF3/4 during somatic embryogenesis in dimocarpus longan lour. Front. Plant Sci. 6:1119. 10.3389/fpls.2015.01119 26734029 PMC4680215

[B37] Ling H. Q. Zhao S. Liu D. Wang J. Sun H. Zhang C.. (2013). Draft genome of the wheat A-genome progenitor *Triticum urartu*. Nature 496, 87–90. 10.1038/nature11997 23535596

[B38] Liu H. H. Tian X. Li Y. J. Wu C. A. Zheng C. C. (2008). Microarray-based analysis of stress-regulated microRNAs in Arabidopsis thaliana. RNA 14, 836–843. 10.1261/rna.895308 18356539 PMC2327369

[B39] Liu H. Qin C. Chen Z. Zuo T. Yang X. Zhou H.. (2014). Identification of miRNAs and their target genes in developing maize ears by combined small RNA and degradome sequencing. BMC Genomics 15:25. 10.1186/1471-2164-15-25 24422852 PMC3901417

[B40] Liu Q. Yao X. Pi L. Wang H. Cui X. Huang H. (2009). The ARGONAUTE10 gene modulates shoot apical meristem maintenance and leaf polarity establishment by repressing miR165/166 in Arabidopsis. Plant J. 58, 27–40. 10.1111/j.1365-313X.2008.03757.x 19054365

[B41] Livak K. J. Schmittgen T. D. (2001). Analysis of relative gene expression data using real-time quantitative PCR and the 2^−ΔΔC_T_^ method. Methods 25, 402–408. 10.1006/meth.2001.1262 11846609

[B42] Lucas S. J. Budak H. (2012). Sorting the wheat from the chaff: identifying miRNAs in genomic survey sequences of *Triticum aestivum* chromosome 1AL. PLoS ONE 7:e40859. 10.1371/journal.pone.0040859 22815845 PMC3398953

[B43] Luo Y. C. Zhou H. Li Y. Chen J. Y. Yang J. H. Chen Y. Q.. (2006). Rice embryogenic calli express a unique set of microRNAs, suggesting regulatory roles of microRNAs in plant post-embryogenic development. FEBS Lett. 580, 5111–5116. 10.1016/j.febslet.2006.08.046 16959252

[B44] Ma X. Xin Z. Wang Z. Yang Q. Guo S. Guo X.. (2015). Identification and comparative analysis of differentially expressed miRNAs in leaves of two wheat (*Triticum aestivum* L.) genotypes during dehydration stress. BMC Plant Biol. 15:21. 10.1186/s12870-015-0413-9 25623724 PMC4312605

[B45] Mallory A. C. Dugas D. V. Bartel D. P. Bartel B. (2004). MicroRNA regulation of NAC-domain targets is required for proper formation and separation of adjacent embryonic, vegetative, and floral organs. Curr. Biol. 14, 1035–1046. 10.1016/j.cub.2004.06.022 15202996

[B46] Mantiri F. R. Kurdyukov S. Lohar D. P. Sharopova N. Saeed N. A. Wang X. D.. (2008). The transcription factor MtSERF1 of the ERF subfamily identified by transcriptional profiling is required for somatic embryogenesis induced by auxin plus cytokinin in *Medicago truncatula*. Plant Physiol. 146, 1622–1636. 10.1104/pp.107.110379 18235037 PMC2287338

[B47] Meng F. Liu H. Wang K. Liu L. Wang S. Zhao Y.. (2013). Development-associated microRNAs in grains of wheat (*Triticum aestivum* L.). BMC Plant Biol. 13:140. 10.1186/1471-2229-13-140 24060047 PMC4015866

[B48] Meyers B. C. Axtell M. J. Bonnie B. Bartel D. P. David B. Bowman J. L.. (2008). Criteria for annotation of plant MicroRNAs. Plant Cell 20, 3186–3190. 10.1105/tpc.108.064311 19074682 PMC2630443

[B49] Murashige T. Skoog F. (1962). A revised medium for rapid growth and bio assays with tobacco tissue cultures. Physiol. Plant. 15, 473–497. 10.1111/j.1399-3054.1962.tb08052.x

[B50] Papp I. Mette M. F. Aufsatz W. Daxinger L. Schauer S. E. Ray A.. (2003). Evidence for nuclear processing of plant micro RNA and short interfering RNA precursors. Plant Physiol. 132, 1382–1390. 10.1104/pp.103.021980 12857820 PMC167078

[B51] Qin D. Wu H. Peng H. Yao Y. Ni Z. Li Z.. (2008). Heat stress-responsive transcriptome analysis in heat susceptible and tolerant wheat (*Triticum aestivum* L.) by using Wheat Genome Array. BMC Genomics 9:432. 10.1186/1471-2164-9-432 18808683 PMC2614437

[B52] Quiroz-Figueroa F. R. Rojas-Herrera R. Galaz-Avalos R. M. Loyola-Vargas V. M. (2006). Embryo production through somatic embryogenesis can be used to study cell differentiation in plants. Plant Cell Tissue Organ Cult. 86, 285–301. 10.1007/s11240-006-9139-6

[B53] Singla B. Tyagi A. K. Khurana J. P. Khurana P. (2007). Analysis of expression profile of selected genes expressed during auxin-induced somatic embryogenesis in leaf base system of wheat (*Triticum aestivum*) and their possible interactions. Plant Mol. Biol. 65, 677–692. 10.1007/s11103-007-9234-z 17849219

[B54] Song Q. X. Liu Y. F. Hu X. F. Zhang W. K. Ma B. Chen S. Y.. (2011). Identification of miRNAs and their target genes in developing soybean seeds by deep sequencing. BMC Plant Biol. 11:5. 10.1186/1471-2229-11-5 21219599 PMC3023735

[B55] Su Y. H. Zhang X. S. (2009). Auxin gradients trigger de novo formation of stem cells during somatic embryogenesis. Plant Signal. Behav. 4, 574–576. 10.4161/psb.4.7.8730 19820347 PMC2710545

[B56] Sun F. Guo G. Du J. Guo W. Peng H. Ni Z.. (2014). Whole-genome discovery of miRNAs and their targets in wheat (*Triticum aestivum* L.). BMC Plant Biol. 14:142. 10.1186/1471-2229-14-142 24885911 PMC4048363

[B57] Sunkar R. Kapoor A. Zhu J. K. (2006). Posttranscriptional induction of two Cu/Zn superoxide dismutase genes in Arabidopsis is mediated by downregulation of miR398 and important for oxidative stress tolerance. Plant Cell 18, 2051–2065. 10.1105/tpc.106.041673 16861386 PMC1533975

[B58] Sunkar R. Zhu J. K. (2004). Novel and stress-regulated microRNAs and other small RNAs from Arabidopsis. Plant Cell 16, 2001–2019. 10.1105/tpc.104.022830 15258262 PMC519194

[B59] Tanaka T. Kobayashi F. Joshi G. P. Onuki R. Sakai H. Kanamori H.. (2014). Next-generation survey sequencing and the molecular organization of wheat chromosome 6B. DNA Res. 21, 103–114. 10.1093/dnares/dst041 24086083 PMC3989483

[B60] Tao L.-L. Yin G.-X. Du L.-P. Shi Z.-Y. She M.-Y. Xu H.-J. (2011). Improvement of plant regeneration from immature embryos of wheat infected by *Agrobacterium tumefaciens*. Agric. Sci. China 10, 317–326. 10.1016/S1671-2927(11)60010-2

[B61] Thibaud-Nissen F. Shealy R. T. Khanna A. Vodkin L. O. (2003). Clustering of microarray data reveals transcript patterns associated with somatic embryogenesis in soybean. Plant Physiol. 132, 118–136. 10.1104/pp.103.019968 12746518 PMC166958

[B62] Wang J. W. (2014). Regulation of flowering time by the miR156-mediated age pathway. J. Exp. Bot. 65, 4723–4730. 10.1093/jxb/eru246 24958896

[B63] Wang J. W. Czech B. Weigel D. (2009). miR156-regulated SPL transcription factors define an endogenous flowering pathway in *Arabidopsis thaliana*. Cell 138, 738–749. 10.1016/j.cell.2009.06.014 19703399

[B64] Wong C. E. Zhao Y. T. Wang X. J. Croft L. Wang Z. H. Haerizadeh F.. (2011). MicroRNAs in the shoot apical meristem of soybean. J. Exp. Bot. 62, 2495–2506. 10.1093/jxb/erq437 21504877

[B65] Wu H. Sparks C. Amoah B. Jones H. D. (2003). Factors influencing successful Agrobacterium-mediated genetic transformation of wheat. Plant Cell Rep. 21, 659–668. 10.1007/s00299-002-0564-7 12789416

[B66] Wu X. M. Liu M. Y. Ge X. X. Xu Q. Guo W. W. (2011). Stage and tissue-specific modulation of ten conserved miRNAs and their targets during somatic embryogenesis of Valencia sweet orange. Planta 233, 495–505. 10.1007/s00425-010-1312-9 21103993

[B67] Xie K. Wu C. Xiong L. (2006). Genomic organization, differential expression, and interaction of SQUAMOSA promoter-binding-like transcription factors and microRNA156 in rice. Plant Physiol. 142, 280–293. 10.1104/pp.106.084475 16861571 PMC1557610

[B68] Xin M. Wang Y. Yao Y. Xie C. Peng H. Ni Z.. (2010). Diverse set of microRNAs are responsive to powdery mildew infection and heat stress in wheat (*Triticum aestivum* L.). BMC Plant Biol. 10:123. 10.1186/1471-2229-10-123 20573268 PMC3095282

[B69] Yang J. H. Han S. J. Yoon E. K. Lee W. S. (2006). ‘Evidence of an auxin signal pathway, microRNA167-ARF8-GH3, and its response to exogenous auxin in cultured rice cells’. Nucleic Acids Res. 34, 1892–1899. 10.1093/nar/gkl118 16598073 PMC1447648

[B70] Yang X. Wang L. Yuan D. Lindsey K. Zhang X. (2013). Small RNA and degradome sequencing reveal complex miRNA regulation during cotton somatic embryogenesis. J. Exp. Bot. 64, 1521–1536. 10.1093/jxb/ert013 23382553 PMC3617824

[B71] Yang X. Zhang X. (2010). Regulation of somatic embryogenesis in higher plants. Crit. Rev. Plant Sci. 29, 36–57. 10.1080/07352680903436291

[B72] Yanik H. Turktas M. Dundar E. Hernandez P. Dorado G. Unver T. (2013). Genome-wide identification of alternate bearing-associated microRNAs (miRNAs) in olive (*Olea europaea* L.). BMC Plant Biol. 13:10. 10.1186/1471-2229-13-10 23320600 PMC3564680

[B73] Yao Y. Guo G. Ni Z. Sunkar R. Du J. Zhu J. K.. (2007). Cloning and characterization of microRNAs from wheat (*Triticum aestivum* L.). Genome Biol. 8:R96. 10.1186/gb-2007-8-6-r96 17543110 PMC2394755

[B74] Yue L. Zhuo Z. Feng L. Wanwipa V. Qing J. Bairong S. (2012). Performance comparison and evaluation of software tools for microRNA deep-sequencing data analysis. Nucleic Acids Res. 40, 4298–4305. 10.1093/nar/gks043 22287634 PMC3378883

[B75] Zale J. M. Borchardt-Wier H. Kidwell K. K. Steber C. M. (2004). Callus induction and plant regeneration from mature embryos of a diverse set of wheat genotypes. Plant Cell Tissue Organ Cult. 76, 277–281. 10.1023/B:TICU.0000009248.32457.4c

[B76] Zhang B. Wang Q. Pan X. (2007). MicroRNAs and their regulatory roles in animals and plants. J. Cell. Physiol. 210, 279–289. 10.1002/jcp.20869 17096367

[B77] Zhang J. Zhang S. Han S. Wu T. Li X. Li W.. (2012). Genome-wide identification of microRNAs in larch and stage-specific modulation of 11 conserved microRNAs and their targets during somatic embryogenesis. Planta 236, 647–657. 10.1007/s00425-012-1643-9 22526500

[B78] Zhang S. Zhou J. Han S. Yang W. Li W. Wei H.. (2010). Four abiotic stress-induced miRNA families differentially regulated in the embryogenic and non-embryogenic callus tissues of Larix leptolepis. Biochem. Biophys. Res. Commun. 398, 355–360. 10.1016/j.bbrc.2010.06.056 20599742

[B79] Zhao Z. Andersen S. U. Ljung K. Dolezal K. Miotk A. Schultheiss S. J.. (2010). Hormonal control of the shoot stem-cell niche. Nature 465, 1089–1092. 10.1038/nature09126 20577215

[B80] Zhou Y. Honda M. Zhu H. Zhang Z. Guo X. Li T.. (2015). Spatiotemporal sequestration of miR165/166 by Arabidopsis Argonaute10 promotes shoot apical meristem maintenance. Cell Rep. 10, 1819–1827. 10.1016/j.celrep.2015.02.047 25801022

[B81] Zimmerman J. L. (1993). Somatic embryogenesis: a model for early development in higher plants. Plant Cell 5, 1411–1423. 10.1105/tpc.5.10.1411 12271037 PMC160372

