# Supplementary material for: Identification and Comparative Analysis of microRNA in Wheat (Triticum aestivum L.) Callus Derived from Mature and Immature Embryos during In vitro Culture
Source: Front Plant Sci. 2016 Aug 30;7:1302. doi: 10.3389/fpls.2016.01302 (PMC5003897; doi:10.3389/fpls.2016.01302)
Supplement: Image S1 — Secondary structure of a part of known miRNA. Detailed information includes locus sequences, abundance, precursor miRNA sequences, and structure of novel miRNAs. [file Image1.pdf]

Provisional ID : ta_iwgsc_5bl_v1_10899037_4961273
Score total : 2.9
Score for star read(s) : -1.3
Score for read counts : 0
Score for mfe : 2.6
Score for randfold : 1.6
Score for cons. seed :
Total read count : 16983
Mature read count : 17168
Loop read count : 0
Star read count : 0

aly-miR166c-3p

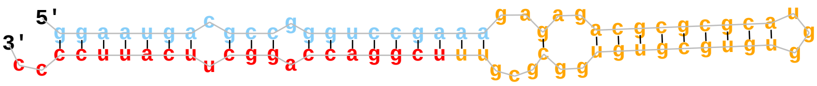

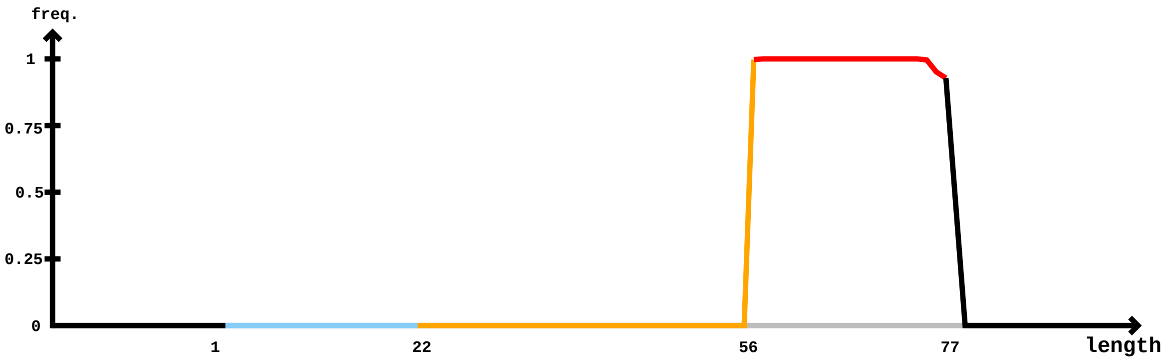

Star Mature

```
5'- gucaugguugucgaggggaaugacgccggguccgaaagagagacgcgcgcauggugugcguguggcgcguuucggaccaggcuucauuccccaugacuccaucaugguuca -3'   exp
    .(((((((.((((.(((((((((.(((.(((((((((..(..(((((((((...)))))))))..)...))))))))).))).))))))))).))))...)))))))....    reads  mm  sample
    ......................................................................uucggaccaggcuucauuccc....................    1      0   S01
    ......................................................................uucggaccaggcuucauuccc....................    1      0   S03
    .......................................................................ucggaccaggcuucauuc......................    13     0   S02
    .......................................................................ucggaccaggcuucauuc......................    16     0   S05
    .......................................................................ucggaccaggcuucauuc......................    3      0   S06
    .......................................................................ucggaccaggcuucauuc......................    12     0   S01
    .......................................................................ucggaccaggcuucauuc......................    18     0   S04
    .......................................................................ucggaccaggcuucauuc......................    5      0   S03
    .......................................................................ucggaccaggcuucauucc.....................    200    0   S04
    .......................................................................ucggaccaggcuucauucc.....................    89     0   S02
    .......................................................................ucggaccaggcuucauucc.....................    42     0   S03
    .......................................................................ucggaccaggcuucauucc.....................    46     0   S06
    .......................................................................ucggaccaggcuucauucc.....................    164    0   S01
    .......................................................................ucggaccaggcuucauucc.....................    213    0   S05
    .......................................................................ucggaccaggcuucauuccc....................    106    0   S04
    .......................................................................ucggaccaggcuucauuccc....................    52     0   S02
    .......................................................................ucggaccaggcuucauuccc....................    13     0   S03
    .......................................................................ucggaccaggcuucauuccc....................    16     0   S06
    .......................................................................ucggaccaggcuucauuccc....................    127    0   S01
    .......................................................................ucggaccaggcuucauuccc....................    65     0   S05
    .......................................................................ucggaccaggcuucauucccU...................    38     1   S02
    .......................................................................ucggaccaggcuucauucccc...................    3794   0   S01
    .......................................................................ucggaccaggcuucauucccc...................    526    0   S06
    .......................................................................ucggaccaggcuucauucccc...................    4427   0   S04
    .......................................................................ucggaccaggcuucauucccU...................    5      1   S03
    .......................................................................ucggaccaggcuucauucccU...................    48     1   S05
    .......................................................................ucggaccaggcuucauucccc...................    2784   0   S02
    .......................................................................ucggaccaggcuucauucccU...................    71     1   S04
    .......................................................................ucggaccaggcuucauucccU...................    49     1   S01
    .......................................................................ucggaccaggcuucauucccU...................    6      1   S06
    .......................................................................ucggaccaggcuucauucccc...................    3198   0   S05
    .......................................................................ucggaccaggcuucauucccc...................    765    0   S03
    .......................................................................ucggaccaggcuucauucccca..................    4      0   S05
    .......................................................................ucggaccaggcuucauucccca..................    3      0   S02
```

```
                Star                                                   Mature
gucaugguugucgaggggaaugacgccggguccgaaagagagacgcgcgcauggugugcguguggcgcguuucggaccaggcuucauuccccaugacuccaucaugguuca
.......................................................................ucggaccaggcuucauucccca..................    5    0    S01
.......................................................................ucggaccaggcuucauucccca..................    2    0    S03
.......................................................................ucggaccaggcuucauucccca..................    3    0    S06
.......................................................................ucggaccaggcuucauucccca..................    7    0    S04
.......................................................................ucggaccaggcuucauuccccau.................    1    0    S05
........................................................................cggaccaggcuucauucc.....................    1    0    S05
........................................................................cggaccaggcuucauucc.....................    5    0    S01
........................................................................cggaccaggcuucauucc.....................    3    0    S02
........................................................................cggaccaggcuucauucc.....................    1    0    S03
........................................................................cggaccaggcuucauuccc....................    2    0    S05
........................................................................cggaccaggcuucauuccc....................    1    0    S04
........................................................................cggaccaggcuucauuccc....................    2    0    S02
........................................................................cggaccaggcuucauuccc....................    1    0    S03
........................................................................cggaccaggcuucauuccc....................    7    0    S01
........................................................................cggaccaggcuucauucccc...................    8    0    S01
........................................................................cggaccaggcuucauucccc...................    6    0    S02
........................................................................cggaccaggcuucauucccc...................    3    0    S04
........................................................................cggaccaggcuucauucccc...................    3    0    S03
........................................................................cggaccaggcuucauucccc...................    2    0    S05
```

Provisional ID : ta_iwgsc_2bl_v1_7931332_1353700

ata-miR396b-5p

Score total : 212544.3
Score for star read(s) : 3.9
Score for read counts : 212536.5
Score for mfe : 2.2
Score for randfold : 1.6
Score for cons. seed :
Total read count : 416893
Mature read count : 416872
Loop read count : 1
Star read count : 9

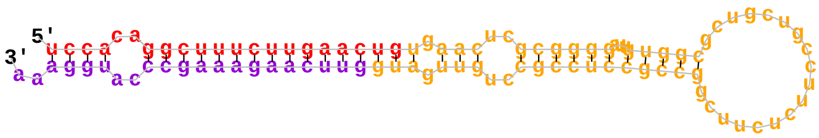

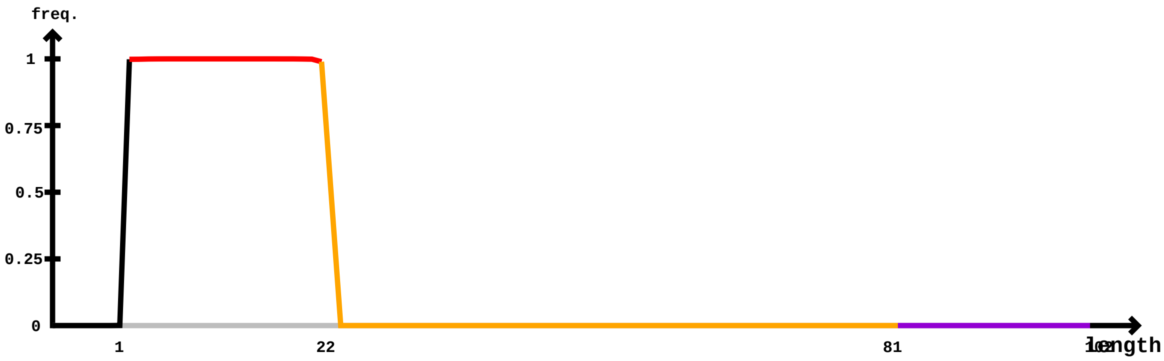

Mature **Star**

```
5'- augcucuccacaggcuuucuugaacugugaacucgcggggauuguggcgcugcugccuucucuucggccgccuccgccuguugaugguucaagaaagcccauggaaaccau  -3'   obs
    augcucuccacaggcuuucuugaacugugaacucgcggggauuguggcgcugcugccuucucuucggccgccuccgccuguugaugguucaagaaagcccauggaaaccau         exp
    ......((((..((((((((((((((((.(((..((((((...(((((..................)))))))))))..))).))))))))))))))))..)))).....   reads   mm   sample
    .ugcucuccacaggcuuucuug.........................................................................................   1       0    S01
    .ugcucuccacaggcuuucuug.........................................................................................   4       0    S05
    .ugcucuccacaggcuuucuug.........................................................................................   2       0    S04
    ....ucuccacaggcuuucuugaacug....................................................................................   2       0    S02
    ....ucuccacaggcuuucuugaacug....................................................................................   2       0    S01
    ....ucuccacaggcuuucuugaacug....................................................................................   2       0    S06
    ....ucuccacaggcuuucuugaacug....................................................................................   1       0    S04
    ....ucuccacaggcuuucuugaacug....................................................................................   1       0    S03
    .....cuccacaggcuuucuugaacu.....................................................................................   1       0    S06
    .....cuccacaggcuuucuugaacu.....................................................................................   2       0    S02
    .....cuccacaggcuuucuugaacug....................................................................................   44      0    S02
    .....cuccacaggcuuucuugaacug....................................................................................   54      0    S01
    .....cuccacaggcuuucuugaacug....................................................................................   12      0    S03
    .....cuccacaggcuuucuugaacug....................................................................................   5       0    S04
    .....cuccacaggcuuucuugaacug....................................................................................   12      0    S06
    .....cuccacaggcuuucuugaacug....................................................................................   13      0    S05
    .....cuccacaggcuuucuugaacugu...................................................................................   1       0    S02
    ......uccacaggcuuucuugaa.......................................................................................   7       0    S04
    ......uccacaggcuuucuugaa.......................................................................................   39      0    S02
    ......uccacaggcuuucuugaa.......................................................................................   47      0    S03
    ......uccacaggcuuucuugaa.......................................................................................   29      0    S06
    ......uccacaggcuuucuugaa.......................................................................................   24      0    S05
    ......uccacaggcuuucuugaa.......................................................................................   29      0    S01
    ......uccacaggcuuucuugaac......................................................................................   35      0    S05
    ......uccacaggcuuucuugaac......................................................................................   90      0    S02
    ......uccacaggcuuucuugaac......................................................................................   23      0    S06
    ......uccacaggcuuucuugaac......................................................................................   32      0    S03
    ......uccacaggcuuucuugaac......................................................................................   19      0    S04
    ......uccacaggcuuucuugaac......................................................................................   65      0    S01
    ......uccacaggcuuucuugaacu.....................................................................................   479     0    S06
    ......uccacaggcuuucuugaacu.....................................................................................   578     0    S05
    ......uccacaggcuuucuugaacG.....................................................................................   1       1    S04
    ......uccacaggcuuucuugaacG.....................................................................................   1       1    S05
```

Mature **Star**

augcucuccacaggcuuucuugaacugugaacucgcggggauuguggcgcugcugccuucucuucggccgccuccgccuguugaugguucaagaaagcccauggaaaccau			
......uccacaggcuuucuugaacu...	253	0	S04
......uccacaggcuuucuugaacG...	2	1	S03
......uccacaggcuuucuugaacG...	5	1	S06
......uccacaggcuuucuugaacu...	1214	0	S02
......uccacaggcuuucuugaacu...	958	0	S01
......uccacaggcuuucuugaacu...	487	0	S03
......uccacaggcuuucuugaacG...	5	1	S01
......uccacaggcuuucuugaacug..	44554	0	S06
......uccacaggcuuucuugaacGg..	77	1	S05
......uccacaggcuuucuugaacGg..	152	1	S02
......uccacaggcuuucuugaacGg..	47	1	S06
......uccacaggcuuucuugaacGg..	148	1	S01
......uccacaggcuuucuugaacug..	41167	0	S04
......uccacaggcuuucuugaacug..	116735	0	S02
......uccacaggcuuucuugaacGg..	43	1	S04
......uccacaggcuuucuugaacug..	28179	0	S03
......uccacaggcuuucuugaacug..	112980	0	S01
......uccacaggcuuucuugaacGg..	6	1	S03
......uccacaggcuuucuugaacug..	66638	0	S05
......uccacaggcuuucuugaacugu...	377	0	S01
......uccacaggcuuucuugaacugu...	206	0	S02
......uccacaggcuuucuugaacugu...	84	0	S03
......uccacaggcuuucuugaacugu...	71	0	S06
......uccacaggcuuucuugaacGgu...	1	1	S02
......uccacaggcuuucuugaacugu...	113	0	S05
......uccacaggcuuucuugaacugu...	84	0	S04
......uccacaggcuuucuugaacugug..	2	0	S06
.......ccacaggcuuucuugaac..	1	0	S05
.......ccacaggcuuucuugaac..	1	0	S01
.......ccacaggcuuucuugaac..	1	0	S02
.......ccacaggcuuucuugaacu...	3	0	S05
.......ccacaggcuuucuugaacu...	2	0	S02
.......ccacaggcuuucuugaacu...	1	0	S04
.......ccacaggcuuucuugaacu...	3	0	S03
.......ccacaggcuuucuugaacu...	4	0	S01
.......ccacaggcuuucuugaacug..	16	0	S06
.......ccacaggcuuucuugaacug..	35	0	S02
.......ccacaggcuuucuugaacug..	27	0	S01
.......ccacaggcuuucuugaacug..	16	0	S05
.......ccacaggcuuucuugaacug..	15	0	S03
.......ccacaggcuuucuugaacug..	10	0	S04
........cacaggcuuucuugaacu...	1	0	S01
........cacaggcuuucuugaacu...	1	0	S04
........cacaggcuuucuugaacu...	1	0	S06
........cacaggcuuucuugaacug..	44	0	S04
........cacaggcuuucuugaacug..	114	0	S02
........cacaggcuuucuugaacug..	68	0	S05
........cacaggcuuucuugaacug..	7	0	S03
........cacaggcuuucuugaacug..	87	0	S01
........cacaggcuuucuugaacug..	47	0	S06
........cacaggcuuucuugaacugu...	1	0	S06
........cacaggcuuucuugaacugu...	1	0	S02
.........acaggcuuucuugaacug..	8	0	S03
.........acaggcuuucuugaacug..	54	0	S05
.........acaggcuuucuugaacug..	13	0	S01
.........acaggcuuucuugaacug..	34	0	S06
.........acaggcuuucuugaacug..	7	0	S02
.........acaggcuuucuugaacug..	13	0	S04
..........caggcuuucuugaacugu...	1	0	S01
..........caggcuuucuugaacugu...	2	0	S06
..........caggcuuucuugaacugu...	3	0	S05
..........caggcuuucuugaacugug..	1	0	S03
..........caggcuuucuugaacugug..	1	0	S04
..............cuuucuugaacugugaacucg..	1	0	S01
...................uugaacugugaacucgcggg..	1	0	S05
....ccuccgccuguugaugguuca....................	1	0	S04
....uguugaugguucaagaaagcc............	2	0	S05
....uguugaugguucaagaaagcc............	1	0	S06
....guucaagaaagcccaugga......	2	0	S03
....guucaagaaagcccauggaa.....	1	0	S04

```
      Mature                                                                        Star
augcucuccacaggcuuucuugaacugugaacucgcggggauuguggcgcugcugccuucucuucggccgccuccgccuguugaugguucaagaaagcccauggaaaccau
......................................................................................guucaagaaagcccauggaa.....    1    0    S03
......................................................................................guucaagaaagcccauggaa.....    1    0    S06
......................................................................................guucaagaaagcccauggaaa....    2    0    S04
......................................................................................guucaagaaagcccauggaaa....    2    0    S03
........................................................................................ucaagaaagcccauggaaacc..    1    0    S01
```

Provisional ID	:	ta_iwgsc_6ds_v1_2084038_6398828
Score total	:	2.7
Score for star read(s)	:	-1.3
Score for read counts	:	0
Score for mfe	:	2.4
Score for randfold	:	1.6
Score for cons. seed	:	
Total read count	:	20336
Mature read count	:	20206
Loop read count	:	0
Star read count	:	276

ata-miR9674b-3p (5p)

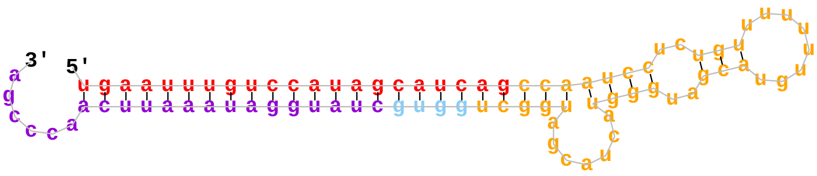

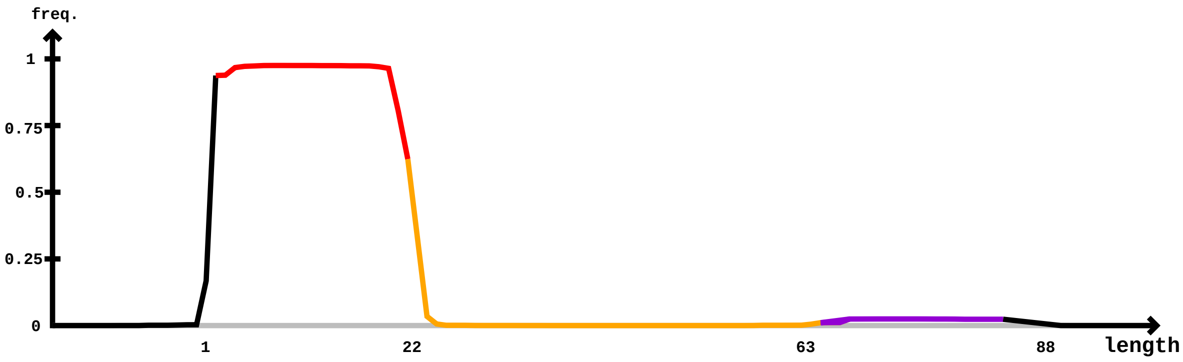

Mature Star

```
5'- agccaauccucugguugaauuuguccauagcaucagccaauccucuguuuuuuuguacgauggguacuacgauggcuggugcuauggauaaauucaacccgaugguugacaaa -3'  obs
    agccaauccucugguugaauuuguccauagcaucagccaauccucuguuuuuuuguacgauggguacuacgauggcugguggcuauggauaaauucaacccgaugguugacaaa       exp
    ...(((((.((.(((((((((((((((((((((((((((((((..(((........)))..)))).......))))))))))))))))))))))))))).)).))))).....      reads   mm   sample
    ..ccaauccucugguugaauuug..........................................................................................      1       0    S01
    .....auccucugguugaauuugucc.......................................................................................      1       0    S05
    ......uccucugguugaauuugucc.......................................................................................      1       0    S03
    ........cucugguugaauuugucc.......................................................................................      1       0    S02
    ........cucugguugaauuugucc.......................................................................................      3       0    S01
    ........cucugguugaauuugucc.......................................................................................      4       0    S05
    ........cucugguugaauuuguccau.....................................................................................      1       0    S03
    ........cucugguugaauuuguccaua....................................................................................      1       0    S02
    ........cucugguugaauuuguccaua....................................................................................      3       0    S06
    ........cucugguugaauuuguccaua....................................................................................      1       0    S04
    ........cucugguugaauuuguccaua....................................................................................      5       0    S05
    ........cucugguugaauuuguccaua....................................................................................      2       0    S01
    ..........cugguugaauuuguccauagc..................................................................................      1       0    S05
    ...........ugguugaauuuguccauagc..................................................................................      1       0    S05
    ...........ugguugaauuuguccauagc..................................................................................      1       0    S01
    ...........ugguugaauuuguccauagca.................................................................................      5       0    S01
    ...........ugguugaauuuguccauagca.................................................................................      1       0    S05
    ...........ugguugaauuuguccauagca.................................................................................      2       0    S02
    ...........ugguugaauuuguccauagca.................................................................................      2       0    S06
    ...........ugguugaauuuguccauagca.................................................................................      3       0    S04
    ...........ugguugaauuuguccauagca.................................................................................      1       0    S03
    ............gguugaauuuguccauagc..................................................................................      1       0    S03
    ............gguugaauuuguccauagc..................................................................................      2       0    S06
    ............gguugaauuuguccauagc..................................................................................      1       0    S02
    ............gguugaauuuguccauagca.................................................................................      1       0    S05
    ............gguugaauuuguccauagca.................................................................................      2       0    S04
    ............gguugaauuuguccauagca.................................................................................      1       0    S06
    ............gguugaauuuguccauagca.................................................................................      1       0    S02
    ............gguugaauuuguccauagca.................................................................................      2       0    S01
    ............gguugaauuuguccauagcau................................................................................      1       0    S02
    ............gguugaauuuguccauagcauc...............................................................................      1       0    S05
    ............gguugaauuuguccauagcauc...............................................................................      1       0    S03
    ............gguugaauuuguccauagcauc...............................................................................      1       0    S02
```

Mature / Star			
agccaauccucugguugaauuuguccauagcaucagccaauccucuguuuuuuuguacgauggguacuacgauggcugguggcuauggauaaauucaacccgaugguugacaaa			
............gguugaauuuguccauagcauc...	1	0	S06
............ggCugaauuuguccauagcauc...	1	1	S05
............gguugaauuuguccauagcauc...	2	0	S01
.............guugaauuuguccauagc..	1	0	S01
..............uugaauuuguccauagca...	6	0	S05
..............uugaauuuguccauagca...	8	0	S01
..............uugaauuuguccauagca...	5	0	S04
..............uugaauuuguccauagca...	10	0	S02
..............uugaauuuguccauagca...	9	0	S06
..............uugaauuuguccauagca...	8	0	S03
..............uugaauuuguccauagcau..	4	0	S01
..............uugaauuuguccauagcau..	6	0	S05
..............uugaauuuguccauagcau..	5	0	S02
..............uugaauuuguccauagcau..	2	0	S06
..............uugaauuuguccauagcau..	4	0	S03
..............uugaauuuguccauagcau..	6	0	S04
..............uugaauuuguccauagcauc...	210	0	S05
..............uugaauuuguccauagcauc...	123	0	S03
..............uugaauuuguccauagcauc...	289	0	S01
..............uugaauuuguccauagcauc...	137	0	S02
..............uugaauuuguccauagcauc...	161	0	S06
..............uugaauuuguccauagcauc...	181	0	S04
..............uugaauuuguccauagcauca..	238	0	S06
..............uugaauuuguccauagcauca..	467	0	S01
..............uugaauuuguccauagcauca..	326	0	S04
..............uugaauuuguccauagcauca..	372	0	S02
..............uugaauuuguccauagcauca..	265	0	S03
..............uugaauuuguccauagcauca..	450	0	S05
..............uugaauuuguccauagcaucag...	11	0	S02
..............uugaauuuguccauagcaucag...	9	0	S05
..............uugaauuuguccauagcaucag...	9	0	S01
..............uugaauuuguccauagcaucag...	6	0	S06
..............uugaauuuguccauagcaucag...	4	0	S04
..............uugaauuuguccauagcaucag...	5	0	S03
..............uugaauuuguccauagcaucagU..	2	1	S05
..............uugaauuuguccauagcaucagc..	1	0	S04
..............uugaauuuguccauagcaucagc..	1	0	S06
..............uugaauuuguccauagcaucagc..	1	0	S03
..............uugaauuuguccauagcaucagU..	1	1	S04
..............uugaauuuguccauagcaucagc..	2	0	S05
..............uugaauuuguccauagcaucagcc...	1	0	S03
..............uugaauuuguccauagcaucagcc...	10	0	S05
..............uugaauuuguccauagcaucagcc...	2	0	S01
..............uugaauuuguccauagcaucagcc...	6	0	S06
..............uugaauuuguccauagcaucagcc...	4	0	S04
..............uugaauuuguccauagcaucagcc...	4	0	S02
...............ugaauuuguccauagcau..	9	0	S05
...............ugaauuuguccauagcau..	7	0	S04
...............ugaauuuguccauagcau..	11	0	S06
...............ugaauuuguccauagcau..	27	0	S01
...............ugaauuuguccauagcau..	15	0	S03
...............ugaauuuguccauagcau..	26	0	S02
...............ugaauuuguccauagcauc...	315	0	S03
...............ugaauuuguccauagcauc...	350	0	S02
...............ugaauuuguccauagcauc...	571	0	S01
...............ugaauuuguccauagcauc...	177	0	S04
...............ugaauuuguccauagcauc...	395	0	S05
...............ugaauuuguccauagcauc...	331	0	S06
...............ugaauuuguccauagcauca..	201	0	S04
...............ugaauuuguccauagcauca..	309	0	S01
...............ugaauuuguccauagcauca..	220	0	S06
...............ugaauuuguccauagcauca..	274	0	S03
...............ugaauuuguccauagcauca..	191	0	S05
...............ugaauuuguccauagcauca..	362	0	S02
...............ugaauuuguccauagcaucag...	2524	0	S01
...............ugaauuuguccauagcaucag...	1031	0	S06
...............ugaauuuguccauagcaucag...	2656	0	S05
...............ugaauuuguccauagcaucag...	1985	0	S04
...............ugaauuuguccauagcaucag...	2164	0	S02
...............ugaauuuguccauagcaucag...	1281	0	S03

Mature   Star

```
agccaauccucugguugaauuuguccauagcaucagccaauccucuguuuuuuuguacgauggguacuacgauggcuggugcuauggauaaauucaacccgaugguugacaaa
...............ugaauuuguccauagcaucagc............................................................................  41   0   S03
...............ugaauuuguccauagcaucagc............................................................................  35   0   S02
...............ugaauuuguccauagcaucagc............................................................................  41   0   S04
...............ugaauuuguccauagcaucagc............................................................................  31   0   S06
...............ugaauuuguccauagcaucagc............................................................................  87   0   S05
...............ugaauuuguccauagcaucagc............................................................................  49   0   S01
...............ugaauuuguccauagcaucagcc...........................................................................  2    0   S06
...............ugaauuuguccauagcaucagcc...........................................................................  1    0   S02
...............ugaauuuguccauagcaucagcc...........................................................................  1    0   S05
...............ugaauuuguccauagcaucagcc...........................................................................  1    0   S01
...............ugaauuuguccauagcaucagcc...........................................................................  1    0   S04
...............ugaauuuguccauagcaucagcca..........................................................................  1    0   S02
...............ugaauuuguccauagcaucagcca..........................................................................  3    0   S06
...............ugaauuuguccauagcaucagcca..........................................................................  1    0   S04
...............ugaauuuguccauagcaucagcca..........................................................................  1    0   S03
...............ugaauuuguccauagcaucagcca..........................................................................  4    0   S05
................gaauuuguccauagcauc...............................................................................  1    0   S02
................gaauuuguccauagcauc...............................................................................  3    0   S01
................gaauuuguccauagcauc...............................................................................  2    0   S03
................gaauuuguccauagcauca..............................................................................  1    0   S04
................gaauuuguccauagcauca..............................................................................  3    0   S01
................gaauuuguccauagcauca..............................................................................  2    0   S03
................gaauuuguccauagcauca..............................................................................  1    0   S06
................gaauuuguccauagcaucag.............................................................................  4    0   S05
................gaauuuguccauagcaucag.............................................................................  1    0   S04
................gaauuuguccauagcaucag.............................................................................  1    0   S01
................gaauuuguccauagcaucag.............................................................................  2    0   S03
................gaauuuguccauagcaucag.............................................................................  1    0   S02
................gaauuuguccauagcaucagc............................................................................  1    0   S04
................gaauuuguccauagcaucagc............................................................................  1    0   S03
.................aauuuguccauagcauca..............................................................................  4    0   S05
.................aauuuguccauagcauca..............................................................................  4    0   S06
.................aauuuguccauagcauca..............................................................................  2    0   S01
.................aauuuguccauagcauca..............................................................................  1    0   S04
.................aauuuguccauagcauca..............................................................................  2    0   S02
.................aauuuguccauagcauca..............................................................................  7    0   S03
.................aauuuguccauagcaucag.............................................................................  6    0   S06
.................aauuuguccauagcaucag.............................................................................  7    0   S02
.................aauuuguccauagcaucag.............................................................................  3    0   S05
.................aauuuguccauagcaucag.............................................................................  3    0   S01
.................aauuuguccauagcaucag.............................................................................  14   0   S03
.................aauuuguccauagcaucagc............................................................................  1    0   S06
.................aauuuguccauagcaucagc............................................................................  1    0   S02
.................aauuuguccauagcaucagc............................................................................  2    0   S03
.................aauuuguccauagcaucagcc...........................................................................  77   0   S06
.................aauuuguccauagcaucagcc...........................................................................  91   0   S03
.................aauuuguccauagcaucagcc...........................................................................  85   0   S02
.................aauuuguccauagcaucagcc...........................................................................  124  0   S05
.................aauuuguccauagcaucagcc...........................................................................  65   0   S04
.................aauuuguccauagcaucagcc...........................................................................  82   0   S01
.................aauuuguccauagcaucagcca..........................................................................  1    0   S04
.................aauuuguccauagcaucagcca..........................................................................  1    0   S06
..................auuuguccauagcaucag.............................................................................  2    0   S03
..................auuuguccauagcaucag.............................................................................  2    0   S06
..................auuuguccauagcaucag.............................................................................  4    0   S01
..................auuuguccauagcaucag.............................................................................  5    0   S05
..................auuuguccauagcaucag.............................................................................  9    0   S04
..................auuuguccauagcaucagc............................................................................  1    0   S05
..................auuuguccauagcaucagc............................................................................  1    0   S03
..................auuuguccauagcaucagc............................................................................  1    0   S04
..................auuuguccauagcaucagcc...........................................................................  2    0   S01
..................auuuguccauagcaucagcc...........................................................................  3    0   S02
..................auuuguccauagcaucagcc...........................................................................  2    0   S05
..................auuuguccauagcaucagcc...........................................................................  1    0   S04
..................auuuguccauagcaucagcc...........................................................................  1    0   S06
..................auuuguccauagcaucagcca..........................................................................  12   0   S01
..................auuuguccauagcaucagcca..........................................................................  12   0   S04
..................auuuguccauagcaucagcca..........................................................................  3    0   S06
..................auuuguccauagcaucagcca..........................................................................  12   0   S02
..................auuuguccauagcaucagcca..........................................................................  12   0   S05
```

Mature **Star**

agccaauccucugguugaauuuguccauagcaucagccaauccucuguuuuuuuguacgauggguacuacgauggcuggugcuauggauaaauucaacccgaugguugacaaa			
..................auuuguccauagcaucagcca..	10	0	S03
...................uuuguccauagcaucagc..	1	0	S05
...................uuuguccauagcaucagc..	1	0	S01
...................uuuguccauagcaucagcc...	1	0	S03
...................uuuguccauagcaucagcc...	1	0	S02
...................uuuguccauagcaucagcc...	1	0	S04
...................uuuguccauagcaucagcca..	5	0	S05
...................uuuguccauagcaucagcca..	5	0	S01
...................uuuguccauagcaucagcca..	4	0	S03
...................uuuguccauagcaucagcca..	5	0	S02
...................uuuguccauagcaucagcca..	1	0	S04
...................uuuguccauagcaucagcca..	4	0	S06
....................uuguccauagcaucagcc...	4	0	S05
....................uuguccauagcaucagcc...	4	0	S01
....................uuguccauagcaucagcc...	1	0	S06
....................uuguccauagcaucagcc...	1	0	S04
....................uuguccauagcaucagcc...	2	0	S03
....................uuguccauagcaucagcca..	2	0	S02
....................uuguccauagcaucagcca..	2	0	S04
....................uuguccauagcaucagcca..	1	0	S06
....................uuguccauagcaucagccaau..	1	0	S03
....................uuguccauagcaucagccaau..	1	0	S02
....................uuguccauagcaucagccaauc...	2	0	S01
....................uuguccauagcaucagccaauc...	3	0	S02
....................uuguccauagcaucagccaauc...	2	0	S06
....................uuguccauagcaucagccaauc...	2	0	S04
....................uuguccauagcaucagccaauc...	1	0	S05
....................uuguccauagcaucagccaauc...	2	0	S03
....................uuguccauagcaucagccaaucc..	1	0	S04
....................uuguccauagcaucagccaauccucug..	1	0	S03
....................uuguccauagcaucagccaauccucuguu..	1	0	S03
.....................uguccauagcaucagcca..	1	0	S06
.....................uguccauagcaucagccaauccu...	1	0	S03
.....................uguccauagcaucagccaauccucuguuu...	1	0	S04
................................ucagccaauccucuguuu...	1	0	S02
....auggcuggugcuauggau........................	2	0	S01
....auggcuggugcuauggaua.......................	1	0	S03
....auggcuggugcuauggaua.......................	1	0	S05
....auggcuggugcuauggauaaauucaacc..............	1	0	S03
....uggcuggugcuauggaua.......................	1	0	S02
....uggcuggugcuauggaua.......................	1	0	S06
....uggcuggugcuauggauaaa.....................	4	0	S03
....uggcuggugcuauggauaaau....................	3	0	S06
....uggcuggugcuauggauaaau....................	2	0	S05
....uggcuggugcuauggauaaau....................	2	0	S03
....uggcuggugcuauggauaaau....................	2	0	S02
....uggcuggugcuauggauaaau....................	3	0	S04
....cuggugcuauggauaaauucaacc..............	1	0	S04
....uggugcuauggauaaauucaac...............	2	0	S03
....uggugcuauggauaaauucaac...............	2	0	S01
....uggugcuauggauaaauucaac...............	1	0	S02
....ggugcuauggauaaauucaac...............	1	0	S06
....ggugcuauggauaaauucaac...............	19	0	S03
....ggugcuauggauaaauucaac...............	32	0	S01
....ggugcuauggauaaauucaac...............	14	0	S02
....ggugcuauggauaaauucaac...............	6	0	S05
....ggugcuauggauaaauucaac...............	6	0	S04
....gugcuauggauaaauucaac...............	3	0	S02
....gugcuauggauaaauucaac...............	3	0	S01
....gugcuauggauaaauucaac...............	1	0	S06
....gugcuauggauaaauucaac...............	2	0	S04
....gugcuauggauaaauucaacc..............	35	0	S01
....gugcuauggauaaauucaacc..............	8	0	S06
....gugcuauggauaaauucaacc..............	10	0	S05
....gugcuauggauaaauucaacc..............	12	0	S03
....gugcuauggauaaauucaacc..............	21	0	S04
....gugcuauggauaaauucaacc..............	18	0	S02
....gugcuauggauaaauucaacccga...........	1	0	S04
....ugcuauggauaaauucaacc..............	3	0	S03
....ugcuauggauaaauucaacc..............	1	0	S02

Mature / Star			
agccaauccucugguugaauuuguccauagcaucagccaauccucuguuuuuuuguacgauggguacuacgauggcugguggcuauggauaaauucaacccgaugguugacaaa			
....ugcuauggauaaauucaaccc.............	1	0	S03
....ugcuauggauaaauucaacccg............	1	0	S04
....ugcuauggauaaauucaacccg............	3	0	S01
....ugcuauggauaaauucaacccg............	1	0	S03
....gcuauggauaaauucaacccg............	1	0	S01
....gcuauggauaaauucaacccg............	1	0	S04
....cuauggauaaauucaacc..............	1	0	S03
....cuauggauaaauucaacc..............	1	0	S01
....cuauggauaaauucaaccc.............	1	0	S06
....cuauggauaaauucaaccc.............	2	0	S04
....cuauggauaaauucaacccg............	11	0	S01
....cuauggauaaauucaacccg............	13	0	S06
....cuauggauaaauucaacccg............	6	0	S02
....cuauggauaaauucaacccg............	5	0	S03
....cuauggauaaauucaacccg............	6	0	S05
....cuauggauaaauucaacccg............	9	0	S04
....cuauggauaaauucaacccga...........	36	0	S05
....cuauggauaaauucaacccga...........	85	0	S04
....cuauggauaaauucaacccga...........	17	0	S02
....cuauggauaaauucaacccga...........	48	0	S01
....cuauggauaaauucaacccga...........	17	0	S06
....cuauggauaaauucaacccga...........	10	0	S03
....uauggauaaauucaacccga...........	1	0	S06
....uauggauaaauucaacccga...........	1	0	S04
....auggauaaauucaacccgaug.........	1	0	S05
....uggauaaauucaacccga...........	1	0	S04
....uggauaaauucaacccgaugg........	1	0	S04

Provisional ID : ta_iwgsc_6ds_v1_2075519_6387891

ata-miR168-5p

Score total	:	3954.8
Score for star read(s)	:	3.9
Score for read counts	:	3946.7
Score for mfe	:	2.7
Score for randfold	:	1.6
Score for cons. seed	:	
Total read count	:	7753
Mature read count	:	4441
Loop read count	:	0
Star read count	:	3295

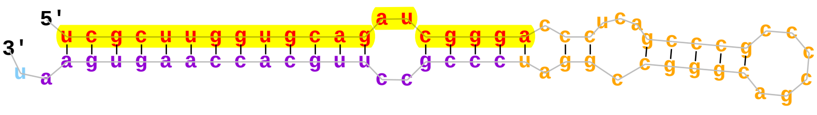

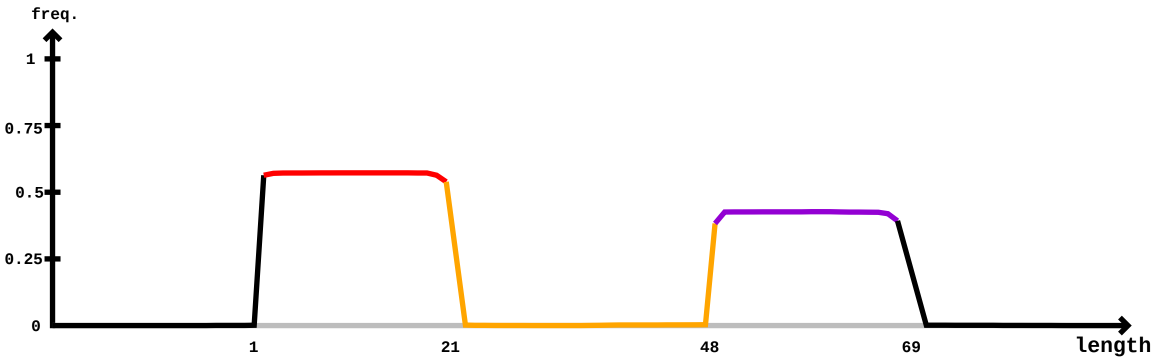


```
                    Mature                                      Star
5'- ccgucgccgccgccucgggcucgcuuggugcagaucgggacccucagcccgccccgacgggccggaucccgccuugcaccaagugaaucggagccggcgcagcgaccacg  -3'  obs
    ccgucgccgccgccucgggcucgcuuggugcagaucgggacccucagcccgccccgacgggccggaucccgccuugcaccaagugaaucggagccggcgcagcgaccacg       exp
    ..((((((((((.(((.((.(((((((((((((..(((((.((...(((((......))))).)).)))))..))))))))))))).)).))).)))))..))))).... reads   mm   sample
    ...............cgggcucgcuuggugcagauc.......................................................................... 1       0    S02
    ...............cgggcucgcuuggugcagauc.......................................................................... 1       0    S03
    ...............cgggcucgcuuggugcagauc.......................................................................... 2       0    S05
    ...................cucgcuuggugcagaucggga...................................................................... 2       0    S04
    ...................cucgcuuggugcagaucggga...................................................................... 1       0    S05
    ...................cucgcuuggugcagaucggga...................................................................... 1       0    S02
    ...................cucgcuuggugcagaucgggac..................................................................... 1       0    S02
    ....................ucgcuuggugcagaucgg........................................................................ 18      0    S06
    ....................ucgcuuggugcagaucgg........................................................................ 9       0    S05
    ....................ucgcuuggugcagaucgg........................................................................ 3       0    S04
    ....................ucgcuuggugcagaucgg........................................................................ 18      0    S03
    ....................ucgcuuggugcagaucgg........................................................................ 12      0    S02
    ....................ucgcuuggugcagaucgg........................................................................ 5       0    S01
    ....................ucgcuuggugcagaucggg....................................................................... 24      0    S03
    ....................ucgcuuggugcagaucggg....................................................................... 48      0    S02
    ....................ucgcuuggugcagaucggg....................................................................... 23      0    S01
    ....................ucgcuuggugcagaucggg....................................................................... 27      0    S05
    ....................ucgcuuggugcagaucggg....................................................................... 13      0    S04
    ....................ucgcuuggugcagaucggg....................................................................... 51      0    S06
    ....................ucgcuuggugcagaucggga...................................................................... 679     0    S05
    ....................ucgcuuggugcagaucggga...................................................................... 515     0    S04
    ....................ucgcuuggugcagaucggga...................................................................... 408     0    S01
    ....................ucgcuuggugcagaucggga...................................................................... 568     0    S03
    ....................ucgcuuggugcagaucggga...................................................................... 677     0    S06
    ....................ucgcuuggugcagaucggga...................................................................... 377     0    S02
    ....................ucgcuuggugcagaucgggac..................................................................... 135     0    S06
    ....................ucgcuuggugcagaucgggac..................................................................... 262     0    S05
    ....................ucgcuuggugcagaucgggac..................................................................... 144     0    S01
    ....................ucgcuuggugcagaucgggac..................................................................... 112     0    S04
    ....................ucgcuuggugcagaucgggac..................................................................... 124     0    S02
    ....................ucgcuuggugcagaucgggac..................................................................... 119     0    S03
    ....................ucgcuuggugcagaucgggacc.................................................................... 2       0    S02
    ....................ucgcuuggugcagaucgggacc.................................................................... 1       0    S01
```

Mature **Star**

ccgucgccgccgccucgggcucgcuuggugcagaucgggacccucagcccgccccgacgggccggaucccgccuugcaccaagugaaucggagccggcgcagcgaccacg			
....................ucgcuuggugcagaucgggacc..	4	0	S06
....................ucgcuuggugcagaucgggacc..	1	0	S04
....................ucgcuuggugcagaucgggacc..	1	0	S05
....................ucgcuuggugcagaucgggacccu..	1	0	S01
.....................cgcuuggugcagaucggg...	3	0	S02
.....................cgcuuggugcagaucggg...	2	0	S05
.....................cgcuuggugcagaucggg...	1	0	S06
.....................cgcuuggugcagaucggga..	10	0	S05
.....................cgcuuggugcagaucggga..	12	0	S03
.....................cgcuuggugcagaucggga..	11	0	S01
.....................cgcuuggugcagaucggga..	3	0	S04
.....................cgcuuggugcagaucggga..	8	0	S06
.....................cgcuuggugcagaucggga..	6	0	S02
......................gcuuggugcagaucggga..	1	0	S06
......................gcuuggugcagaucggga..	5	0	S03
......................gcuuggugcagaucggga..	1	0	S04
......................gcuuggugcagaucggga..	1	0	S02
......................gcuuggugcagaucgggac...	1	0	S01
.......................cuuggugcagaucgggacccu..	1	0	S02
.........................uggugcagaucgggacccuc...	1	0	S06
.........................uggugcagaucgggacccucagcccgcc...	1	0	S01
..........................ggugcagaucgggacccu..	1	0	S05
............................ugcagaucgggacccucagc..	1	0	S04
....ccgacgggccggaucccgccuugcacca.............................	1	0	S02
....cgacgggccggaucccgccuugcacc..............................	1	0	S02
....cgacgggccggaucccgccuugcacc..............................	1	0	S01
....cgacgggccggaucccgccuugcaccaagu..........................	2	0	S02
....gacgggccggaucccgccuugcacc..............................	1	0	S03
....gacgggccggaucccgccuugcacca.............................	1	0	S02
....gacgggccggaucccgccuugcaccaag...........................	1	0	S01
....acgggccggaucccgccuugcacc..............................	1	0	S06
....acgggccggaucccgccuugcacc..............................	1	0	S02
....acgggccggaucccgccuugcacc..............................	1	0	S01
....acgggccggaucccgccuugcacca.............................	1	0	S02
....acgggccggaucccgccuugcacca.............................	1	0	S03
....cgggccggaucccgccuugcac...............................	1	0	S06
....cgggccggaucccgccuugcacc..............................	1	0	S05
....cgggccggaucccgccuugcacca.............................	1	0	S02
....cgggccggaucccgccuugcacca.............................	1	0	S01
....cggaucccgccuugcaccaag...........................	2	0	S01
....cggaucccgccuugcaccaag...........................	1	0	S06
....gaucccgccuugcaccaagugaau......................	1	0	S06
....aucccgccuugcaccaagugaau......................	1	0	S03
....ucccgccuugcaccaagug.........................	1	0	S05
....ucccgccuugcaccaagugaa.......................	1	0	S05
....ucccgccuugcaccaagugaau......................	1	0	S05
....cccgccuugcaccaagug.........................	2	0	S06
....cccgccuugcaccaagug.........................	14	0	S03
....cccgccuugcaccaagug.........................	10	0	S01
....cccgccuugcaccaagug.........................	2	0	S04
....cccgccuugcaccaagug.........................	7	0	S05
....cccgccuugcaccaagug.........................	6	0	S02
....cccgccuugcaccaaguga........................	30	0	S01
....cccgccuugcaccaaguga........................	18	0	S02
....cccgccuugcaccaaguga........................	23	0	S04
....cccgccuugcaccaaguga........................	71	0	S03
....cccgccuugcaccaaguga........................	26	0	S06
....cccgccuugcaccaaguga........................	23	0	S05
....cccgccuugcaccaagugaa.......................	201	0	S05
....cccgccuugcaccaagugaa.......................	223	0	S06
....cccgccuugcaccaagugaa.......................	128	0	S02
....cccgccuugcaccaagugaa.......................	439	0	S03
....cccgccuugcaccaagugaa.......................	171	0	S01
....cccgccuugcaccaagugaa.......................	249	0	S04
....cccgccuugcaccaagugaau......................	346	0	S03
....cccgccuugcaccaagugaau......................	235	0	S05
....cccgccuugcaccaagugaau......................	168	0	S04
....cccgccuugcaccaagugaau......................	125	0	S06
....cccgccuugcaccaagugaau......................	256	0	S01
....cccgccuugcaccaagugaau......................	182	0	S02

```
                    Mature                                      Star
ccgucgccgccgccucgggcucgcuuggugcagaucgggacccucagcccgccccgacgggccggaucccgccuugcaccaagugaaucggagccggcgcagcgaccacg
...................................................................cccgccuugcaccaagugaauc.....................   1   0   S05
...................................................................cccgccuugcaccaagugaauc.....................   1   0   S02
....................................................................ccgccuugcaccaaguga........................   1   0   S01
....................................................................ccgccuugcaccaaguga........................   2   0   S05
....................................................................ccgccuugcaccaaguga........................   2   0   S06
....................................................................ccgccuugcaccaaguga........................   8   0   S03
....................................................................ccgccuugcaccaaguga........................   3   0   S02
....................................................................ccgccuugcaccaaguga........................   1   0   S04
....................................................................ccgccuugcaccaagugaa.......................  29   0   S06
....................................................................ccgccuugcaccaagugaa.......................  44   0   S03
....................................................................ccgccuugcaccaagugaa.......................  26   0   S05
....................................................................ccgccuugcaccaagugaa.......................  16   0   S01
....................................................................ccgccuugcaccaagugaa.......................  14   0   S02
....................................................................ccgccuugcaccaagugaa.......................  41   0   S04
....................................................................ccgccuugcaccaagugaau......................  16   0   S02
....................................................................ccgccuugcaccaagugaau......................  23   0   S04
....................................................................ccgccuugcaccaagugaau......................  27   0   S03
....................................................................ccgccuugcaccaagugaau......................  29   0   S01
....................................................................ccgccuugcaccaagugaau......................  16   0   S06
....................................................................ccgccuugcaccaagugaau......................  36   0   S05
.....................................................................cgccuugcaccaagugaa.......................   1   0   S03
.....................................................................cgccuugcaccaagugaa.......................   1   0   S06
.....................................................................cgccuugcaccaagugaau......................   1   0   S01
.......................................................................ccuugcaccaagugaaucgga..................   1   0   S01
........................................................................cuugcaccaagugaaucgga..................   1   0   S05
............................................................................accaagugaaucggagccgg..............   1   0   S05
............................................................................accaagugaaucggagccgg..............   2   0   S04
............................................................................accaagugaaucggagccgg..............   1   0   S06
............................................................................accaagugaaucggagccggcgcagc........   1   0   S04
...............................................................................aagugaaucggagccggcgc...........   1   0   S06
................................................................................agugaaucggagccggcgcagcg.......   1   0   S05
..................................................................................ugaaucggagccggcgcagc........   1   0   S03
```

Provisional ID	:	ta_iwgsc_1ds_v1_1896382_837967
Score total	:	82752.6
Score for star read(s)	:	3.9
Score for read counts	:	82744.7
Score for mfe	:	2.3
Score for randfold	:	1.6
Score for cons. seed	:	
Total read count	:	162312
Mature read count	:	241621
Loop read count	:	11
Star read count	:	1282

ata-miR9863-3p (-5p)

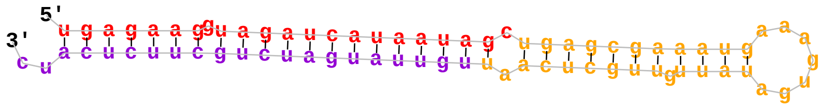

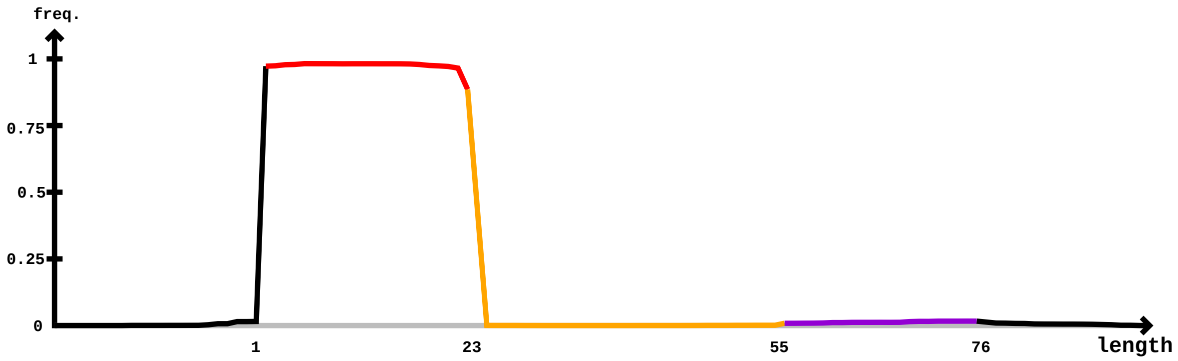

Mature   Star

```
5'- ccgcuaaacaagucuuuagaugagaagguagaucauaauagcugagcgaaaugaaagugauauuguugcucaauuguuaugaucugcuucucaucugaagacuaguuuauua  -3'   obs
    ccgcuaaacaagucuuuagaugagaagguagaucauaauagcugagcgaaaugaaagugauauuguugcucaauuguuaugaucugcuucucaucugaagacuaguuuauua        exp
    ....(((((.(((((((((((((((((.(((((((((((((.(((((((((((.......)))).))))))).)))))))))))))))))))))))))))))).)))))...  reads  mm  sample
    ....uaaacaagucuuuagaugaga.......................................................................................  1  0  S04
    ....uaaacaagucuuuagaugaga.......................................................................................  2  0  S05
    ......aacaagucuuuagaugaga.......................................................................................  2  0  S03
    ......aacaagucuuuagaugaga.......................................................................................  1  0  S01
    ......aacaagucuuuagaugaga.......................................................................................  4  0  S05
    ......aacaagucuuuagaugaga.......................................................................................  2  0  S04
    ......aacaagucuuuagaugagaa......................................................................................  3  0  S05
    ......aacaagucuuuagaugagaa......................................................................................  2  0  S02
    ......aacaagucuuuagaugagaa......................................................................................  2  0  S04
    ......aacaagucuuuagaugagaa......................................................................................  11  0  S06
    ......aacaagucuuuagaugagaa......................................................................................  1  0  S03
    ......aacaagucuuuagaugagaag.....................................................................................  4  0  S03
    ......aacaagucuuuagaugagaag.....................................................................................  5  0  S06
    ......aacaagucuuuagaugagaag.....................................................................................  3  0  S01
    ......aacaagucuuuagaugagaag.....................................................................................  3  0  S02
    ......aacaagucuuuagaugagaagg....................................................................................  12  0  S05
    ......aacaagucuuuagaugagaagg....................................................................................  3  0  S04
    ......aacaagucuuuagaugagaagg....................................................................................  6  0  S02
    ......aacaagucuuuagaugagaagg....................................................................................  8  0  S01
    ......aacaagucuuuagaugagaagg....................................................................................  7  0  S03
    ......aacaagucuuuagaugagaagg....................................................................................  10  0  S06
    .......acaagucuuuagaugagaag.....................................................................................  1  0  S02
    .......acaagucuuuagaugagaagg....................................................................................  2  0  S06
    .........aagucuuuagaugagaagg....................................................................................  2  0  S06
    .........aagucuuuagaugagaagg....................................................................................  1  0  S04
    ..........agucuuuagaugagaagg....................................................................................  1  0  S04
    ..........agucuuuagaugagaaggua..................................................................................  1  0  S01
    ..........agucuuuagaugagaagguag.................................................................................  1  0  S05
    ..........agucuuuagaugagaagguag.................................................................................  1  0  S04
    ..........agucuuuagaugagaagguag.................................................................................  1  0  S02
    ..........agucuuuagaugagaagguaga................................................................................  1  0  S01
    ..........agucuuuagaugagaagguaga................................................................................  1  0  S02
    ..........agucuuuagaugagaagguaga................................................................................  1  0  S03
```

```
                     Mature                                                       Star
ccgcuaaacaagucuuuagaugagaagguagaucauaauagcugagcgaaaugaaagugauauuguugcucaauuguuaugaucugcuucucaucugaagacuaguuuauua
..........agucuuuagaugagaagguaga................................................................................   2     0     S04
............ucuuuagaugagaagguagauc..............................................................................   1     0     S03
............ucuuuagaugagaagguagauc..............................................................................   1     0     S02
............ucuuuagaugagaagguagauc..............................................................................   4     0     S01
............ucuuuagaugagaagguagauc..............................................................................   2     0     S06
............ucuuuagaugagaagguagauc..............................................................................   2     0     S04
............ucuuuagaugagaagguagauc..............................................................................   6     0     S05
............ucuuuagaugagaagguagauca.............................................................................   1     0     S04
.............cuuuagaugagaagguaga................................................................................   1     0     S01
.............cuuuagaugagaagguaga................................................................................   1     0     S04
.............cuuuagaugagaagguagauca.............................................................................   3     0     S04
.............cuuuagaugagaagguagauca.............................................................................   6     0     S05
.............cuuuagaugagaagguagauca.............................................................................   3     0     S01
.............cuuuagaugagaagguagauca.............................................................................   1     0     S03
.............cuuuagaugagaagguagauca.............................................................................   1     0     S06
.............cuuuagaugagaagguagauca.............................................................................   2     0     S02
.............cuuuagaugagaagguagaucau............................................................................   1     0     S03
..............uuuagaugagaagguaga................................................................................   1     0     S02
..............uuuagaugagaagguaga................................................................................   1     0     S01
..............uuuagaugagaagguaga................................................................................   1     0     S04
..............uuuagaugagaagguaga................................................................................   1     0     S05
..............uuuagaugagaagguagau...............................................................................   1     0     S01
..............uuuagaugagaagguagauc..............................................................................   2     0     S04
..............uuuagaugagaagguagauc..............................................................................   1     0     S05
..............uuuagaugagaagguagauca.............................................................................   14    0     S03
..............uuuagaugagaagguagauca.............................................................................   12    0     S04
..............uuuagaugagaagguagauca.............................................................................   23    0     S05
..............uuuagaugagaagguagauca.............................................................................   4     0     S01
..............uuuagaugagaagguagauca.............................................................................   10    0     S02
..............uuuagaugagaagguagauca.............................................................................   6     0     S06
..............uuuagaugagaagguagaucau............................................................................   44    0     S06
..............uuuagaugagaagguagaucau............................................................................   10    0     S03
..............uuuagaugagaagguagaucau............................................................................   82    0     S05
..............uuuagaugagaagguagaucau............................................................................   50    0     S04
..............uuuagaugagaagguagaucau............................................................................   36    0     S01
..............uuuagaugagaagguagaucau............................................................................   58    0     S02
..............uuuagaugagaagguagaucaua...........................................................................   1     0     S03
..............uuuagaugagaagguagaucaua...........................................................................   1     0     S06
..............uuuagaugagaagguagaucaua...........................................................................   2     0     S05
..............uuuagaugagaagguagaucaua...........................................................................   1     0     S04
...............uuagaugagaagguagau...............................................................................   1     0     S01
...............uuagaugagaagguagauc..............................................................................   1     0     S04
...............uuagaugagaagguagauc..............................................................................   3     0     S05
...............uuagaugagaagguagauca.............................................................................   2     0     S02
...............uuagaugagaagguagauca.............................................................................   2     0     S03
...............uuagaugagaagguagauca.............................................................................   1     0     S01
...............uuagaugagaagguagaucau............................................................................   3     0     S03
...............uuagaugagaagguagaucau............................................................................   10    0     S05
...............uuagaugagaagguagaucau............................................................................   8     0     S04
...............uuagaugagaagguagaucau............................................................................   10    0     S01
...............uuagaugagaagguagaucau............................................................................   7     0     S06
...............uuagaugagaagguagaucau............................................................................   3     0     S02
...............uuagaugagaagguagaucaua...........................................................................   140   0     S05
...............uuagaugagaagguagaucaua...........................................................................   87    0     S01
...............uuagaugagaagguagaucaua...........................................................................   137   0     S02
...............uuagaugagaagguagaucaua...........................................................................   97    0     S04
...............uuagaugagaagguagaucaua...........................................................................   37    0     S03
...............uuagaugagaagguagaucaua...........................................................................   87    0     S06
...............uuagaugagaagguagaucauaa..........................................................................   1     0     S04
...............uuagaugagaagguagaucauaa..........................................................................   1     0     S03
...............uuagaugagaagguagaucauaa..........................................................................   1     0     S05
................uagaugagaagguagaucaua...........................................................................   1     0     S01
................uagaugagaagguagaucaua...........................................................................   3     0     S04
................uagaugagaagguagaucauaa..........................................................................   1     0     S04
................uagaugagaagguagaucauaa..........................................................................   1     0     S05
.................agaugagaagguagauca.............................................................................   1     0     S02
.................agaugagaagguagauca.............................................................................   1     0     S01
.................agaugagaagguagauca.............................................................................   1     0     S04
.................agaugagaagguagaucau............................................................................   1     0     S04
.................agaugagaagguagaucau............................................................................   1     0     S05
```

```
                    Mature                                                Star
ccgcuaaacaagucuuuagaugagaagguagaucauaauagcugagcgaaaugaaagugauauuguugcucaauuguuaugaucugcuucucaucugaagacuaguuuauua
.................agaugagaagguagaucaua...........................................................................   1      0   S01
.................agaugagaagguagaucaua...........................................................................   4      0   S03
.................agaugagaagguagaucaua...........................................................................   1      0   S06
.................agaugagaagguagaucaua...........................................................................   2      0   S02
.................agaugagaagguagaucaua...........................................................................   1      0   S05
.................agaugagaagguagaucauaa..........................................................................   1      0   S05
.................agaugagaagguagaucauaa..........................................................................   1      0   S06
.................agaugagaagguagaucauaa..........................................................................   3      0   S04
.................agaugagaagguagaucauaau.........................................................................   3      0   S06
.................agaugagaagguagaucauaau.........................................................................   1      0   S02
.................agaugagaagguagaucauaaua........................................................................   1      0   S05
.................agaugagaagguagaucauaaua........................................................................   2      0   S01
.................agaugagaagguagaucauaaua........................................................................   1      0   S03
.................agaugagaagguagaucauaaua........................................................................   4      0   S02
.................agaugagaagguagaucauaauag.......................................................................   4      0   S05
.................agaugagaagguagaucauaauag.......................................................................   25     0   S03
.................agaugagaagguagaucauaauag.......................................................................   36     0   S06
.................agaugagaagguagaucauaauag.......................................................................   20     0   S01
.................agaugagaagguagaucauaauag.......................................................................   13     0   S04
.................agaugagaagguagaucauaauag.......................................................................   52     0   S02
.................agaugaCaagguagaucauaauagc......................................................................   1      1   S03
.................agaugagaagguagaucauaauagc......................................................................   211    0   S05
.................agaugagaagguagaucauaauagc......................................................................   173    0   S04
.................agaugagaagguagaucauaauagc......................................................................   152    0   S01
.................agaugagaagguagaucauaauagc......................................................................   139    0   S03
.................agaugagaagguagaucauaauagc......................................................................   143    0   S06
.................agaugagaagguagaucauaauagc......................................................................   312    0   S02
.................agaugagaagguagaucauaauagcu.....................................................................   1      0   S02
.................agaugagaagguagaucauaauagcu.....................................................................   2      0   S05
.................agaugagaagguagaucauaauagcu.....................................................................   2      0   S06
.................agaugagaagguagaucauaauagcuga...................................................................   1      0   S02
..................gaugagaagguagaucau............................................................................   1      0   S06
..................gaugagaagguagaucauaauagc......................................................................   1      0   S04
..................gaugagaagguagaucauaauagcu.....................................................................   1      0   S06
...................augagaagguagaucaua...........................................................................   1      0   S02
...................augagaagguagaucauaa..........................................................................   2      0   S03
...................augagaagguagaucauaau.........................................................................   1      0   S06
...................augagaagguagaucauaauag.......................................................................   2      0   S05
...................augagaagguagaucauaauag.......................................................................   1      0   S04
...................augagaagguagaucauaauag.......................................................................   8      0   S03
...................augagaagguagaucauaauag.......................................................................   4      0   S06
...................augagaagguagaucauaauag.......................................................................   2      0   S02
...................augagaagguagaucauaauagc......................................................................   6      0   S06
...................augagaagguagaucauaauagc......................................................................   5      0   S04
...................augagaagguagaucauaauagc......................................................................   10     0   S01
...................augagaagguagaucauaauagc......................................................................   9      0   S03
...................augagaagguagaucauaauagc......................................................................   17     0   S02
...................augagaagguagaucauaauagc......................................................................   14     0   S05
....................ugagaagguagaucauaa..........................................................................   59     0   S02
....................ugagaagguagaucauaa..........................................................................   24     0   S01
....................ugagaagguagaucauaa..........................................................................   21     0   S05
....................ugagaagguagaucauaa..........................................................................   49     0   S06
....................ugagaagguagaucauaa..........................................................................   23     0   S04
....................ugagaagguagaucauaa..........................................................................   54     0   S03
....................ugagaagguagaucauaau.........................................................................   56     0   S06
....................ugagaagguagaucauaau.........................................................................   45     0   S04
....................ugagaagguagaucauaau.........................................................................   77     0   S03
....................ugagaagguagaucauaau.........................................................................   61     0   S01
....................ugagaagguagaucauaau.........................................................................   93     0   S02
....................ugagaagguagaucauaau.........................................................................   42     0   S05
....................ugagaagguagaucauaaua........................................................................   244    0   S03
....................ugagaagguagaucauaaua........................................................................   100    0   S05
....................ugagaagguagaucauaaua........................................................................   47     0   S04
....................ugagaagguagaucauaaua........................................................................   284    0   S06
....................ugagaagguagaucauaaua........................................................................   121    0   S01
....................ugagaagguagaucauaaua........................................................................   214    0   S02
....................ugaCaagguagaucauaauag.......................................................................   2      1   S05
....................ugagaagguagaucauaauag.......................................................................   3175   0   S02
....................ugaCaagguagaucauaauag.......................................................................   1      1   S04
....................ugaCaagguagaucauaauag.......................................................................   1      1   S01
```

Mature **Star**

```
ccgcuaaacaagucuuuagaugagaagguagaucauaauagcugagcgaaaugaaagugauauuguugcucaauuguuaugaucugcuucucaucugaagacuaguuuauua
....................ugagaagguagaucauaauag.......................................................................    774    0  S04
....................ugagaagguagaucauaauag.......................................................................    1403   0  S01
....................ugaCaagguagaucauaauag.......................................................................    3      1  S06
....................ugagaagguagaucauaauag.......................................................................    3958   0  S03
....................ugagaagguagaucauaauag.......................................................................    854    0  S05
....................ugagaagguagaucauaauag.......................................................................    2808   0  S06
....................ugagaagguagaucauaauagc......................................................................    20137  0  S04
....................ugagaagguagaucauaauagc......................................................................    18821  0  S01
....................ugaCaagguagaucauaauagc......................................................................    11     1  S02
....................ugaCaagguagaucauaauagc......................................................................    8      1  S01
....................ugaCaagguagaucauaauagc......................................................................    11     1  S04
....................ugaCaagguagaucauaauagc......................................................................    6      1  S06
....................ugagaagguagaucauaauagc......................................................................    30020  0  S02
....................ugagaagguagaucauaauagc......................................................................    33193  0  S05
....................ugaCaagguagaucauaauagc......................................................................    5      1  S03
....................ugagaagguagaucauaauagc......................................................................    19988  0  S06
....................ugagaagguagaucauaauagc......................................................................    21677  0  S03
....................ugaCaagguagaucauaauagc......................................................................    12     1  S05
....................ugagaagguagaucauaauagcu.....................................................................    71     0  S04
....................ugagaagguagaucauaauagcu.....................................................................    51     0  S06
....................ugagaagguagaucauaauagcu.....................................................................    90     0  S02
....................ugagaagguagaucauaauagcu.....................................................................    101    0  S05
....................ugagaagguagaucauaauagcu.....................................................................    61     0  S01
....................ugagaagguagaucauaauagcu.....................................................................    40     0  S03
....................ugagaagguagaucauaauagcug....................................................................    2      0  S03
....................ugagaagguagaucauaauagcug....................................................................    1      0  S05
....................ugagaagguagaucauaauagcug....................................................................    1      0  S06
....................ugagaagguagaucauaauagcug....................................................................    1      0  S04
....................ugagaagguagaucauaauagcuga...................................................................    1      0  S01
....................ugagaagguagaucauaauagcuga...................................................................    5      0  S06
....................ugagaagguagaucauaauagcuga...................................................................    1      0  S02
....................ugagaagguagaucauaauagcugag..................................................................    2      0  S02
....................ugagaagguagaucauaauagcugag..................................................................    1      0  S05
....................ugagaagguagaucauaauagcugag..................................................................    1      0  S01
....................ugagaagguagaucauaauagcugagc.................................................................    4      0  S05
....................ugagaagguagaucauaauagcugagc.................................................................    2      0  S02
....................ugagaagguagaucauaauagcugagc.................................................................    1      0  S01
....................ugagaagguagaucauaauagcugagc.................................................................    1      0  S04
....................ugagaagguagaucauaauagcugagc.................................................................    3      0  S03
....................ugagaagguagaucauaauagcugagcg................................................................    1      0  S06
....................ugagaagguagaucauaauagcugagcg................................................................    1      0  S01
....................ugagaagguagaucauaauagcugagcga...............................................................    1      0  S04
....................ugagaagguagaucauaauagcugagcga...............................................................    2      0  S05
....................ugagaagguagaucauaauagcugagcgaa..............................................................    1      0  S06
.....................gagaagguagaucauaau.........................................................................    1      0  S01
.....................gagaagguagaucauaau.........................................................................    1      0  S03
.....................gagaagguagaucauaaua........................................................................    1      0  S03
.....................gagaagguagaucauaaua........................................................................    3      0  S06
.....................gagaagguagaucauaaua........................................................................    1      0  S02
.....................gagaagguagaucauaaua........................................................................    1      0  S01
.....................gagaagguagaucauaauag.......................................................................    3      0  S02
.....................gagaagguagaucauaauag.......................................................................    3      0  S03
.....................gagaagguagaucauaauag.......................................................................    3      0  S04
.....................gagaagguagaucauaauag.......................................................................    2      0  S01
.....................gagaagguagaucauaauag.......................................................................    2      0  S05
.....................gagaagguagaucauaauag.......................................................................    6      0  S06
.....................gagaagguagaucauaauagc......................................................................    49     0  S02
.....................gagaagguagaucauaauagc......................................................................    26     0  S03
.....................gagaagguagaucauaauagc......................................................................    17     0  S01
.....................gagaagguagaucauaauagc......................................................................    18     0  S06
.....................gagaagguagaucauaauagc......................................................................    35     0  S05
.....................gagaagguagaucauaauagc......................................................................    24     0  S04
.....................gagaagguagaucauaauagcu.....................................................................    7      0  S01
.....................gagaagguagaucauaauagcu.....................................................................    2      0  S04
.....................gagaagguagaucauaauagcu.....................................................................    5      0  S06
.....................gagaagguagaucauaauagcu.....................................................................    6      0  S02
.....................gagaagguagaucauaauagcu.....................................................................    4      0  S03
.....................gagaagguagaucauaauagcu.....................................................................    7      0  S05
......................agaagguagaucauaaua........................................................................    1      0  S03
......................agaagguagaucauaaua........................................................................    1      0  S02
```

Mature **Star**

```
ccgcuaaacaagucuuuagaugagaagguagaucauaauagcugagcgaaaugaaagugauauuguugcucaauuguuaugaucugcuucucaucugaagacuaguuuauua
......................agaagguagaucauaaua........................................................................  1    0  S06
......................agaagguagaucauaaua........................................................................  1    0  S05
......................agaagguagaucauaauag.......................................................................  27   0  S06
......................agaagguagaucauaauag.......................................................................  1    0  S05
......................agaagguagaucauaauag.......................................................................  37   0  S03
......................agaagguagaucauaauag.......................................................................  1    0  S04
......................agaagguagaucauaauag.......................................................................  3    0  S01
......................agaagguagaucauaauag.......................................................................  12   0  S02
......................agaagguagaucauaauagc......................................................................  28   0  S04
......................agaagguagaucauaauagc......................................................................  30   0  S01
......................agaagguagaucauaauagc......................................................................  137  0  S02
......................agaagguagaucauaauagc......................................................................  207  0  S03
......................agaagguagaucauaauagc......................................................................  139  0  S06
......................agaagguagaucauaauagc......................................................................  39   0  S05
......................agaagguagaucauaauagcu.....................................................................  1    0  S05
......................agaagguagaucauaauagcug....................................................................  1    0  S02
.......................gaagguagaucauaauag.......................................................................  4    0  S01
.......................gaagguagaucauaauag.......................................................................  1    0  S05
.......................gaagguagaucauaauag.......................................................................  4    0  S06
.......................gaagguagaucauaauag.......................................................................  1    0  S03
.......................gaagguagaucauaauagc......................................................................  11   0  S04
.......................Caagguagaucauaauagc......................................................................  1    1  S01
.......................gaagguagaucauaauagc......................................................................  23   0  S06
.......................gaagguagaucauaauagc......................................................................  21   0  S05
.......................gaagguagaucauaauagc......................................................................  40   0  S03
.......................gaagguagaucauaauagc......................................................................  20   0  S02
.......................gaagguagaucauaauagc......................................................................  10   0  S01
.......................gaagguagaucauaauagcuga...................................................................  1    0  S06
.......................gaagguagaucauaauagcugagc.................................................................  1    0  S02
........................aagguagaucauaauagc......................................................................  32   0  S04
........................aagguagaucauaauagc......................................................................  75   0  S02
........................aagguagaucauaauagc......................................................................  55   0  S05
........................aagguaAaucauaauagc......................................................................  1    1  S03
........................aagguagaucauaauagc......................................................................  156  0  S06
........................aagguagaucauaauagc......................................................................  140  0  S03
........................aagguagaucauaauagc......................................................................  54   0  S01
.........................agguagaucauaauagcu.....................................................................  1    0  S04
.........................agguagaucauaauagcugagc.................................................................  1    0  S06
.........................agguagaucauaauagcugagc.................................................................  2    0  S04
.........................agguagaucauaauagcugagcga...............................................................  1    0  S01
.........................agguagaucauaauagcugagcgaaaug...........................................................  1    0  S03
.........................agguagaucauaauagcugagcgaaaug...........................................................  1    0  S02
.........................agguagaucauaauagcugagcgaaaug...........................................................  1    0  S06
.........................agguagaucauaauagcugagcgaaaugaa.........................................................  1    0  S03
.............................agaucauaauagcugagc.................................................................  3    0  S02
.............................agaucauaauagcugagc.................................................................  3    0  S03
.............................agaucauaauagcugagc.................................................................  3    0  S06
.............................agaucauaauagcugagc.................................................................  3    0  S05
.............................agaucauaauagcugagcg................................................................  1    0  S02
.............................agaucauaauagcugagcg................................................................  3    0  S06
.............................agaucauaauagcugagcga...............................................................  1    0  S03
.............................agaucauaauagcugagcgaa..............................................................  2    0  S04
.............................agaucauaauagcugagcgaaaug...........................................................  1    0  S06
.............................agaucauaauagcugagcgaaaug...........................................................  1    0  S05
.............................agaucauaauagcugagcgaaaug...........................................................  1    0  S03
.............................agaucauaauagcugagcgaaauga..........................................................  1    0  S03
.............................agaucauaauagcugagcgaaaugaaagug.....................................................  1    0  S03
..............................gaucauaauagcugagcg................................................................  1    0  S06
..............................gaucauaauagcugagcgaaaug...........................................................  1    0  S03
..............................gaucauaauagcugagcgaaaug...........................................................  2    0  S06
..................................auaauagcugagcgaaaugaa.........................................................  1    0  S01
..................................auaauagcugagcgaaaugaa.........................................................  1    0  S05
..................................auaauagcugagcgaaaugaa.........................................................  1    0  S03
..........................................ugagcgaaaugaaaguga....................................................  1    0  S03
..........................................ugagcgaaaugaaagugauauuguugc...........................................  1    0  S05
............................................agcgaaaugaaagugauauuguugcuca........................................  1    0  S02
...............................................gaaaugaaagugauauuguugc...........................................  1    0  S01
.................................................aaugaaagugauauuguugcu..........................................  1    0  S05
..................................................augaaagugauauuguugcuc.........................................  1    0  S05
...................................................ugaaagugauauuguugcuc.........................................  1    0  S05
```

```
                     Mature                                              Star
ccgcuaaacaagucuuuagaugagaagguagaucauaauagcugagcgaaaugaaagugauauuguugcucaauuguuaugaucugcuucucaucugaagacuaguuuauua
...................................................ugaaagugauauuguugcuca........................................  1  0  S06
...................................................ugaaagugauauuguugcuca........................................  1  0  S01
.....................................................aaagugauauuguugcucaauuguuaug...............................  1  0  S06
......................................................aagugauauuguugcucaau......................................  1  0  S04
.......................................................agugauauuguugcucaau......................................  1  0  S02
........................................................gugauauuguugcucaauuguuaugauc............................  1  0  S04
........................................................gugauauuguugcucaauuguuaugauc............................  2  0  S02
........................................................gugauauuguugcucaauuguuaugaucu...........................  1  0  S02
.........................................................ugauauuguugcucaauuguuaugauc............................  1  0  S06
.........................................................ugauauuguugcucaauuguuaugauc............................  1  0  S05
.........................................................ugauauuguugcucaauuguuaugauc............................  2  0  S03
.........................................................ugauauuguugcucaauuguuaugaucugc.........................  1  0  S03
.........................................................ugauauuguugcucaauuguuaugaucugc.........................  1  0  S06
...........................................................auauuguugcucaauuguuaugau.............................  1  0  S05
...........................................................auauuguugcucaauuguuaugauc............................  1  0  S04
...........................................................auauuguugcucaauuguuaugaucugc.........................  1  0  S03
............................................................uauuguugcucaauuguuaugauc............................  1  0  S05
............................................................uauuguugcucaauuguuaugaucug..........................  1  0  S01
.............................................................auuguugcucaauuguuaugauc............................  1  0  S05
.............................................................auuguugcucaauuguuaugauc............................  1  0  S06
.............................................................auuguugcucaauuguuaugaucu...........................  1  0  S03
...............................................................uguugcucaauuguuaugauc............................  1  0  S06
...............................................................uguugcucaauuguuaugauc............................  2  0  S04
...............................................................uguugcucaauuguuaugauc............................  2  0  S03
...............................................................uguugcucaauuguuaugauc............................  1  0  S05
...............................................................uguugcucaauuguuaugaucu...........................  1  0  S01
...............................................................uguugcucaauuguuaugaucugc.........................  1  0  S03
...............................................................uguugcucaauuguuaugaucugc.........................  1  0  S06
...............................................................uguugcucaauuguuaugaucugc.........................  1  0  S01
...............................................................uguugcucaauuguuaugaucugc.........................  1  0  S02
...............................................................uguugcucaauuguuaugaucugcu........................  1  0  S03
................................................................guugcucaauuguuaugaucu...........................  1  0  S05
................................................................guugcucaauuguuaugaucug..........................  1  0  S01
................................................................guugcucaauuguuaugaucugc.........................  1  0  S06
.................................................................uugcucaauuguuaugaucu...........................  1  0  S04
.................................................................uugcucaauuguuaugaucu...........................  2  0  S03
.................................................................uugcucaauuguuaugaucu...........................  1  0  S05
.................................................................uugcucaauuguuaugaucug..........................  1  0  S03
.................................................................uugcucaauuguuaugaucug..........................  1  0  S01
.................................................................uugcucaauuguuaugaucug..........................  2  0  S04
.................................................................uugcucaauuguuaugaucug..........................  1  0  S05
.................................................................uugcucaauuguuaugaucugc.........................  2  0  S05
.................................................................uugcucaauuguuaugaucugc.........................  2  0  S01
.................................................................uugcucaauuguuaugaucugc.........................  3  0  S02
.................................................................uugcucaauuguuaugaucugc.........................  2  0  S03
.................................................................uugcucaauuguuaugaucugcuucu.....................  1  0  S06
.................................................................uugcucaauuguuaugaucugcuucucauc.................  1  0  S02
.................................................................uugcucaauuguuaugaucugcuucucauc.................  1  0  S03
..................................................................ugcucaauuguuaugaucug..........................  4  0  S06
..................................................................ugcucaauuguuaugaucug..........................  2  0  S03
..................................................................ugcucaauuguuaugaucug..........................  1  0  S01
..................................................................ugcucaauuguuaugaucug..........................  2  0  S02
..................................................................ugcucaauuguuaugaucugc.........................  7  0  S06
..................................................................ugcucaauuguuaugaucugc.........................  2  0  S03
..................................................................ugcucaauuguuaugaucugc.........................  15 0  S05
..................................................................ugcucaauuguuaugaucugc.........................  5  0  S02
..................................................................ugcucaauuguuaugaucugc.........................  4  0  S01
..................................................................ugcucaauuguuaugaucugc.........................  3  0  S04
..................................................................ugcucaauuguuaugaucugcuucuc....................  1  0  S01
..................................................................ugcucaauuguuaugaucugcuucucau..................  1  0  S03
...................................................................gcucaauuguuaugaucugcu........................  1  0  S05
...................................................................gcucaauuguuaugaucugcu........................  1  0  S02
...................................................................gcucaauuguuaugaucugcu........................  1  0  S06
...................................................................gcucaauuguuaugaucugcu........................  1  0  S01
...................................................................gcucaauuguuaugaucugcuu.......................  1  0  S06
...................................................................gcucaauuguuaugaucugcuuc......................  2  0  S04
...................................................................gcucaauuguuaugaucugcuuc......................  1  0  S03
...................................................................gcucaauuguuaugaucugcuucuc....................  1  0  S03
...................................................................gcucaauuguuaugaucugcuucucauc.................  1  0  S02
....................................................................cucaauuguuaugaucugcuu.......................  1  0  S06
```

Mature **Star**

ccgcuaaacaagucuuuagaugagaagguagaucauaauagcugagcgaaaugaaagugauauuguugcucaauuguuaugaucugcuucucaucugaagacuaguuuauua			
....ucaauuguuaugaucugc.........................	1	0	S01
....ucaauuguuaugaucugc.........................	1	0	S05
....ucaauuguuaugaucugc.........................	1	0	S03
....ucaauuguuaugaucugcu........................	1	0	S01
....ucaauuguuaugaucugcuu.......................	2	0	S03
....ucaauuguuaugaucugcuu.......................	2	0	S01
....ucaauuguuaugaucugcuu.......................	1	0	S06
....ucaauuguuaugaucugcuu.......................	1	0	S04
....ucaauuguuaugaucugcuu.......................	1	0	S05
....ucaauuguuaugaucugcuuc......................	5	0	S03
....ucaauuguuaugaucugcuuc......................	8	0	S05
....ucaauuguuaugaucugcuuc......................	3	0	S01
....ucaauuguuaugaucugcuuc......................	2	0	S06
....ucaauuguuaugaucugcuuc......................	6	0	S04
....ucaauuguuaugaucugcuuc......................	2	0	S02
....ucaauuguuaugaucugcuucu.....................	1	0	S05
....ucaauuguuaugaucugcuucu.....................	4	0	S03
....ucaauuguuaugaucugcuucu.....................	1	0	S01
....ucaauuguuaugaucugcuucuca...................	1	0	S01
....ucaauuguuaugaucugcuucucauc.................	1	0	S05
....ucaauuguuaugaucugcuucucauc.................	1	0	S01
....caauuguuaugaucugcuucu.....................	1	0	S04
....caauuguuaugaucugcuucu.....................	1	0	S03
....caauuguuaugaucugcuucucauc.................	1	0	S02
....caauuguuaugaucugcuucucauc.................	1	0	S04
....caauuguuaugaucugcuucucauc.................	2	0	S03
....caauuguuaugaucugcuucucauc.................	1	0	S05
....aauuguuaugaucugcuucucauc.................	1	0	S04
....aauuguuaugaucugcuucucauc.................	5	0	S05
....aauuguuaugaucugcuucucauc.................	1	0	S02
....aauuguuaugaucugcuucucauc.................	5	0	S01
....aauuguuaugaucugcuucucauc.................	6	0	S06
....aauuguuaugaucugcuucucauc.................	3	0	S03
....aauuguuaugaucugcuucucaucu................	1	0	S04
....auuguuaugaucugcuucuca...................	1	0	S06
....auuguuaugaucugcuucucau..................	1	0	S03
....auuguuaugaucugcuucucauc.................	3	0	S01
....auuguuaugaucugcuucucauc.................	2	0	S02
....auuguuaugaucugcuucucauc.................	3	0	S06
....auuguuaugaucugcuucucauc.................	2	0	S05
....auuguuaugaucugcuucucauc.................	1	0	S04
....uguuaugaucugcuucucau..................	4	0	S04
....uguuaugaucugcuucucau..................	4	0	S02
....uguuaugaucugcuucucau..................	6	0	S03
....uguuaugaucugcuucucau..................	5	0	S01
....uguuaugaucugcuucucau..................	2	0	S05
....uguuaugaucugcuucucauc.................	250	0	S01
....uguuaugaucugcuucucauc.................	138	0	S06
....uguuaugaucugcuucucauc.................	137	0	S02
....uguuaugaucugcuucucauc.................	196	0	S03
....uguuaugaucugcuucucauc.................	230	0	S05
....uguuaugaucugcuucucauc.................	163	0	S04
....uguuaugaucugcuucucaucu................	1	0	S01
....uguuaugaucugcuucucaucug...............	2	0	S01
....uguuaugaucugcuucucaucug...............	6	0	S03
....uguuaugaucugcuucucaucug...............	6	0	S06
....uguuaugaucugcuucucaucug...............	2	0	S05
....uguuaugaucugcuucucaucug...............	1	0	S04
....uguuaugaucugcuucucaucug...............	7	0	S02
....uguuaugaucugcuucucaucuga..............	8	0	S04
....uguuaugaucugcuucucaucuga..............	11	0	S02
....uguuaugaucugcuucucaucuga..............	9	0	S03
....uguuaugaucugcuucucaucuga..............	16	0	S01
....uguuaugaucugcuucucaucuga..............	11	0	S05
....uguuaugaucugcuucucaucuga..............	12	0	S06
....guuaugaucugcuucucaucu................	1	0	S03
....guuaugaucugcuucucaucugaagacu.........	1	0	S06
....uuaugaucugcuucucauc.................	1	0	S01
....uuaugaucugcuucucauc.................	4	0	S04
....uuaugaucugcuucucauc.................	1	0	S02

```
                             Mature                                               Star
ccgcuaaacaagucuuuagaugagaagguagaucauaauagcugagcgaaaugaaagugauauuguugcucaauuguuaugaucugcuucucaucugaagacuaguuuauua
............................................................................uuaugaucugcuucucauc.................   1   0   S03
............................................................................uuaugaucugcuucucaucu................   3   0   S04
............................................................................uuaugaucugcuucucaucu................   2   0   S03
............................................................................uuaugaucugcuucucaucu................   3   0   S01
............................................................................uuaugaucugcuucucaucug...............   6   0   S04
............................................................................uuaugaucugcuucucaucug...............   1   0   S06
............................................................................uuaugaucugcuucucaucug...............   3   0   S03
............................................................................uuaugaucugcuucucaucug...............   5   0   S05
............................................................................uuaugaucugcuucucaucug...............   5   0   S01
............................................................................uuaugaucugcuucucaucug...............   4   0   S02
............................................................................uuaugaucugcuucucaucuga..............   1   0   S02
............................................................................uuaugaucugcuucucaucuga..............   1   0   S05
............................................................................uuaugaucugcuucucaucuga..............   1   0   S03
............................................................................uuaugaucugcuucucaucuga..............   1   0   S04
............................................................................uuaugaucugcuucucaucugaa.............   1   0   S03
............................................................................uuaugaucugcuucucaucugaag............   2   0   S01
............................................................................uuaugaucugcuucucaucugaag............   2   0   S02
.............................................................................uaugaucugcuucucauc.................   2   0   S03
.............................................................................uaugaucugcuucucaucug...............   1   0   S02
.............................................................................uaugaucugcuucucaucug...............   1   0   S06
.............................................................................uaugaucugcuucucaucug...............   2   0   S05
.............................................................................uaugaucugcuucucaucug...............   6   0   S03
.............................................................................uaugaucugcuucucaucuga..............   7   0   S01
.............................................................................uaugaucugcuucucaucuga..............   3   0   S05
.............................................................................uaugaucugcuucucaucuga..............   4   0   S04
.............................................................................uaugaucugcuucucaucuga..............   4   0   S03
.............................................................................uaugaucugcuucucaucuga..............   5   0   S02
.............................................................................uaugaucugcuucucaucugaaga...........   1   0   S02
..............................................................................augaucugcuucucaucug...............   8   0   S03
..............................................................................augaucugcuucucaucug...............   2   0   S06
..............................................................................augaucugcuucucaucuga..............   10  0   S02
..............................................................................augaucugcuucucaucuga..............   5   0   S05
..............................................................................augaucugcuucucaucuga..............   8   0   S06
..............................................................................augaucugcuucucaucuga..............   18  0   S01
..............................................................................augaucugcuucucaucuga..............   7   0   S03
..............................................................................augaucugcuucucaucuga..............   13  0   S04
..............................................................................augaucugcuucucaucugaa.............   1   0   S05
..............................................................................augaucugcuucucaucugaa.............   2   0   S02
..............................................................................augaucugcuucucaucugaa.............   1   0   S04
..............................................................................augaucugcuucucaucugaa.............   2   0   S01
..............................................................................augaucugcuucucaucugaa.............   1   0   S03
..............................................................................augaucugcuucucaucugaag............   1   0   S01
..............................................................................augaucugcuucucaucugaag............   1   0   S05
..............................................................................augaucugcuucucaucugaaga...........   2   0   S05
..............................................................................augaucugcuucucaucugaaga...........   1   0   S01
..............................................................................augaucugcuucucaucugaaga...........   2   0   S04
..............................................................................augaucugcuucucaucugaaga...........   1   0   S02
..............................................................................augaucugcuucucaucugaaga...........   2   0   S06
..............................................................................augaucugcuucucaucugaagac..........   1   0   S05
..............................................................................augaucugcuucucaucugaagac..........   2   0   S04
..............................................................................augaucugcuucucaucugaagac..........   2   0   S01
..............................................................................augaucugcuucucaucugaagac..........   2   0   S06
..............................................................................augaucugcuucucaucugaagac..........   2   0   S02
..............................................................................augaucugcuucucaucugaagacu.........   1   0   S04
...............................................................................ugaucugcuucucaucug...............   1   0   S01
...............................................................................ugaucugcuucucaucug...............   1   0   S05
...............................................................................ugaucugcuucucaucuga..............   1   0   S04
...............................................................................ugaucugcuucucaucuga..............   1   0   S05
...............................................................................ugaucugcuucucaucuga..............   2   0   S03
...............................................................................ugaucugcuucucaucuga..............   1   0   S06
...............................................................................ugaucugcuucucaucugaa.............   4   0   S05
...............................................................................ugaucugcuucucaucugaa.............   3   0   S02
...............................................................................ugaucugcuucucaucugaa.............   7   0   S06
...............................................................................ugaucugcuucucaucugaa.............   5   0   S03
...............................................................................ugaucugcuucucaucugaa.............   11  0   S01
...............................................................................ugaucugcuucucaucugaa.............   3   0   S04
...............................................................................ugaucugcuucucaucugaag............   37  0   S05
...............................................................................ugaucugcuucucaucugaag............   25  0   S03
...............................................................................ugaucugcuucucaucugaag............   28  0   S06
...............................................................................ugaucugcuucucaucugaag............   34  0   S01
```

Mature / Star			
ccgcuaaacaagucuuuagaugagaagguagaucauaauagcugagcgaaaugaaagugauauuguugcucaauuguuaugaucugcuucucaucugaagacuaguuuauua			
....ugaucugcuucucaucugaag............	25	0	S02
....ugaucugcuucucaucugaag............	55	0	S04
....ugaucugcuucucaucugaagac..........	1	0	S02
....ugaucugcuucucaucugaagacu.........	1	0	S04
....ugaucugcuucucaucugaagacu.........	3	0	S02
....ugaucugcuucucaucugaagacu.........	1	0	S06
....gaucugcuucucaucuga..............	1	0	S03
....gaucugcuucucaucugaag............	1	0	S04
....gaucugcuucucaucugaag............	1	0	S05
....gaucugcuucucaucugaaga...........	1	0	S03
....aucugcuucucaucugaa.............	1	0	S06
....aucugcuucucaucugaag............	4	0	S04
....aucugcuucucaucugaag............	6	0	S05
....aucugcuucucaucugaag............	10	0	S01
....aucugcuucucaucugaag............	18	0	S03
....aucugcuucucaucugaag............	5	0	S02
....aucugcuucucaucugaag............	35	0	S06
....aucugcuucucaucugaaga...........	1	0	S06
....aucugcuucucaucugaaga...........	5	0	S04
....aucugcuucucaucugaaga...........	5	0	S01
....aucugcuucucaucugaaga...........	11	0	S05
....aucugcuucucaucugaaga...........	2	0	S02
....aucugcuucucaucugaaga...........	3	0	S03
....aucugcuucucaucugaagac..........	7	0	S05
....aucugcuucucaucugaagac..........	4	0	S06
....aucugcuucucaucugaagac..........	1	0	S01
....aucugcuucucaucugaagac..........	4	0	S04
....aucugcuucucaucugaagac..........	1	0	S03
....aucugcuucucaucugaagacuag.......	1	0	S02
....ucugcuucucaucugaag............	2	0	S04
....ucugcuucucaucugaaga...........	1	0	S02
....ucugcuucucaucugaagac..........	1	0	S01
....ucugcuucucaucugaagacu.........	2	0	S02
....ucugcuucucaucugaagacu.........	5	0	S01
....ucugcuucucaucugaagacu.........	2	0	S05
....ucugcuucucaucugaagacu.........	1	0	S04
....ucugcuucucaucugaagacu.........	1	0	S03
....ucugcuucucaucugaagacu.........	2	0	S06
....ugcuucucaucugaagacu.........	2	0	S06
....ugcuucucaucugaagacu.........	2	0	S03
....ugcuucucaucugaagacu.........	1	0	S04
....ugcuucucaucugaagacua........	1	0	S06
....ugcuucucaucugaagacuag.......	2	0	S02
....ugcuucucaucugaagacuag.......	1	0	S01
....ugcuucucaucugaagacuag.......	2	0	S04
....ugcuucucaucugaagacuag.......	4	0	S05
....ugcuucucaucugaagacuag.......	3	0	S03
....ugcuucucaucugaagacuagu......	2	0	S05
....ugcuucucaucugaagacuagu......	1	0	S06
....ugcuucucaucugaagacuagu......	3	0	S01
....ugcuucucaucugaagacuagu......	2	0	S03
....ugcuucucaucugaagacuagu......	3	0	S04
....ugcuucucaucugaagacuaguuuauu.	1	0	S04
....gcuucucaucugaagacuaguuuauu.	1	0	S02
....gcuucucaucugaagacuaguuuauu.	1	0	S06
....gcuucucaucugaagacuaguuuauu.	1	0	S03
....cuucucaucugaagacuag.......	1	0	S02
....cuucucaucugaagacuag.......	1	0	S04
....cuucucaucugaagacuag.......	1	0	S01
....cuucucaucugaagacuag.......	1	0	S03
....cuucucaucugaagacuagu......	7	0	S01
....cuucucaucugaagacuagu......	5	0	S05
....cuucucaucugaagacuagu......	7	0	S04
....cuucucaucugaagacuagu......	5	0	S06
....cuucucaucugaagacuagu......	4	0	S02
....cuucucaucugaagacuaguu.....	1	0	S05
....cuucucaucugaagacuaguu.....	2	0	S06
....cuucucaucugaagacuaguu.....	3	0	S02
....cuucucaucugaagacuaguu.....	1	0	S01
....cuucucaucugaagacuaguu.....	1	0	S04

Mature **Star**

ccgcuaaacaagucuuuagaugagaagguagaucauaauagcugagcgaaaugaaagugauauuguugcucaauuguuaugaucugcuucucaucugaagacuaguuuauua

....cuucucaucugaagacuaguuuauu.	1	0	S01
....uucucaucugaagacuag.......	1	0	S02
....uucucaucugaagacuag.......	1	0	S01
....uucucaucugaagacuag.......	5	0	S06
....uucucaucugaagacuag.......	1	0	S03
....uucucaucugaagacuag.......	4	0	S05
....uucucaucugaagacuagu......	6	0	S04
....uucucaucugaagacuagu......	5	0	S05
....uucucaucugaagacuagu......	5	0	S06
....uucucaucugaagacuagu......	6	0	S02
....uucucaucugaagacuagu......	3	0	S01
....uucucaucugaagacuagu......	2	0	S03
....uucucaucugaagacuaguu.....	12	0	S03
....uucucaucugaagacuaguu.....	29	0	S06
....uucucaucugaagacuaguu.....	20	0	S02
....uucucaucugaagacuaguu.....	16	0	S01
....uucucaucugaagacuaguu.....	24	0	S04
....uucucaucugaagacuaguu.....	34	0	S05
....uucucaucugaagacuaguuu....	6	0	S06
....uucucaucugaagacuaguuu....	16	0	S02
....uucucaucugaagacuaguuu....	9	0	S03
....uucucaucugaagacuaguuu....	19	0	S01
....uucucaucugaagacuaguuu....	26	0	S04
....uucucaucugaagacuaguuu....	39	0	S05
....uucucaucugaagacuaguuua...	30	0	S02
....uucucaucugaagacuaguuua...	45	0	S05
....uucucaucugaagacuaguuua...	39	0	S04
....uucucaucugaagacuaguuua...	18	0	S03
....uucucaucugaagacuaguuua...	33	0	S01
....uucucaucugaagacuaguuua...	17	0	S06
....uucucaucugaagacuaguuuau..	1	0	S03
....uucucaucugaagacuaguuuau..	1	0	S01
....uucucaucugaagacuaguuuau..	1	0	S05
....uucucaucugaagacuaguuuauu.	1	0	S01
....ucucaucugaagacuagu......	1	0	S01
....ucucaucugaagacuagu......	1	0	S04
....ucucaucugaagacuagu......	2	0	S05
....ucucaucugaagacuagu......	1	0	S03
....ucucaucugaagacuaguu.....	1	0	S03
....ucucaucugaagacuaguu.....	2	0	S01
....ucucaucugaagacuaguu.....	1	0	S05
....ucucaucugaagacuaguu.....	2	0	S04
....ucucaucugaagacuaguu.....	2	0	S06
....ucucaucugaagacuaguuu....	6	0	S04
....ucucaucugaagacuaguuu....	5	0	S03
....ucucaucugaagacuaguuu....	5	0	S01
....ucucaucugaagacuaguuu....	5	0	S02
....ucucaucugaagacuaguuu....	8	0	S05
....ucucaucugaagacuaguuu....	3	0	S06
....ucucaucugaagacuaguuua...	11	0	S03
....ucucaucugaagacuaguuua...	37	0	S05
....ucucaucugaagacuaguuua...	13	0	S06
....ucucaucugaagacuaguuua...	25	0	S04
....ucucaucugaagacuaguuua...	19	0	S02
....ucucaucugaagacuaguuua...	15	0	S01
....ucucaucugaagacuaguuuauu.	1	0	S05
....ucaucugaagacuaguuu....	2	0	S05
....ucaucugaagacuaguuu....	2	0	S04
....ucaucugaagacuaguuu....	1	0	S02
....ucaucugaagacuaguuua...	1	0	S03
....ucaucugaagacuaguuua...	1	0	S05
....ucaucugaagacuaguuua...	3	0	S01
....ucaucugaagacuaguuuau..	5	0	S03
....ucaucugaagacuaguuuau..	4	0	S04
....ucaucugaagacuaguuuau..	4	0	S05
....ucaucugaagacuaguuuau..	1	0	S02
....ucaucugaagacuaguuuau..	2	0	S06
....ucaucugaagacuaguuuau..	5	0	S01
....ucaucugaagacuaguuuauu.	24	0	S01
....ucaucugaagacuaguuuauu.	32	0	S03

```
                    Mature                                                 Star
ccgcuaaacaagucuuuagaugagaagguagaucauaauagcugagcgaaaugaaagugauauuguugcucaauuguuaugaucugcuucucaucugaagacuaguuuauua
..........................................................................................ucaucugaagacuaguuuauu.    23    0    S02
..........................................................................................ucaucugaagacuaguuuauu.    29    0    S05
..........................................................................................ucaucugaagacuaguuuauu.    30    0    S04
..........................................................................................ucaucugaagacuaguuuauu.    13    0    S06
..........................................................................................ucaucugaagacuaguuuauua    1     0    S05
.............................................................................................ucugaagacuaguuuauu.    3     0    S06
.............................................................................................ucugaagacuaguuuauu.    1     0    S04
.............................................................................................ucugaagacuaguuuauu.    5     0    S05
.............................................................................................ucugaagacuaguuuauu.    1     0    S01
.............................................................................................ucugaagacuaguuuauu.    3     0    S02
.............................................................................................ucugaagacuaguuuauua    1     0    S03
.............................................................................................ucugaagacuaguuuauua    3     0    S04
.............................................................................................ucugaagacuaguuuauua    1     0    S02
.............................................................................................ucugaagacuaguuuauua    1     0    S05
.............................................................................................ucugaagacuaguuuauua    3     0    S06
.............................................................................................ucugaagacuaguuuauua    1     0    S01
```

Provisional ID	:	ta_iwgsc_5ds_v1_2769792_5536271
Score total	:	5833.2
Score for star read(s)	:	3.9
Score for read counts	:	5825.4
Score for mfe	:	2.3
Score for randfold	:	1.6
Score for cons. seed	:	
Total read count	:	11438
Mature read count	:	12937
Loop read count	:	0
Star read count	:	188

bdi-miR167g (-5p)

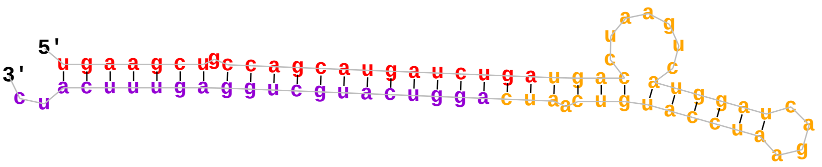

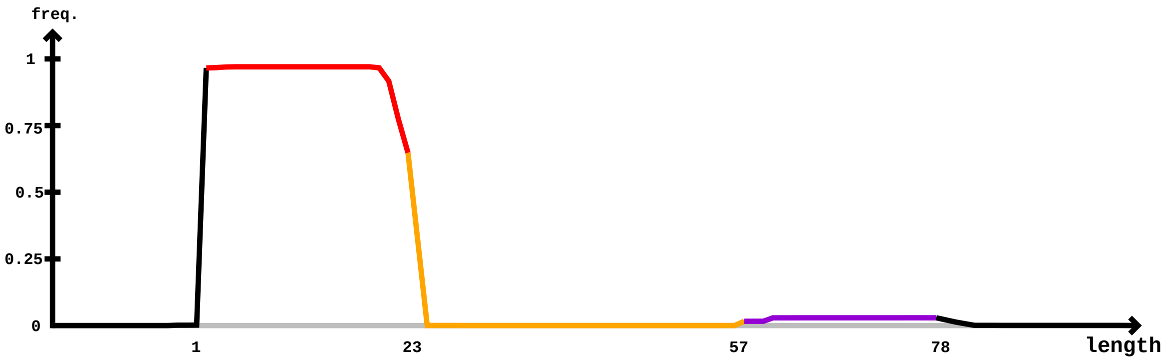

Mature Star

```
5'- gcaccacaagcugguugaagcugccagcaugaucugaugaccuaaagucauggaucagaauccaugucaaucaggucaugcuggaguuucaucugcuggucggagcacaacga   -3'   obs
    gcaccacaagcugguugaagcugccagcaugaucugaugaccuaaagucauggaucagaauccaugucaaucaggucaugcuggaguuucaucugcuggucggagcacaacga         exp
    ((.((((.(((.(((((((((.((((((((((((((((((.......((((((....))))))))).)))))))))))))))))))))))))).))).)).)).)).......   reads   mm   sample
    ...........uggugaagcugccagcaugau................................................................................   1       0    S05
    ...........uggugaagcugccagcaugauc...............................................................................   2       0    S03
    ...........uggugaagcugccagcaugauc...............................................................................   3       0    S04
    ...........uggugaagcugccagcaugauc...............................................................................   1       0    S02
    ...........uggugaagcugccagcaugauc...............................................................................   4       0    S06
    ...........uggugaagcugccagcaugauc...............................................................................   4       0    S05
    ...........uggugaagcugccagcaugaucuga............................................................................   1       0    S05
    .............gugaagcugccagcaugauc...............................................................................   2       0    S01
    .............gugaagcugccagcaugaucuga............................................................................   1       0    S04
    ..............ugaagcugccagcaugau................................................................................   8       0    S06
    ..............ugaagcugccagcaugau................................................................................   6       0    S03
    ..............ugaagcugccagcaugau................................................................................   10      0    S04
    ..............ugaagcugccagcaugau................................................................................   7       0    S01
    ..............ugaagcugccagcaugau................................................................................   4       0    S02
    ..............ugaagcugccagcaugau................................................................................   9       0    S05
    ..............ugaagcugccagcaugauc...............................................................................   174     0    S05
    ..............ugaagcugccagcaugauc...............................................................................   80      0    S01
    ..............ugaagcugccagcaugauc...............................................................................   121     0    S04
    ..............ugaagcugccagcaugauc...............................................................................   78      0    S03
    ..............ugaagcugccagcaugauc...............................................................................   53      0    S06
    ..............ugaagcugccagcaugauc...............................................................................   45      0    S02
    ..............ugaagcugccagcaugaucu..............................................................................   237     0    S01
    ..............ugaagcugccagcaugaucu..............................................................................   218     0    S03
    ..............ugaagcugccagcaugaucu..............................................................................   353     0    S05
    ..............ugaagcugccagcaugaucu..............................................................................   173     0    S02
    ..............ugaagcugccagcaugaucu..............................................................................   387     0    S04
    ..............ugaagcugccagcaugaucu..............................................................................   262     0    S06
    ..............ugaagcugccagcaugaucug.............................................................................   417     0    S06
    ..............ugaagcugccagcaugaucug.............................................................................   279     0    S05
    ..............ugaagcugccagcaugaucug.............................................................................   137     0    S02
    ..............ugaagcugccagcaugaucug.............................................................................   119     0    S01
    ..............ugaagcugccagcaugaucug.............................................................................   299     0    S03
    ..............ugaagcugccagcaugaucug.............................................................................   176     0    S04
```

Mature Star

```
gcaccacaagcuggugaagcugccagcaugaucugaugaccuaagucauggaucagaauccaugucaaucaggucaugcuggaguuucaucugcuggucggagcacaacga
..............ugaagcugccagcaugaucuga...........................................................................   1745   0   S04
..............ugaagcugccagcaugaucuga...........................................................................   923    0   S06
..............ugaagcugccagcaugaucuga...........................................................................   984    0   S02
..............ugaagcugccagcaugaucuga...........................................................................   2357   0   S05
..............ugaagcugccagcaugaucuga...........................................................................   519    0   S03
..............ugaagcugccagcaugaucuga...........................................................................   870    0   S01
..............ugaagcugccagcaugaucugau..........................................................................   1      0   S05
..............ugaagcugccagcaugaucugau..........................................................................   1      0   S03
..............ugaagcugccagcaugaucugau..........................................................................   1      0   S04
..............ugaagcugccagcaugaucugau..........................................................................   1      0   S01
..............ugaagcugccagcaugaucugaugacc......................................................................   1      0   S01
...............gaagcugccagcaugauc..............................................................................   1      0   S04
...............gaagcugccagcaugauc..............................................................................   3      0   S03
...............gaagcugccagcaugauc..............................................................................   1      0   S05
...............gaagcugccagcaugaucu.............................................................................   3      0   S03
...............gaagcugccagcaugaucu.............................................................................   1      0   S05
...............gaagcugccagcaugaucug............................................................................   1      0   S05
...............gaagcugccagcaugaucuga...........................................................................   1      0   S01
................aagcugccagcaugaucu.............................................................................   1      0   S02
................aagcugccagcaugaucu.............................................................................   1      0   S01
................aagcugccagcaugaucu.............................................................................   1      0   S04
................aagcugccagcaugaucug............................................................................   3      0   S05
................aagcugccagcaugaucug............................................................................   7      0   S06
................aagcugccagcaugaucug............................................................................   1      0   S04
................aagcugccagcaugaucug............................................................................   1      0   S02
................aagcugccagcaugaucug............................................................................   2      0   S03
................aagcugccagcaugaucuga...........................................................................   1      0   S01
................aagcugccagcaugaucuga...........................................................................   1      0   S05
................aagcugccagcaugaucuga...........................................................................   6      0   S03
................aagcugccagcaugaucuga...........................................................................   3      0   S06
.................agcugccagcaugaucug............................................................................   2      0   S05
.................agcugccagcaugaucug............................................................................   1      0   S03
.................agcugccagcaugaucuga...........................................................................   2      0   S04
.................agcugccagcaugaucuga...........................................................................   1      0   S05
.................agcugccagcaugaucuga...........................................................................   3      0   S06
......................................................................aggucaugcuggaguuuca......................   1      0   S05
......................................................................aggucaugcuggaguuucauc....................   28     0   S01
......................................................................aggucaugcuggaguuucauc....................   50     0   S03
......................................................................aggucaugcuggaguuucauc....................   39     0   S04
......................................................................aggucaugcuggaguuucauc....................   26     0   S05
......................................................................aggucaugcuggaguuucauc....................   16     0   S02
......................................................................aggucaugcuggaguuucauc....................   23     0   S06
......................................................................aggucaugcuggaguuucaucugc.................   1      0   S05
.......................................................................ggucaugcuggaguuuca......................   1      0   S05
.......................................................................ggucaugcuggaguuucauc....................   1      0   S06
.........................................................................ucaugcuggaguuucauc....................   1      0   S05
.........................................................................ucaugcuggaguuucauc....................   1      0   S04
.........................................................................ucaugcuggaguuucaucu...................   1      0   S05
.........................................................................ucaugcuggaguuucaucug..................   20     0   S05
.........................................................................ucaugcuggaguuucaucug..................   4      0   S02
.........................................................................ucaugcuggaguuucaucug..................   13     0   S04
.........................................................................ucaugcuggaguuucaucug..................   3      0   S01
.........................................................................ucaugcuggaguuucaucug..................   27     0   S06
.........................................................................ucaugcuggaguuucaucug..................   6      0   S03
.........................................................................ucaugcuggaguuucaucugc.................   1      0   S03
.........................................................................ucaugcuggaguuucaucugc.................   14     0   S01
.........................................................................ucaugcuggaguuucaucugc.................   25     0   S05
.........................................................................ucaugcuggaguuucaucugc.................   14     0   S04
.........................................................................ucaugcuggaguuucaucugc.................   6      0   S02
.........................................................................ucaugcuggaguuucaucugc.................   10     0   S06
............................................................................ugcuggaguuucaucugcugg..............   1      0   S04
............................................................................ugcuggaguuucaucugcugg..............   1      0   S05
.......................................................................................caucugcuggucggagcacaacg.   1      0   S03
...........................................................................................ugcuggucggagcacaacg.   1      0   S04
...........................................................................................ugcuggucggagcacaacga   3      0   S05
...........................................................................................ugcuggucggagcacaacga   1      0   S06
```

Provisional ID	:	ta_iwgsc_6dl_v1_3241952_6251670
Score total	:	1.8
Score for star read(s)	:	-1.3
Score for read counts	:	0
Score for mfe	:	1.5
Score for randfold	:	1.6
Score for cons. seed	:	
Total read count	:	219
Mature read count	:	200
Loop read count	:	0
Star read count	:	52

cme-miR166e

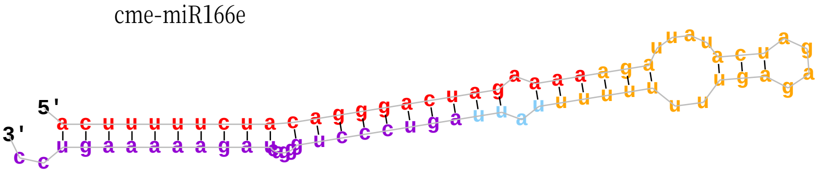

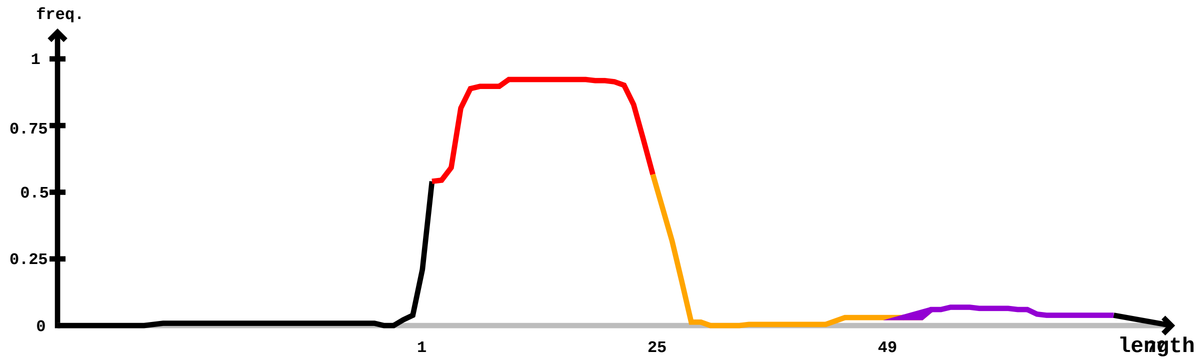


```
                                     Mature                                            Star
5'- aaggucccuaaaaagucccuaaaaaccaaacggggagacuuuuucuacagggacuagaaaaagauuauacuagagaguuuuuuuuuauuagucccugggacuagaaaaaguccu  -3'  obs
    aaggucccuaaaaagucccuaaaaaccaaacggggagacuuuuucuacagggacuagaaaaagauuauacuagagaguuuuuuuuuauuagucccugggacuagaaaaaguccu       exp
    ...............(((((...........)))))(((((((((((((((((((((.((((((....(((....)))..)))))).))))))))))....)))))))))))..   reads   mm   sample
    ........uaaaaagucccuaaaaaccaaacg..................................................................................   1       0    S06
    .........aaaaagucccuaaaaaccaaacg..................................................................................   1       0    S06
    ..................................gagacuuuuucuacagggacuag.........................................................   1       0    S06
    ..................................gagacuuuuucuacagggacuaga........................................................   1       0    S04
    ..................................gagacuuuuucuacagggacuaga........................................................   1       0    S06
    ..................................gagacuuuuucuacagggacuaga........................................................   2       0    S05
    ...................................agacuuuuucuacagggacuaga........................................................   1       0    S05
    ...................................agacuuuuucuacagggacuaga........................................................   1       0    S06
    ...................................agacuuuuucuacagggacuagaa.......................................................   1       0    S05
    ...................................agacuuuuucuacagggacuagaa.......................................................   1       0    S06
    ....................................gacuuuuucuacagggac............................................................   1       0    S04
    ....................................gacuuuuucuacagggacuaga........................................................   1       0    S04
    ....................................gacuuuuucuacagggacuaga........................................................   2       0    S01
    ....................................gacuuuuucuacagggacuagaa.......................................................   1       0    S03
    ....................................gacuuuuucuacagggacuagaa.......................................................   2       0    S04
    ....................................gacuuuuucuacagggacuagaa.......................................................   2       0    S02
    ....................................gacuuuuucuacagggacuagaa.......................................................   1       0    S01
    ....................................gacuuuuucuacagggacuagaa.......................................................   5       0    S05
    ....................................gacuuuuucuacagggacuagaa.......................................................   9       0    S06
    ....................................gacuuuuucuacagggacuagaaa......................................................   1       0    S01
    ....................................gacuuuuucuacagggacuagaaa......................................................   1       0    S06
    ....................................gacuuuuucuacagggacuagaaa......................................................   7       0    S05
    ....................................gacuuuuucuacagggacuagaaa......................................................   2       0    S02
    ....................................gacuuuuucuacagggacuagaaa......................................................   3       0    S04
    ....................................gacuuuuucuacagggacuagaaa......................................................   1       0    S03
    ....................................gacuuuuucuacagggacuagaaaa.....................................................   1       0    S06
    .....................................acuuuuucuacagggacua..........................................................   1       0    S03
    .....................................acuuuuucuacagggacuag.........................................................   1       0    S06
    .....................................acuuuuucuacagggacuaga........................................................   1       0    S02
    .....................................acuuuuucuacagggacuaga........................................................   3       0    S05
    .....................................acuuuuucuacagggacuaga........................................................   1       0    S06
    .....................................acuuuuucuacagggacuagaa.......................................................   2       0    S03
    .....................................acuuuuucuacagggacuagaaa......................................................   2       0    S04
```

```
                                     Mature                                           Star
aaggucccuaaaaagucccuaaaaaccaaacggggagacuuuuucuacagggacuagaaaaagauuauacuagagaguuuuuuuuuauuagucccugggacuagaaaaaguccu
.....................................acuuuuucuacagggacuagaaa......................................................    4    0    S03
.....................................acuuuuucuacagggacuagaaa......................................................    2    0    S02
.....................................acuuuuucuacagggacuagaaa......................................................    3    0    S05
.....................................acuuuuucuacagggacuagaaa......................................................    1    0    S06
.....................................acuuuuucuacagggacuagaaa......................................................    1    0    S01
.....................................acuuuuucuacagggacuagaaaa.....................................................    1    0    S03
.....................................acuuuuucuacagggacuagaaaa.....................................................    17   0    S04
.....................................acuuuuucuacagggacuagaaaa.....................................................    7    0    S06
.....................................acuuuuucuacagggacuagaaaa.....................................................    4    0    S01
.....................................acuuuuucuacagggacuagaaaa.....................................................    6    0    S02
.....................................acuuuuucuacagggacuagaaaa.....................................................    20   0    S05
......................................cuuuuucuacagggacuaga........................................................    1    0    S06
.......................................uuuuucuacagggacuag.........................................................    1    0    S04
.......................................uuuuucuacagggacuaga........................................................    1    0    S01
.......................................uuuuucuacagggacuaga........................................................    1    0    S04
.......................................uuuuucuacagggacuagaa.......................................................    1    0    S03
.......................................uuuuucuacagggacuagaa.......................................................    1    0    S06
.......................................uuuuucuacagggacuagaa.......................................................    2    0    S05
.......................................uuuuucuacagggacuagaaa......................................................    1    0    S02
.......................................uuuuucuacagggacuagaaa......................................................    1    0    S04
.......................................uuuuucuacagggacuagaaaaag...................................................    1    0    S04
.......................................uuuuucuacagggacuagaaaaag...................................................    1    0    S05
........................................uuuucuacagggacuagaaa......................................................    1    0    S06
........................................uuuucuacagggacuagaaaaag...................................................    4    0    S04
........................................uuuucuacagggacuagaaaaag...................................................    8    0    S06
........................................uuuucuacagggacuagaaaaag...................................................    5    0    S02
........................................uuuucuacagggacuagaaaaag...................................................    4    0    S01
........................................uuuucuacagggacuagaaaaag...................................................    4    0    S05
........................................uuuucuacagggacuagaaaaag...................................................    3    0    S03
........................................uuuucuacagggacuagaaaaaga..................................................    2    0    S02
........................................uuuucuacagggacuagaaaaaga..................................................    3    0    S01
........................................uuuucuacagggacuagaaaaaga..................................................    9    0    S05
........................................uuuucuacagggacuagaaaaaga..................................................    7    0    S04
........................................uuuucuacagggacuagaaaaaga..................................................    2    0    S06
.........................................uuucuacagggacuagaa.......................................................    1    0    S05
.........................................uuucuacagggacuagaa.......................................................    1    0    S03
.........................................uuucuacagggacuagaaaaa....................................................    1    0    S05
.........................................uuucuacagggacuagaaaaag...................................................    2    0    S04
.........................................uuucuacagggacuagaaaaag...................................................    1    0    S06
.........................................uuucuacagggacuagaaaaaga..................................................    3    0    S04
.........................................uuucuacagggacuagaaaaaga..................................................    1    0    S01
.........................................uuucuacagggacuagaaaaaga..................................................    1    0    S03
.........................................uuucuacagggacuagaaaaaga..................................................    4    0    S05
.........................................uuucuacagggacuagaaaaaga..................................................    2    0    S02
..........................................uucuacagggacuagaaaa.....................................................    1    0    S06
..........................................uucuacagggacuagaaaaag...................................................    1    0    S04
.............................................uacagggacuagaaaaag...................................................    1    0    S02
.............................................uacagggacuagaaaaaga..................................................    1    0    S04
.............................................uacagggacuagaaaaaga..................................................    1    0    S06
.............................................uacagggacuagaaaaagauu................................................    3    0    S04
......................................................................uagagaguuuuuuuuuauuagucc....................    1    0    S06
...............................................................................uuuuuuuauuagucccugg................    1    0    S05
...............................................................................uuuuuuuauuagucccuggga..............    1    0    S04
...............................................................................uuuuuuuauuagucccugggac.............    1    0    S04
................................................................................uuuuuuauuagucccuggga..............    1    0    S05
................................................................................uuuuuuauuagucccuggga..............    2    0    S04
.........................................................................................agucccugggacuagaaaaaguc..    1    0    S05
.........................................................................................agucccugggacuagaaaaagucc.    1    0    S01
.........................................................................................agucccugggacuagaaaaagucc.    2    0    S05
.........................................................................................agucccugggacuagaaaaagucc.    1    0    S03
.........................................................................................agucccugggacuagaaaaagucc.    2    0    S04
...........................................................................................ucccugggacuagaaaaaguc..    1    0    S03
...........................................................................................ucccugggacuagaaaaaguc..    1    0    S04
```

Provisional ID	:	ta_iwgsc_7bs_v1_3165007_7183033
Score total	:	60655.9
Score for star read(s)	:	3.9
Score for read counts	:	60648.4
Score for mfe	:	2
Score for randfold	:	1.6
Score for cons. seed	:	
Total read count	:	118971
Mature read count	:	116770
Loop read count	:	0
Star read count	:	2069

lja-miR396

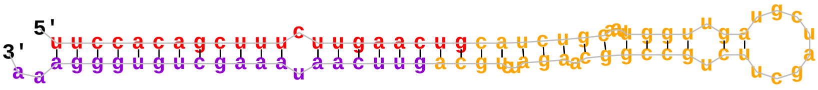

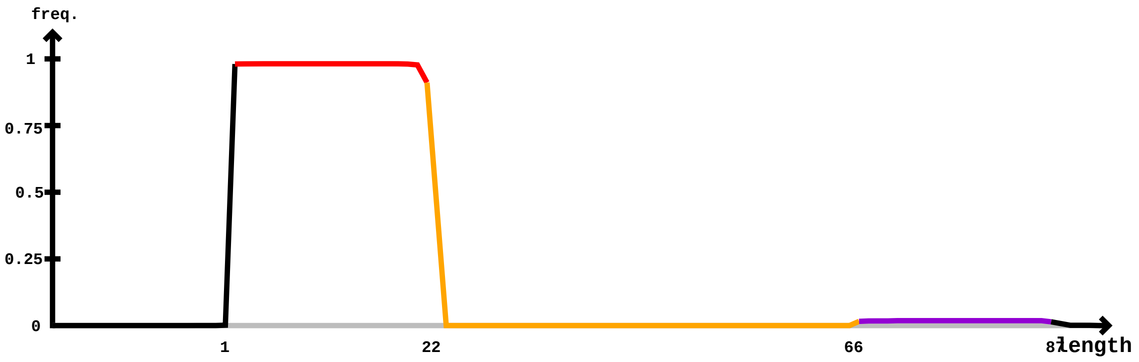

Mature **Star**

5'- uggcccucuuugcugucuuccacagcuuucuugaacugcaucugcaauugguugaugcuagcuucugccggcaagaucugcaguucaauaaagcugugggaaauugca -3'	obs		
uggcccucuuugcugucuuccacagcuuucuugaacugcaucugcaauugguugaugcuagcuucugccggcaagaucugcaguucaauaaagcugugggaaauugca	exp		
..........(((.((.((((((((((((.(((((((((((((((...((((.((........)).)))))).)))..)))))))))).)))))))))))).)).)))	reads	mm	sample
............cugucuuccacagcuuucuugaacu...	1	0	S04
.............ugucuuccacagcuuucuuga..	1	0	S03
.............ugucuuccacagcuuucuuga..	1	0	S06
.............ugucuuccacagcuuucuuga..	5	0	S05
...............ucuuccacagcuuucuugaac..	1	0	S01
...............ucuuccacagcuuucuugaac..	2	0	S02
...............ucuuccacagcuuucuugaacu...	1	0	S05
...............ucuuccacagcuuucuugaacu...	1	0	S03
...............ucuuccacagcuuucuugaacug..	1	0	S02
................cuuccacagcuuucuugaac..	2	0	S04
................cuuccacagcuuucuugaacu...	2	0	S01
................cuuccacagcuuucuugaacu...	3	0	S04
................cuuccacagcuuucuugaacu...	3	0	S05
................cuuccacagcuuucuugaacu...	3	0	S06
................cuuccacagcuuucuugaacu...	3	0	S02
................cuuccacagcuuucuugaacug..	16	0	S04
................cuuccacagcuuucuugaacug..	30	0	S01
................cuuccacagcuuucuugaacug..	28	0	S06
................cuuccacagcuuucuugaacug..	9	0	S03
................cuuccacagcuuucuugaacug..	42	0	S05
................cuuccacagcuuucuugaacug..	12	0	S02
................cuuccacagcuuucuugaacugc...	1	0	S05
.................uuccacagcuuucuugaa...	10	0	S04
.................uuccacagcuuucuugaa...	28	0	S03
.................uuccacagcuuucuugaa...	21	0	S05
.................uuccacagcuuucuugaa...	13	0	S02
.................uuccacagcuuucuugaa...	32	0	S06
.................uuccacagcuuucuugaa...	9	0	S01
.................uuccacagcuuucuugaac..	73	0	S06
.................uuccacagcuuucuugaac..	39	0	S02
.................uuccacagcuuucuugaac..	42	0	S01
.................uuccacagcuuucuugaac..	58	0	S03
.................uuccacagcuuucuugaac..	113	0	S05

Mature **Star**

uggcccucuuugcugucuuccacagcuuucuugaacugcaucugcaauugguugaugcuagcuucugccggcaagaucugcaguucaauaaagcugugggaaauugca			
.................uuccacagcuuucuugaac..	45	0	S04
.................uuccacagcuuucuugaacu...	1973	0	S06
.................uuccacagcuuucuugaacu...	956	0	S02
.................uuccacagcuuucuugaacu...	1056	0	S03
.................uuccacagcuuucuugaacu...	793	0	S04
.................uuccacagcuuucuugaacu...	942	0	S01
.................uuccacagcuuucuugaacu...	2112	0	S05
.................uuccacagcuuucuugaacug..	15879	0	S04
.................uuccacagcuuucuugaacuU..	51	1	S05
.................uuccacagcuuucuugaacug..	29088	0	S05
.................uuccacagcuuucuugaacuU..	24	1	S01
.................uuccacagcuuucuugaacuU..	9	1	S03
.................uuccacagcuuucuugaacuU..	28	1	S06
.................uuccacagcuuucuugaacug..	16913	0	S01
.................uuccacagcuuucuugaacug..	22186	0	S06
.................uuccacagcuuucuugaacug..	13467	0	S02
.................uuccacagcuuucuugaacug..	10253	0	S03
.................uuccacagcuuucuugaacuU..	23	1	S04
.................uuccacagcuuucuugaacuU..	34	1	S02
.................uuccacagcuuucuugaacuUc...	1	1	S01
.................uuccacagcuuucuugaacuUc...	3	1	S05
.................uuccacagcuuucuugaacugc...	17	0	S02
.................uuccacagcuuucuugaacugc...	47	0	S06
.................uuccacagcuuucuugaacuUc...	1	1	S02
.................uuccacagcuuucuugaacugc...	90	0	S05
.................uuccacagcuuucuugaacugc...	37	0	S01
.................uuccacagcuuucuugaacugc...	29	0	S04
.................uuccacagcuuucuugaacugc...	26	0	S03
.................uuccacagcuuucuugaacuUc...	3	1	S04
.................uuccacagcuuucuugaacugca..	1	0	S01
.................uuccacagcuuucuugaacugca..	1	0	S06
.................uuccacagcuuucuugaacugca..	1	0	S05
.................uuccacagcuuucuugaacugca..	1	0	S03
.................uuccacagcuuucuugaacugcaucugc...	1	0	S04
..................uccacagcuuucuugaacu...	3	0	S04
..................uccacagcuuucuugaacu...	3	0	S03
..................uccacagcuuucuugaacu...	1	0	S05
..................uccacagcuuucuugaacu...	2	0	S06
..................uccacagcuuucuugaacu...	1	0	S01
..................uccacagcuuucuugaacu...	1	0	S02
..................uccacagcuuucuugaacuU..	1	1	S04
..................uccacagcuuucuugaacug..	10	0	S06
..................uccacagcuuucuugaacug..	6	0	S01
..................uccacagcuuucuugaacug..	4	0	S02
..................uccacagcuuucuugaacug..	9	0	S04
..................uccacagcuuucuugaacug..	6	0	S05
..................uccacagcuuucuugaacug..	3	0	S03
...................ccacagcuuucuugaacu...	1	0	S05
...................ccacagcuuucuugaacu...	2	0	S03
...................ccacagcuuucuugaacu...	2	0	S06
...................ccacagcuuucuugaacu...	1	0	S01
...................ccacagcuuucuugaacu...	5	0	S02
...................ccacagcuuucuugaacug..	5	0	S06
...................ccacagcuuucuugaacug..	2	0	S03
...................ccacagcuuucuugaacug..	3	0	S05
...................ccacagcuuucuugaacug..	3	0	S01
...................ccacagcuuucuugaacug..	2	0	S04
...................ccacagcuuucuugaacugca..	2	0	S04
....................cacagcuuucuugaacug..	1	0	S03
....................cacagcuuucuugaacug..	4	0	S04
....................cacagcuuucuugaacug..	2	0	S06
....................cacagcuuucuugaacug..	1	0	S02
....................cacagcuuucuugaacug..	2	0	S01
....................cacagcuuucuugaacug..	5	0	S05
........................gcuuucuugaacugcaucugcaa...	1	0	S06
........................gcuuucuugaacugcaucugcaau..	2	0	S06
..........................uuucuugaacugcaucugcaauu...	1	0	S06
....aguucaauaaagcugugggaa......	2	0	S04
....guucaauaaagcuguggg........	2	0	S06
....guucaauaaagcuguggg........	1	0	S04

Mature **Star**

```
uggcccucuuugcugucuuccacagcuuucuugaacugcaucugcaauugguugaugcuagcuucugccggcaagaucugcaguucaauaaagcugugggaaauugca
..................................................................................guucaauaaagcuguggga.......	4	0	S05
..................................................................................guucaauaaagcuguggga.......	5	0	S06
..................................................................................guucaauaaagcuguggga.......	2	0	S03
..................................................................................guucaauaaagcuguggga.......	1	0	S02
..................................................................................guucaauaaagcugugggaa......	62	0	S06
..................................................................................guucaauaaagcugugggaa......	68	0	S02
..................................................................................guucaauaaagcugugggaa......	65	0	S05
..................................................................................guucaauaaagcugugggaa......	118	0	S04
..................................................................................guucaauaaagcugugggaa......	72	0	S01
..................................................................................guucaauaaagcugugggaa......	66	0	S03
..................................................................................guucaauaaagcugugggaaG.....	1	1	S04
..................................................................................guucaauaaagcugugggaaa.....	379	0	S04
..................................................................................guucaauaaagcugugggaaa.....	173	0	S03
..................................................................................guucaauaaagcugugggaaa.....	201	0	S01
..................................................................................guucaauaaagcugugggaaa.....	109	0	S06
..................................................................................guucaauaaagcugugggaaa.....	347	0	S05
..................................................................................guucaauaaagcugugggaaa.....	202	0	S02
..................................................................................guucaauaaagcugugggaaG.....	1	1	S06
...................................................................................uucaauaaagcuguggga.......	1	0	S04
...................................................................................uucaauaaagcuguggga.......	1	0	S06
...................................................................................uucaauaaagcugugggaa......	4	0	S03
...................................................................................uucaauaaagcugugggaa......	4	0	S01
...................................................................................uucaauaaagcugugggaa......	8	0	S04
...................................................................................uucaauaaagcugugggaa......	7	0	S02
...................................................................................uucaauaaagcugugggaa......	10	0	S05
...................................................................................uucaauaaagcugugggaa......	8	0	S06
...................................................................................uucaauaaagcugugggaaa.....	15	0	S01
...................................................................................uucaauaaagcugugggaaa.....	29	0	S05
...................................................................................uucaauaaagcugugggaaa.....	16	0	S06
...................................................................................uucaauaaagcugugggaaa.....	21	0	S04
...................................................................................uucaauaaagcugugggaaa.....	25	0	S02
...................................................................................uucaauaaagcugugggaaa.....	7	0	S03
...................................................................................uucaauaaagcugugggaaau....	6	0	S04
...................................................................................uucaauaaagcugugggaaau....	2	0	S02
...................................................................................uucaauaaagcugugggaaau....	3	0	S06
...................................................................................uucaauaaagcugugggaaau....	6	0	S05
...................................................................................uucaauaaagcugugggaaau....	1	0	S03
...................................................................................uucaauaaagcugugggaaauu...	2	0	S02
...................................................................................uucaauaaagcugugggaaauu...	1	0	S01
...................................................................................uucaauaaagcugugggaaauug..	1	0	S04
...................................................................................uucaauaaagcugugggaaauugc.	1	0	S06
....................................................................................ucaauaaagcugugggaa......	1	0	S06
....................................................................................ucaauaaagcugugggaa......	1	0	S02
....................................................................................ucaauaaagcugugggaaa.....	2	0	S01
....................................................................................ucaauaaagcugugggaaa.....	1	0	S05
....................................................................................ucaauaaagcugugggaaa.....	1	0	S06
....................................................................................ucaauaaagcugugggaaa.....	2	0	S04
....................................................................................ucaauaaagcugugggaaau....	1	0	S04
....................................................................................ucaauaaagcugugggaaau....	1	0	S05
....................................................................................ucaauaaagcugugggaaau....	1	0	S01
....................................................................................ucaauaaagcugugggaaau....	1	0	S06
.....................................................................................caauaaagcugugggaaa.....	1	0	S02
.....................................................................................caauaaagcugugggaaa.....	1	0	S01
......................................................................................aauaaagcugugggaaauug..	2	0	S04
......................................................................................aauaaagcugugggaaauug..	1	0	S05
......................................................................................aauaaagcugugggaaauug..	1	0	S06
......................................................................................aauaaagcugugggaaauug..	1	0	S03
......................................................................................aauaaagcugugggaaauugc.	35	0	S05
......................................................................................aauaaagcugugggaaauugc.	39	0	S04
......................................................................................aauaaagcugugggaaauugc.	9	0	S02
......................................................................................aauaaagcugugggaaauugc.	2	0	S03
......................................................................................aauaaagcugugggaaauugc.	11	0	S01
......................................................................................aauaaagcugugggaaauugc.	12	0	S06
.......................................................................................auaaagcugugggaaauugca	1	0	S04
........................................................................................uaaagcugugggaaauugca	1	0	S02
........................................................................................uaaagcugugggaaauugca	1	0	S01
```

Provisional ID	:	ta_iwgsc_2bl_v1_7971091_1403592
Score total	:	468.7
Score for star read(s)	:	3.9
Score for read counts	:	461
Score for mfe	:	2.2
Score for randfold	:	1.6
Score for cons. seed	:	
Total read count	:	916
Mature read count	:	854
Loop read count	:	23
Star read count	:	38

mdm-miR171i

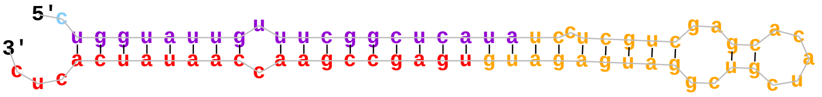

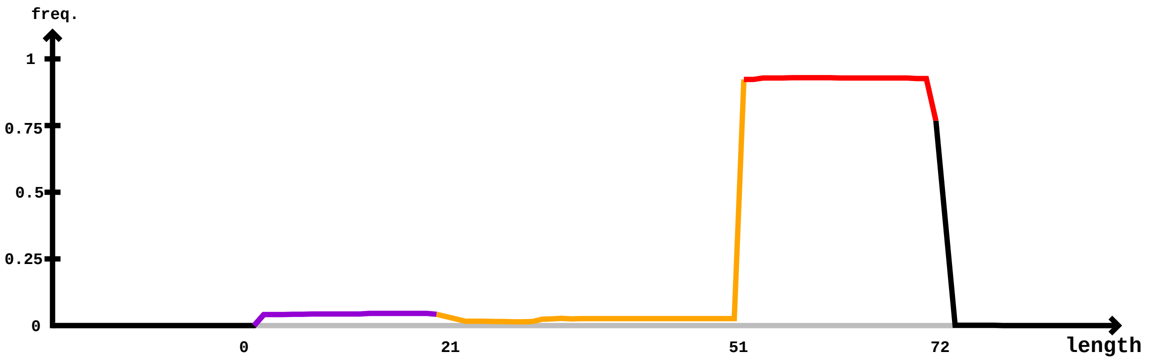


```
                           Star                                      Mature
5'- gagagaagaagaagacaugcugguauuguuucggcucauauccucgucgagcacaucgucggaugagaugugagccgaaccaauaucacucauguauucuuccucaga   -3'   obs
    gagagaagaagaagacaugcugguauuguuucggcucauauccucgucgagcacaucgucggaugagaugugagccgaaccaauaucacucauguauucuuccucaga         exp
    ....(((((((((.(((((.((((((((.(((((((((((((.(((((..((.....))..))))))))))))))))))).))))))))..))))).))))).))))...   reads   mm   sample
    ....................ugguauuguuucggcuca......................................................................   1   0   S02
    ....................ugguauuguuucggcuca......................................................................   2   0   S03
    ....................ugguauuguuucggcucau.....................................................................   1   0   S05
    ....................ugguauuguuucggcucau.....................................................................   2   0   S03
    ....................ugguauuguuucggcucau.....................................................................   1   0   S06
    ....................ugguauuguuucggcucau.....................................................................   2   0   S02
    ....................ugguauuguuucggcucaua....................................................................   6   0   S05
    ....................ugguauuguuucggcucaua....................................................................   7   0   S01
    ....................ugguauuguuucggcucaua....................................................................   4   0   S02
    ....................ugguauuguuucggcucaua....................................................................   8   0   S03
    ....................ugguauuguuucggcucaua....................................................................   1   0   S04
    ....................ugguauuguuucggcucaua....................................................................   3   0   S06
    .......................uauuguuucggcucauauccu................................................................   1   0   S04
    .........................uuguuucggcucauauccucg..............................................................   1   0   S03
    ...............................cggcucauauccucgucgagc........................................................   1   0   S03
    ...............................cggcucauauccucgucgagc........................................................   1   0   S06
    ........................................uccucgucgagcacaucgucggaugagaug......................................   2   0   S01
    ........................................uccucgucgagcacaucgucggaugagaug......................................   1   0   S05
    ........................................uccucgucgagcacaucgucggaugagaug......................................   2   0   S04
    ........................................uccucgucgagcacaucgucggaugagaug......................................   1   0   S06
    ........................................uccucgucgagcacaucgucggaugagaug......................................   4   0   S03
    ........................................uccucgucgagcacaucgucggaugagaug......................................   1   0   S02
    ................................................gagcacaucgucggaugagaug......................................   1   0   S04
    .................................................agcacaucgucggaugagaug......................................   1   0   S04
    .................................................agcacaucgucggaugagaug......................................   3   0   S03
    .................................................agcacaucgucggaugagaug......................................   2   0   S01
    .................................................agcacaucgucggaugagaug......................................   1   0   S06
    .................................................agcacaucgucggaugagaug......................................   1   0   S05
    ..................................................gcacaucgucggaugagaug......................................   1   0   S06
    ...................................................cacaucgucggaugagaug......................................   1   0   S06
    ...................................................cacaucgucggaugagaug......................................   1   0   S03
    .....................................................caucgucggaugagaugugagccgaac............................   1   0   S02
    ......................................................................ugagccgaaccaauauca....................   1   0   S06
```

Star Mature

```
gagagaagaagaagacaugcugguauuguuucggcucauauccucgucgagcacaucgucggaugagaugugagccgaaccaauaucacucauguauucuuccuucaga
......................................................................ugagccgaaccaauauca.....................   1     0   S03
......................................................................ugagccgaaccaauaucacu...................   43    0   S02
......................................................................ugagccgaaccaauaucacu...................   9     0   S05
......................................................................ugagccgaaccaauaucacu...................   3     0   S04
......................................................................ugagccgaaccaauaucacu...................   31    0   S03
......................................................................ugagccgaaccaauaucacC...................   1     1   S06
......................................................................ugagccgaaccaauaucacu...................   22    0   S01
......................................................................ugagccgaaccaauaucacu...................   35    0   S06
......................................................................ugagccgaaccaauaucacC...................   1     1   S05
......................................................................ugagccgaaccaauaucacC...................   1     1   S02
......................................................................ugagccgaaccaauaucacuc..................   4     0   S04
......................................................................ugagccgaaccaauaucacuc..................   137   0   S01
......................................................................ugagccgaaccaauaucacCc..................   1     1   S01
......................................................................ugagccgaaccaauaucacuc..................   130   0   S06
......................................................................ugagccgaaccaauaucacuc..................   76    0   S05
......................................................................ugagccgaaccaauaucacuc..................   161   0   S02
......................................................................ugagccgaaccaauaucacuc..................   192   0   S03
........................................................................agccgaaccaauaucacuc..................   1     0   S06
........................................................................agccgaaccaauaucacuc..................   1     0   S01
........................................................................agccgaaccaauaucacuc..................   1     0   S03
........................................................................agccgaaccaauaucacuca.................   2     0   S03
...........................................................................cgaaccaauaucacucauguau............   1     0   S06
```

Provisional ID	:	ta_iwgsc_6bl_v1_4395451_6016607
Score total	:	144.5
Score for star read(s)	:	3.9
Score for read counts	:	136.2
Score for mfe	:	2.8
Score for randfold	:	1.6
Score for cons. seed	:	
Total read count	:	279
Mature read count	:	3257
Loop read count	:	0
Star read count	:	2

mtr-miR164c

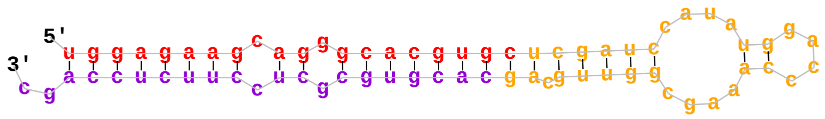

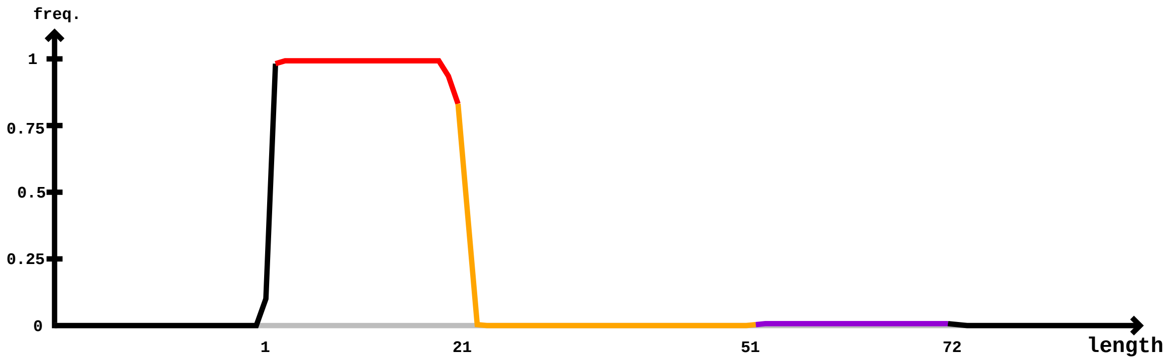

Mature Star

```
5'- gagggggcgagcgaacggugcuggagaagcagggcacgugcucgauccauauggacccaaagcgguugcagcacgugcgcuccuucuccagcauggcuucucgccccgucg  -3'  obs
    gagggggcgagcgaacggugcuggagaagcagggcacgugcucgauccauauggacccaaagcgguugcagcacgugcgcuccuucuccagcauggcuucucgccccgucg       exp
    ((.((((((((.((.(.((((((((((((.((.((((((((((((((....((....))....))))).))))))))).)).))))))))))))).).)))))))))).)).     reads      mm       sample
    ....................cuggagaagcagggcacgugc......................................................................     10         0        S05
    ....................cuggagaagcagggcacgugc......................................................................     4          0        S03
    ....................cuggagaagcagggcacgugc......................................................................     4          0        S06
    ....................cuggagaagcagggcacgugc......................................................................     2          0        S02
    ....................cuggagaagcagggcacgugc......................................................................     6          0        S04
    ....................cuggagaagcagggcacgugc......................................................................     2          0        S01
    .....................uggagaagcagggcacgu........................................................................     5          0        S03
    .....................uggagaagcagggcacgu........................................................................     1          0        S01
    .....................uggagaagcagggcacUu........................................................................     1          1        S05
    .....................uggagaagcagggcacgu........................................................................     1          0        S06
    .....................uggagaagcagggcacgu........................................................................     4          0        S04
    .....................uggagaagcagggcacgu........................................................................     4          0        S05
    .....................uggagaagcagggcacgug.......................................................................     2          0        S02
    .....................uggagaagcagggcacgug.......................................................................     3          0        S01
    .....................uggagaagcagggcacgug.......................................................................     8          0        S03
    .....................uggagaagcagggcacgug.......................................................................     4          0        S05
    .....................uggagaagcagggcacgug.......................................................................     3          0        S06
    .....................uggagaagcagggcacgug.......................................................................     9          0        S04
    .....................uggagaagcagggcacgugc......................................................................     19         0        S06
    .....................uggagaagcagggcacgugc......................................................................     55         0        S05
    .....................uggagaagcagggcacgugc......................................................................     11         0        S01
    .....................uggagaagcagggcacgugc......................................................................     11         0        S02
    .....................uggagaagcagggcacgugc......................................................................     74         0        S04
    .....................uggagaagcagggcacgugc......................................................................     12         0        S03
    .....................uggagaagcagggcacgugcu.....................................................................     1          0        S03
    .....................uggagaagcagggcacgugcu.....................................................................     4          0        S04
    .....................uggagaagcagggcacgugcu.....................................................................     3          0        S05
    .....................uggagaagcagggcacgugcu.....................................................................     1          0        S06
    .....................uggagaagcagggcacgugcu.....................................................................     4          0        S02
    .....................uggagaagcagggcacgugcu.....................................................................     5          0        S01
    .....................uggagaagcagggcacgugcuc....................................................................     1          0        S05
    ......................ggagaagcagggcacgugc......................................................................     1          0        S04
    ......................ggagaagcagggcacgugc......................................................................     2          0        S06
```

```
                     Mature                                              Star
gagggggcgagcgaacggugcuggagaagcagggcacgugcucgauccauauggacccaaagcgguugcagcacgugcgcuccuucuccagcauggcuucucgccccgucg
.......................................................................cacgugcgcuccuucuccagc...................    1    0    S03
........................................................................acgugcgcuccuucuccagc...................    1    0    S05
```

Provisional ID	:	ta_iwgsc_3as_v1_3426198_2535691
Score total	:	16264.2
Score for star read(s)	:	3.9
Score for read counts	:	16256.4
Score for mfe	:	2.2
Score for randfold	:	1.6
Score for cons. seed	:	
Total read count	:	31898
Mature read count	:	32093
Loop read count	:	0
Star read count	:	153

rco-miR156c

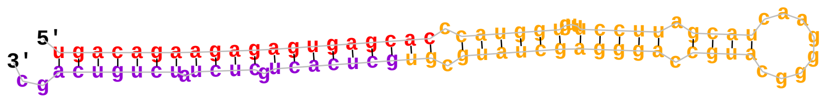

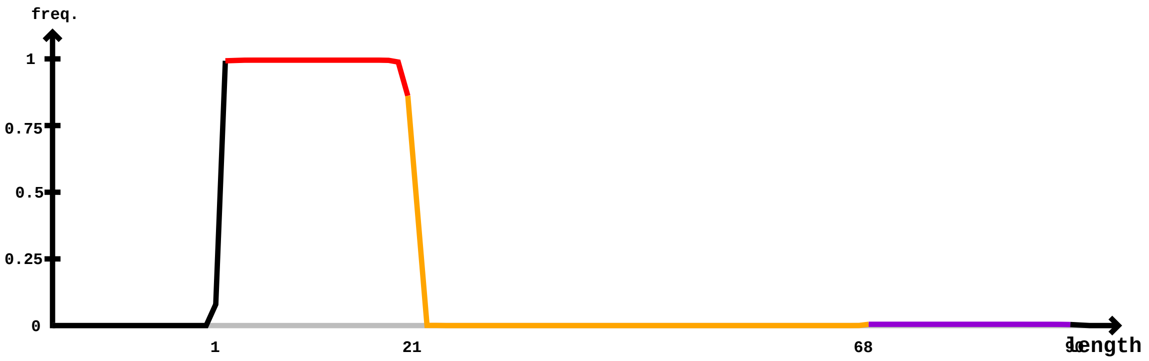

Mature **Star**

```
5'- ugcuguguguggaaguugacagaagagagugagcacccaugguguuuccuuagcaucaaggggcaugccagggagcuaugcgugcucacugcucuaucugucagccacu -3'  obs
    ugcuguguguggaaguugacagaagagagugagcacccaugguguuuccuuagcaucaaggggcaugccagggagcuaugcgugcucacugcucuaucugucagccacu       exp
    ........((((...(((((((((((((((((((((.((((((...(((((.((((........)))).))))))))))).)))))))))).)))).))))))))))))).   reads  mm  sample
    ...............uugacagaagagagugag............................................................................   4      0   S06
    ...............uugacagaagagagugag............................................................................   9      0   S03
    ...............uugacagaagagagugag............................................................................   4      0   S04
    ...............uugacagaagagagugag............................................................................   3      0   S05
    ...............uugacagaagagagugag............................................................................   3      0   S02
    ...............uugacagaagagagugagc...........................................................................   2      0   S04
    ...............uugacagaagagagugagc...........................................................................   1      0   S06
    ...............uugacagaagagagugagc...........................................................................   1      0   S01
    ...............uugacagaagagagugagc...........................................................................   1      0   S03
    ...............uugacagaagagagugagc...........................................................................   6      0   S05
    ...............uugacagaagagagugagc...........................................................................   4      0   S02
    ...............uugacagaagagagugagca..........................................................................   41     0   S06
    ...............uugacagaagagagugagca..........................................................................   62     0   S02
    ...............uugacagaagagagugagca..........................................................................   13     0   S01
    ...............uugacagaagagagugagca..........................................................................   33     0   S04
    ...............uugacagaagagagugagca..........................................................................   42     0   S05
    ...............uugacagaagagagugagca..........................................................................   88     0   S03
    ...............uugacagaagagagugagcac.........................................................................   212    0   S01
    ...............uugacagaagagagugagcac.........................................................................   512    0   S02
    ...............uugacagaagagagugagcac.........................................................................   535    0   S05
    ...............uugacagaagagagugagcac.........................................................................   323    0   S04
    ...............uugacagaagagagugagcac.........................................................................   379    0   S03
    ...............uugacagaagagagugagcac.........................................................................   246    0   S06
    ...............uugacagaagagagugagcacc........................................................................   3      0   S02
    ...............uugacagaagagagugagcacc........................................................................   3      0   S05
    ...............uugacagaagagagugagcacc........................................................................   2      0   S03
    ...............uugacagaagagagugagcacc........................................................................   1      0   S04
    ...............uugacagaagagagugagcaccc.......................................................................   1      0   S02
    ...............uugacagaagagagugagcaccca......................................................................   1      0   S01
    ................ugacagaagagagugagc...........................................................................   23     0   S02
    ................ugacagaagagagugagc...........................................................................   23     0   S01
    ................ugacagaagagagugagc...........................................................................   35     0   S04
    ................ugacagaagagagugagc...........................................................................   19     0   S03
```

```
                Mature                                                             Star
ugcuguguguggaaguugacagaagagagugagcacccaugguguuuccuuagcaucaaggggcaugccagggagcuaugcgugcucacugcucuaucugucagccacu
................ugacagaagagagugagc...........................................................................   57     0   S05
................ugacagaagagagugagc...........................................................................   29     0   S06
................ugacagaagagagugagca..........................................................................   589    0   S06
................ugacagaagagagugagca..........................................................................   786    0   S04
................ugacagaagagagugagca..........................................................................   933    0   S05
................ugacagaagagagugagca..........................................................................   291    0   S01
................ugacagaagagagugagca..........................................................................   644    0   S03
................ugacagaagagagugagca..........................................................................   505    0   S02
................ugacagaagagagugagcac.........................................................................   3114   0   S02
................ugacagaagagagugagcac.........................................................................   2357   0   S03
................ugacagaagagagugagcac.........................................................................   8503   0   S05
................ugacagaagagagugagcac.........................................................................   2273   0   S01
................ugacagaagagagugagcac.........................................................................   6183   0   S04
................ugacagaagagagugagcac.........................................................................   2614   0   S06
................ugacagaagagagugagcacc........................................................................   19     0   S06
................ugacagaagagagugagcacc........................................................................   14     0   S01
................ugacagaagagagAgagcacc........................................................................   1      1   S02
................ugacagaagagagugagcacc........................................................................   24     0   S03
................ugacagaagagagugagcacc........................................................................   40     0   S05
................ugacagaagagagugagcacc........................................................................   13     0   S02
................ugacagaagagagugagcacc........................................................................   34     0   S04
................ugacagaagagagugagcaccc.......................................................................   1      0   S04
................ugacagaagagagugagcaccca......................................................................   1      0   S02
................ugacagaagagagugagcaccca......................................................................   2      0   S04
................ugacagaagagagugagcaccca......................................................................   1      0   S06
................ugacagaagagagugagcaccca......................................................................   1      0   S03
................ugacagaagagagugagcaccca......................................................................   5      0   S05
.................gacagaagagagugagca..........................................................................   12     0   S03
.................gacagaagagagugagca..........................................................................   1      0   S05
.................gacagaagagagugagca..........................................................................   1      0   S06
.................gacagaagagagugagca..........................................................................   1      0   S01
.................gacagaagagagugagca..........................................................................   1      0   S02
.................gacagaagagagugagcac.........................................................................   3      0   S01
.................gacagaagagagugagcac.........................................................................   3      0   S04
.................gacagaagagagugagcac.........................................................................   7      0   S03
.................gacagaagagagugagcac.........................................................................   2      0   S02
.................gacagaagagagugagcac.........................................................................   1      0   S06
.................gacagaagagagugagcac.........................................................................   7      0   S05
..................acagaagagagugagcac.........................................................................   2      0   S04
..................acagaagagagugagcac.........................................................................   14     0   S03
..................acagaagagagugagcac.........................................................................   3      0   S05
..................acagaagagagugagcac.........................................................................   6      0   S06
..................acagaagagagugagcac.........................................................................   2      0   S01
..................acagaagagagugagcac.........................................................................   6      0   S02
..................acagaagagagugagcaccca......................................................................   2      0   S04
...................cagaagagagugagcaccca......................................................................   1      0   S02
....................agaagagagugagcaccca......................................................................   1      0   S03
..................................................................................ugcucacugcucuaucug.........   2      0   S03
..................................................................................ugcucacugcucuaucuguc.......   1      0   S06
..................................................................................ugcucacugcucuaucuguca......   1      0   S06
..................................................................................ugcucacugcucuaucuguca......   1      0   S01
...................................................................................gcucacugcucuaucuguc.......   1      0   S02
...................................................................................gcucacugcucuaucuguca......   1      0   S02
...................................................................................gcucacugcucuaucuguca......   1      0   S01
...................................................................................gcucacugcucuaucuguca......   1      0   S03
...................................................................................gcucacugcucuaucugucag.....   7      0   S04
...................................................................................gcucacugcucuaucugucag.....   2      0   S02
...................................................................................gcucacugcucuaucugucag.....   1      0   S01
...................................................................................gcucacugcucuaucugucag.....   7      0   S03
...................................................................................gcucacugcucuaucugucag.....   1      0   S06
...................................................................................gcucacugcucuaucugucag.....   2      0   S05
...................................................................................gcucacugcucuaucugucagc....   5      0   S02
...................................................................................gcucacugcucuaucugucagc....   9      0   S01
...................................................................................gcucacugcucuaucugucagc....   48     0   S04
...................................................................................gcucacugcucuaucugucagc....   36     0   S03
...................................................................................gcucacugcucuaucugucagc....   25     0   S05
...................................................................................gcucacugcucuaucugucagcc...   1      0   S03
```

Provisional ID	: ta_iwgsc_1bl_v1_3820936_373703	
Score total	:	68
Score for star read(s)	:	3.9
Score for read counts	:	60.3
Score for mfe	:	2.2
Score for randfold	:	1.6
Score for cons. seed	:	
Total read count	:	130
Mature read count	:	2553
Loop read count	:	0
Star read count	:	39

vvi-miR171e

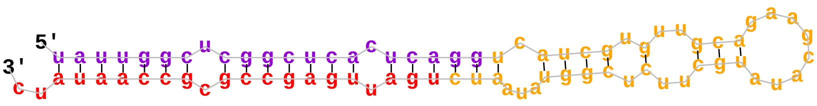

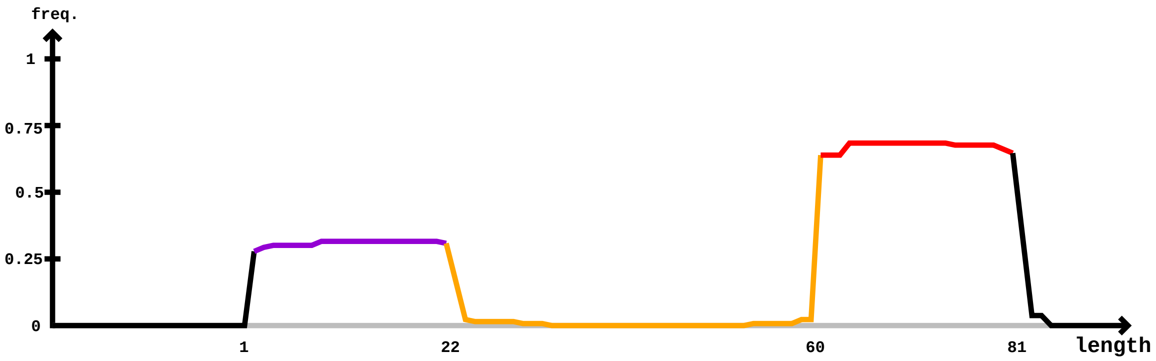

Star **Mature**

```
5'- aaugaaaucgcagcugcgguauuggcucggcucacucaggucaucguguugcagaagcauaugcuucucgguauaaaucugauugagccgcgccaauaucucagugccauu   -3'    obs
    aaugaaaucgcagcugcgguauuggcucggcucacucaggucaucguguugcagaagcauaugcuucucgguauaaaucugauugagccgcgccaauaucucagugccauu          exp
    .........(((.(((.(((((((((.(((((((.((((((.((((.(..(((........)))..).))))...)))))).))))))).))))))))).))))))....    reads    mm    sample
    ...................uauuggcucggcucacucag........................................................................    1        0     S05
    ...................uauuggcucggcucacucagg.......................................................................    3        0     S03
    ...................uauuggcucggcucacucagg.......................................................................    1        0     S02
    ...................uauuggcucggcucacucagg.......................................................................    16       0     S04
    ...................uauuggcucggcucacucagg.......................................................................    10       0     S05
    ...................uauuggcucggcucacucagg.......................................................................    6        0     S01
    ....................auuggcucggcucacucagg.......................................................................    1        0     S03
    ....................auuggcucggcucacucagg.......................................................................    1        0     S04
    .....................uuggcucggcucacucagguc.....................................................................    1        0     S03
    ..........................ucggcucacucaggucaucgu................................................................    1        0     S01
    ..........................ucggcucacucaggucaucguguu.............................................................    1        0     S01
    .......................................................................uauaaaucugauugagccgcgc..................    1        0     S02
    .............................................................................ucugauugagccgcgccaaua.............    1        0     S05
    .............................................................................ucugauugagccgcgccaaua.............    1        0     S04
    ...............................................................................ugauugagccgcgccaauau............    1        0     S03
    ...............................................................................ugauugagccgcgccaauau............    1        0     S02
    ...............................................................................ugauugagccgcgccaauauc...........    19       0     S01
    ...............................................................................ugauugagccgcgccaauauc...........    7        0     S02
    ...............................................................................ugauugagccgcgccaauauc...........    8        0     S03
    ...............................................................................ugauugagccgcgccaauauc...........    21       0     S04
    ...............................................................................ugauugagccgcgccaauauc...........    19       0     S06
    ...............................................................................ugauugagccgcgccaauauc...........    6        0     S05
    ..................................................................................uugagccgcgccaauauc...........    1        0     S06
    ..................................................................................uugagccgUgccaauaucuca........    1        1     S06
    ..................................................................................uugagccgUgccaauaucuca........    1        1     S03
    ..................................................................................uugagccgUgccaauaucuca........    3        1     S05
```

Provisional ID	:	ta_iwgsc_2bl_v1_8036763_1489078
Score total	:	253.4
Score for star read(s)	:	3.9
Score for read counts	:	245.4
Score for mfe	:	2.5
Score for randfold	:	1.6
Score for cons. seed	:	
Total read count	:	493
Mature read count	:	435
Loop read count	:	0
Star read count	:	48

gma-miR393j

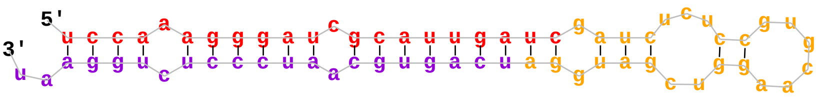

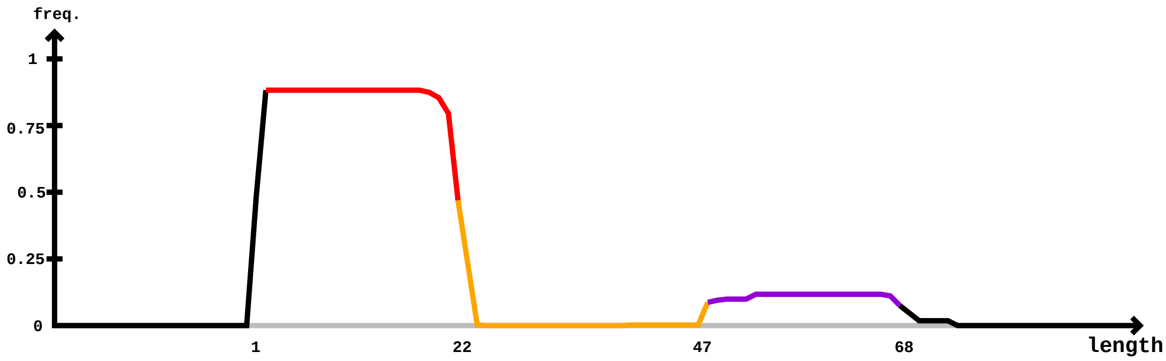

Mature **Star**

```
5'- ucaggaagcuaguggaggauuccaaagggaucgcauugaucgaucucuccgugcaaggucgauggaucagugcaaucccucuggaauucuccgcuugccuccgccuccguc   -3'   obs
    ucaggaagcuaguggaggauuccaaagggaucgcauugaucgaucucuccgugcaaggucgauggaucagugcaaucccucuggaauucuccgcuugccuccgccuccguc         exp
    ...(((.((.(((((((((((((.((((((.(((((((((.(((...((......))..))).))))))))).)))))).))))))))))))).)).)))...........   reads   mm   sample
    ...................uuccaaagggaucgcauu..........................................................................   1   0   S02
    ...................uuccaaagggaucgcauu..........................................................................   1   0   S01
    ...................uuccaaagggaucgcauu..........................................................................   1   0   S04
    ...................uuccaaagggaucgcauu..........................................................................   1   0   S05
    ...................uuccaaagggaucgcauug.........................................................................   2   0   S01
    ...................uuccaaagggaucgcauug.........................................................................   2   0   S02
    ...................uuccaaagggaucgcauug.........................................................................   1   0   S06
    ...................uuccaaagggaucgcauug.........................................................................   4   0   S04
    ...................uuccaaagggaucgcauuga........................................................................   6   0   S04
    ...................uuccaaagggaucgcauuga........................................................................   5   0   S01
    ...................uuccaaagggaucgcauuga........................................................................   6   0   S03
    ...................uuccaaagggaucgcauuga........................................................................   3   0   S02
    ...................uuccaaagggaucgcauuga........................................................................   9   0   S05
    ...................uuccaaagggaucgcauugau.......................................................................   4   0   S06
    ...................uuccaaagggaucgcauugau.......................................................................   24  0   S02
    ...................uuccaaagggaucgcauugau.......................................................................   34  0   S01
    ...................uuccaaagggaucgcauugau.......................................................................   12  0   S03
    ...................uuccaaagggaucgcauugau.......................................................................   47  0   S04
    ...................uuccaaagggaucgcauugau.......................................................................   37  0   S05
    ...................uuccaaagggaucgcauugauc......................................................................   10  0   S05
    ...................uuccaaagggaucgcauugauc......................................................................   3   0   S03
    ...................uuccaaagggaucgcauugauc......................................................................   17  0   S04
    ...................uuccaaagggaucgcauugauc......................................................................   3   0   S02
    ...................uuccaaagggaucgcauugauc......................................................................   6   0   S01
    ...................uuccaaagggaucgcauugaucga....................................................................   1   0   S04
    ....................uccaaagggaucgcauug.........................................................................   1   0   S03
    ....................uccaaagggaucgcauugau.......................................................................   1   0   S06
    ....................uccaaagggaucgcauugau.......................................................................   2   0   S04
    ....................uccaaagggaucgcauugauc......................................................................   32  0   S01
    ....................uccaaagggaucgcauugauc......................................................................   73  0   S04
    ....................uccaaagggaucgcauugauc......................................................................   22  0   S02
    ....................uccaaagggaucgcauugauc......................................................................   16  0   S03
    ....................uccaaagggaucgcauugauc......................................................................   7   0   S06
```

```
                    Mature                                           Star
ucaggaagcuaguggaggauuccaaagggaucgcauugaucgaucucuccgugcaaggucgauggaucagugcaaucccucuggaauucuccgcuugccuccgccuccguc
....................uccaaagggaucgcauugauc......................................................................    42    0    S05
..........................................................ucgauggaucagugcaaucccucuggaauu.......................    1     0    S04
..................................................................ucagugcaaucccucugga..........................    2     0    S02
..................................................................ucagugcaaucccucugga..........................    1     0    S05
..................................................................ucagugcaaucccucuggaa.........................    6     0    S04
..................................................................ucagugcaaucccucuggaa.........................    2     0    S03
..................................................................ucagugcaaucccucuggaa.........................    4     0    S01
..................................................................ucagugcaaucccucuggaa.........................    1     0    S06
..................................................................ucagugcaaucccucuggaa.........................    3     0    S02
..................................................................ucagugcaaucccucuggaa.........................    2     0    S05
..................................................................ucagugcaaucccucuggaau........................    5     0    S05
..................................................................ucagugcaaucccucuggaau........................    3     0    S04
..................................................................ucagugcaaucccucuggaau........................    1     0    S06
..................................................................ucagugcaaucccucuggaau........................    2     0    S02
..................................................................ucagugcaaucccucuggaau........................    3     0    S03
..................................................................ucagugcaaucccucuggaau........................    5     0    S01
..................................................................ucagugcaaucccucuggaauu.......................    2     0    S05
...................................................................cagugcaaucccucuggaau........................    1     0    S02
...................................................................cagugcaaucccucuggaauu.......................    1     0    S03
...................................................................cagugcaaucccucuggaauu.......................    2     0    S04
....................................................................agugcaaucccucuggaau........................    1     0    S01
....................................................................agugcaaucccucuggaauu.......................    1     0    S05
.......................................................................gcaaucccucuggaauucucc...................    4     0    S04
.......................................................................gcaaucccucuggaauucucc...................    3     0    S05
.......................................................................gcaaucccucuggaauucucc...................    2     0    S01
```
