# Supplementary material for: Identification and Comparative Analysis of microRNA in Wheat (Triticum aestivum L.) Callus Derived from Mature and Immature Embryos during In vitro Culture
Source: Front Plant Sci. 2016 Aug 30;7:1302. doi: 10.3389/fpls.2016.01302 (PMC5003897; doi:10.3389/fpls.2016.01302)
Supplement: Image S2 — Secondary structure of a set of novel miRNAs. [file Image2.pdf]

Provisional ID	:	ta_iwgsc_1al_v2_3909805_79647
Score total	:	1.3
Score for star read(s)	:	-1.3
Score for read counts	:	0
Score for mfe	:	1
Score for randfold	:	1.6
Score for cons. seed	:	
Total read count	:	84
Mature read count	:	92
Loop read count	:	0
Star read count	:	39

novel -m0412-5p

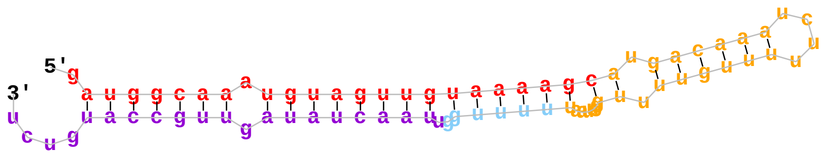

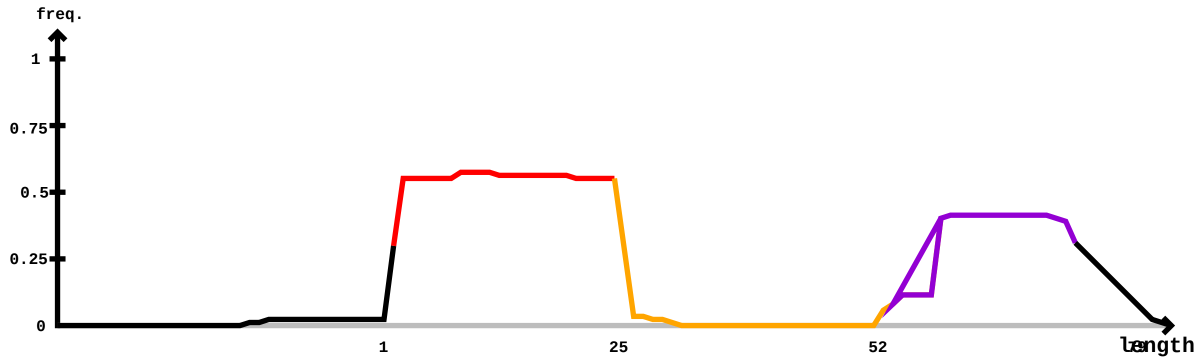

Mature **Star**

```
5'- aguuaccucaggaugguaacuuuaguuauuugagauggcaaauguaguuguaaaagcaugacaaaucuuuuuguuuugguuaauuuuugguuaacuauaguugccaugucuaau  -3'   obs
    aguuaccucaggaugguaacuuuaguuauuugagauggcaaauguaguuguaaaagcaugacaaaucuuuuuguuuugguuaauuuuugguuaacuauaguugccaugucuaau        exp
    ((((((.......)))))))..........((.(((((((.((((((((((((((((.((((((....)))))).)).....))))))..)))))))).))))))).))....   reads   mm   sample
    ..................acuuuaguuauuugagauggcaaa........................................................................   1       0    S04
    ....................uuuaguuauuugagauggcaaaug......................................................................   1       0    S01
    .................................gauggcaaauguaguuguaaaagc.........................................................   5       0    S05
    .................................gauggcaaauguaguuguaaaagc.........................................................   4       0    S02
    .................................gauggcaaauguaguuguaaaagc.........................................................   8       0    S04
    .................................gauggcaaauguaguuguaaaagc.........................................................   4       0    S06
    .................................gauggcaaauguaguuguaaaagc.........................................................   3       0    S01
    ..................................auggcaaauguaguugua..............................................................   1       0    S05
    ..................................auggcaaauguaguuguaaaagc.........................................................   4       0    S05
    ..................................auggcaaauguaguuguaaaUgc.........................................................   1       1    S05
    ..................................auggcaaauguaguuguaaaagc.........................................................   2       0    S02
    ..................................auggcaaauguaguuguaaaagc.........................................................   2       0    S06
    ..................................auggcaaauguaguuguaaaagc.........................................................   6       0    S04
    ..................................auggcaaauguaguuguaaaagc.........................................................   1       0    S03
    ..................................auggcaaauguaguuguaaaagca........................................................   5       0    S04
    ........................................aauguaguuguaaaagcaugac....................................................   1       0    S05
    ........................................aauguGguuguaaaagcaugaca...................................................   1       1    S04
    ...........................................uguaguuguaaaagcaug......................................................   1       0    S04
    ....................................................................................uuuugguuaacuauaguu............   1       0    S01
    ....................................................................................uuuugguuaacuauaguuU...........   1       1    S04
    ....................................................................................uuuugguuaacuauaguugc..........   1       0    S01
    ....................................................................................uuuugguuaacuauaguugc..........   1       0    S04
    ....................................................................................uuuugguuaacuauaguugcca........   1       0    S04
    .....................................................................................uuugguuaacuauaguugc..........   1       0    S05
    .....................................................................................uuugguuaacuauaguugc..........   1       0    S01
    ......................................................................................uugguuaacuauaguugc..........   1       0    S01
    ......................................................................................uugguuaacuauaguugc..........   2       0    S05
    ..........................................................................................uGaacuauaguugccauguc....   1       1    S05
    ..........................................................................................uuaacuauaguugccauguc....   2       0    S05
    ..........................................................................................uuaacuauaguugccauguc....   3       0    S01
    ..........................................................................................uuaacuauaguugccauguc....   1       0    S04
    ..........................................................................................uuaacuauaguugccauguc....   1       0    S02
    ..........................................................................................uuaacuauaguugccaugucu...   3       0    S01
```

```
                                 Mature                                            Star
aguuaccucaggaugguaacuuuaguuauuugagauggcaaauguaguuguaaaagcaugacaaaucuuuuuguuuuugguuaauuuuugguuaacuauaguugccaugucuaau
...........................................................................................uuaacuauaguugccaugucu...    2    0    S04
...........................................................................................uuaacuauaguugccaugucu...    5    0    S05
...........................................................................................uuaacuauaguugccaugucua..    3    0    S05
...........................................................................................uuaacuauaguugccaugucua..    1    0    S01
...........................................................................................uuaacuauaguugccaugucua..    1    0    S04
...........................................................................................uuaacuauaguugccaugucuaa.    1    0    S03
...........................................................................................uuaacuauaguugccaugucuaau    1    0    S02
............................................................................................uaacuauaguugccaugucu...    1    0    S03
```

Provisional ID	:	ta_iwgsc_1al_v2_3924239_100520
Score total	:	2.2
Score for star read(s)	:	-1.3
Score for read counts	:	0
Score for mfe	:	1.9
Score for randfold	:	1.6
Score for cons. seed	:	
Total read count	:	63
Mature read count	:	94
Loop read count	:	0
Star read count	:	64

novel-m0816-38

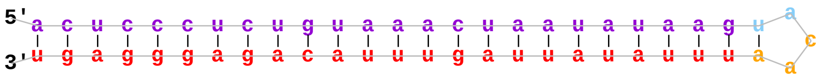

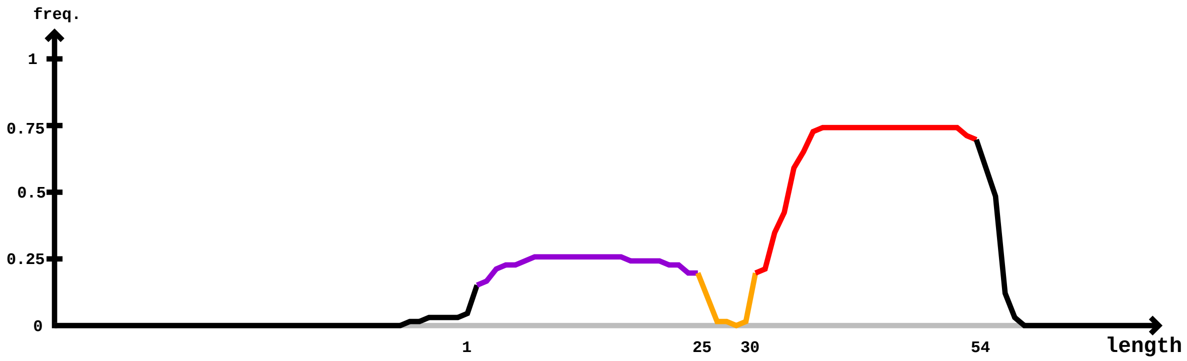

Star Mature

```
5'- cuuaggauauucccuaauuuuccuuuaaaaagguuacaaacuacucccucuguaaacuaauauaaguacaauuuauauuaguuuacagagggaguaccauagaaaccaguuaa   -3'    obs
    cuuaggauauucccuaauuuuccuuuaaaaagguuacaaacuacucccucuguaaacuaauauaaguacaauuuauauuaguuuacagagggaguaccauagaaaccaguuaa          exp
    .(((((......))))).............((((......((((((((((((((((((((((((((...))))))))))))))))))))))))))......)))).......     reads    mm    sample
    ...................................aAaaacuacucccucuguaaacu.......................................................     1        1     S06
    .....................................aaacuacucccucuguaaacuaaua...................................................     1        0     S04
    .........................................uacucccucuguaaacuaauauaagu..............................................     1        0     S05
    ..........................................acucAcucuguaaacuaauauaag...............................................     1        1     S02
    ..........................................acuGccucuguaaacuaauauaag...............................................     1        1     S05
    ..........................................acucccucuguaCacuaauauaag...............................................     1        1     S06
    ..........................................acucAcucuguaaacuaauauaag...............................................     2        1     S05
    ..........................................aGucccucuguaaacuaauauaag...............................................     1        1     S04
    ..........................................acucccucuguaaacuaauauaagu..............................................     1        0     S01
    ...........................................cucccucuguaaacuaauaGa.................................................     1        1     S05
    ............................................uGccucuguaaacuaauaua.................................................     1        1     S05
    ............................................uccUucuguaaacuaauauaagu..............................................     2        1     S05
    .............................................cccucuguaaacuaauauaagu..............................................     1        0     S05
    ...............................................Uucuguaaacuaauauaagu..............................................     1        1     S05
    ................................................ucuguaaacuaauauaaguUc............................................     1        1     S01
    ......................................................................auuuauauuaguuuacagaggga....................     1        0     S04
    .......................................................................uuuauauuaguuuacagagggag...................     1        0     S06
    .......................................................................uuuauauuaguuuacagagggagu..................     2        0     S05
    .......................................................................uuuauauuaguuuacagagggagu..................     1        0     S01
    .......................................................................uuuauauuaguuuacagagggagu..................     5        0     S04
    .......................................................................uuuauauuaguuuacagagggagu..................     1        0     S06
    .......................................................................uuuauauuaAuuuacagagggagu..................     1        1     S05
    .......................................................................uuuauauuaguuuacagagggagu..................     1        0     S02
    ........................................................................uuauauuaguuuacagagCga...................     1        1     S02
    .........................................................................uauauuaguuuacagagggaguac................     1        0     S01
    .........................................................................uauauuaguuuacagagggaguac................     3        0     S05
    .........................................................................uauauuaguuuacagagggaguac................     3        0     S04
    .........................................................................uauauuaguuuacagagggaguac................     1        0     S03
    .........................................................................uauauuaguuuacagagggaguacc...............     1        0     S02
    ..........................................................................auauuaguuuacagagggagG..................     1        1     S01
    ..........................................................................auauuaguuuacagagggaguac................     1        0     S06
    ..........................................................................auauuaguuuacagagggaguac................     1        0     S05
    ..........................................................................auauuaguuuacagagggaguac................     1        0     S03
```

Star Mature

```
cuuaggauauucccuaauuuuccuuuaaaaagguuacaaacuacucccucuguaaacuaauauaaguacaauuuauauuaguuuacagagggaguaccauagaaaccaguuaa
..........................................................................auauuaguuuacagagggaguac................   1   0   S04
...........................................................................uauuaguuuacagagCgagua.................   1   1   S05
...........................................................................uauuaguuuacagagggagGa.................   1   1   S02
...........................................................................uauuaguuuacagagggaguac................   4   0   S05
...........................................................................uauuaguuuacagagggaguac................   1   0   S06
...........................................................................uauuaguuuacagagggaguac................   1   0   S04
...........................................................................uauuaguuuacagagggaguac................   1   0   S02
...........................................................................uauuaguuuacagagCgaguac................   1   1   S05
...........................................................................uauuUguuuacagagggaguacca..............   1   1   S05
............................................................................auuaguuuacagagggaguac................   1   0   S01
............................................................................auuaguuuacagagggaguac................   2   0   S04
............................................................................auuaguuuacagagggaguacca..............   1   0   S03
.............................................................................uuaguuuacagagggaguacc...............   1   0   S02
.............................................................................uuaguuuacagagggaguacc...............   3   0   S01
.............................................................................uuaguuuacagagggaguacc...............   1   0   S05
..............................................................................uaguuuacagagggaguac................   1   0   S04
```

Provisional ID	: ta_iwgsc_1as_v1_2484491_217314	novel-m0234-5p
Score total	:	2.8
Score for star read(s)	:	-1.3
Score for read counts	:	0
Score for mfe	:	2.5
Score for randfold	:	1.6
Score for cons. seed	:	
Total read count	:	150
Mature read count	:	128
Loop read count	:	0
Star read count	:	72

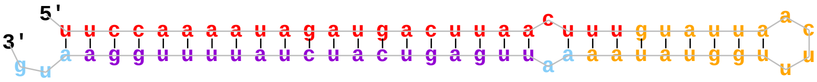

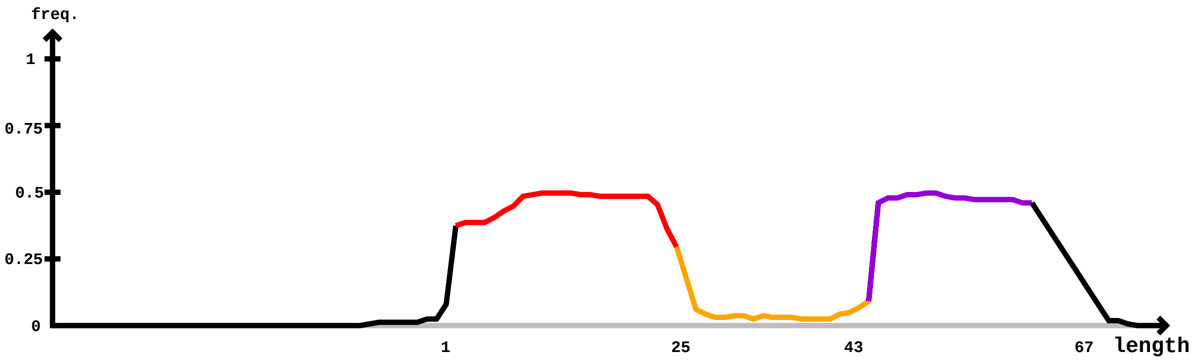

Mature **Star**

```
5'- gcugguuccgccgccagugaauaaaaaucuacucccuucauuccaaaauagaugacuuaacuuuguauuaacuuugguauaaaauugagucaucuauuuuggaauggagggagu  -3'   obs
    gcugguuccgccgccagugaauaaaaaucuacucccuucauuccaaaauagaugacuuaacuuuguauuaacuuugguauaaaauugagucaucuauuuuggaauggagggagu        exp
    ((((((......))))))............((((((((((((((((((((((((((((((.(((((((((....))))))))).))))))))))))))))))))))))))))))   reads   mm   sample
    ...............................cuAccuucauuccaaaauagauga...........................................................   1       1    S02
    ................................uUccuucauuccaaaauagau.............................................................   1       1    S04
    .....................................ucauuccaaaauagaugacuuaac.....................................................   1       0    S04
    .....................................ucauuccaaaauagaugacuuaac.....................................................   1       0    S05
    .......................................auuccaaaauagaugacuuaacuu...................................................   1       0    S05
    .......................................auuccaaaauagaugacuuaacuu...................................................   2       0    S01
    .......................................auuccaaaauagaugacuuaacuu...................................................   2       0    S04
    .......................................auuccaaaauagaugacuuaacuu...................................................   3       0    S02
    .......................................auuccaaaauagaugacuuaacuu...................................................   1       0    S03
    ........................................uuccaaaauagaugacuu........................................................   1       0    S01
    ........................................uuccaaaauagaugacuuaac.....................................................   1       0    S02
    ........................................uuccaaaauagaugacuuaac.....................................................   1       0    S01
    ........................................uuccaaaauagaugacuuaac.....................................................   1       0    S04
    ........................................uuccaaaauagaugacuuaacu....................................................   4       0    S05
    ........................................uuccaaaauagaugacuuaacu....................................................   2       0    S02
    ........................................uuccaaaauagaugacuuaacu....................................................   3       0    S01
    ........................................uuccaaaauagaugacuuaacu....................................................   5       0    S04
    ........................................uuccaaaauagaugacuuaacuu...................................................   1       0    S04
    ........................................uuccaaaauagaugacuuaacuu...................................................   1       0    S05
    ........................................uuccaaaauagaugacuuaacuuu..................................................   4       0    S02
    ........................................uuccaaaauagaugacuuaacuuu..................................................   8       0    S04
    ........................................uuccaaaauagaugacuuaacuuu..................................................   8       0    S05
    ........................................uuccaaaauagaugacuuaacuuu..................................................   4       0    S01
    ........................................uuccaaaauagaugacuuaacuuu..................................................   3       0    S06
    ........................................uuccaaaauagaugacuuaacuuu..................................................   1       0    S03
    .........................................uccaaaauagaugacuuaacu....................................................   1       0    S03
    .........................................uccaaaauagaugacuuaacuuu..................................................   1       0    S01
    ............................................aaaauagaugacuuaacuuu..................................................   2       0    S06
    ............................................aaaauagaugacuuaacuuu..................................................   1       0    S05
    .............................................aaauagaugacuuaacuuu..................................................   1       0    S04
    .............................................aaauagaugacuuaacuuu..................................................   2       0    S06
    .............................................aaauagaugacuuaacuuug.................................................   1       0    S06
    ..............................................aauagaugacuuaacuuu..................................................   1       0    S04
```

```
Mature Star
gcugguuccgccgccagugaauaaaaaucuacucccuucauuccaaaauagaugacuuaacuuuguauuaacuuugguauaaaauugagucaucuauuuuggaauggagggagu
..............................................aauagaugacuuaacuuugu................................................ 1 0 S04
..............................................aauagaugacuuaacuuugua............................................... 1 0 S03
...............................................auagaugacuuaacuuug................................................. 1 0 S04
...............................................auagaugacuuaacuuugu................................................ 1 0 S05
...............................................auagaugacuuaacuuugu................................................ 1 0 S04
...............................................auagaugacuuaacuuuguauua............................................ 1 0 S02
...............................................auagaugacuuaacuuuguauuaa........................................... 2 0 S01
................................................uagaugacuuaacuuugua............................................... 1 0 S04
.................................................agaugacuuaacuuuguauuaacu......................................... 1 0 S05
..........................................................aacuuuguauuaacuuug...................................... 1 0 S06
.....................................................................aacuuugguauaaaauugaguc....................... 1 0 S03
......................................................................acuuugguauaaaauugagucauc.................... 1 0 S04
........................................................................uuugguauaaaauugaguc....................... 1 0 S02
........................................................................uuugguauaaaauugaguca...................... 1 0 S05
................................................................................aaaauugagucaucuauuu............... 1 0 S03
................................................................................aaaauugagucaucuauuu............... 1 0 S04
................................................................................aaaauugagucaucuauuuugga........... 1 0 S01
.................................................................................aaauugagucaucuauuuugg............ 1 0 S03
..................................................................................aauugagucaucuauuuugg............ 1 0 S06
..................................................................................aauugagucaucuauuuugga........... 1 0 S06
..................................................................................aauugagucaucuauuuugga........... 1 0 S05
...................................................................................auugagucaucuauuuugga........... 1 0 S04
...................................................................................auugagucaucuauuuugga........... 2 0 S06
...................................................................................auugagucaucuauuuuggaaugg....... 1 0 S06
....................................................................................uugagucaucuauuuugg............ 1 0 S06
....................................................................................uugagucaucuauuuugg............ 1 0 S02
....................................................................................uugagucaucuauuuugga........... 5 0 S05
....................................................................................uugagucaucuauuuugga........... 4 0 S03
....................................................................................uugagucaucuauuuugga........... 6 0 S06
....................................................................................uugagucaucuauuuugga........... 8 0 S01
....................................................................................uugagucaucuauuuugga........... 2 0 S04
....................................................................................uugagucaucuauuuuggaa.......... 5 0 S04
....................................................................................uugagucaucuauuuuggaa.......... 3 0 S03
....................................................................................uugagucaucuauuuuggaa.......... 4 0 S01
....................................................................................uugagucaucuauuuuggaa.......... 1 0 S05
....................................................................................uugagucaucuauuuuggaa.......... 2 0 S06
....................................................................................uugagucaucuauuuuggaU.......... 1 1 S01
....................................................................................uugagucaucuauuuuggaa.......... 8 0 S02
....................................................................................uugagucaucuauuuuggaaugga...... 1 0 S05
....................................................................................uugagucaucuauuuuggaaugga...... 1 0 S01
....................................................................................uugagucaucuauuuuggaaugga...... 5 0 S04
....................................................................................uugagucaucuauuuuggaaugga...... 2 0 S06
.....................................................................................ugagucaucuauuuugga........... 1 0 S05
.....................................................................................ugagucaucuauuuugga........... 1 0 S04
.....................................................................................ugagucaucuauuuugga........... 1 0 S01
.......................................................................................agucaucuauuuuggaauggagg.... 1 0 S01
.......................................................................................agucaucuauuuuggaauggaggg... 1 0 S04
.........................................................................................ucaucuauuuuggaauggagg.... 1 0 S04
```

Provisional ID	:	ta_iwgsc_1as_v1_3271665_252067
Score total	:	3.1
Score for star read(s)	:	-1.3
Score for read counts	:	0
Score for mfe	:	2.8
Score for randfold	:	1.6
Score for cons. seed	:	
Total read count	:	46
Mature read count	:	54
Loop read count	:	0
Star read count	:	35

novel-m0144-3p

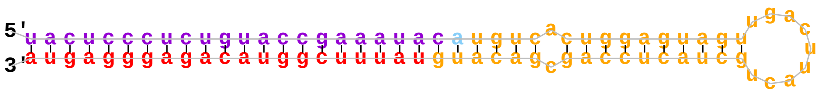

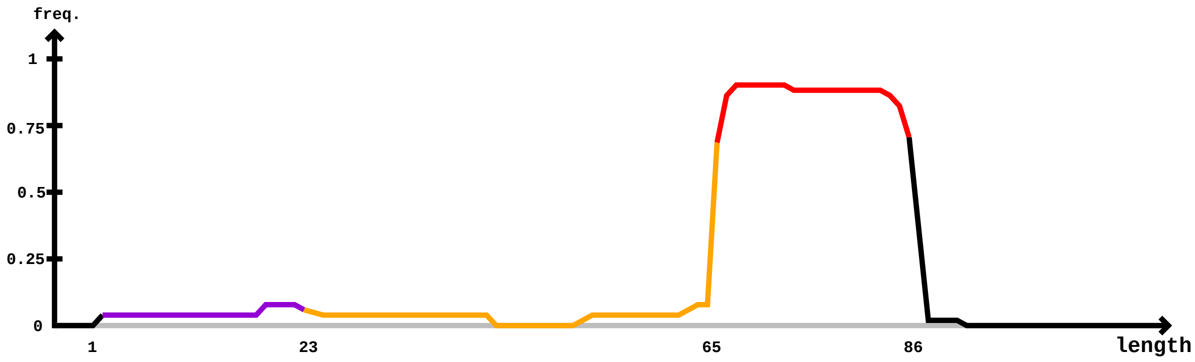

Star **Mature**

```
5'- guauacucccucuguaccgaaauacaugucacuggaguaguugacuuacugcuacuccagcgacauguauuucgguacagagggaguacauccguauuauacuguggauuuuuu  -3'   obs
    guauacucccucuguaccgaaauacaugucacuggaguaguugacuuacugcuacuccagcgacauguauuucgguacagagggaguacauccguauuauacuguggauuuuuu         exp
    ...(((((((((((((((((((((((((((.((((((((((.........)))))))))).))))))))))))))))))))))))))).(((((((......))))))).....    reads    mm    sample
    ...uacucccucuguaccgaaaua..........................................................................................    1        0     S04
    ...uacucccucuguaccgaaauac.........................................................................................    1        0     S01
    ....................aauacaugucacuggaguaguuga......................................................................    2        0     S04
    .....................................................acuccagcgacauguauuucg........................................    1        0     S05
    ......................................................cuccagcgacauguauuucgg.......................................    1        0     S01
    ................................................................auguauuucgguacagaggga.............................    1        0     S05
    .................................................................uguauuucgguaUagagggag............................    1        1     S04
    ...................................................................uauuucgguacagagggag............................    1        0     S02
    ...................................................................uauuucgguacagagggagu...........................    1        0     S02
    ...................................................................uauuucgguacagagggagu...........................    1        0     S05
    ...................................................................uauuucgguacagagggagu...........................    1        0     S03
    ...................................................................uauuucgguacagagggagu...........................    1        0     S06
    ...................................................................uauuucgguacagagggagua..........................    5        0     S05
    ...................................................................uauuucgguacagagggagua..........................    6        0     S02
    ...................................................................uauuucgguacagagggagua..........................    5        0     S01
    ...................................................................uauuucgguacagagggagua..........................    3        0     S03
    ...................................................................uauuucgguacagagggagua..........................    3        0     S06
    ...................................................................uauuucgguacagagggagua..........................    4        0     S04
    ....................................................................auuucgguacagagggagu...........................    2        0     S05
    ....................................................................auuucgguacagagggagua..........................    1        0     S06
    ....................................................................auuucgguacagagggagua..........................    2        0     S05
    ....................................................................auuucgguacagagggagua..........................    1        0     S03
    ....................................................................auuucgguacagagggaguac.........................    2        0     S01
    ....................................................................auuucgguacagagggaguac.........................    1        0     S02
    .....................................................................uuucgguacagagggagua..........................    2        0     S06
    ..........................................................................Uuacagagggaguacaucc.....................    1        1     S06
```

Provisional ID	:	ta_iwgsc_1bl_v1_3872318_439837
Score total	:	206.5
Score for star read(s)	:	3.9
Score for read counts	:	199
Score for mfe	:	2.1
Score for randfold	:	1.6
Score for cons. seed	:	
Total read count	:	402
Mature read count	:	450
Loop read count	:	0
Star read count	:	5

novel-m0244-5p

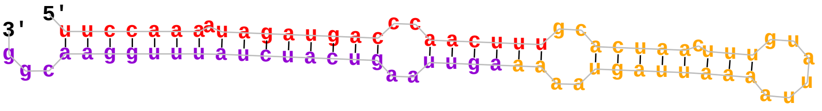

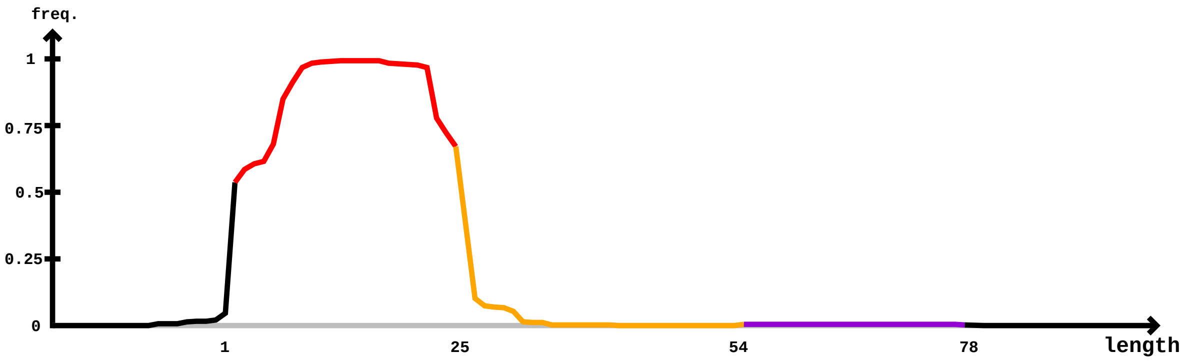

Mature Star

```
5'- aucaucuacucccuccauuccaaaauagaugacccaacuuugcacuaacuuuguauuaaaauuaguaaaaaguuaagucaucuauuuggaacggagggagggaguaguuaaua  -3'   obs
    aucaucuacucccuccauuccaaaauagaugacccaacuuugcacuaacuuuguauuaaaauuaguaaaaaguuaagucaucuauuuggaacggagggagggaguaguuaaua         exp
    .....(((((((((((.(((((((.((((((((..((((((..(((((.(((......))))))))..))))))..))))))))))))))).....)))))))))))......   reads   mm   sample
    .........uUccuccauuccaaaauagaugac................................................................................   1   1   S05
    .........ucccuccauuccaaaauagaugac................................................................................   2   0   S04
    ............cuccauuccaaaauagaugac................................................................................   1   0   S04
    ............cuccauuccaaaauagaugaccc..............................................................................   2   0   S04
    .............uccauuccaaaauagaugacc...............................................................................   1   0   S04
    ...............cauuccaaaauagaugacccaacu..........................................................................   2   0   S06
    ................auuccaaaauagaugacccaac...........................................................................   2   0   S01
    ................auuccaaaauagaugacccaacu..........................................................................   1   0   S03
    ................auuccaaaauagaugacccaacuu.........................................................................   1   0   S02
    ................auuccaaaauagaugacccaacuu.........................................................................   2   0   S05
    ................auuccaaaauagaugacccaacuu.........................................................................   1   0   S04
    ................auuccaaaauagaugacccaacuu.........................................................................   3   0   S06
    ................auuccaaaauagaugacccaacuuu........................................................................   1   0   S05
    .................uuccaaaauagaugaccca.............................................................................   1   0   S04
    .................uuccaaaauagaugacccaa............................................................................   3   0   S03
    .................uuccaaaauagaugacccaa............................................................................   1   0   S01
    .................uuccaaaauagaugacccaac...........................................................................   12  0   S03
    .................uuccaaaauagaugacccaac...........................................................................   16  0   S06
    .................uuccaaaauagaugacccaac...........................................................................   6   0   S01
    .................uuccaaaauagaugacccaac...........................................................................   12  0   S02
    .................uuccaaaauagaugacccaCc...........................................................................   1   1   S06
    .................uuccaaaauagaugacccaac...........................................................................   11  0   S04
    .................uuccaaaauagaugacccaac...........................................................................   20  0   S05
    .................uuccaaaauagaugacccaacu..........................................................................   2   0   S01
    .................uuccaaaauagaugacccaacu..........................................................................   1   0   S03
    .................uuccaaaauagaugacccaacuu.........................................................................   1   0   S04
    .................uuccaaaauagaugacccaacuu.........................................................................   1   0   S06
    .................uuccaaaauagaugacccaacuu.........................................................................   1   0   S01
    .................uuccaaaauagaugacccaacuu.........................................................................   1   0   S02
    .................uuccaaaauagaugacccaacuu.........................................................................   1   0   S05
    .................uuccaaaauagaugacccaacuuu........................................................................   14  0   S02
    .................uuccaaaauagaugacccaacuuu........................................................................   3   0   S03
    .................uuccaaaauagaugacccaacuuu........................................................................   39  0   S04
```

Mature / Star			
aucaucuacucccucca**uuccaaaauagaugacccaacuuu**gcacuaacuuuguauuaaaauuaguaaaa**aguuaagucaucuauuuggaacgg**agggagggaguaguuaaua			
.................uuccaaaauagaugacccaacuuu..	15	0	S06
.................uuccaaaauagaugacccaacuuu..	16	0	S01
.................uuccaaaauagaugacccaacuuu..	34	0	S05
..................uccaaaauagaugacccaac...	1	0	S06
..................uccaaaauagaugacccaac...	1	0	S03
..................uccaaaauagaugacccaacu..	3	0	S02
..................uccaaaauagaugacccaacu..	3	0	S03
..................uccaaaauagaugacccaacu..	2	0	S05
..................uccaaaauagaugacccaacu..	1	0	S01
..................uccaaaauagaugacccaacu..	3	0	S04
..................uccaaaauagaugacccaacu..	3	0	S06
..................uccaaaauagaugacccaacuu...	1	0	S03
..................uccaaaauagaugacccaacuuu..	1	0	S05
..................uccaaaauagaugacccaacuuu..	1	0	S02
..................uccaaaauagaugacccaacuuug...	1	0	S01
...................ccaaaauagaugacccaacu..	1	0	S05
...................ccaaaauagaugacccaacuu...	1	0	S03
...................ccaaaauagaugacccaacuu...	1	0	S06
...................ccaaaauagaugacccaacuu...	2	0	S01
...................ccaaaauagaugacccaacuuu..	1	0	S02
...................ccaaaauagaugacccaacuuu..	1	0	S04
...................ccaaaauagaugacccaacuuugc..	2	0	S06
....................caaaauagaugacccaacuuu..	1	0	S05
....................caaaauagaugacccaacuuu..	1	0	S06
....................caaaauagaugacccaacuuu..	1	0	S01
....................caaaauagaugacccaacuuug...	1	0	S06
.....................aaaauagaugacccaacu..	1	0	S01
.....................aaaauagaugacccaacu..	1	0	S06
.....................aaaauagaugacccaacuuu..	5	0	S06
.....................aaaauagaugacccaacuuu..	3	0	S03
.....................aaaauagaugacccaacuuu..	2	0	S01
.....................aaaauagaugacccaacuuu..	3	0	S05
.....................aaaauagaugacccaacuuug...	3	0	S06
.....................aaaauagaugacccaacuuug...	1	0	S02
.....................aaaauagaugacccaacuuug...	1	0	S03
.....................aaaauagaugacccaacuuug...	2	0	S01
.....................aaaauagaugacccaacuuug...	1	0	S04
.....................aaaauagaugacccaacuuug...	4	0	S05
.....................aaaauagaugacccaacuuugcac..	1	0	S01
......................aaauagaugacccaacuu...	2	0	S06
......................aaauagaugacccaacuu...	1	0	S04
......................aaauagaugacccaacuu...	1	0	S05
......................aaauagaugacccaacuu...	1	0	S02
......................aaauagaugacccaacuuu..	1	0	S04
......................aaauagaugacccaacuuu..	2	0	S03
......................aaauagaugacccaacuuu..	1	0	S02
......................aaauagaugacccaacuuug...	8	0	S01
......................aaauagaugacccaacuuug...	3	0	S05
......................aaauagaugacccaacuuug...	9	0	S04
......................aaauagaugacccaacuuug...	13	0	S06
......................aaauagaugacccaacuuug...	17	0	S03
......................aaauagaugacccaacuuug...	9	0	S02
......................aaauagaugacccaacuuugc..	3	0	S02
......................aaauagaugacccaacuuugc..	1	0	S01
......................aaauagaugacccaacuuugcacu...	1	0	S05
.......................aauagaugacccaacuuu..	1	0	S01
.......................aauagaugacccaacuuu..	2	0	S05
.......................aauagaugacccaacuuu..	1	0	S06
.......................aauagaugacccaacuuu..	1	0	S02
.......................aauagaugacccaacuuug...	7	0	S03
.......................aauagaugacccaacuuug...	1	0	S04
.......................aauagaugacccaacuuug...	2	0	S05
.......................aauagaugacccaacuuug...	2	0	S01
.......................aauagaugacccaacuuug...	2	0	S02
.......................aauagaugacccaacuuug...	1	0	S06
.......................aauagaugacccaacuuugc..	1	0	S06
.......................aauagaugacccaacuuugc..	1	0	S05
.......................aauagaugacccaacuuugcacua..	1	0	S02
.......................aauagaugacccaacuuugcacua..	1	0	S04
.......................aauagaugacccaacuuugcacua..	3	0	S05

```
                 Mature                                                 Star
aucaucuacucccuccauuccaaaauagaugacccaacuuugcacuaacuuuguauuaaaauuaguaaaaaguuaagucaucuauuuggaacggagggagggaguaguuaaua
........................auagaugacccaacuuug.......................................................................   3   0   S05
........................auagaugacccaacuuug.......................................................................   1   0   S06
........................auagaugacccaacuuug.......................................................................   2   0   S01
........................auagaugacccaacuuug.......................................................................   1   0   S03
........................auagaugacccaacuuugc......................................................................   1   0   S05
........................auagaugacccaacuuugG......................................................................   1   1   S05
........................auagaugacccaacuuugc......................................................................   2   0   S02
........................auagaugacccaacuuugca.....................................................................   1   0   S06
........................auagaugacccaacuuugGa.....................................................................   1   1   S04
........................auagaugacccaacuuugcacua..................................................................   5   0   S04
........................auagaugacccaacuuugcacua..................................................................   2   0   S05
........................auagaugacccaacuuugcacua..................................................................   1   0   S01
........................auagaugacccaacuuugcacua..................................................................   2   0   S06
........................auagaugacccaacuuugcacuaa.................................................................   1   0   S05
.........................uagaugacccaacuuugcacu...................................................................   1   0   S03
.........................uagaugacccaacuuugcacu...................................................................   1   0   S05
.........................uagaugacccaacuuugcacu...................................................................   2   0   S02
.........................uagaugacccaacuuugcacua..................................................................   1   0   S01
.........................uagaugacccaacuuugcacua..................................................................   1   0   S02
.........................uagaugacccaacuuugcacuaacu...............................................................   1   0   S03
..........................agaugacccaacuuugcacu...................................................................   1   0   S05
..........................agaugacccaacuuugcacuaacu...............................................................   1   0   S05
...........................gaugacccaacuuugcacuaacu...............................................................   1   0   S04
............................augacccaacuuuAcacuaacu...............................................................   1   1   S05
...................................aacuuugcacuaacuuuguaCu........................................................   1   1   S01
......................................................................aguuaagucaucuauuuggaacg....................   1   0   S05
......................................................................aguuaagucaucuauuuggaacgg...................   1   0   S06
```

Provisional ID : ta_iwgsc_3b_v1_10578297_2743600

novel-m0649-5p

Score total : 852.5
Score for star read(s) : 3.9
Score for read counts : 844.4
Score for mfe : 2.6
Score for randfold : 1.6
Score for cons. seed :
Total read count : 1668
Mature read count : 1451
Loop read count : 16
Star read count : 231

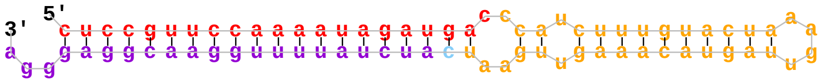

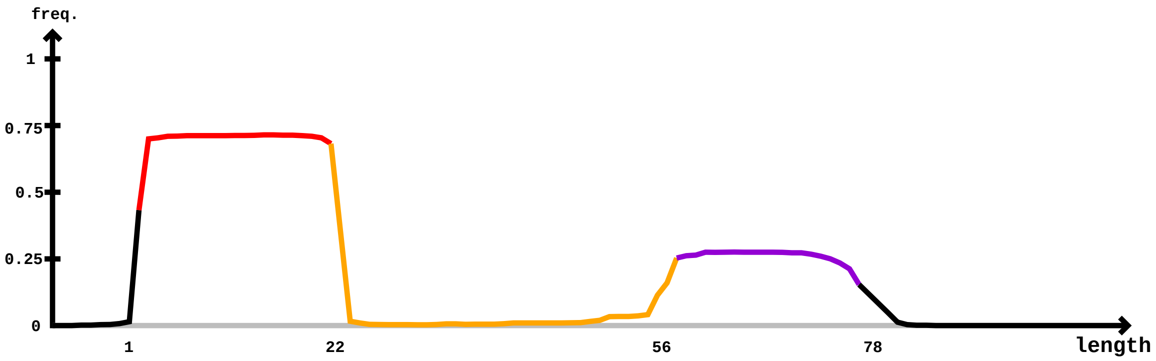

Mature   Star

5'- guacucccuccguuccaaaauagaugacccaucuuuguacuaaaguuaguacaaaguugaaucaucuauuuuggaacggagggaguaaguacggguacggcccugaaauc -3' obs

guacucccuccguuccaaaauagaugacccaucuuuguacuaaaguuaguacaaaguugaaucaucuauuuuggaacggaggg aguaaguacggguacggcccugaaauc exp

.((((((((((((((((((((((((((..((.((((((((((....)))))))))).))..))))))))))))))))))))))))))....((((......)))).....	reads	mm	sample
.uacucccuccguuccaaaaua...	1	0	S02
.uacucccuccguuccaaaaua...	1	0	S05
.uacucccuccguuccaaaauaga...	1	0	S02
...cucccuccguuccaaaauagauga..	1	0	S01
...cucccuccguuccaaaauagaugacc..	1	0	S01
...cucccuccguuccaaaauagaugacc..	1	0	S03
....ucccuccguuccaaaauagaugac...	1	0	S05
.....cccuccguuccaaaauagau..	1	0	S02
.....cccuccguuccaaaauagaug...	1	0	S01
.....cccuccguuccaaaauagauga..	1	0	S02
.....cccuccguuccaaaauagaugac...	1	0	S01
.....cccuccguuccaaaauagaugacc..	1	0	S05
.....cccuccguuccaaaauagaugaccc...	1	0	S03
......ccuccguuccaaaauaga...	1	0	S01
......ccuccguuccaaaauaga...	1	0	S04
......ccuccguuccaaaauagauga..	2	0	S05
......ccuccguuccaaaauagauga..	1	0	S03
......ccuccguuccaaaauagauga..	1	0	S02
......ccuccguuccaaaauagauga..	2	0	S04
......ccuccguuccaaaauagaugacc..	1	0	S04
......ccuccguuccaaaauagaugaccc...	3	0	S04
.......cuccguuccaaaauagau..	2	0	S02
.......cuccguuccaaaauagau..	1	0	S03
.......cuccguuccaaaauagaug...	2	0	S06
.......cuccguuccaaaauagaug...	1	0	S04
.......cuccguuccaaaauagaug...	2	0	S03
.......cuccguuccaaaauagauga..	4	0	S06
.......cuccguuccaaaauagauga..	9	0	S03
.......cuccguuccaaaauagauga..	5	0	S05
.......cuccguuccaaaauagauga..	4	0	S02
.......cuccguuccaaaauagauga..	4	0	S04
.......cuccguuccaaaauagauga..	3	0	S01
.......cuccguuccaaaauagaugac...	96	0	S03

```
       Mature                                                 Star
guacucccuccguuccaaaauagaugacccaucuuuguacuaaaguuaguacaaaguugaaucaucuauuuuggaacggagggaguaaguacggguacggcccugaaauc
.......cuccguuccaaaauagaugac..................................................................................    69    0    S06
.......cuccguuccaaaauagaugac..................................................................................    101   0    S04
.......cuccguuccaaaauagaugac..................................................................................    192   0    S05
.......cuccguuccaaaauagaugac..................................................................................    127   0    S01
.......cuccguuccaaaauagaugac..................................................................................    92    0    S02
.......cuccguuccaaaauagaugacc.................................................................................    4     0    S05
.......cuccguuccaUaauagaugacc.................................................................................    1     1    S05
.......cuccguuccaaaauagaugacc.................................................................................    1     0    S03
.......cuccguuccaaaauagaugacc.................................................................................    1     0    S04
.......cuccguuccaaaauagaugacc.................................................................................    2     0    S02
.......cuccguuccaaaauagaugacc.................................................................................    1     0    S06
........uccguuccaaaauagaug....................................................................................    1     0    S04
........uccguuccaaaauagaug....................................................................................    2     0    S03
........uccguuccaaaauagaug....................................................................................    1     0    S01
........uccguuccaaaauagauga...................................................................................    1     0    S04
........uccguuccaaaauagaugac..................................................................................    2     0    S01
........uccguuccaaaauagaugac..................................................................................    1     0    S04
........uccguuccaaaauagaugac..................................................................................    4     0    S06
........uccguuccaaaauagaugac..................................................................................    1     0    S02
........uccguuccaaaauagaugac..................................................................................    6     0    S03
........uccguuccaaaauagaugac..................................................................................    9     0    S05
........uccguuccaaaUuagaugacc.................................................................................    1     1    S03
........uccguuccaaaauagaugacc.................................................................................    70    0    S01
........uccguuccaaaauagaugacc.................................................................................    67    0    S03
........uccguuccaaaUuagaugacc.................................................................................    1     1    S01
........uccguuccaUaauagaugacc.................................................................................    1     1    S02
........uccguuccaaaauagaugacc.................................................................................    54    0    S02
........uccguuccaaaauagaugacc.................................................................................    56    0    S04
........uccguuccaUaauagaugacc.................................................................................    4     1    S05
........uccguuccaaaauagaugacc.................................................................................    61    0    S06
........uccguuccaaaauagaugacc.................................................................................    110   0    S05
........uccguuccaUaauagaugacc.................................................................................    1     1    S04
........uccguuccaUaauagaugacc.................................................................................    3     1    S03
........uccguuccaaaauagaugaccc................................................................................    1     0    S04
........uccguuccaaaauagaugaccc................................................................................    1     0    S01
........uccguuccaaaauagaugaccc................................................................................    1     0    S05
........uccguuccaaaauagaugaccca...............................................................................    1     0    S04
........uccguuccaaaauagaugacccUu..............................................................................    1     1    S05
.........ccguuccaaaauagaugac..................................................................................    1     0    S03
.........ccguuccaaaauagaugacc.................................................................................    2     0    S02
.........ccguuccaaaauagaugacc.................................................................................    2     0    S05
.........ccguuccaaaauagaugaccc................................................................................    1     0    S04
.........ccguuccaaaauagaugaccc................................................................................    1     0    S05
..........cguuccaaaauagaugac..................................................................................    1     0    S03
..........cguuccaaaauagaugacc.................................................................................    1     0    S03
..........cguuccaaaauagaugaccc................................................................................    1     0    S01
..........cguuccaaaauagaugaccca...............................................................................    1     0    S06
..........cguuccaaaauagaugaccca...............................................................................    1     0    S02
..........cguuccaaaauagaugaccca...............................................................................    3     0    S05
..........cguuccaaaauagaugaccca...............................................................................    2     0    S01
...........guuccaaaauagaugaccc................................................................................    1     0    S04
............uuccaaaauagaugaccca...............................................................................    1     0    S04
............uuccaaaauagaugacccaCc.............................................................................    1     1    S06
............uuccaaaauagaugacUcaucuuu..........................................................................    1     1    S04
.................aaauagaugacccaucuuuguacu.....................................................................    1     0    S05
...................auagaugacccaucuuuguacu.....................................................................    1     0    S03
....................uagaugacccaucuuuguac......................................................................    1     0    S02
....................uagaugacccaucuuuguacu.....................................................................    1     0    S06
....................uagaugacccaucuuuguacu.....................................................................    1     0    S05
......................................acuaaagCuaguacaaaguugaa.................................................    1     1    S05
......................................aUuaaaguuaguacaaaguugaau................................................    1     1    S03
.......................................Uuaaaguuaguacaaaguugaau................................................    2     1    S04
.......................................Uuaaaguuaguacaaaguugaauc...............................................    1     1    S04
.......................................cuaaagCuaguacaaaguugaauc...............................................    1     1    S05
........................................uaaaguuaguacaaaguugaauc...............................................    1     0    S04
.........................................aaaguuaguacaaaguugaau................................................    1     0    S04
..........................................aaguuaguacaaaguugaaucauc............................................    1     0    S04
.............................................uuaguacaaaguugaaucauUu...........................................    3     1    S01
..............................................uaguacaaaguugaaucauc............................................    1     0    S02
..............................................uaguacaaaguugaaucaucu...........................................    1     0    S01
```

Mature **Star**

guacucccuccguuccaaaauagaugacccaucuuuguacuaaaguuaguacaaaguugaaucaucuauuuuggaacggagggaguaaguacggguacggcccugaaauc			
....uaguacaaaguugaaucauUu...	1	1	S03
....uaguacaaaguugaaucaucu...	1	0	S05
....uaguacaaaguugaaucaucuauu..	1	0	S02
....aaaguugaaucaucuauuuugga...................................	1	0	S03
....aaguugaaucaucuauuuugg....................................	1	0	S01
....aguugaaucaucuauuuuggaacg................................	2	0	S05
....aguugaaucaucuauuuuggaacg................................	1	0	S03
....aguugaaucaucuauuuuggaacg................................	1	0	S02
....aguugaaucaucuauuuuggaacg................................	2	0	S06
....aguugaaucaucuauuuuggaacg................................	1	0	S04
....aAuugaaucaucuauuuuggaacg................................	1	1	S05
....guugaaucaucuauuuuggaacg................................	2	0	S06
....Auugaaucaucuauuuuggaacg................................	2	1	S04
....guugaUucaucuauuuuggaacgg...............................	1	1	S02
....Auugaaucaucuauuuuggaacgg...............................	1	1	S04
....guugaaucaucuauuuuggaacgg...............................	1	0	S02
....uugaaucaucuauuuugga...................................	1	0	S05
....uugaaucaucuauuuugga...................................	1	0	S04
....uugaaucaucuauuuuggaac.................................	1	0	S01
....uugaaucaucuauuuuggaac.................................	2	0	S06
....uugaaucaucuauuuuggaac.................................	3	0	S05
....uugaaucaucuauuuuggaac.................................	3	0	S03
....uugaaucaucuauuuuggaacg................................	1	0	S06
....uugaaucaucuauuuuggaacgA...............................	1	1	S05
....uugaaucaucuauuuuggaacgA...............................	1	1	S04
....uugaaucaucuauuuuggaacgg...............................	1	0	S05
....uugaaucaucuauuuuggaacgg...............................	1	0	S04
....uugaaucaucuauuuuggaacgga..............................	2	0	S05
....uugaaucaucuauuuuggaacgga..............................	5	0	S04
....uugaaucaucuauuuuggaacgga..............................	1	0	S02
....ugaaucaucuauuuuggaacggag.............................	1	0	S06
....aaucaucuauuuuggaacgg...............................	1	0	S02
....aaucaucuauuuuggaacgga..............................	1	0	S04
....aaucaucuauuuuggaacggagg............................	1	0	S02
....aaucaucuauuuuggaacggaggg...........................	1	0	S05
....aucaucuauuuuggaacgg...............................	1	0	S01
....aucaucuauuuuggaacggagg............................	1	0	S06
....aucaucuauuuuggaacggagg............................	1	0	S04
....aucaucuauuuuggaacggaggga..........................	3	0	S06
....aucaucuauuuuggaacggaggga..........................	2	0	S04
....ucaucuauuuuggaacgg...............................	3	0	S06
....ucaucuauuuuggaacgg...............................	1	0	S03
....ucaucuauuuuggaacgg...............................	1	0	S01
....ucaucuauuuuggaacgg...............................	2	0	S05
....ucaucuauuuuggaacgg...............................	1	0	S02
....ucaucuauuuuggaacgga..............................	2	0	S02
....ucaucuauuuuggaacgga..............................	1	0	S06
....ucaucuauuuuggaacgga..............................	2	0	S03
....ucaucuauuuuggaacgga..............................	1	0	S04
....ucaucuauuuuggaacgga..............................	5	0	S01
....ucaucuauuuuggaacggag.............................	2	0	S05
....ucaucuauuuuggaacggag.............................	3	0	S04
....ucaucuauuuuggaacggag.............................	9	0	S02
....ucaucuauuuuggaacggag.............................	3	0	S06
....ucaucuauuuuggaacggag.............................	2	0	S01
....ucaucuauuuuggaacggag.............................	8	0	S03
....ucaucuauuuuggaacggagg............................	19	0	S05
....ucaucuauuuuggaacggagg............................	15	0	S02
....ucaucuauuuuggaacggagg............................	14	0	S04
....ucaucuauuuuggaacggagg............................	6	0	S06
....ucaucuauuuuggaacggagg............................	17	0	S01
....ucaucuauuuuggaacggagg............................	5	0	S03
....ucaucuauuuuggaacggaggga..........................	1	0	S04
....ucaucuauuuuggaacggagggag.........................	1	0	S01
....ucaucuauuuuggaacggagggagu........................	1	0	S02
....ucaucuauuuuggaacggagggagu........................	1	0	S04
....ucaucuauuuuggaacggagggagu........................	1	0	S06
....caucuauuuuggaacgga..............................	1	0	S03
....caucuauuuuggaacgga..............................	1	0	S04
....caucuauuuuggaacgga..............................	3	0	S06

Mature **Star**

```
guacucccuccguuccaaaauagaugacccaucuuuguacuaaaguuaguacaaaguugaaucaucuauuuuggaacggagggaguaaguacggguacggcccugaaauc
..............................................................caucuauuuuggaacgga..............................  1   0   S01
..............................................................caucuauuuuggaacgga..............................  1   0   S05
..............................................................caucuauuuuggaacggag.............................  1   0   S03
..............................................................caucuauuuuggaacggag.............................  1   0   S06
..............................................................caucuauuuuggaacggag.............................  1   0   S05
..............................................................caucuauuuuggaacggagg............................  1   0   S03
..............................................................caucuauuuuggaacggagg............................  10  0   S06
..............................................................caucuauuuuggaacggagg............................  2   0   S02
..............................................................caucuauuuuggaacggagg............................  3   0   S01
..............................................................caucuauuuuggaacggaggg...........................  3   0   S01
..............................................................caucuauuuuggaacggaggg...........................  3   0   S05
..............................................................caucuauuuuggaacggaggg...........................  1   0   S06
..............................................................caucuauuuuggaacggaggg...........................  2   0   S02
..............................................................caucuauuuuggaacggaggg...........................  5   0   S04
..............................................................caucuauuuuggaacggaggg...........................  2   0   S03
..............................................................caucuauuuuggaacggaggga..........................  1   0   S03
..............................................................caucuauuuuggaacggagggag.........................  1   0   S03
..............................................................caucuauuuuggaacggagggag.........................  1   0   S06
..............................................................caucuauuuuggaacggagggagu........................  4   0   S02
..............................................................caucuauuuuggaacggagggagu........................  5   0   S05
..............................................................caucuauuuuggaacggagggagu........................  3   0   S01
..............................................................caucuauuuuggaacggagggagu........................  17  0   S04
..............................................................caucuauuuuggaacggagggagu........................  2   0   S03
..............................................................caucuauuuuggaacggagggagu........................  8   0   S06
...............................................................aucuauuuuggaacggag.............................  3   0   S05
...............................................................aucuauuuuggaacggag.............................  1   0   S04
...............................................................aucuauuuuggaacggag.............................  3   0   S06
...............................................................aucuauuuuggaacggagg............................  2   0   S02
...............................................................aucuauuuuggaacggagg............................  2   0   S04
...............................................................aucuauuuuggaacggagg............................  2   0   S06
...............................................................aucuauuuuggaacggaggg...........................  5   0   S02
...............................................................aucuauuuuggaacggaggg...........................  4   0   S06
...............................................................aucuauuuuggaacggaggg...........................  2   0   S04
...............................................................aucuauuuuggaacggaggg...........................  3   0   S05
...............................................................aucuauuuuggaacggaggg...........................  2   0   S01
...............................................................aucuauuuuggaacggaggg...........................  5   0   S03
...............................................................aucuauuuuggaacggaggga..........................  26  0   S02
...............................................................aucuauuuuggaacggaggga..........................  20  0   S06
...............................................................aucuauuuuggaacggaggga..........................  38  0   S05
...............................................................aucuauuuuggaacggaggga..........................  9   0   S01
...............................................................aucuauuuuggaacggaggga..........................  5   0   S03
...............................................................aucuauuuuggaacggaggga..........................  25  0   S04
...............................................................aucuauuuuggaacggagggag.........................  1   0   S04
...............................................................aucuauuuuggaacggagggagu........................  1   0   S04
...............................................................aucuauuuuggaacggagggagu........................  1   0   S06
...............................................................aucuauuuuggaacggagggagu........................  1   0   S05
...............................................................aucuauuuuggaacggagggagu........................  1   0   S03
...............................................................aucuauuuuggaacggagggagua.......................  1   0   S04
...............................................................aucuauuuuggaacggagggagua.......................  1   0   S05
................................................................ucuauuuuggaacggagg............................  1   0   S04
................................................................ucuauuuuggaacggaggg...........................  1   0   S04
................................................................ucuauuuuggaacggaggga..........................  1   0   S02
................................................................ucuauuuuggaacggaggga..........................  1   0   S04
................................................................ucuauuuuggaacggagggag.........................  1   0   S03
................................................................ucuauuuuggaacggagggag.........................  1   0   S02
................................................................ucuauuuuggaacggagggag.........................  2   0   S05
................................................................ucuauuuuggaacggagggag.........................  3   0   S04
................................................................ucuauuuuggaacggagggagu........................  2   0   S02
................................................................ucuauuuuggaacggagggagu........................  1   0   S04
................................................................ucuauuuuggaacggagggagu........................  1   0   S05
.................................................................cuauuuuggaacggagggagu........................  3   0   S01
.................................................................cuauuuuggaacggagggagua.......................  1   0   S01
..................................................................uauuuuggaacggagggagu........................  1   0   S06
..................................................................uauuuuggaacggagggagu........................  4   0   S04
..................................................................uauuuuggaacggagggagu........................  3   0   S05
..................................................................uauuuuggaacggagggagu........................  1   0   S02
..................................................................uauuuuggaacggagggagua.......................  1   0   S01
..................................................................uauuuuggaacggagggagua.......................  1   0   S03
..................................................................uauuuuggaacggagggagua.......................  4   0   S02
..................................................................uauuuuggaacggagggagua.......................  1   0   S06
```

```
       Mature                                                 Star
guacucccuccguuccaaaauagaugacccaucuuuguacuaaaguuaguacaaaguugaaucaucuauuuuggaacggagggaguaaguacggguacggcccugaaauc
..................................................................uauuuuggaacggagggagua.......................	3	0	S04
..................................................................uauuuuggaacggagggagua.......................	1	0	S05
..................................................................uauuuuggaacggagggaguaa......................	1	0	S05
...................................................................auuuuggaacggagggagua.......................	1	0	S03
...................................................................auuuuggaacggagggagua.......................	1	0	S01
...................................................................auuuuggaacggagggaguaa......................	2	0	S06
...................................................................auuuuggaacggagggaguaa......................	1	0	S02
....................................................................uuuAggaacggagggaguaagu....................	1	1	S04
......................................................................uuggaacggagggaguaagu....................	1	0	S04
```

Provisional ID : ta_iwgsc_1dl_v1_1338648_600510
Score total : 2.6
Score for star read(s) : -1.3
Score for read counts : 0
Score for mfe : 2.3
Score for randfold : 1.6
Score for cons. seed :
Total read count : 279
Mature read count : 355
Loop read count : 0
Star read count : 78

novel-m0598-5p

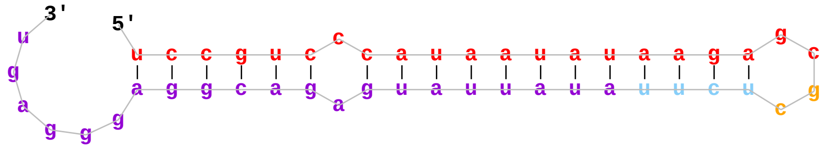

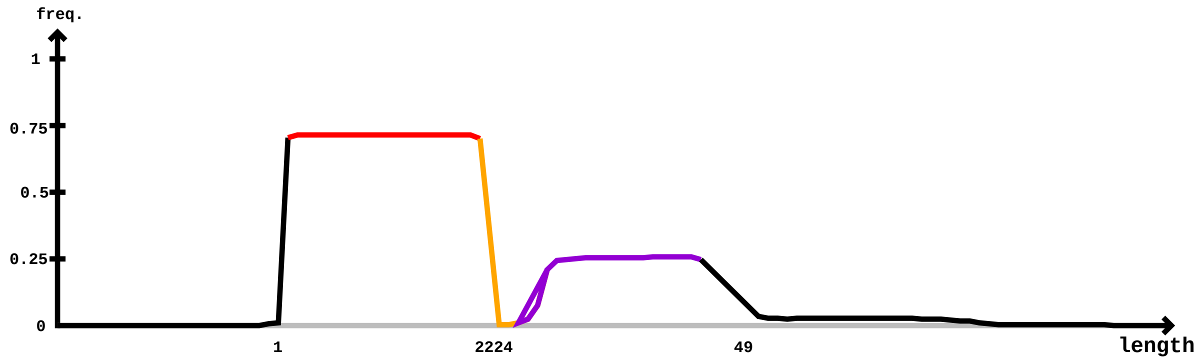

Mature **Star**

```
5'- caguaagaaaaguaguacuccuuccgucccauaauauaagagcgcucuuauauuaugagacggagggaguaguacucagugaugguguaggcgccugguaacacccgcacgaga   -3'    obs
    caguaagaaaaguaguacuccuuccgucccauaauauaagagcgcucuuauauuaugagacggagggaguacucagugaugguguaggcgccugguaacacccgcacgaga          exp
    .........((((.(((((((((((((.((((((((((((....)))))))))))).))))))))))))).))))..(((..(((((..((.....)).)))))..)))....   reads   mm   sample
    ....................cuuccgucccauaauauaagagc.......................................................................   1   0   S04
    ....................cuuccgucccauaauauaagagc.......................................................................   1   0   S05
    .....................uuccgucccauaauauaagagcgc.....................................................................   1   0   S06
    ......................uccgucccauaauauaagag........................................................................   2   0   S06
    ......................uccgucccauaauauaagag........................................................................   1   0   S03
    ......................uccgucccauaauauaagag........................................................................   1   0   S02
    ......................uccgucccauaauauaagagc.......................................................................   71   0   S05
    ......................uccgucccauaauauaagCgc.......................................................................   1   1   S04
    ......................uccgucccauaauauaagagc.......................................................................   24   0   S06
    ......................uccgucccauaauauaagagc.......................................................................   33   0   S02
    ......................uccgucccauaauauaagagc.......................................................................   19   0   S03
    ......................uccgucccauaauauaagagc.......................................................................   23   0   S01
    ......................uccgucccauaauauaagagc.......................................................................   25   0   S04
    ......................uccgucccauaGuauaagagc.......................................................................   1   1   S05
    ......................uccgucccauaauauaagagcg......................................................................   1   0   S01
    .......................ccgucccauaauauaagagc.......................................................................   1   0   S05
    .......................ccgucccauaauauaagagc.......................................................................   1   0   S02
    .......................ccgucccauaauauaagagcg......................................................................   1   0   S05
    .............................................ucuuauauuaugagacggag.................................................   1   0   S05
    ..............................................cuuauauuaugagacggag.................................................   1   0   S03
    ..............................................cuuauauuaugagacggagggagu............................................   1   0   S05
    ...............................................uuauauuaugagacggag.................................................   1   0   S02
    ...............................................uuauCuuaugagacggaggga..............................................   1   1   S05
    ...............................................uuauauuaugagacggaggga..............................................   1   0   S02
    ...............................................uuauauuaugagacggagggagu............................................   1   0   S05
    ................................................uauauuaugagacggagg................................................   1   0   S04
    ................................................uauauuaugagacggagggag.............................................   1   0   S04
    ................................................uauauuaugagacggagggag.............................................   1   0   S01
    ................................................uauauuaugagacggagggagu............................................   3   0   S05
    ................................................uauauuaugagacggagggagu............................................   1   0   S01
    ................................................uauauuaugagacggagggagu............................................   7   0   S02
    ................................................uauauuaugagacggagggagu............................................   1   0   S04
    .................................................auauuaugagacggagggag.............................................   1   0   S02
```

Mature **Star**

```
caguaagaaaaguaguacuccuuccgucccauaauauaagagcgcucuuauauuaugagacggagggaguaguacucagugaugguguaggcgccugguaacacccgcacgaga
.................................................auauuaugagacggagggag.............................................	1	0	S06
.................................................auauuaugagacggagggagu............................................	1	0	S03
.................................................auauuaugagacggagggagu............................................	3	0	S01
.................................................auauuaugagacggagggagu............................................	4	0	S04
.................................................auauuaugagacggagggagu............................................	10	0	S02
.................................................auauuaugagacggagggagu............................................	6	0	S06
.................................................auauuaugagacggagggagu............................................	3	0	S05
.................................................auauuaugagacggagggagua...........................................	1	0	S04
.................................................auauuaugagacggagggagua...........................................	1	0	S01
.................................................auauuaugagacggagggagua...........................................	1	0	S05
.................................................auauuaugagacggagggagua...........................................	1	0	S03
.................................................auauuaugagacggagggagua...........................................	5	0	S02
.................................................auauuaugagacggagggaguag..........................................	1	0	S05
..................................................uauuaugagacggaggga..............................................	2	0	S06
..................................................uauuaugagacggagggag.............................................	1	0	S03
..................................................uauuaugagacggagggagua...........................................	3	0	S02
..................................................uauuaugagacggagggagua...........................................	1	0	S01
..................................................uauuaugagacggagggagua...........................................	2	0	S04
..................................................uauuaugagacggagggaguag..........................................	1	0	S06
...................................................auuaugagacggagggagua...........................................	1	0	S02
....................................................uuaugagacggagggaguagua........................................	1	0	S04
.....................................................uaugagacggagggagua...........................................	1	0	S04
............................................................cggagggaguaguacucagugaug..............................	1	0	S03
...................................................................aguaguacucagugauggugu..........................	1	0	S05
...................................................................aguaguacucagugaugguguagg.......................	1	0	S05
....................................................................guaguacucagugaugguguaggc......................	1	0	S05
......................................................................aguacucagugaugguguaggcgc....................	1	0	S04
......................................................................aguacucagugaugguguaggcgc....................	1	0	S06
.......................................................................guacucagugaugguguaggcgcc...................	1	0	S03
...........................................................................ucagugaugguguaggcgccu..................	1	0	S01
....................................................................................guguaggcgccugguaacacccgc......	1	0	S06
```

Provisional ID	:	ta_iwgsc_1dl_v1_2233756_651100
Score total	:	290.9
Score for star read(s)	:	3.9
Score for read counts	:	283.6
Score for mfe	:	1.8
Score for randfold	:	1.6
Score for cons. seed	:	
Total read count	:	568
Mature read count	:	702
Loop read count	:	0
Star read count	:	98

novel-m0186-5p

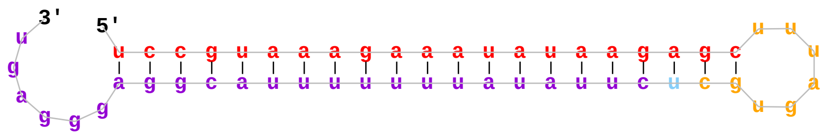

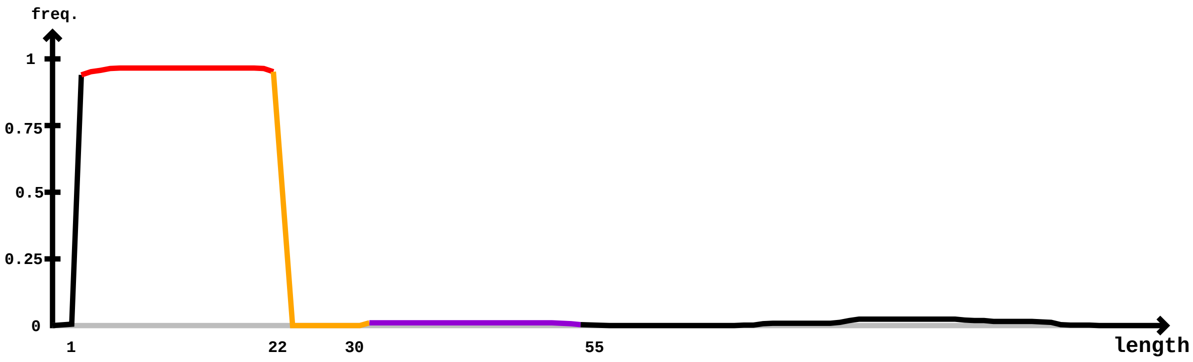

Mature **Star**

```
5'- cuccguaaagaaauauaagagcuuuagugcucuuauauuuuuuuacggagggaguacuaaaguuguaccaaaugugcgucaauuaauuuggaucggagggaguaguagaagugu  -3'   obs
    cuccguaaagaaauauaagagcuuuagugcucuuauauuuuuuuacggagggaguacuaaaguuguaccaaaugugcgucaauuaauuuggaucggagggaguaguagaagugu         exp
    ((((((((((((((((((((((......))))))))))))))))))))))..(.((((....(((..((((((...........))))))..)))....)))).)........  reads   mm   sample
    cuccguaaagaaauauaaga..............................................................................................  1       0    S01
    cuccguaaagaaauauaagagc............................................................................................  1       0    S01
    cuccguaaagaaauauaagagc............................................................................................  1       0    S05
    .uccguaaagaaauauaagag.............................................................................................  1       0    S03
    .uccguaaagaaauauaagag.............................................................................................  1       0    S06
    .uccguaaagaaauauaagag.............................................................................................  3       0    S04
    .uccgGaaagaaauauaagag.............................................................................................  1       1    S06
    .uccgGaaagaaauauaagag.............................................................................................  1       1    S03
    .uccguaaUgaaauauaagagc............................................................................................  1       1    S05
    .uccguaaagaaauauaagagc............................................................................................  67      0    S02
    .uccguaaUgaaauauaagagc............................................................................................  1       1    S03
    .uccguaaagaaauauaagagc............................................................................................  68      0    S06
    .uccguaaUgaaauauaagagc............................................................................................  1       1    S01
    .uccguaaUgaaauauaagagc............................................................................................  1       1    S04
    .uccguaaagaaauauaagagc............................................................................................  150     0    S05
    .uccguaaagaaauauaagagc............................................................................................  62      0    S03
    .uccguaaagaaauauaagagc............................................................................................  96      0    S04
    .uccguaaagaaauauaagagc............................................................................................  88      0    S01
    .uccguaaagaaauauaagagcu...........................................................................................  1       0    S05
    .uccguaaagaaauauaagagcu...........................................................................................  1       0    S04
    ..ccguaaagaaauauaagagc............................................................................................  1       0    S01
    ..ccguaaagaaauauaagagc............................................................................................  1       0    S04
    ..ccguaaagaaauauaagagc............................................................................................  2       0    S03
    ..ccguaaagaaauauaagagc............................................................................................  1       0    S06
    ..ccguaaagaaauauaagagc............................................................................................  2       0    S05
    ...cgGaaagaaauauaagagc............................................................................................  1       1    S06
    ...cguaaagaaauauaagagc............................................................................................  2       0    S01
    ....guaaagaaauauaagagc............................................................................................  1       0    S06
    ....guaaagaaauauaagagc............................................................................................  1       0    S01
    ....guaaagaaauauaagagc............................................................................................  2       0    S04
    .....uaaagaaauauaagagcu...........................................................................................  1       0    S06
    ...............................cuuauauuuuuuuacggaUg...............................................................  1       1    S01
    ...............................cuuauauuuuuuuacggaUgg..............................................................  1       1    S02
```

Mature **Star**

cuccguaaagaaauauaagagcuuuagugcucuuauauuuuuuuacggagggaguacuaaaguuguaccaaaugugcgucaauuaauuuggaucggagggaguaguagaagugu			
...............................cuuauauuuuuuuacggaUgga...	1	1	S03
...............................cuuauauuuuuuuacggaUgga...	1	1	S02
...............................cuuauauuuuuuuacggagggagu...	1	0	S02
...............................cuuauauuuuuuuacggaUggagu...	1	1	S02
....aaugugcgucaauuaauuuggauc....................	1	0	S01
....ugugcgucaauuaauuuggau.....................	1	0	S01
....ugugcgucaauuaauuuggau.....................	1	0	S06
....ugugcgucaauuaauuuggaucgg..................	1	0	S05
....gugcgucaauuaauuuggaucgg..................	1	0	S03
....aauuaauuuggaucggaggga.............	1	0	S05
....aauuaauuuggaucggagggagu...........	1	0	S01
....auuaauuuggaucggagggag............	1	0	S02
....auuaauuuggaucggagggagu...........	1	0	S01
....auuaauuuggaucggagggagua..........	1	0	S04
....auuaauuuggaucggaggUaguagua.......	1	1	S04
....uuaauuuggaucggagggagu...........	2	0	S04
....uuaauuuggaucggagggagu...........	1	0	S02

Provisional ID	:	ta_iwgsc_1ds_v1_1226703_793287
Score total	:	1.4
Score for star read(s)	:	-1.3
Score for read counts	:	0
Score for mfe	:	1.1
Score for randfold	:	1.6
Score for cons. seed	:	
Total read count	:	62
Mature read count	:	83
Loop read count	:	0
Star read count	:	30

novel-m0687-5p

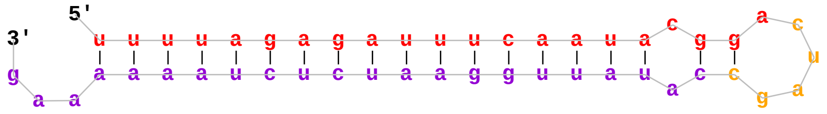

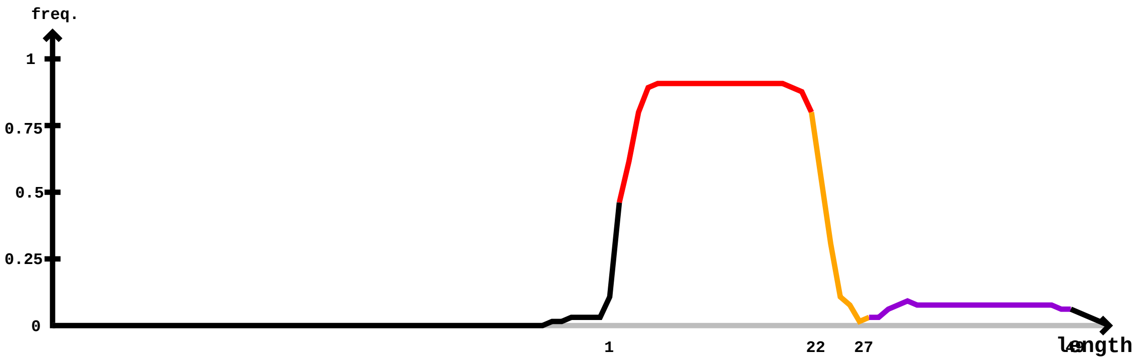


```
                                                            Mature         Star
5'- gaggcaauuucauaagcaccucuaaaagcaacaacuuccguuuuuagauauaagccuuuuuagagauuucaauacggacuagccauauuggaaucucuaaaaaagcuu  -3'   obs
    gaggcaauuucauaagcaccucuaaaagcaacaacuuccguuuuuagauauaagccuuuuuagagauuucaauacggacuagccauauuggaaucucuaaaaaagcuu        exp
    ......................(((((((((..........)))))))))...((((.(((((((((((((((((.((.....)).))))))))))))))))).))))   reads   mm   sample
    ..................................................uaagccuuuuuagagauuuc......................................   1       0    S02
    ....................................................agccCuuuuagagauuucaauacg................................   1       1    S02
    ........................................................uuuuuagagauuucaauac.................................   1       0    S04
    ........................................................uuuuuagagauuucaauacgg...............................   2       0    S01
    ........................................................uuuuuagagauuucaauacgga..............................   1       0    S04
    ........................................................uuuuuagagauuucaauacgga..............................   1       0    S05
    .........................................................uuuuagagauuucaauacgg...............................   1       0    S01
    .........................................................uuuuagagauuucaauacgga..............................   5       0    S05
    .........................................................uuuuagagauuucaauacgga..............................   2       0    S03
    .........................................................uuuuagagauuucaauacgga..............................   5       0    S02
    .........................................................uuuuagagauuucaauacgga..............................   7       0    S04
    .........................................................uuuuagagauuucaauacgga..............................   1       0    S01
    .........................................................uuuuagagauuucaauacgga..............................   1       0    S06
    .........................................................uuuuagagauuucaauacggaU.............................   1       1    S04
    ..........................................................uuuagagauuucaauacgg...............................   1       0    S04
    ..........................................................uuuagagauuucaauacgg...............................   1       0    S02
    ..........................................................uuuagagauuucaauacgga..............................   1       0    S04
    ..........................................................uuuagagauuucaauacgga..............................   1       0    S05
    ..........................................................uuuagagauuucaauacgga..............................   1       0    S01
    ..........................................................uuuagagauuucaauacgga..............................   1       0    S06
    ..........................................................uuuagagauuucaauacggaUu............................   1       1    S02
    ..........................................................uuuagagauuucaauacggaUu............................   1       1    S05
    ..........................................................uuuagagauuucaauacggacua...........................   1       0    S05
    ..........................................................uuuagagauuucaauacggacuaU..........................   1       1    S05
    ...........................................................uuagagauuucaauacgga..............................   2       0    S04
    ...........................................................uuagagauuucaauacgga..............................   1       0    S05
    ...........................................................uuagagauuucaauacggacu............................   1       0    S02
    ...........................................................uuagagauuucaauacggacu............................   1       0    S06
    ...........................................................uuagagauuucaauacggaUu............................   2       1    S05
    ...........................................................uuagagauuucaauacggacu............................   2       0    S01
    ...........................................................uuagagauuucaauacggacu............................   1       0    S05
    ...........................................................uuagagauuucaauacggacuaU..........................   2       1    S05
    ............................................................uagagauuucaauacggaU.............................   1       1    S02
```

```
                                                        Mature          Star
gaggcaauuucauaagcaccucuaaaagcaacaacuuccguuuuuagauauaagccuuuuuagagauuucaauacggacuagccauauuggaaucucuaaaaaaagcuu
............................................................uagagauuucaauacggacu.............................    1    0    S01
............................................................uagagauuucaauacggacu.............................    2    0    S02
............................................................uagagauuucaauacggacua............................    1    0    S05
............................................................uagagauuucaauacggacuaU...........................    1    1    S01
.............................................................agagauuucaauacggacu.............................    1    0    S04
......................................................................aauacggacuagccauau.....................    1    0    S06
...................................................................................cauauuggaaucucuaaaaaaag...    1    0    S04
.....................................................................................uauuggaaucucuaaaaaa.....    1    0    S01
.....................................................................................uauuggaaucucuaaaaaaag...    1    0    S05
......................................................................................auuggaaucucuaaaaaaagcu.    1    0    S01
.......................................................................................uuggaaucucuaaaaaaagcuu    1    0    S05
```

Provisional ID	:	ta_iwgsc_1ds_v1_1901389_847124
Score total	:	142.6
Score for star read(s)	:	3.9
Score for read counts	:	134.7
Score for mfe	:	2.3
Score for randfold	:	1.6
Score for cons. seed	:	
Total read count	:	276
Mature read count	:	280
Loop read count	:	0
Star read count	:	107

novel-m0049-3p

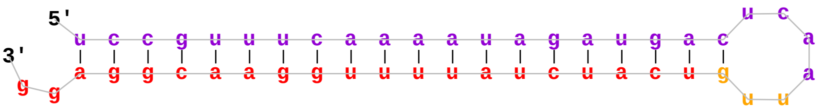

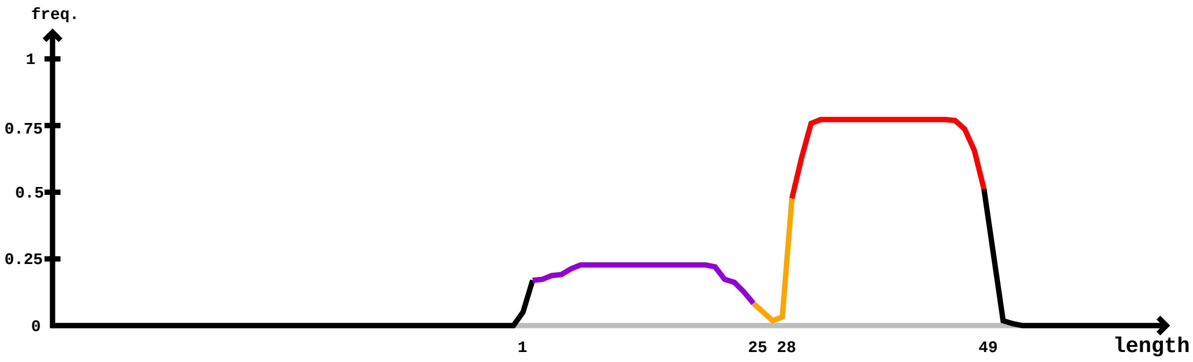

Star Mature

```
5'- gauccagugguuucagguuguaauuugggcuggauagaucguacucccuccguuucaaaauagaugacucaauugucaucuauuuuggaacggagggggggccggcggacaucg  -3'   obs
    gauccagugguuucagguuguaauuugggcuggauagaucguacucccuccguuucaaaauagaugacucaauugucaucuauuuuggaacggagggggggccggcggacaucg        exp
    .(((((((....((((((....)))))))))))))...(((..(((((((((((((((((((((((((......)))))))))))))))))))))))))...)))......... reads   mm   sample
    ...............................................cuccguuucaaaauagauga...............................................  1      0    S04
    ...............................................cuccguuucaaaauagauga...............................................  1      0    S02
    ...............................................cuccguuucaaaauagaugac..............................................  4      0    S01
    ...............................................cuccguuucaaaauagaugac..............................................  2      0    S05
    ...............................................cuccguuucaaaauagaugac..............................................  3      0    S03
    ...............................................cuccguuucaaaauagaugac..............................................  2      0    S02
    ...............................................cuccguuucaaaauagaugacuca...........................................  1      0    S03
    ................................................uccguuucaaaauagaugac..............................................  2      0    S02
    ................................................uccguuucaaaauagaugacu.............................................  2      0    S05
    ................................................uccguuucaaaauagaugacuc............................................  3      0    S05
    ................................................uccguuucaaaauagaugacuc............................................  5      0    S04
    ................................................uccguuucaaaauagaugacuc............................................  1      0    S02
    ................................................uccguuucaaaauagaugacuca...........................................  1      0    S05
    ................................................uccguuucaaaauagaugacuca...........................................  2      0    S02
    ................................................uccguuucaaaauagaugacuca...........................................  2      0    S04
    ................................................uccguuucaaaauagaugacucaa..........................................  1      0    S03
    ................................................uccguuucaaaauagaugacucaa..........................................  2      0    S06
    ................................................uccguuucaaaauagaugacucaa..........................................  7      0    S04
    ................................................uccguuucaaaauagaugacucaa..........................................  5      0    S05
    .................................................ccguuucaaaauagaugacuca...........................................  1      0    S05
    ..................................................cguuucaaaauagaugacuca...........................................  2      0    S05
    ..................................................cguuucaaaauagaugacuca...........................................  1      0    S01
    ..................................................cguuucaaaauagaugacuca...........................................  1      0    S04
    ...................................................guuucaaaauagaugacu.............................................  1      0    S01
    ....................................................uuucaaaauagaugacuc............................................  1      0    S05
    ....................................................uuucaaaauagaugacuc............................................  1      0    S06
    ....................................................uuucaaaauagaugacuca...........................................  1      0    S01
    ....................................................uuucaaaauagaugacucaa..........................................  2      0    S01
    ....................................................uuucaaaauagaugacucaauu........................................  1      0    S05
    .....................................................uucaaaauagaugacucaa..........................................  2      0    S05
    .....................................................uucaaaauagaugacucaauu........................................  1      0    S05
    .....................................................uucaaaauagaugacucaauu........................................  1      0    S01
    ......................................................................aauugucaucuauuuuggaacgga....................  1      0    S06
```

```
                                                 Star                       Mature
gauccagugguuucagguuguaauuugggcuggauagaucguacucccuccguuucaaaauagaugacucaauugucaucuauuuuggaacggagggggggccggcggacaucg
.........................................................................ugucaucuauuuuggaacgg.....................   1   0   S02
..........................................................................gucaucuauuuuggaacg......................   1   0   S05
..........................................................................gucaucuauuuuggaacgga....................   1   0   S06
..........................................................................gucaucuauuuuggaacgga....................   2   0   S04
..........................................................................gucaucuauuuuggaacggag...................   2   0   S06
..........................................................................gucaucuauuuuggaacggag...................   1   0   S01
...........................................................................ucaucuauuuuggaacgg.....................   1   0   S02
...........................................................................ucaucuauuuuggaacgg.....................   1   0   S03
...........................................................................ucaucuauuuuggaacgg.....................   3   0   S06
...........................................................................ucaucuauuuuggaacgg.....................   1   0   S01
...........................................................................ucaucuauuuuggaacgg.....................   2   0   S05
...........................................................................ucaucuauuuuggaacgga....................   1   0   S04
...........................................................................ucaucuauuuuggaacgga....................   5   0   S01
...........................................................................ucaucuauuuuggaacgga....................   2   0   S03
...........................................................................ucaucuauuuuggaacgga....................   2   0   S02
...........................................................................ucaucuauuuuggaacgga....................   1   0   S06
...........................................................................ucaucuauuuuggaacggag...................   2   0   S01
...........................................................................ucaucuauuuuggaacggag...................   3   0   S06
...........................................................................ucaucuauuuuggaacggag...................   2   0   S05
...........................................................................ucaucuauuuuggaacggag...................   8   0   S03
...........................................................................ucaucuauuuuggaacggag...................   3   0   S04
...........................................................................ucaucuauuuuggaacggag...................   9   0   S02
...........................................................................ucaucuauuuuggaacggagg..................   19  0   S05
...........................................................................ucaucuauuuuggaacggagg..................   17  0   S01
...........................................................................ucaucuauuuuggaacggagg..................   5   0   S03
...........................................................................ucaucuauuuuggaacggagg..................   14  0   S04
...........................................................................ucaucuauuuuggaacggagg..................   6   0   S06
...........................................................................ucaucuauuuuggaacggagg..................   15  0   S02
...........................................................................ucaucuauuuuggaacggagAgg................   1   1   S06
............................................................................caucuauuuuggaacgga....................   1   0   S04
............................................................................caucuauuuuggaacgga....................   1   0   S05
............................................................................caucuauuuuggaacgga....................   1   0   S01
............................................................................caucuauuuuggaacgga....................   3   0   S06
............................................................................caucuauuuuggaacgga....................   1   0   S03
............................................................................caucuauuuuggaacggag...................   1   0   S03
............................................................................caucuauuuuggaacggag...................   1   0   S06
............................................................................caucuauuuuggaacggag...................   1   0   S05
............................................................................caucuauuuuggaacggagg..................   2   0   S02
............................................................................caucuauuuuggaacggagg..................   10  0   S06
............................................................................caucuauuuuggaacggagg..................   1   0   S03
............................................................................caucuauuuuggaacggagg..................   3   0   S01
............................................................................caucuauuuuggaacggaggg.................   1   0   S06
............................................................................caucuauuuuggaacggaggg.................   2   0   S02
............................................................................caucuauuuuggaacggaggg.................   3   0   S01
............................................................................caucuauuuuggaacggaggg.................   2   0   S03
............................................................................caucuauuuuggaacggaggg.................   5   0   S04
............................................................................caucuauuuuggaacggaggg.................   3   0   S05
.............................................................................aucuauuuuggaacggag...................   1   0   S04
.............................................................................aucuauuuuggaacggag...................   3   0   S06
.............................................................................aucuauuuuggaacggag...................   3   0   S05
.............................................................................aucuauuuuggaacggagg..................   2   0   S06
.............................................................................aucuauuuuggaacggagg..................   2   0   S02
.............................................................................aucuauuuuggaacggagg..................   2   0   S04
.............................................................................aucuauuuuggaacggaggg.................   5   0   S02
.............................................................................aucuauuuuggaacggaggg.................   5   0   S03
.............................................................................aucuauuuuggaacggaggg.................   3   0   S05
.............................................................................aucuauuuuggaacggaggg.................   2   0   S01
.............................................................................aucuauuuuggaacggaggg.................   2   0   S04
.............................................................................aucuauuuuggaacggaggg.................   4   0   S06
.............................................................................aucuauuuuggaacggagggg................   1   0   S05
.............................................................................aucuauuuuggaacggagggg................   1   0   S04
..............................................................................ucuauuuuggaacggagg..................   1   0   S04
..............................................................................ucuauuuuggaacggaggg.................   1   0   S04
..............................................................................ucuauuuuggaacggaggggg...............   1   0   S02
..............................................................................ucuauuuuggaacggaggggg...............   1   0   S06
```

Provisional ID	:	ta_iwgsc_2al_v1_6345426_968131
Score total	:	185.4
Score for star read(s)	:	3.9
Score for read counts	:	177.5
Score for mfe	:	2.3
Score for randfold	:	1.6
Score for cons. seed	:	
Total read count	:	360
Mature read count	:	254
Loop read count	:	0
Star read count	:	342

novel-m0294-5p

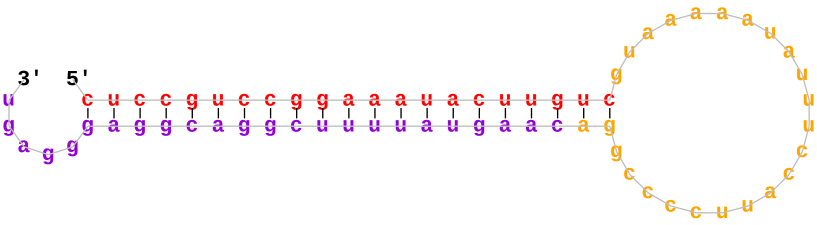

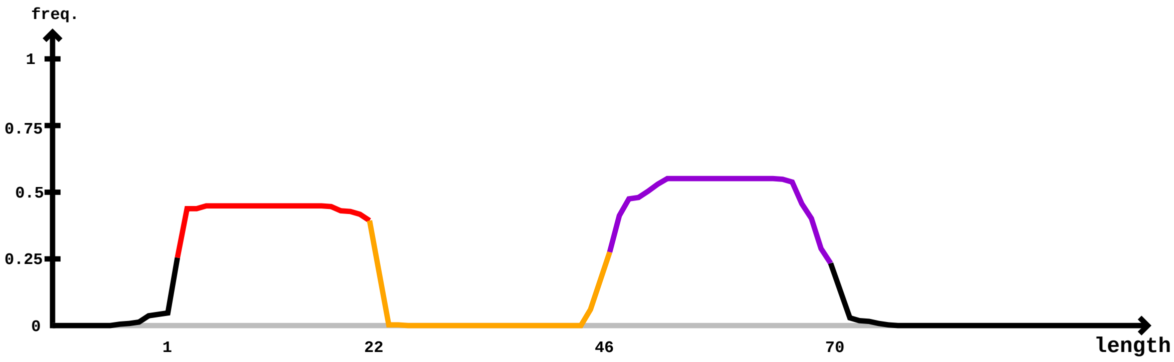


```
                  Mature                                    Star
5'- ccugauacucccuccguccggaaauacuugucguaaaaauauuuccauuccccggacaaguauuuucggacggagggaguagaaaucacuacagaugugaucuucuuuccua  -3'   obs
    ccugauacucccuccguccggaaauacuugucguaaaaauauuuccauuccccggacaaguauuuucggacggagggaguagaaaucacuacagaugugaucuucuuuccua        exp
    .....(((((((((((((((((((((((((((......................)))))))))))))))))))))))))))((((((((.......))))).))).......   reads   mm   sample
    .....uacucccuccguccggaaauacu....................................................................................   1       0    S03
    .....uacucccuccguccggaaauacC....................................................................................   1       1    S03
    ......acucccuccguccggaaauac.....................................................................................   1       0    S02
    .......cucccuccguccggaaauacu....................................................................................   1       0    S05
    .......cucccuccguccggaaauacuuguc................................................................................   1       0    S05
    ........ucccuccguccggaaauacu....................................................................................   1       0    S01
    ........ucccuccguccggaaauacuuguc................................................................................   2       0    S02
    ........ucccuccguccggaaauacuuguc................................................................................   1       0    S03
    ........ucccuccguccggaaauacuuguc................................................................................   1       0    S05
    ........ucccuccguccggaaauacuuguc................................................................................   1       0    S06
    ........ucccuccguccggaaauacuuguc................................................................................   3       0    S04
    .........cccuccguccggaaauacuuguc................................................................................   1       0    S01
    .........cccuccguccggaaauacuuguc................................................................................   1       0    S03
    ..........ccuccguccggaaauacu....................................................................................   2       0    S04
    ...........cuccguccggaaauacuu...................................................................................   1       0    S04
    ...........cuccguccggaaauacuug..................................................................................   1       0    S02
    ...........cuccguccggaaauacuugu.................................................................................   1       0    S02
    ...........cuccguccggaaauacuugu.................................................................................   1       0    S03
    ...........cuccguccggaaauacuuguc................................................................................   10      0    S06
    ...........cuccguccggaaauacuuguc................................................................................   15      0    S01
    ...........cuccguccggaaauacuuguc................................................................................   12      0    S02
    ...........cuccguccggaaauacuuguc................................................................................   8       0    S04
    ...........cuccguccggaaauacuuguc................................................................................   12      0    S03
    ...........cuccguccggaaauacuuguc................................................................................   18      0    S05
    ............uccguccggaaauacuug..................................................................................   1       0    S01
    ............uccguccggaaauacuug..................................................................................   2       0    S04
    ............uccguccggaaauacuugu.................................................................................   1       0    S03
    ............uccguccggaaauacuugu.................................................................................   2       0    S04
    ............uccguccggaaauacuugu.................................................................................   1       0    S06
    ............uccguccggaaauacuugu.................................................................................   2       0    S02
    ............uccguccggaaauacuugu.................................................................................   1       0    S01
    ............uccguccggaaauacuuguc................................................................................   7       0    S05
    ............uccguccggaaauacuuguc................................................................................   4       0    S03
```

```
            Mature                                            Star
ccugauacucccuccguccggaaauacuugucguaaaaaauauuuccauucccggacaaguauuuucggacggagggaguagaaaucacuacagaugugaucuucuuuccua
............uccguccggaaauacuuguc................................................................................   8    0   S01
............uccguccggaaauacuuguc................................................................................   10   0   S02
............uccguccggaaauacuuguc................................................................................   3    0   S04
............uccguccggaaauacuuguc................................................................................   2    0   S06
............uccguccggaaauacuugucg...............................................................................   3    0   S03
............uccUuccggaaauacuugucg...............................................................................   1    1   S02
............uccguccggaaauacuugucg...............................................................................   7    0   S05
............uccguccggaaauacuugucg...............................................................................   4    0   S04
............uccguccggaaauacuugucg...............................................................................   4    0   S06
............uccguccggaaauacuugucg...............................................................................   3    0   S01
............uccguccggaaauacuugucg...............................................................................   4    0   S02
..............cguccggaaauacuuguc................................................................................   1    0   S01
..............cguccggaaauacuuguc................................................................................   1    0   S05
..............cguccggaaauacuugucg...............................................................................   1    0   S06
..............cguccggaaauacuugucgua.............................................................................   1    0   S04
......................................................gacaaguauuuucggacggag.....................................   1    0   S06
......................................................gacaaguauuuucggacggagg....................................   2    0   S01
......................................................gacaaguauuuucggacggaggg...................................   1    0   S04
......................................................gacaaguauuuucggacUgaggg...................................   1    1   S05
......................................................gacaaAuauuuucggacggaggga..................................   3    1   S04
......................................................gacaaguauuuucggacggaggga..................................   2    0   S02
......................................................gacaaguauuuucggacggaggga..................................   2    0   S06
......................................................gacaaguauuuucggacggaggga..................................   8    0   S04
......................................................gacaaguauuuucggacggaggga..................................   3    0   S05
.......................................................acaaguauuuucggacgga......................................   1    0   S05
.......................................................acaaguauuuucggacggag.....................................   1    0   S04
.......................................................acaaguauuuucggacggag.....................................   1    0   S06
.......................................................acaaguauuuucggacggagg....................................   2    0   S01
.......................................................acaaguauuuucggacggagg....................................   8    0   S02
.......................................................acaaguauuuucggacggagg....................................   7    0   S05
.......................................................acaaguauuuucggacggagg....................................   1    0   S03
.......................................................acaaguauuuucggacggagg....................................   4    0   S04
.......................................................acaaguauuuucggacggagg....................................   3    0   S06
.......................................................acaaguauuuucggacggaggg...................................   1    0   S05
.......................................................acaaguauuuucggacggaggg...................................   1    0   S03
.......................................................acaaguauuuucggacggaggg...................................   1    0   S04
.......................................................acaaguauuuucggacggaggga..................................   3    0   S04
.......................................................acaaguauuuucggacggagggag.................................   1    0   S05
.......................................................acaaguauuuucggacggagggag.................................   1    0   S02
.......................................................acaaguauuuucggacggagggag.................................   2    0   S04
.......................................................acaaAuauuuucggacggagggag.................................   1    1   S04
.......................................................acaaguauuuucggacggagggagu................................   1    0   S02
.......................................................acaaguauuuucggacggagggagu................................   1    0   S01
........................................................caaguauuuucggacggag.....................................   1    0   S05
........................................................caaguauuuucggacggagg....................................   1    0   S03
........................................................caaguauuuucggacggagg....................................   1    0   S01
........................................................caaguauuuucggacggagg....................................   1    0   S05
........................................................caaguauuuucggacggaggg...................................   1    0   S04
........................................................caaguauuuucggacggaggg...................................   1    0   S05
........................................................caaguauuuucggacggaggg...................................   3    0   S01
........................................................caaguauuuucggacggaggga..................................   1    0   S04
........................................................caaguauuuucggacggagggag.................................   1    0   S02
........................................................caaguauuuucggacggagggag.................................   1    0   S05
........................................................caaguauuuucggacggagggag.................................   1    0   S01
........................................................caaAuauuuucggacggagggagu................................   1    1   S02
........................................................caaguauuuucggacggagggagu................................   5    0   S06
........................................................caaguauuuucggacggagggagu................................   5    0   S04
........................................................caaguauuuucggacggagggagu................................   4    0   S05
........................................................caaAuauuuucggacggagggagu................................   2    1   S05
........................................................caaguauuuucggacggagggagu................................   9    0   S02
........................................................caaguauuuucggacggagggagA................................   1    1   S04
........................................................caaAuauuuucggacggagggagu................................   1    1   S06
.........................................................aaguauuuucggacggagg....................................   1    0   S04
.........................................................aaguauuuucggacggaggg...................................   1    0   S05
.........................................................aaguauuuucggacggaggg...................................   2    0   S02
.........................................................aaguauuuucggacggaggg...................................   1    0   S03
.........................................................aaguauuuucggacggaggg...................................   1    0   S01
.........................................................aaguauuuucggacggaggg...................................   2    0   S04
.........................................................aaguauuuucggacggaggga..................................   4    0   S02
.........................................................aaguauuuucggacggaggga..................................   7    0   S05
```

```
           Mature                                        Star
ccugauacucccuccguccggaaauacuugucguaaaaaauauuuccauuccccggacaaguauuuucggacggagggaguagaaaucacuacagaugugaucuucuuuccua
..........................................................aaguauuuucggacggaggga..................................   3   0   S01
..........................................................aaguauuuucggacggaggga..................................   6   0   S04
..........................................................aaguauuuucggacggagggagA................................   1   1   S05
..........................................................aaguauuuucggacUgagggagu................................   1   1   S06
..........................................................aaguauuuucggacggagggagu................................   2   0   S02
..........................................................aaguauuuucggacggagggagA................................   1   1   S04
..........................................................aaguauuuucggacggagggagu................................   6   0   S05
..........................................................aaguauuuucggacggagggagu................................   1   0   S06
..........................................................aaguauuuucggacggagggagu................................   2   0   S04
..........................................................aaguauuuucggacggagggagua...............................   2   0   S03
..........................................................aaguauuuucggacggagggagua...............................   4   0   S04
..........................................................aaguauuuucggacggagggagua...............................   3   0   S01
..........................................................aaguauuuCcggacggagggaguagaaa...........................   1   1   S02
...........................................................aguauuuucggacggaggg...................................   2   0   S02
...........................................................aguauuuucggacggaggg...................................   1   0   S04
...........................................................aguauuuucggacggaggga..................................   1   0   S02
...........................................................aguauuuucggacggagggag.................................   1   0   S03
...........................................................aguauuuucggacggagggag.................................   6   0   S02
...........................................................aguauuuucggacggagggag.................................   1   0   S01
...........................................................aguauuuucggacggagggag.................................   3   0   S06
...........................................................aguauuuucggacggagggagua...............................   8   0   S04
...........................................................aguauuuucggacggagggaguag..............................   1   0   S04
............................................................guauuuucggacggaggg...................................   1   0   S04
............................................................guauuuucggacggagggagu................................   1   0   S02
.............................................................uauuuucggacggagggag.................................   1   0   S04
.............................................................uauuuucggacggagggagu................................   1   0   S04
.............................................................uauuuucggacggagggagu................................   1   0   S05
.............................................................uauuuucggacggagggagua...............................   2   0   S01
.............................................................uauuuucggacggagggagua...............................   1   0   S04
.............................................................uauuuucggacggagggagua...............................   1   0   S03
.............................................................uauuuucggacggagggaguaga.............................   1   0   S04
.............................................................uauuuucggacggagggaguagaaa...........................   1   0   S03
..............................................................auuuucggacggagggag.................................   1   0   S04
..............................................................auuuucggacggagggagu................................   2   0   S04
..............................................................auuuucggacggagggagua...............................   1   0   S04
..............................................................auuuucggacggagggagua...............................   1   0   S06
..............................................................auuuucggacggagggaguag..............................   1   0   S02
..............................................................auuuucggacggagggaguag..............................   1   0   S05
..............................................................auuuucggacggagggaguaAaa............................   1   1   S06
..............................................................auuuucggacggagggaguagaa............................   2   0   S02
...............................................................uuuucggacggagggagu................................   3   0   S05
...............................................................uuuucggacggagggagu................................   1   0   S01
...............................................................uuuucggacggagggagu................................   2   0   S04
...............................................................uuuucggacggagggaguag..............................   1   0   S01
...............................................................uuuucggacggagggaguagaaau..........................   1   0   S05
```

Provisional ID	:	ta_iwgsc_2al_v1_6363060_994006
Score total	:	2332.1
Score for star read(s)	:	3.9
Score for read counts	:	2323.9
Score for mfe	:	2.7
Score for randfold	:	1.6
Score for cons. seed	:	
Total read count	:	4570
Mature read count	:	5139
Loop read count	:	0
Star read count	:	6

novel-m0661-5p

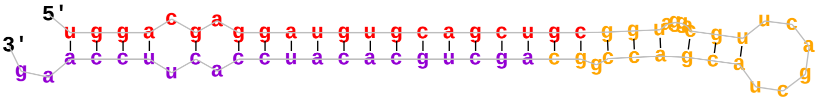

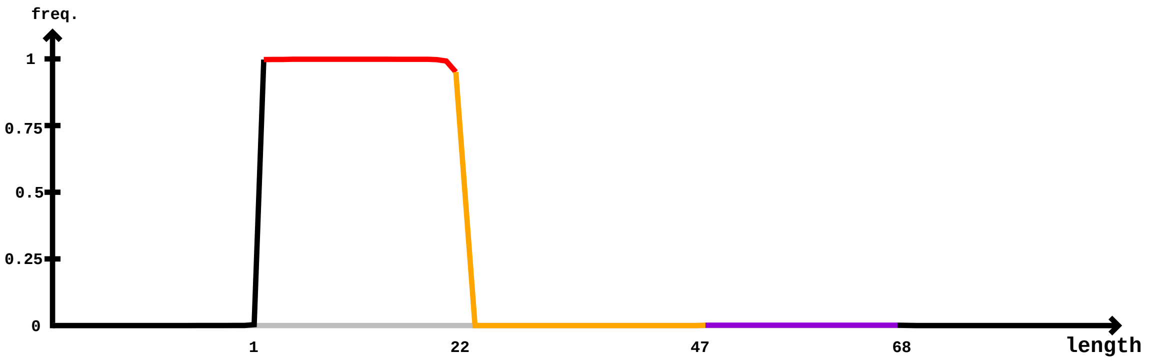

Mature Star

```
5'- guacgguccugguuagaguuuggacgaggaugugcagcugcgguaggacguucagcuacgaccggcagcugcacauccacuuccaagcgcuagcuaggaucgucaacaa  -3'  obs
    guacgguccugguuagaguuuggacgaggaugugcagcugcgguaggacguucagcuacgaccggcagcugcacauccacuuccaagcgcuagcuaggaucgucaacaa       exp
    ..((((((((((((((.(((((((.(.(((((((((((((((((....(((......)))))).)))))))))))))).).))))))).))))))))))))))......  reads  mm  sample
    .............uagaguuuggacgaggaugug...........................................................................  1      0   S05
    .................guuuggacgaggaugugcagc.......................................................................  1      0   S04
    ...................uuggacgaggaugugcagcu......................................................................  1      0   S01
    ...................uuggacgaggaugugcagcu......................................................................  1      0   S04
    ...................uuggacgaggaugugcagcug.....................................................................  3      0   S05
    ...................uuggacgaggaugugcagcug.....................................................................  2      0   S01
    ...................uuggacgaggaugugcagcug.....................................................................  6      0   S04
    ...................uuggacgaggaugugcagcugc....................................................................  1      0   S02
    ....................uggacgaggaugugcagc.......................................................................  3      0   S05
    ....................uggacgaggaugugcagc.......................................................................  2      0   S02
    ....................uggacgaggaugugcagcu......................................................................  5      0   S02
    ....................uggacgaggaugugcagcu......................................................................  3      0   S04
    ....................uggacgaggaugugcagcu......................................................................  7      0   S01
    ....................uggacgaggaugugcagcu......................................................................  4      0   S03
    ....................uggacgaggaugugcagcu......................................................................  2      0   S05
    ....................uggacgaggaugugcagcug.....................................................................  47     0   S02
    ....................uggacgaggaugugcagcug.....................................................................  33     0   S04
    ....................uggacgaggaugugcagcug.....................................................................  20     0   S01
    ....................uggacgaggaugugcagcug.....................................................................  9      0   S06
    ....................uggacgaggaugugcagcug.....................................................................  20     0   S05
    ....................uggacgaggaugugcagcug.....................................................................  49     0   S03
    ....................uggacgaggaugugcagcugc....................................................................  121    0   S06
    ....................uggacgaggaugugcagcugc....................................................................  892    0   S04
    ....................uggacgaggaugugcagcugc....................................................................  634    0   S05
    ....................uggacgaggaugugcagcugc....................................................................  842    0   S01
    ....................uggacgaggaugugcagcugc....................................................................  382    0   S03
    ....................uggacgaggaugugcagcugc....................................................................  1469   0   S02
    .....................ggacgaggaugugcagcugc....................................................................  1      0   S02
    .....................ggacgaggaugugcagcugc....................................................................  1      0   S01
    .......................acgaggaugugcagcugc....................................................................  1      0   S01
    .......................acgaggaugugcagcugc....................................................................  2      0   S03
    .......................acgaggaugugcagcugc....................................................................  1      0   S02
    ..................................................................agcugcacauccacuuccaag......................  2      0   S02
```

```
                    Mature                                            Star
guacgguccugguuagaguuuggacgaggaugugcagcugcgguaggacguucagcuacgaccggcagcugcacauccacuuccaagcgcuagcuaggaucgucaacaa
..................................................................agcugcacauccacuuccaag......................   3   0   S03
..................................................................agcugcacauccacuuccaag......................   1   0   S01
```

Provisional ID	: ta_iwgsc_2al_v1_6418433_1076127
Score total	: 2332.1
Score for star read(s)	: 3.9
Score for read counts	: 2323.9
Score for mfe	: 2.7
Score for randfold	: 1.6
Score for cons. seed	:
Total read count	: 4570
Mature read count	: 5139
Loop read count	: 0
Star read count	: 6

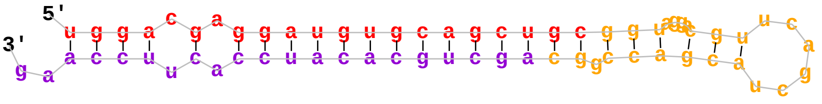

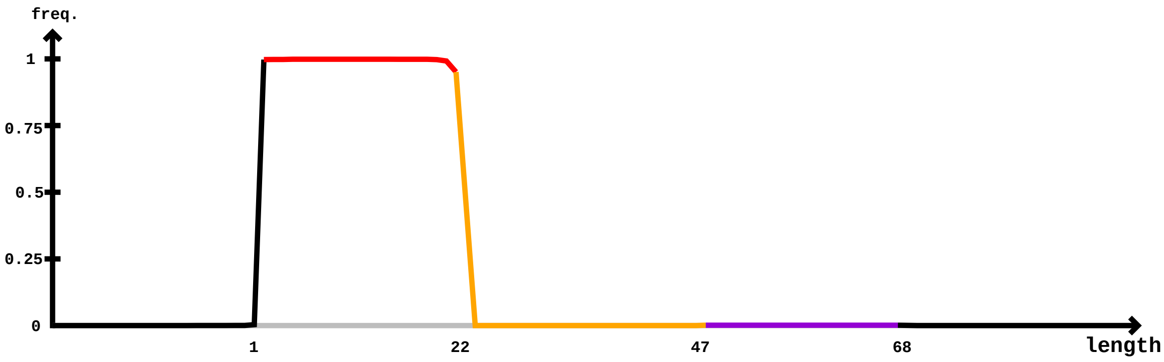

Mature **Star**

```
5'- guacgguccugguuagaguuuggacgaggaugugcagcugcgguaggacguucagcuacgaccggcagcugcacauccacuuccaagcgcuagcuaggaucgucaacaacg  -3'  obs
    guacgguccugguuagaguuuggacgaggaugugcagcugcgguaggacguucagcuacgaccggcagcugcacauccacuuccaagcgcuagcuaggaucgucaacaacg        exp
    ..((((((((((((((.(((((((.(.(((((((((((((((((....(((......)))))).))))))))))))))).).))))))).))))))))))))))........  reads  mm  sample
    .............uagaguuuggacgaggaugug.............................................................................  1     0   S05
    .................guuuggacgaggaugugcagc.........................................................................  1     0   S04
    ...................uuggacgaggaugugcagcu........................................................................  1     0   S01
    ...................uuggacgaggaugugcagcu........................................................................  1     0   S04
    ...................uuggacgaggaugugcagcug.......................................................................  3     0   S05
    ...................uuggacgaggaugugcagcug.......................................................................  2     0   S01
    ...................uuggacgaggaugugcagcug.......................................................................  6     0   S04
    ...................uuggacgaggaugugcagcugc......................................................................  1     0   S02
    ....................uggacgaggaugugcagc.........................................................................  3     0   S05
    ....................uggacgaggaugugcagc.........................................................................  2     0   S02
    ....................uggacgaggaugugcagcu........................................................................  5     0   S02
    ....................uggacgaggaugugcagcu........................................................................  3     0   S04
    ....................uggacgaggaugugcagcu........................................................................  7     0   S01
    ....................uggacgaggaugugcagcu........................................................................  4     0   S03
    ....................uggacgaggaugugcagcu........................................................................  2     0   S05
    ....................uggacgaggaugugcagcug.......................................................................  47    0   S02
    ....................uggacgaggaugugcagcug.......................................................................  33    0   S04
    ....................uggacgaggaugugcagcug.......................................................................  20    0   S01
    ....................uggacgaggaugugcagcug.......................................................................  9     0   S06
    ....................uggacgaggaugugcagcug.......................................................................  20    0   S05
    ....................uggacgaggaugugcagcug.......................................................................  49    0   S03
    ....................uggacgaggaugugcagcugc......................................................................  121   0   S06
    ....................uggacgaggaugugcagcugc......................................................................  892   0   S04
    ....................uggacgaggaugugcagcugc......................................................................  634   0   S05
    ....................uggacgaggaugugcagcugc......................................................................  842   0   S01
    ....................uggacgaggaugugcagcugc......................................................................  382   0   S03
    ....................uggacgaggaugugcagcugc......................................................................  1469  0   S02
    .....................ggacgaggaugugcagcugc......................................................................  1     0   S02
    .....................ggacgaggaugugcagcugc......................................................................  1     0   S01
    .......................acgaggaugugcagcugc......................................................................  1     0   S01
    .......................acgaggaugugcagcugc......................................................................  2     0   S03
    .......................acgaggaugugcagcugc......................................................................  1     0   S02
    ..................................................................agcugcacauccacuuccaag........................  2     0   S02
```

```
                    Mature                                       Star
guacgguccugguuagaguuuggacgaggaugugcagcugcgguaggacguucagcuacgaccggcagcugcacauccacuuccaagcgcuagcuaggaucgucaacaacg
..................................................................agcugcacauccacuuccaag........................    3    0    S03
..................................................................agcugcacauccacuuccaag........................    1    0    S01
```

Provisional ID	:	ta_iwgsc_2ds_v1_5338269_2133933
Score total	:	155.5
Score for star read(s)	:	3.9
Score for read counts	:	148
Score for mfe	:	2.1
Score for randfold	:	1.6
Score for cons. seed	:	
Total read count	:	302
Mature read count	:	97
Loop read count	:	0
Star read count	:	338

novel-m0238-3p

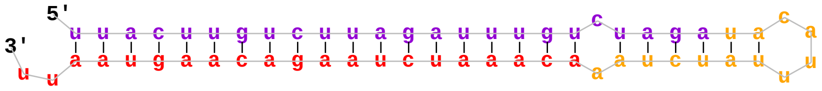

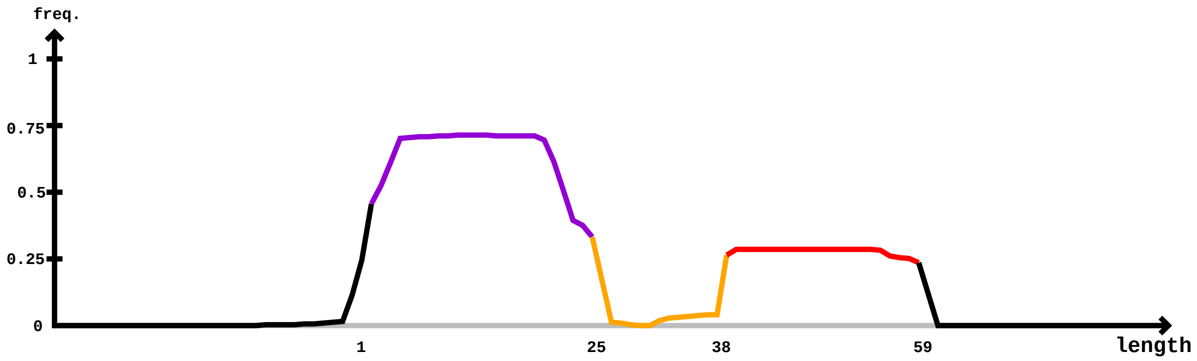

Star **Mature**

```
5'- auaauacgcuaguauaguccucuguccugaauuacuugucuuagauuugucuagauacauuuaucuaaacaaaucuaagacaaguaauuuaauuuuaggacgcagguaauauua  -3'  obs
    auaauacgcuaguauaguccucuguccugaauuacuugucuuagauuugucuagauacauuuaucuaaacaaaucuaagacaaguaauuuaauuuuaggacgcagguaauauua       exp
    ...((((....))))...(((.((((((((((((((((((((((((((((.((((((....)))))).))))))))))))))))))))......)))))))).)))........  reads  mm  sample
    ....................ucuguccugaauuacuugucuuag......................................................................  1  0  S05
    ........................uccugaauuacuugucuuagauuuguc...............................................................  1  0  S03
    ..........................cugaauuacuugucuuagauuug.................................................................  1  0  S04
    ...........................ugaauuacuugucuuagauuuguc...............................................................  1  0  S01
    ............................gaauuacuugucuuagauuugucu..............................................................  1  0  S06
    .............................aauuacuugucuuagauuug.................................................................  3  0  S03
    .............................aauuacuugucuuagauuugu................................................................  5  0  S02
    .............................aauuacuugucuuagauuugu................................................................  7  0  S05
    .............................aauuacuugucuuagauuugu................................................................  2  0  S03
    .............................aauuacuugucuuagauuugu................................................................  1  0  S01
    .............................aauuacuugucuuagauuugu................................................................  1  0  S06
    .............................aauuacuugucuuagauuugu................................................................  7  0  S04
    .............................aauuacuugucuuagauuuguc...............................................................  1  0  S03
    .............................aauuacuugucuuagauuuguc...............................................................  1  0  S01
    .............................aauuacuugucuuagauuugucu..............................................................  1  0  S01
    .............................aauuacuugucuuagauuugucu..............................................................  1  0  S04
    .............................aauuacuugucuuagauuugucua.............................................................  1  0  S04
    .............................aauuacuugucuuagauuugucua.............................................................  1  0  S02
    ..............................auuacuugucuuagauuug.................................................................  1  0  S05
    ..............................auuacuugucuuagauuugu................................................................  1  0  S06
    ..............................auuacuugucuuagauuuguc...............................................................  3  0  S04
    ..............................auuacuugucuuagauuuguc...............................................................  3  0  S06
    ..............................auuacuugucuuagauuuguc...............................................................  2  0  S03
    ..............................auuacuugucuuagauuuguc...............................................................  4  0  S02
    ..............................auuacuugucuuagauuuguc...............................................................  2  0  S05
    ..............................auuacuugucuuagauuuguc...............................................................  5  0  S01
    ..............................auuacuugucuuagauuugucu..............................................................  1  0  S05
    ..............................auuacuugucuuagauuugucuag............................................................  1  0  S03
    ..............................auuacuugucuuagauuugucuag............................................................  5  0  S04
    ..............................auuacuugucuuagauuugucuag............................................................  1  0  S06
    ..............................auuacuugucuuagauuugucuag............................................................  2  0  S01
    ..............................auuacuugucuuagauuugucuag............................................................  3  0  S05
    ..............................auuacuugucuuagauuugucuaga...........................................................  2  0  S04
```

Star Mature

```
auaauacgcuaguauaguccucuguccugaauuacuugucuuagauuugucuagauacauuuaucuaaacaaaucuaagacaaguaauuuaauuuuaggacgcagguaauauua
..............................auuacuugucuuagauuugucuaga...........................................................	1	0	S01
..............................auuacuugucuuagauuugucuaga...........................................................	1	0	S02
..............................auuacuugucuuagauuugucuaga...........................................................	1	0	S05
..............................auuacuugucuuagauuugucuaga...........................................................	3	0	S06
...............................uuacuugucuuagauuugu................................................................	1	0	S05
...............................uuacuugucuuagauuugu................................................................	1	0	S04
...............................uuacuugucuuagauuuguc...............................................................	2	0	S04
...............................uuacuugucuuagauuuguc...............................................................	2	0	S02
...............................uuacuugucuuagauuuguc...............................................................	2	0	S01
...............................uuacuugucuuagauuuguc...............................................................	1	0	S06
...............................uuacuugucuuagauuuguc...............................................................	3	0	S05
...............................uuacuugucuuagauuugucu..............................................................	6	0	S05
...............................uuacuugucuuagauuugucu..............................................................	4	0	S01
...............................uuacuugucuuagauuugucu..............................................................	3	0	S04
...............................uuacuugucuuagauuugucu..............................................................	2	0	S06
...............................uuacuugucuuagauuugucu..............................................................	4	0	S03
...............................uuacuugucuuagauuugucu..............................................................	2	0	S02
...............................uuacuugucuuagauuugucuU.............................................................	1	1	S06
...............................uuacuugucuuagauuugucuU.............................................................	1	1	S04
...............................uuacuugucuuagauuugucuaga...........................................................	3	0	S01
...............................uuacuugucuuagauuugucuaga...........................................................	3	0	S02
...............................uuacuugucuuagauuugucuaga...........................................................	2	0	S06
...............................uuacuugucuuUgauuugucuaga...........................................................	1	1	S03
...............................uuacuugucuuagauuugucuaga...........................................................	2	0	S03
...............................uuacuugucuuagauuugucuaga...........................................................	13	0	S04
...............................uuacuugucuuagauuugucuaga...........................................................	9	0	S05
................................uacuugucuuagauuuguc...............................................................	1	0	S06
................................uacuugucuuagauuugucu..............................................................	1	0	S03
................................uacuugucuuagauuugucu..............................................................	2	0	S04
................................uacuugucuuagauuugucu..............................................................	4	0	S01
................................uacuugucuuagauuugucu..............................................................	1	0	S02
................................uacuugucuuagauuugucua.............................................................	1	0	S01
................................uacuugucuuagauuugucua.............................................................	1	0	S05
................................uacuugucuuagauuugucuaga...........................................................	4	0	S04
................................uacuugucuuagauuugucuaga...........................................................	2	0	S06
................................uacuugucuuagauuugucuaga...........................................................	1	0	S01
................................uacuugucuuagauuugucuaga...........................................................	2	0	S05
................................uacuugucuuagauuugucuaga...........................................................	2	0	S02
.................................acuugucuuagauuuguc...............................................................	1	0	S04
.................................acuugucuuagauuugucu..............................................................	1	0	S04
.................................acuugucuuagauuugucu..............................................................	1	0	S05
.................................acuugucuuagauuugucuag............................................................	1	0	S04
.................................acuugucuuagauuugucuaga...........................................................	5	0	S01
.................................acuugucuuagauuugucuaga...........................................................	8	0	S05
.................................acuugucuuagauuugucuaga...........................................................	5	0	S06
.................................acuugucuuagauuugucuaga...........................................................	2	0	S04
.................................acuugucuuagauuugucuaga...........................................................	1	0	S02
.................................acuugucuuagauuugucuaga...........................................................	2	0	S03
.................................acuugucuuagauuugucuagaua.........................................................	1	0	S04
..................................cuugucuuagauuugucu..............................................................	1	0	S04
..................................cuugucuuagauuugucuag............................................................	1	0	S01
..................................cuugucuuagauuugucuaga...........................................................	7	0	S04
..................................cuugucuuagauuugucuaga...........................................................	2	0	S01
..................................cuugucuuagauuugucuaga...........................................................	6	0	S05
..................................cuugucuuagauuugucuaga...........................................................	4	0	S03
..................................cuugucuuagauuugucuaga...........................................................	4	0	S06
..................................cuugucuuagauuugucuaga...........................................................	4	0	S02
...................................uugucuuagauuugucuaga...........................................................	1	0	S03
....................................ugucuuagauuugucuagauac........................................................	1	0	S02
......................................ucuuagauuugucuagauaca.......................................................	1	0	S05
........................................uuagauuugucuagauac........................................................	1	0	S04
.............................................................uaucuaaacaaaucuaagacaag..............................	1	0	S03
.............................................................uaucuaaacaaaucuaagacaagA.............................	1	1	S06
.............................................................uaucuaaacaaaucuaagacaagA.............................	1	1	S04
.............................................................uaucuaaacaaaucuaagacaagA.............................	1	1	S01
.............................................................uaucuaaacaaaucuaagacaagu.............................	1	0	S01
.............................................................uaucuaaacaaaucuaagacaagA.............................	1	1	S03
..............................................................aucuaaacaaaucuaagacaagA.............................	1	1	S04
..............................................................aucuaaacaaaucuaagacaagua............................	1	0	S02
..............................................................aucuaaacaaaucuaagacaagua............................	1	0	S04
```

```
                              Star                                 Mature
auaauacgcuaguauaguccucuguccugaauuacuugucuuagauuugucuagauacauuuaucuaaacaaaucuaagacaaguaauuuaauuuuaggacgcagguaauauua
...............................................................ucuaaacaaaucuaagacaagA.............................   1   1   S02
................................................................cuaaacaaaucuaagacaaguaauu.........................   1   0   S05
.................................................................uaaacaaaucuaagacaaguaauu.........................   1   0   S01
..................................................................aaaAaaaucuaagacaaguaauu.........................   1   1   S04
....................................................................acaaaucuaagacaaguaa...........................   1   0   S01
....................................................................acaaaucuaagacaaguaau..........................   1   0   S03
....................................................................acaaaucuaagacaaguaau..........................   2   0   S01
....................................................................acaaaucuaagacaaguaau..........................   1   0   S05
....................................................................acaaaucuaagacaaguaauu.........................   4   0   S06
....................................................................acaaaucuaagacaaguaauu.........................  17   0   S05
....................................................................acaaaucuaagacaaguaauu.........................  12   0   S04
....................................................................acaaaucuaagacaaguaauu.........................  21   0   S01
....................................................................acaaaucuaagacaaguaauu.........................  11   0   S02
....................................................................acaaaucuaagacaaguaauu.........................   2   0   S03
.....................................................................caaaucuaagacaaguaau..........................   1   0   S02
.....................................................................caaaucuaagacaaguaauu.........................   3   0   S01
.....................................................................caaaucuaagacaaguaauu.........................   1   0   S04
.....................................................................caaaucuaagacaaguaauuu........................   1   0   S01
.....................................................................caaaucuaagacaaguaauuu........................   1   0   S05
```

Provisional ID	: ta_iwgsc_2bl_v1_7950530_1378157	
Score total	:	1.9
Score for star read(s)	:	-1.3
Score for read counts	:	0
Score for mfe	:	1.6
Score for randfold	:	1.6
Score for cons. seed	:	
Total read count	:	185
Mature read count	:	331
Loop read count	:	0
Star read count	:	39

novel-m0430-3p

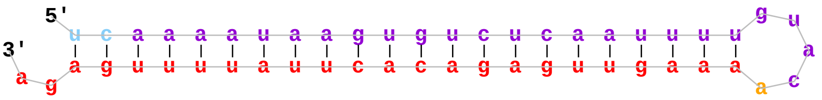

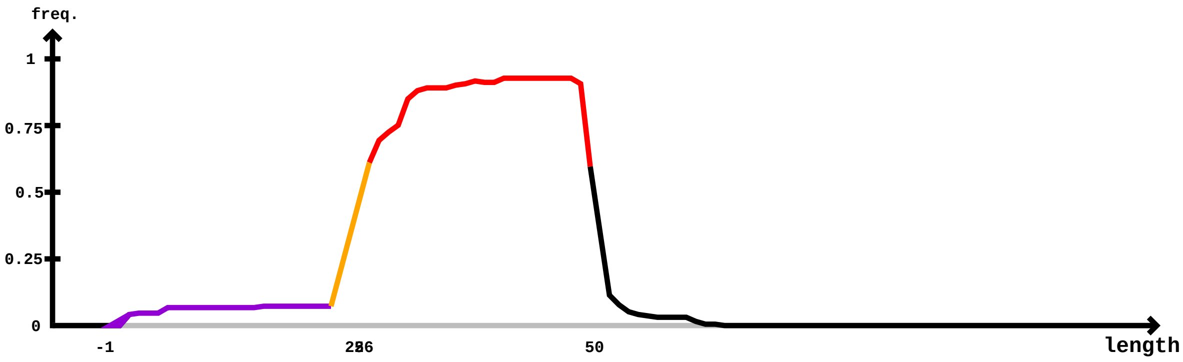

Star **Mature**

```
5'- ugucucaaaauaagugucucaauuuuguacaaaaguugagacacuuauuuugagacggagagaguauuauaugauuuuaaaacaagaaaacacgauauauuauaaaauagaau   -3'   obs
    ugucucaaaauaagugucucaauuuuguacaaaaguugagacacuuauuuugagacggagagaguauuauaugauuuuaaaacaagaaaacacgauauauuauaaaauagaau         exp
    ((((((((((((((((((((((((((.....))))))))))))))))))))))))))).......((((.((((((.......................)))))).))))....   reads   mm   sample
    ......aaaauaagugucucaauuuuguac...................................................................................   3   0   S04
    ......aaaauaagugucucaauuuuguac...................................................................................   1   0   S02
    ......aaaauaagugucucaauuuuguac...................................................................................   1   0   S03
    ......aaaauaagugucucaauuuuguac...................................................................................   2   0   S05
    ......aaaauaagugucucaauuuuguac...................................................................................   1   0   S06
    .......aaauaagugucucaauuuuguac...................................................................................   1   0   S02
    ..........uaagugucucaauuuuguac...................................................................................   2   0   S05
    ..........uaagugucucaauuuuguac...................................................................................   1   0   S03
    ..........uaagugucucaauuuuguac...................................................................................   1   0   S04
    ....................aauuuuguacaaaaguugagaca......................................................................   1   0   S02
    ..............................aaaaguugagacacuuauuuuga............................................................   1   0   S05
    ..............................aaaaguugagacacuuauuuugag...........................................................   3   0   S04
    ..............................aaaaguugagUcacuuauuuugag...........................................................   1   1   S05
    ..............................aaaaguugagacacuuauuuugaga..........................................................   1   0   S04
    ...............................aaaguugagacacuuauuuugag...........................................................   1   0   S01
    ...............................aaaguugagacacuuauuuugag...........................................................   3   0   S06
    ...............................aaaguugagacacuuauuuugag...........................................................   17  0   S04
    ...............................aaUguugagacacuuauuuugag...........................................................   1   1   S01
    ...............................aaUguugagacacuuauuuugag...........................................................   1   1   S04
    ...............................aaaguugagacacuuauuuugag...........................................................   4   0   S02
    ...............................aaaUuugagacacuuauuuugag...........................................................   1   1   S04
    ...............................aaaguugagacacuuauuuugag...........................................................   14  0   S05
    ...............................aaaguugagacacuuauuuugag...........................................................   2   0   S03
    ...............................aaaguCgagacacuuauuuugaga..........................................................   1   1   S06
    ...............................aaaguugagacacuuauuuugaga..........................................................   3   0   S03
    ...............................aaUguugagacacuuauuuugaga..........................................................   1   1   S05
    ...............................aaaguugagacacuuauuuugaga..........................................................   4   0   S01
    ...............................aaaguugagacacuuauuuugaga..........................................................   19  0   S05
    ...............................aaaguugagacacuuauuuugaga..........................................................   6   0   S02
    ...............................aaaguugagacacuuauuuugaga..........................................................   1   0   S06
    ...............................aaaguugagacacuuauuuugaga..........................................................   30  0   S04
    ...............................aaaguugagacacuuauuuugagac.........................................................   1   0   S05
    ...............................aaaguugagacacuuauuuugagac.........................................................   1   0   S02
```

Star **Mature**

ugucucaaaauaagugucucaauuuuguacaaaaguugagacacuuauuuugagacggagagaguauuauaugauuuuaaaacaagaaaacacgauauauuauaaaauagaau			
................................aaguugagacacuuauuuuga..	1	0	S04
................................aaguugagacacuuauuuugag...	1	0	S05
................................aaguugagacacuuauuuugag...	1	0	S02
................................aaguugagacacuuauuuugag...	1	0	S01
................................aaguugagacacuuauuuugaga..	2	0	S04
................................aaguugagacacuuauuuugaga..	2	0	S03
................................aaguugagacacuuauuuugaga..	1	0	S02
................................aaguugagacacuuauuuugaga..	1	0	S05
................................aaguugagacacuuauuuugaga..	1	0	S01
................................aaguugagacacuuauuuugagac...	3	0	S05
................................aaguugagacacuuauuuugagac...	2	0	S04
.................................aguugagacacuuauuuugagac...	1	0	S04
.................................aguugagacacuuauuuugagacg..	2	0	S04
.................................aguugagacacuuauuuugagacg..	1	0	S05
.................................aguugagacacuuauuuugUgacg..	1	1	S04
.................................aguugagacacuuauuuugagacgga..	1	0	S05
..................................guugagacacuuauuuugagacg..	1	0	S04
..................................guugagacacuuauuuugagacg..	1	0	S05
..................................guugagacacuuauuuugagacgg...	1	0	S04
..................................guugagacacuuauuuugagacgg...	2	0	S05
...................................uugagacacuuauuuuga..	1	0	S01
...................................uugagacacuuauuuuga..	1	0	S04
...................................uugagacacuuauuuugag...	3	0	S04
...................................uugagacacuuauuuugag...	4	0	S01
...................................uugagacacuuauuuugag...	1	0	S05
...................................uugagacacuuauuuugaga..	1	0	S04
...................................uugagacacuuauuuugaga..	1	0	S05
...................................uugagacacuuauuuugaga..	2	0	S01
...................................uugagacacuuauuuugagac...	2	0	S04
...................................uugagacacuuauuuugagac...	1	0	S01
...................................uugagacacuuauuuugagacgg...	1	0	S04
...................................uugagacacuuauuuugagacgga..	1	0	S05
....................................ugagacacuuauuuugag...	1	0	S01
....................................ugagacacuuauuuugagac...	1	0	S05
....................................ugagacacuuauuuugagac...	3	0	S02
....................................ugagacacuuauuuugagacg..	1	0	S05
.....................................gagacacuuauuuugaga..	1	0	S05
.....................................gagacaUuuauuuugagacggaga..	1	1	S06
....acacuuauuuugagacgg...	1	0	S03
....acacuuauuuugagacggag...	1	0	S03
....cacuuauuuugagacggagagagu..	1	0	S04
....acuuUuuugagacggagagagua..	2	1	S04
....uauuuugagacggagagagu..	2	0	S02
....uauuuugagacggagagaguauu...	1	0	S04

Provisional ID	:	ta_iwgsc_2bl_v1_7964427_1395889
Score total	:	615.3
Score for star read(s)	:	3.9
Score for read counts	:	607.8
Score for mfe	:	1.9
Score for randfold	:	1.6
Score for cons. seed	:	
Total read count	:	1204
Mature read count	:	555
Loop read count	:	0
Star read count	:	370

novel-m0121-3p

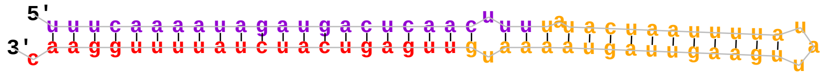

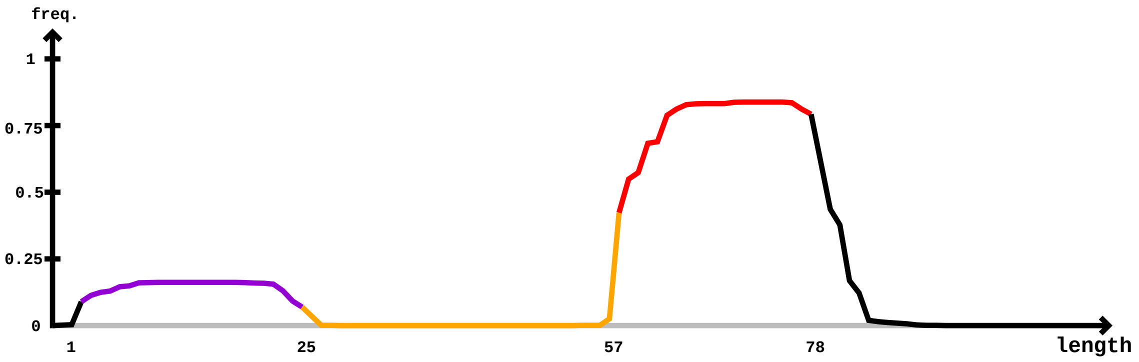

Star **Mature**

```
5'- guuucaaaauagaugacucaacuuuuauacuaauuuuauauugaaguuaguaaaauguugagucaucuauuuuggaacggaggaaguagaacuuuauguaaagaaguu  -3'   obs
    guuucaaaauagaugacucaacuuuuauacuaauuuuauauugaaguuaguaaaauguugagucaucuauuuuggaacggaggaaguagaacuuuauguaaagaaguu         exp
    ((((((((((((((((((((.(((.((((((((((((...))))))))))))).))))))))))))))))))))))...........((((((.......))))))  reads   mm   sample
    guuucaaaauagaugacu..........................................................................................  1       0    S01
    guuucaaaauagaugacucaacu.....................................................................................  2       0    S05
    guuucaaaauagaugacucaacuu....................................................................................  1       0    S04
    .uuucaaaauagaugacuc.........................................................................................  1       0    S05
    .uuucaaaauagaugacuc.........................................................................................  1       0    S06
    .uuucaaaauagaugacuca........................................................................................  1       0    S01
    .uuucaaaauagaugacucaa.......................................................................................  2       0    S01
    .uuucaaaauagaugacucaac......................................................................................  3       0    S01
    .uuucaaaauagaugacucaac......................................................................................  4       0    S06
    .uuucaaaauagaugacucaac......................................................................................  6       0    S05
    .uuucaaaauagaugacucaac......................................................................................  7       0    S04
    .uuucaaaauagaugacucaac......................................................................................  2       0    S03
    .uuucaaaauagaugacucaac......................................................................................  5       0    S02
    .uuucaaaauagaugacucaacu.....................................................................................  5       0    S04
    .uuucaaaauagaugacucaacu.....................................................................................  1       0    S06
    .uuucaaaauagaugacucaacu.....................................................................................  4       0    S01
    .uuucaaaauagaugacucaacu.....................................................................................  1       0    S03
    .uuucaaaauagaugacucaacu.....................................................................................  6       0    S05
    .uuucaaaauagaugacucaacu.....................................................................................  1       0    S02
    .uuucaaaauagaugacucaacuu....................................................................................  5       0    S04
    .uuucaaaauagaugacucaacuu....................................................................................  6       0    S05
    .uuucaaaauagaugacucaacuu....................................................................................  3       0    S02
    .uuucaaaauagaugacucaacuu....................................................................................  2       0    S01
    .uuucaaaauagaugacucaacuuu...................................................................................  11      0    S01
    .uuucaaaauagaugacucaacuuu...................................................................................  2       0    S06
    .uuucaaaauagaugacucaacuuu...................................................................................  4       0    S02
    .uuucaaaauagaugacucaacuuu...................................................................................  9       0    S04
    .uuucaaaauagaugacucaacuuu...................................................................................  14      0    S05
    ..uucaaaauagaugacucaa.......................................................................................  2       0    S05
    ..uucaaaauagaugacucaac......................................................................................  1       0    S05
    ..uucaaaauagaugacucaac......................................................................................  1       0    S03
    ..uucaaaauagaugacucaac......................................................................................  1       0    S02
    ..uucaaaauagaugacucaacu.....................................................................................  6       0    S05
```

Star **Mature**

guuucaaaauagaugacucaacuuuuauacuaauuuuauauugaaguuaguaaaauguugagucaucuauuuuggaacggaggaaguagaacuuuauguaaagaaguu			
..uucaaaauagaugacucaacu...	2	0	S01
..uucaaaauagaugacucaacu...	2	0	S04
..uucaaaauagaugacucaacu...	1	0	S06
..uucaaaauagaugacucaacu...	1	0	S03
..uucaaaauagaugacucaacu...	1	0	S02
..uucaaaauagaugacucaacuu..	1	0	S04
..uucaaaauagaugacucaacuu..	1	0	S01
..uucaaaauagaugacucaacuu..	1	0	S02
..uucaaaauagaugacucaacuuu...	2	0	S01
..uucaaaauagaugacucaacuuu...	1	0	S02
..uucaaaauagaugacucaacuuu...	2	0	S05
..uucaaaauagaugacucaacuuu...	2	0	S04
..uucaaaauagaugacucaacuuu...	1	0	S03
...ucaaaauagaugacucaac..	1	0	S01
...ucaaaauagaugacucaacu...	1	0	S01
...ucaaaauagaugacucaacu...	3	0	S02
...ucaaaauagaugacucaacuu..	1	0	S06
...ucaaaauagaugacucaacuu..	2	0	S02
...ucaaaauagaugacucaacuu..	1	0	S03
...ucaaaauagaugacucaacuuu...	1	0	S02
...ucaaaauagaugacucaacuuu...	2	0	S01
...ucaaaauagaugacucaacuuu...	1	0	S05
...ucaaaauagaugacucaacuuu...	1	0	S04
....caaaauagaugacucaacu...	2	0	S01
....caaaauagaugacucaacuu..	1	0	S06
....caaaauagaugacucaacuuu...	2	0	S01
....caaaauagaugacucaacuuu...	1	0	S05
.....aaaauagaugacucaacu...	1	0	S05
.....aaaauagaugacucaacu...	1	0	S01
.....aaaauagaugacucaacu...	1	0	S04
.....aaaauagaugacucaacu...	5	0	S03
.....aaaauagaugacucaacuu..	2	0	S05
.....aaaauagaugacucaacuuu...	4	0	S06
.....aaaauagaugacucaacuuu...	1	0	S01
.....aaaauagaugacucaacuuu...	2	0	S05
.....aaaauagaugacucaacuuu...	1	0	S04
.....aaaauagaugacucaacuuu...	2	0	S03
......aaauagaugacucaacuu..	1	0	S03
......aaauagaugacucaacuuu...	1	0	S04
......aaauagaugacucaacuuu...	1	0	S05
......aaauagaugacucaacuuu...	1	0	S06
.......aauagaugacucaacuuu...	2	0	S04
.......aauagaugacucaacuuu...	1	0	S02
.......aauagaugacucaacuuu...	5	0	S06
.......aauagaugacucaacuuu...	1	0	S05
.......aauagaugacucaacuuu...	3	0	S01
.......aauagaugacucaacuuuu..	2	0	S05
........auagaugacucaacuuuu..	1	0	S03
.........uagaugacucaacuuuGau..	1	1	S05
....aauguugagucaucuauuuugga................................	1	0	S02
....guugagucaucuauuuugg.................................	1	0	S05
....guugagucaucuauuuuggaac..............................	2	0	S01
....guugagucaucuauuuuggaacg.............................	2	0	S06
....guugagucaucuauuuuggaacg.............................	4	0	S05
....guugagucaucuauuuuggaacg.............................	2	0	S03
....guugagucaucuauuuuggaacg.............................	5	0	S04
....guugagucaucuauuuuggaacg.............................	1	0	S01
....guugagucaucuauuuuggaacgg............................	3	0	S04
....guugagucaucuauuuuggaacgg............................	1	0	S02
....guugagucaucuauuuuggaacgg............................	2	0	S01
....guugagucaucuauuuuggaacgg............................	4	0	S05
....guugaUucaucuauuuuggaacgg............................	1	1	S02
....guugagucaucuauuuuggaacgg............................	1	0	S03
....guugagucaucuauuuuggaacgga...........................	1	0	S05
....uugagucaucuauuuugg.................................	1	0	S06
....uugagucaucuauuuugg.................................	1	0	S02
....uugagucaucuauuuugga................................	8	0	S01
....uugagucaucuauuuugga................................	4	0	S03
....uugagucaucuauuuugga................................	2	0	S04
....uugagucaucuauuuugga................................	6	0	S06

```
 Star                                                    Mature
guuucaaaauagaugacucaacuuuuauacuaauuuuauauugaaguuaguaaaauguugagucaucuauuuuggaacggaggaaguagaacuuuauguaaagaaguu
.........................................................uugagucaucuauuuugga................................ 5 0 S05
.........................................................uugagucaucuauuuuggaU............................... 1 1 S01
.........................................................uugagucaucuauuuuggaa............................... 8 0 S02
.........................................................uugagucaucuauuuuggaa............................... 4 0 S01
.........................................................uugagucaucuauuuuggaa............................... 5 0 S04
.........................................................uugagucaucuauuuuggaa............................... 2 0 S06
.........................................................uugagucaucuauuuuggaa............................... 3 0 S03
.........................................................uugagucaucuauuuuggaa............................... 1 0 S05
.........................................................uugagucaucuauuuuggaac.............................. 28 0 S03
.........................................................uugagucaucuauuuuggaac.............................. 39 0 S02
.........................................................uugagucaucuauuuuggaac.............................. 41 0 S06
.........................................................uugagucaucuauuuuggaac.............................. 48 0 S04
.........................................................uugagucaucuauuuuggaac.............................. 65 0 S01
.........................................................uugagucaucuauuuuggaac.............................. 69 0 S05
.........................................................uugagucaucuauuuuggaacg............................. 1 0 S01
.........................................................uugagucaucuauuuuggaacg............................. 1 0 S03
.........................................................uugagucaucuauuuuggaacg............................. 1 0 S02
.........................................................uugagucaucuauuuuggaacg............................. 2 0 S05
.........................................................uugagucaucuauuuuggaacg............................. 3 0 S06
.........................................................uugagucaucuauuuuggaacg............................. 3 0 S04
.........................................................uugagucaucuauuuuggaacgg............................ 6 0 S02
.........................................................uugagucaucuauuuuggaacgg............................ 5 0 S06
.........................................................uugagucaucuauuuuggaacgg............................ 1 0 S01
.........................................................uugagucaucuauuuuggaacgg............................ 6 0 S04
.........................................................uugagucaucuauuuuggaacgg............................ 4 0 S05
.........................................................uugagucaucuauuuuggaacgg............................ 5 0 S03
.........................................................uugagucaucuauuuuggaacgga........................... 12 0 S01
.........................................................uugagucaucuauuuuggaacgga........................... 32 0 S05
.........................................................uugagucaucuauuuuggUacgga........................... 1 1 S05
.........................................................uugagucaucuauuuuggaacgga........................... 31 0 S04
.........................................................uugagucaucuauuuuggaacgga........................... 16 0 S06
.........................................................uugagucaucuauuuuggaacgga........................... 12 0 S02
.........................................................uugagucaucuauuuuggaacgga........................... 5 0 S03
.........................................................uugagucaucuauuAuggaacgga........................... 1 1 S01
..........................................................ugagucaucuauuuugga................................ 1 0 S01
..........................................................ugagucaucuauuuugga................................ 1 0 S05
..........................................................ugagucaucuauuuugga................................ 1 0 S04
..........................................................ugagucaucuauuuuggaac.............................. 5 0 S02
..........................................................ugagucaucuauuuuggaac.............................. 3 0 S01
..........................................................ugagucaucuauuuuggaac.............................. 3 0 S05
..........................................................ugagucaucuauuuuggaac.............................. 2 0 S03
..........................................................ugagucaucuauuuuggaac.............................. 4 0 S06
..........................................................ugagucaucuauuuuggaac.............................. 1 0 S04
..........................................................ugagucaucuauuuuggaacg............................. 14 0 S03
..........................................................ugagucaucuauuuuggaacg............................. 15 0 S02
..........................................................ugagucaucuauuuuggaacg............................. 19 0 S06
..........................................................ugagucaucuauuuuggaacg............................. 18 0 S01
..........................................................ugagucaucuauuuuggaacg............................. 21 0 S05
..........................................................ugagucaucuauuuuggaacg............................. 13 0 S04
..........................................................ugagucaucuauuuuggaacgg............................ 1 0 S02
..........................................................ugagucaucuauuuuggaacgg............................ 2 0 S04
..........................................................ugagucaucuauuuuggaacgg............................ 1 0 S06
..........................................................ugagucaucuauuuuggaacgga........................... 2 0 S01
..........................................................ugagucaucuauuuuggaacgga........................... 3 0 S04
..........................................................ugagucaucuauuuuggaacgga........................... 5 0 S05
..........................................................ugagucaucuauuuuggaacgga........................... 2 0 S02
..........................................................ugagucaucuauuuuggaacgga........................... 3 0 S06
..........................................................ugagucaucuauuuuggaacggag.......................... 1 0 S02
..........................................................ugagucaucuauuuuggaacggag.......................... 4 0 S05
..........................................................ugagucaucuauuuuggaacggag.......................... 7 0 S04
..........................................................ugagucaucuauuuuggaacggag.......................... 3 0 S06
...........................................................gagucaucuauuuuggaacgg............................ 1 0 S03
...........................................................gagucaucuauuuuggaacgga........................... 1 0 S05
...........................................................gagucaucuauuuuggaacgga........................... 1 0 S06
...........................................................gagucaucuauuuuggaacgga........................... 1 0 S02
...........................................................gagucaucuauuuuggaacggag.......................... 1 0 S04
...........................................................gagucaucuauuuuggaacggagg......................... 4 0 S03
...........................................................gagucaucuauuuuggaacggUgg......................... 1 1 S05
...........................................................gagucaucuauuuuggaacggagg......................... 1 0 S02
...........................................................gagucaucuauuuuggaacggagg......................... 8 0 S06
```

Star **Mature**

guuucaaaauagaugacucaacuuuuauacuaauuuuauauugaaguuaguaaaauguugagucaucuauuuuggaacggaggaaguagaacuuuauguaaagaaguu			
....gagucaucuauuuuggaacggagg.........................	7	0	S05
....gagucaucuauuuuggaacggagg.........................	4	0	S04
....agucaucuauuuuggaac..............................	1	0	S03
....agucaucuauuuuggaacg.............................	1	0	S03
....agucaucuauuuuggaacgg............................	1	0	S04
....agucaucuauuuuggaacgg............................	1	0	S01
....agucaucuauuuuggaacgg............................	4	0	S02
....agucaucuauuuuggaacgg............................	2	0	S06
....agucaucuauuuuggaacgg............................	2	0	S05
....agucaucuauuuuggaacgg............................	10	0	S03
....agucaucuauuuuggaacgga...........................	18	0	S01
....agucaucuauuuuggaacgga...........................	14	0	S04
....agucaucuauuuuggaacgga...........................	9	0	S03
....agucaucuauuuuggaacgga...........................	22	0	S06
....agucaucuauuuuggaacgga...........................	24	0	S02
....agucaucuauuuuggaacgga...........................	20	0	S05
....agucaucuauuuuggaacggag..........................	1	0	S05
....agucaucuauuuuggaacggagg.........................	1	0	S06
....agucaucuauuuuggaacggagg.........................	1	0	S05
....agucaucuauuuuggaacggagga........................	1	0	S04
....agucaucuauuuuggaacCgagga........................	1	1	S05
....agucaucuauuuuggaacggagga........................	1	0	S05
....gucaucuauuuuggaacg.............................	1	0	S05
....gucaucuauuuuggaacgga...........................	2	0	S04
....gucaucuauuuuggaacgga...........................	1	0	S06
....gucaucuauuuuggaacggag..........................	2	0	S06
....gucaucuauuuuggaacggag..........................	1	0	S01
....ucaucuauuuuggaacgg............................	1	0	S01
....ucaucuauuuuggaacgg............................	1	0	S02
....ucaucuauuuuggaacgg............................	3	0	S06
....ucaucuauuuuggaacgg............................	2	0	S05
....ucaucuauuuuggaacgg............................	1	0	S03
....ucaucuauuuuggaacgga...........................	1	0	S06
....ucaucuauuuuggaacgga...........................	1	0	S04
....ucaucuauuuuggaacgga...........................	5	0	S01
....ucaucuauuuuggaacgga...........................	2	0	S02
....ucaucuauuuuggaacgga...........................	2	0	S03
....ucaucuauuuuggaacggag..........................	2	0	S05
....ucaucuauuuuggaacggag..........................	2	0	S01
....ucaucuauuuuggaacggag..........................	3	0	S04
....ucaucuauuuuggaacggag..........................	3	0	S06
....ucaucuauuuuggaacggag..........................	8	0	S03
....ucaucuauuuuggaacggag..........................	9	0	S02
....ucaucuauuuuggaacggagg.........................	19	0	S05
....ucaucuauuuuggaacggagg.........................	5	0	S03
....ucaucuauuuuggaacggagg.........................	17	0	S01
....ucaucuauuuuggaacggagg.........................	6	0	S06
....ucaucuauuuuggaacggagg.........................	14	0	S04
....ucaucuauuuuggaacggagg.........................	15	0	S02
....caucuauuuuggaacgga...........................	1	0	S03
....caucuauuuuggaacgga...........................	1	0	S04
....caucuauuuuggaacgga...........................	1	0	S05
....caucuauuuuggaacgga...........................	1	0	S01
....caucuauuuuggaacgga...........................	3	0	S06
....caucuauuuuggaacggag..........................	1	0	S06
....caucuauuuuggaacggag..........................	1	0	S05
....caucuauuuuggaacggag..........................	1	0	S03
....caucuauuuuggaacggagg.........................	1	0	S03
....caucuauuuuggaacggagg.........................	2	0	S02
....caucuauuuuggaacggagg.........................	3	0	S01
....caucuauuuuggaacggagg.........................	10	0	S06
....caucuauuuuggaacggaggaag......................	1	0	S03
....caucuauuuuggaacggaggaag......................	1	0	S04
....caucuauuuuggaacggaggaag......................	1	0	S05
....aucuauuuuggaacggag..........................	3	0	S06
....aucuauuuuggaacggag..........................	3	0	S05
....aucuauuuuggaacggag..........................	1	0	S04
....aucuauuuuggaacggagg.........................	2	0	S02
....aucuauuuuggaacggagg.........................	2	0	S06
....aucuauuuuggaacggagg.........................	2	0	S04

Star Mature

guuucaaaauagaugacucaacuuuuauacuaauuuuauauugaaguuaguaaaauguugagucaucuauuuuggaacggaggaaguagaacuuuauguaaagaaguu

....aucuauuuuggaacggagga........................	1	0	S01
....aucuauuuuggaacggagga........................	1	0	S05
....aucuauuuuggaacggagga........................	1	0	S03
....aucuauuuuggaacggaggaa.......................	1	0	S02
....aucuauuuuggaacggaggaa.......................	1	0	S05
....aucuauuuuggaacggaggaa.......................	1	0	S01
....aucuauuuuggaacggaggaagu.....................	1	0	S01
....ucuauuuuggaacggagg.........................	1	0	S04
....ucuauuuuggaacggaggaa.......................	1	0	S03
....ucuauuuuggaacggaggaagua....................	2	0	S04
....cuauuuuggaacggaggaagu.....................	1	0	S04
....uuuuggaacggaggaagu.....................	1	0	S04
....uuuuggaacggaggaaguC....................	2	1	S04
....uuuuggaacggaggaagua....................	1	0	S04
....uuuuggaacggaggaaguag...................	1	0	S03
....uuuuggaacggaggaaguag...................	1	0	S05
....uuugAaacggaggaaguagaa.................	1	1	S03

Provisional ID	:	ta_iwgsc_2bl_v1_7975940_1409761
Score total	:	135.2
Score for star read(s)	:	3.9
Score for read counts	:	127.1
Score for mfe	:	2.6
Score for randfold	:	1.6
Score for cons. seed	:	
Total read count	:	261
Mature read count	:	273
Loop read count	:	1
Star read count	:	63

novel-m0049-3p

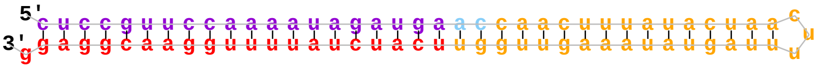

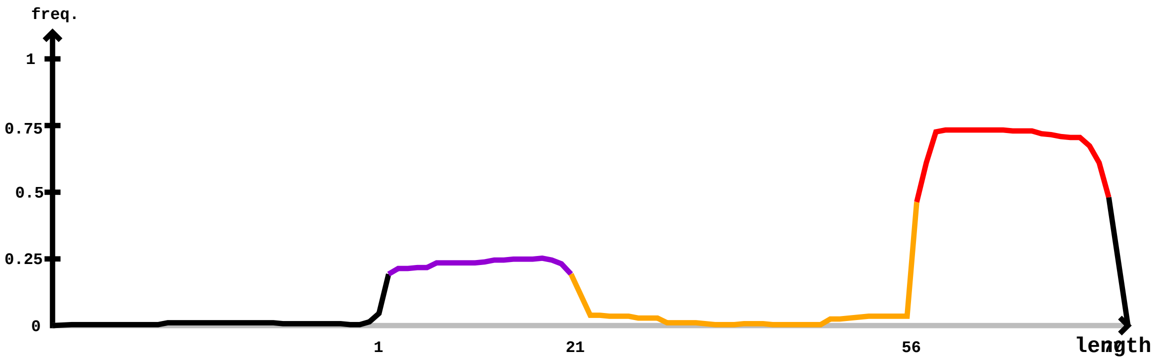


```
                                  Star                                                    Mature
5'- ccaaaauaggugacccaacuuuauacuaauucccuccguuccaaaauagaugaaccaacuuuauacuaacuuuuaguauaaaguugguucaucuauuuuggaacggaggg  -3'   obs
    ccaaaauaggugacccaacuuuauacuaauucccuccguuccaaaauagaugaaccaacuuuauacuaacuuuuaguauaaaguugguucaucuauuuuggaacggaggg        exp
    .......((((......))))..........((((((((((((((((((((((((((((((((((((((...))))))))))))))))))))))))))))))))))))))  reads  mm  sample
    ccaaaauaggugacccaacuuu................................................................................................  1  0  S05
    ..........ugacccaacuuuauacua..................................................................................  1  0  S01
    ..........ugacccaacuuuauacuaa.................................................................................  1  0  S03
    ............................auucccuccguuccaaaauagaug..........................................................  1  0  S04
    ...............................cccuccguuccaaaauagau...........................................................  1  0  S02
    ...............................cccuccguuccaaaauagaug..........................................................  1  0  S01
    ...............................cccuccguuccaaaauagauga.........................................................  1  0  S02
    ................................ccuccguuccaaaauaga............................................................  1  0  S04
    ................................ccuccguuccaaaauaga............................................................  1  0  S01
    ................................ccuccguuccaaaauagauga.........................................................  1  0  S02
    ................................ccuccguuccaaaauagauga.........................................................  2  0  S04
    ................................ccuccguuccaaaauagauga.........................................................  1  0  S03
    ................................ccuccguuccaaaauagauga.........................................................  2  0  S05
    ................................ccuccguuccaaaauagaugaacc......................................................  1  0  S03
    .................................cuccguuccaaaauagau...........................................................  1  0  S03
    .................................cuccguuccaaaauagau...........................................................  2  0  S02
    .................................cuccguuccaaaauagaug..........................................................  1  0  S04
    .................................cuccguuccaaaauagaug..........................................................  2  0  S03
    .................................cuccguuccaaaauagaug..........................................................  2  0  S06
    .................................cuccguuccaaaauagauga.........................................................  4  0  S04
    .................................cuccguuccaaaauagauga.........................................................  3  0  S01
    .................................cuccguuccaaaauagauga.........................................................  9  0  S03
    .................................cuccguuccaaaauagauga.........................................................  4  0  S02
    .................................cuccguuccaaaauagauga.........................................................  5  0  S05
    .................................cuccguuccaaaauagauga.........................................................  4  0  S06
    .................................cuccguuccaaaauagaugaa........................................................  1  0  S03
    .................................cuccguuccaaaauagaugaa........................................................  1  0  S04
    .................................cuccguuccaaaauagaugaa........................................................  2  0  S06
    .................................cuccguuccaaaauagaugaa........................................................  1  0  S01
    ..................................uccguuccaaaauagaug..........................................................  2  0  S03
    ..................................uccguuccaaaauagaug..........................................................  1  0  S04
    ..................................uccguuccaaaauagaug..........................................................  1  0  S01
    ..................................uccguuccaaaauagauga.........................................................  1  0  S04
```

```
                                Star                                                   Mature
ccaaaauaggugacccaacuuuauacuaauucccuccguuccaaaauagaugaaccaacuuuauacuaacuuuuaguauaaaguugguucaucuauuuuggaacggaggg
..................................uccguuccaaaauagaugaa........................................................   1    0    S04
....................................cguuccaaaauagaugaa........................................................   1    0    S05
......................................uuccaaaauagaugaaccaac...................................................   2    0    S06
......................................uuccaaaauagaugaaccaacuuC................................................   1    1    S02
......................................uuccaaaauagaugaaccaacuuu................................................   1    0    S04
......................................uuccaaaauagaugaaccaacuuu................................................   1    0    S06
..........................................aaaauagaugaaccaacuuuauac............................................   1    0    S05
...........................................aaauagaugaaccaacuuu................................................   2    0    S06
..............................................uagaugaaccaacuuuauacu...........................................   1    0    S01
.................................................augaaccaacuuuauacuaacuuu.....................................   1    0    S03
......................................................................uuuuaguauaaaguugguuc....................   1    0    S04
...............................................................................aaaguugguucaucuauuu............   1    0    S04
...............................................................................aaaguugguucaucuauuuugg.........   2    0    S01
...............................................................................aaaguugguucaucuauuuugg.........   1    0    S05
...............................................................................aaaguugguucaucuauuuuggaa.......   1    0    S04
...............................................................................aaaguugguucaucuauuuuggaa.......   1    0    S06
.................................................................................aguugguucaucuauuuugga........   1    0    S05
..................................................................................guugAuucaucuauuuuggaacgg....   1    1    S02
...................................................................................uugguucaucuauuuuggaac......   1    0    S04
........................................................................................ucaucuauuuuggaacgg....   3    0    S06
........................................................................................ucaucuauuuuggaacgg....   2    0    S05
........................................................................................ucaucuauuuuggaacgg....   1    0    S03
........................................................................................ucaucuauuuuggaacgg....   1    0    S02
........................................................................................ucaucuauuuuggaacgg....   1    0    S01
........................................................................................ucaucuauuuuggaacgga...   2    0    S02
........................................................................................ucaucuauuuuggaacgga...   1    0    S06
........................................................................................ucaucuauuuuggaacgga...   5    0    S01
........................................................................................ucaucuauuuuggaacgga...   1    0    S04
........................................................................................ucaucuauuuuggaacgga...   2    0    S03
........................................................................................ucaucuauuuuggaacggag..   2    0    S05
........................................................................................ucaucuauuuuggaacggag..   3    0    S06
........................................................................................ucaucuauuuuggaacggag..   3    0    S04
........................................................................................ucaucuauuuuggaacggag..   2    0    S01
........................................................................................ucaucuauuuuggaacggag..   9    0    S02
........................................................................................ucaucuauuuuggaacggag..   8    0    S03
........................................................................................ucaucuauuuuggaacggagg.   5    0    S03
........................................................................................ucaucuauuuuggaacggagg.   15   0    S02
........................................................................................ucaucuauuuuggaacggagg.   6    0    S06
........................................................................................ucaucuauuuuggaacggagg.   14   0    S04
........................................................................................ucaucuauuuuggaacggagg.   19   0    S05
........................................................................................ucaucuauuuuggaacggagg.   17   0    S01
.........................................................................................caucuauuuuggaacgga...   3    0    S06
.........................................................................................caucuauuuuggaacgga...   1    0    S01
.........................................................................................caucuauuuuggaacgga...   1    0    S03
.........................................................................................caucuauuuuggaacgga...   1    0    S05
.........................................................................................caucuauuuuggaacgga...   1    0    S04
.........................................................................................caucuauuuuggaacggag..   1    0    S05
.........................................................................................caucuauuuuggaacggag..   1    0    S06
.........................................................................................caucuauuuuggaacggag..   1    0    S03
.........................................................................................caucuauuuuggaacggagg.   3    0    S01
.........................................................................................caucuauuuuggaacggagg.   1    0    S03
.........................................................................................caucuauuuuggaacggagg.   10   0    S06
.........................................................................................caucuauuuuggaacggagg.   2    0    S02
.........................................................................................caucuauuuuggaacggaggg   5    0    S04
.........................................................................................caucuauuuuggaacggaggg   2    0    S03
.........................................................................................caucuauuuuggaacggaggg   3    0    S01
.........................................................................................caucuauuuuggaacggaggg   3    0    S05
.........................................................................................caucuauuuuggaacggaggg   2    0    S02
.........................................................................................caucuauuuuggaacggaggg   1    0    S06
..........................................................................................aucuauuuuggaacggag..   3    0    S06
..........................................................................................aucuauuuuggaacggag..   1    0    S04
..........................................................................................aucuauuuuggaacggag..   3    0    S05
..........................................................................................aucuauuuuggaacggagg.   2    0    S04
..........................................................................................aucuauuuuggaacggagg.   2    0    S06
..........................................................................................aucuauuuuggaacggagg.   2    0    S02
..........................................................................................aucuauuuuggaacggaggg   3    0    S05
..........................................................................................aucuauuuuggaacggaggg   2    0    S01
..........................................................................................aucuauuuuggaacggaggg   5    0    S02
..........................................................................................aucuauuuuggaacggaggg   5    0    S03
..........................................................................................aucuauuuuggaacggaggg   2    0    S04
```

```
                                 Star                                                       Mature
ccaaaauaggugacccaacuuuauacuaauucccuccguuccaaaauagaugaaccaacuuuauacuaacuuuuaguauaaaguuggu ucaucuauuuuggaacggaggg
..........................................................................................aucuauuuuggaacggaggg    4    0    S06
...........................................................................................ucuauuuuggaacggagg.    1    0    S04
...........................................................................................ucuauuuuggaacggaggg    1    0    S04
```

Provisional ID	: ta_iwgsc_2bl_v1_8004886_1446426		novel-m0875-5p
Score total	:	2.9	
Score for star read(s)	:	-1.3	
Score for read counts	:	0	
Score for mfe	:	2.6	
Score for randfold	:	1.6	
Score for cons. seed	:		
Total read count	:	2575	
Mature read count	:	2836	
Loop read count	:	0	
Star read count	:	282	

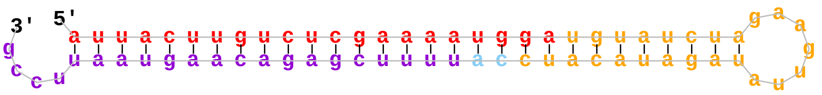

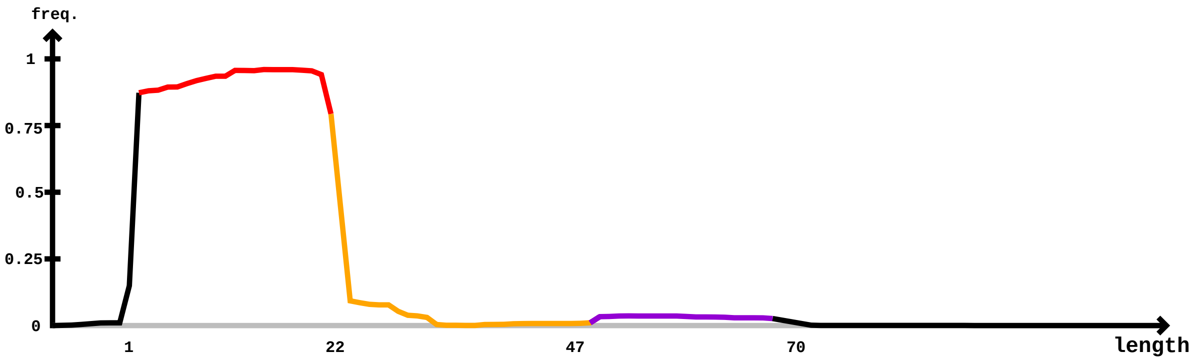

Mature **Star**

```
5'- guucggaauuacuugucucgaaaauggauguaucuagaaguuauagauacauccauuuucgagacaaguaauuccgaacgaguaguaauuugcccugcauccuuuacuuuggac   -3'    obs
    guucggaauuacuugucucgaaaauggauguaucuagaaguuauagauacaucaauuuucgagacaaguaauuccgaacgaguaguaauuugcccugcauccuuuacuuuggac          exp
    ((((((((((((((((((((((((((((((((((((.......))))))))))))))))))))))))))))))))))))((((((.....(((...)))....)))))).....  reads    mm    sample
    guucggaauuacuugucu................................................................................................  1        0     S03
    guucggaauuacuugucucg..............................................................................................  1        0     S06
    guucggaauuacuugucucga.............................................................................................  1        0     S01
    guucggaauuacuugucucgaGaa..........................................................................................  1        1     S04
    guucggaauuacuugucucgaaaaugg.......................................................................................  1        0     S02
    .uucggaauuacuugucuc...............................................................................................  1        0     S04
    .uucggaauuacuugucuc...............................................................................................  1        0     S06
    .uucggaauuacuugucuc...............................................................................................  1        0     S01
    .uucggaauuacuugucucg..............................................................................................  2        0     S04
    .uucggaauuacuugucucgaGaau.........................................................................................  2        1     S05
    ..ucggaauuacuugucucg..............................................................................................  1        0     S05
    ..ucggaauuacuugucucgaGa...........................................................................................  1        1     S04
    ..ucggaauuacuugucucgaaaaug........................................................................................  1        0     S05
    ..ucggaauuacuugucucgaaaaug........................................................................................  2        0     S06
    ..ucggaauuacuugucucgaaaaug........................................................................................  1        0     S04
    ..ucggaauuacuugucucgaaaaug........................................................................................  1        0     S03
    ..ucggaauuacuugucucgaaaaug........................................................................................  1        0     S01
    ...cggaauuacuugucucgaa............................................................................................  1        0     S05
    ...cggaauuacuugucucgaaaa..........................................................................................  1        0     S04
    ...cggaauuacuugucucgaaaaug........................................................................................  1        0     S02
    ...cggaauuacuugucucgaaaaug........................................................................................  1        0     S03
    ...cggaauuacuugucucgaaaaug........................................................................................  1        0     S05
    ...cggaauuacuugucucgaaaaugg.......................................................................................  1        0     S05
    ...cggaauuacuugucucgaaaaugg.......................................................................................  1        0     S06
    ...cggaauuacuugucucgaaaauggaugua...................................................................................  1        0     S05
    ....ggaauuacuugucucgaaaa..........................................................................................  1        0     S03
    ......aauuacuugucucgaaaa..........................................................................................  2        0     S04
    ......aauuacuugucucgaaaa..........................................................................................  1        0     S02
    ......aauuacuugucucgaaaau.........................................................................................  2        0     S02
    ......aauuacuugucucgaaaaug........................................................................................  4        0     S04
    ......aauuacuugucucgaaaaug........................................................................................  2        0     S06
    ......aauuacuugucucgaaaaug........................................................................................  1        0     S05
    ......aauuacuugucucgaaaaug........................................................................................  5        0     S03
```

Mature Star

```
guucggaauuacuugucucgaaaauggauguaucuagaaguuauagauacauccauuucgagacaaguaauuccgaacgaguaguaauuugcccugcauccuuuacuuuggac
......aauuacuugucucgaaaaug.......................................................................................	6	0	S02
......aauuacuugucucgaaaaug.......................................................................................	5	0	S01
......aauuacuugucucgaaaaugg......................................................................................	125	0	S01
......aauuacuugucucgaaaaugg......................................................................................	51	0	S05
......aauuacuugucucgaaaaugg......................................................................................	33	0	S03
......aauuacuugucucgaaaaugg......................................................................................	12	0	S06
......aauuacuugucucgaaaaugg......................................................................................	56	0	S04
......aauuacuugucucgaaaaugg......................................................................................	76	0	S02
......aauuacuugucucgaaaaugga.....................................................................................	1	0	S03
......aauuacuugucucgaaaaugga.....................................................................................	1	0	S06
......aauuacuugucucgaaaaugga.....................................................................................	3	0	S01
......aauuacuugucucgaaaaugga.....................................................................................	1	0	S02
......aauuacuugucucgaaaauggau....................................................................................	1	0	S05
......aauuacuugucucgaaaauggau....................................................................................	1	0	S01
......aauuacuugucucgaaaauggau....................................................................................	1	0	S06
......aauuacuugucucgaaaauggau....................................................................................	1	0	S04
......aauuacuugucucgaaaauggaug..................................................................................	2	0	S03
......aauuacuugucucgaaaauggaug..................................................................................	1	0	S02
......aauuacuugucucgaaaauggaug..................................................................................	3	0	S05
.......auuacuugucucgaaaau........................................................................................	2	0	S01
.......auuacuugucucgaaaau........................................................................................	1	0	S04
.......auuacuugucucgaaaaug.......................................................................................	2	0	S01
.......auuacuugucucgaaaaug.......................................................................................	3	0	S06
.......auuacuugucucgaaaaug.......................................................................................	2	0	S03
.......auuacuugucucgaaaaugg......................................................................................	15	0	S02
.......auuacuugucucgaaaaugg......................................................................................	6	0	S06
.......auuacuugucucgaaaaugg......................................................................................	19	0	S01
.......auuacuugucucgaaaaugg......................................................................................	8	0	S03
.......auuacuugucucgaaaaugg......................................................................................	3	0	S05
.......auuacuugucucgaaaaugg......................................................................................	12	0	S04
.......auuacuugucucgaaaaugga.....................................................................................	508	0	S04
.......auuacuugucucgaaaaugga.....................................................................................	587	0	S01
.......auuacuugucucgaaaaugga.....................................................................................	96	0	S03
.......auuacuugucucgaaaaugga.....................................................................................	315	0	S02
.......auuacuugucucgaaaaugga.....................................................................................	376	0	S05
.......auuacuugucucgaaaaugga.....................................................................................	81	0	S06
.......auuacuugucucgaaaauggau....................................................................................	1	0	S01
.......auuacuugucucgaaaauggaug..................................................................................	2	0	S02
.......auuacuugucucgaaaauggaug..................................................................................	1	0	S05
.......auuacuugucucgaaaauggaug..................................................................................	1	0	S06
.......auuacuugucucgaaaauggaug..................................................................................	2	0	S03
.......auuacuugucucgaaaauggaug..................................................................................	5	0	S04
.......auuacuugucucgaaaauggaugu.................................................................................	4	0	S04
.......auuacuugucucgaaaauggaugu.................................................................................	2	0	S06
.......auuacuugucucgaaaauggaugu.................................................................................	1	0	S03
.......auuacuugucucgaaaauggaugu.................................................................................	2	0	S05
.......auuacuugucucgaaaauggaugu.................................................................................	3	0	S01
........uuacuugucucgaaaaug.......................................................................................	1	0	S02
........uuacuugucucgaaaaugg......................................................................................	1	0	S02
........uuacuugucucgaaaaugga.....................................................................................	1	0	S02
........uuacuugucucgaaaaugga.....................................................................................	4	0	S01
........uuacuugucucgaaaaugga.....................................................................................	3	0	S04
........uuacuugucucgaaaaugga.....................................................................................	2	0	S05
........uuacuugucucgaaaauggau....................................................................................	3	0	S05
........uuacuugucucgaaaauggau....................................................................................	1	0	S03
........uuacuugucucgaaaauggau....................................................................................	2	0	S04
........uuacuugucucgaaaauggaugu.................................................................................	2	0	S01
........uuacuugucucgaaaauggaugu.................................................................................	1	0	S02
.........uacuugucucgaaaaugga.....................................................................................	3	0	S05
.........uacuugucucgaaaaugga.....................................................................................	1	0	S06
.........uacuugucucgaaaauggaug...................................................................................	1	0	S04
.........uacuugucucgaaaauggaug...................................................................................	1	0	S03
.........uacuugucucgaaaauggaugu..................................................................................	1	0	S04
..........acuugucucgaaaaugga.....................................................................................	1	0	S05
..........acuugucucgaaaaugga.....................................................................................	1	0	S01
..........acuugucucgaaaauggaug...................................................................................	1	0	S03
..........acuugucucgaaaauggaugua.................................................................................	1	0	S04
..........acuugucucgaaaauggauguauc...............................................................................	3	0	S01
..........acuugucucgaaaauggauguauc...............................................................................	4	0	S06
..........acuugucucgaaaauggauguauc...............................................................................	6	0	S04
```

Mature Star

```
guucggaauuacuugucucgaaaauggauguaucuagaaguuauagauacauccauuuucgagacaaguaauuccgaacgaguaguaauuugcccugcauccuuuacuuuggac
..........acuugucucgaaaauggauguauc................................................................................ 2   0  S03
..........acuugucucgaaaauggauguauc................................................................................ 9   0  S05
..........acuugucucgaaaauggauguauc................................................................................ 5   0  S02
...........cuugucucgaaaauggaugua.................................................................................. 1   0  S04
............uugucucgaaaauggaugua.................................................................................. 1   0  S05
............uugucucgaaaauggauguauc................................................................................ 8   0  S05
............uugucucgaaaauggauguauc................................................................................ 3   0  S06
............uugucucgaaaauggauguauc................................................................................ 9   0  S04
............uugucucgaaaauggauguauc................................................................................ 2   0  S01
............uugucucgaaaauggauguaucu............................................................................... 1   0  S03
............uugucucgaaaauggauguaucu............................................................................... 2   0  S04
............uugucucgaaaauggauguaucu............................................................................... 9   0  S05
............uugucucgaaaauggauguaucua.............................................................................. 1   0  S02
.............ugucucgaaaauggaugu................................................................................... 1   0  S05
.............ugucucgaaaauggaugua.................................................................................. 1   0  S06
.............ugucucgaaaauggaugua.................................................................................. 1   0  S05
.............ugucucgaaaauggauguauc................................................................................ 6   0  S05
.............ugucucgaaaauggauguauc................................................................................ 5   0  S04
.............ugucucgaaaauggauguauc................................................................................ 1   0  S02
.............ugucucgaaaauggauguauc................................................................................ 2   0  S06
.............ugucucgaaaauggauguaucu............................................................................... 1   0  S01
.............ugucucgaaaauggauguaucu............................................................................... 4   0  S05
.............ugucucgaaaauggauguaucu............................................................................... 2   0  S04
.............ugucucgaaaauggauguaucu............................................................................... 1   0  S02
.............ugucucgaaaauggauguaucua.............................................................................. 1   0  S03
.............ugucucgaaaauggauguaucuaga............................................................................ 1   0  S05
.............ugucucgaaaauggauguaucuaga............................................................................ 3   0  S02
.............ugucucgaaaauggauguaucuaga............................................................................ 1   0  S03
.............ugucucgaaaauggauguaucuaga............................................................................ 1   0  S06
..............gucucgaaaauggauguaucuaga............................................................................ 2   0  S02
..............gucucgaaaauggauguaucuaga............................................................................ 3   0  S06
..............gucucgaaaauggauguaucuaga............................................................................ 2   0  S03
..............gucucgaaaauggauguaucuaga............................................................................ 7   0  S04
..............gucucgaaaauggauguaucuaga............................................................................ 9   0  S05
..............gucucgaaaauggauguaucuaga............................................................................ 1   0  S01
...............ucucgaaaauggauguauc................................................................................ 1   0  S01
...............ucucgaaaauggauguauc................................................................................ 3   0  S05
...............ucucgaaaauggauguaucu............................................................................... 2   0  S05
...............ucucgaaaauggauguaucua.............................................................................. 1   0  S01
...............ucucgaaaauggauguaucua.............................................................................. 2   0  S04
...............ucucgaaaauggauguaucua.............................................................................. 1   0  S03
...............ucucgaaaauggauguaucuag............................................................................. 1   0  S02
...............ucucgaaaauggauguaucuag............................................................................. 2   0  S06
...............ucucgaaaauggauguaucuag............................................................................. 1   0  S05
...............ucucgaaaauggauguaucuaga............................................................................ 1   0  S04
...............ucucgaaaauggauguaucuaga............................................................................ 2   0  S02
...............ucucgaaaauggauguaucuaga............................................................................ 1   0  S06
...............ucucgaaaauggauguaucuaga............................................................................ 1   0  S05
...............ucucgaaaauggauguaucuaga............................................................................ 1   0  S03
...............ucucgaaaauggauguaucuagaa........................................................................... 1   0  S02
...............ucucgaaaauggauguaucuagaa........................................................................... 2   0  S04
.................ucgaaaauggauguaucu............................................................................... 6   0  S01
.................ucgaaaauggauguaucu............................................................................... 3   0  S04
.................ucgaaaauggauguaucu............................................................................... 6   0  S05
.................ucgaaaauggauguaucu............................................................................... 1   0  S06
.................ucgaaaauggauguaucu............................................................................... 4   0  S02
.................ucgaaaauggauguaucua.............................................................................. 1   0  S01
.................ucgaaaauggauguaucuag............................................................................. 1   0  S01
.................ucgaaaauggauguaucuag............................................................................. 5   0  S04
.................ucgaaaauggauguaucuag............................................................................. 4   0  S05
.................ucgaaaauggauguaucuag............................................................................. 1   0  S03
.................ucgaaaauggauguaucuag............................................................................. 1   0  S02
.................ucgaaaauggauguaucuaga............................................................................ 8   0  S04
.................ucgaaaauggauguaucuaga............................................................................ 3   0  S03
.................ucgaaaauggauguaucuaga............................................................................ 1   0  S02
.................ucgaaaauggauguaucuaga............................................................................ 2   0  S06
.................ucgaaaauggauguaucuaga............................................................................ 12  0  S05
.................ucgaaaauggauguaucuagU............................................................................ 1   1  S04
.................ucgaaaauggauguaucuagaa........................................................................... 1   0  S01
.................ucgaaaauggauguaucuagaCgu......................................................................... 1   1  S05
```

Mature **Star**

guucggaauuacuugucucgaaaauggauguaucuagaaguuauagauacauccauuuucgagacaaguaauuccgaacgaguaguaauuugcccugcauccuuuacuuuggac			
...................gaaaauggauguaucuagaa...	1	0	S01
....................aaaauggauguaucuaga..	1	0	S02
....................aaaauggauguaucuaga..	3	0	S06
....................aaaauggauguaucuaga..	2	0	S03
....................aaaauggauguaucuaga..	5	0	S01
....................aaaauggauguaucuaga..	2	0	S05
....................aaaauggauguaucuagaa...	1	0	S03
....................aaaauggauguaucuagaa...	1	0	S05
....................aaaauggauguaucuagaa...	1	0	S04
......................aauggauguaucuagaag..	1	0	S03
.......................auggauguaucuagaagu...	1	0	S06
....uuauagauacauccauuuu...	1	0	S04
....uagauacauccauuuucgaga..	4	0	S04
....uagauacauccauuuucgaga..	1	0	S05
....uagauacauccauuuucgagac...	3	0	S05
....uagauacauccauuuucgagac...	1	0	S04
....uagauacauccauuuucgagacaa...	1	0	S06
....agauacauccauuuucgagacaag..	1	0	S05
....gauacauccauuuucgagacaagu...	1	0	S04
....auacauccauuuucgagac...	1	0	S01
....auacauccauuuucgagacaagu...	4	0	S05
....auacauccauuuucgagacaagua..	1	0	S04
....uacauccauuuucgagacaag..	1	0	S04
....uacauccauuuucgagacaagu...	1	0	S01
....acauccauuuucgagacaaguaau..	1	0	S05
....cauuuucgagacaaguaauuc..	1	0	S04
....cauuuucgagacaaguaauuccg......................................	1	0	S02
....auuuucgagacaaguaauucc.......................................	1	0	S04
....auuuucgagacaaguaauucc.......................................	1	0	S05
....auuuucgagacaaguaauuccga.....................................	1	0	S05
....auuuucgagacaaguaauuccga.....................................	2	0	S04
....auuuucgagacaaguaauuccgaa....................................	1	0	S04
....uuuucgagacaaguaauu...	1	0	S03
....uuuucgagacaaguaauu...	2	0	S01
....uuuucgagacaaguaauu...	2	0	S05
....uuuucgagacaaguaauu...	1	0	S02
....uuuucgagacaaguaauu...	1	0	S04
....uuuucgagacaaguaauuc..	1	0	S06
....uuuucgagacaaguaauuc..	4	0	S05
....uuuucgagacaaguaauuc..	1	0	S02
....uuuucgagacaaguaauuc..	1	0	S04
....uuuucgagacaaguaauuc..	1	0	S01
....uuuucgagacaaguaauucc.......................................	1	0	S01
....uuuucgagacaaguaauucc.......................................	6	0	S05
....uuuucgagacaaguaauucc.......................................	2	0	S04
....uuuucgagacaaguaauucc.......................................	1	0	S06
....uuuucgagacaaguaauuccg......................................	1	0	S02
....uuuucgagacaaguaauuccg......................................	15	0	S04
....uuuucgagacaaguaauuccg......................................	12	0	S05
....uuuucgagacaaguaauuccg......................................	3	0	S01
....uuuucgagacaaguaauuccga.....................................	1	0	S05
....uuuucgagacaaguaauuccga.....................................	1	0	S06
....uuuucgagacaaguaauuccga.....................................	5	0	S04
....uuuucgagacaaguaauuccgaU....................................	2	1	S04
....uuucgagacaaguaauuccg......................................	1	0	S01
....uuucgagacaaguaauuccg......................................	1	0	S06
....uucgagacaaguaauucc.......................................	2	0	S05
....uucgagacaaguaauuccg......................................	1	0	S03
....uucgagacaaguaauuccg......................................	2	0	S05
....ucgagacaaguaauuccg......................................	1	0	S01
....auuccgaacgaguaguaauuugcc....................	1	0	S06

Provisional ID	:	ta_iwgsc_2bl_v1_8011970_1456898
Score total	:	16
Score for star read(s)	:	3.9
Score for read counts	:	7.8
Score for mfe	:	2.8
Score for randfold	:	1.6
Score for cons. seed	:	
Total read count	:	27
Mature read count	:	71
Loop read count	:	2
Star read count	:	63

novel-m0146-3p

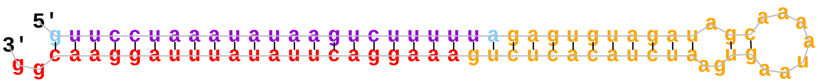

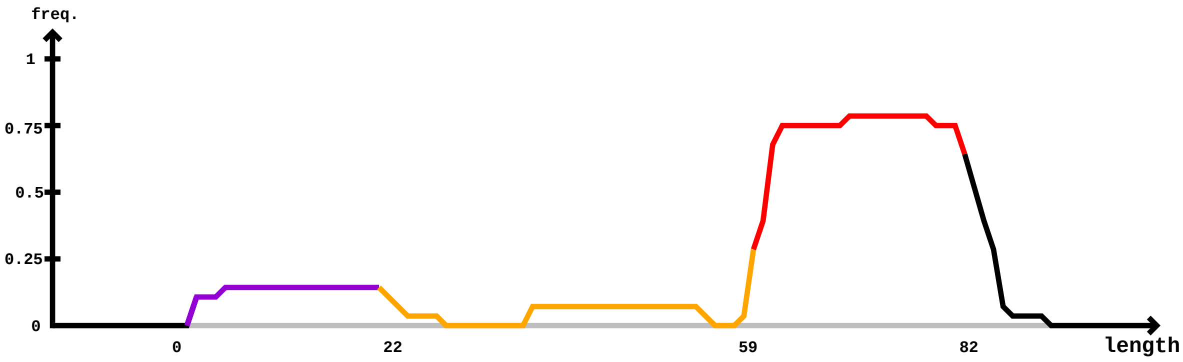

Star Mature

```
5'- aaugcucccuccguuccuaaauauaagucuuuuuagaguguagauagcaaaauaagugaaucuacacucugaaaggacuuauauuuaggaacggaggggggaguaguuaucuu   -3'    obs
    aaugcucccuccguuccuaaauauaagucuuuuuagaguguagauagcaaaauaagugaaucuacacucugaaaggacuuauauuuaggaacggaggggggaguaguuaucuu          exp
    ..(.(((((((((((((((((((((((((((((((((((((((((.((.......))..))))))))))))))))))))))))))))))))))))))))).)...........   reads    mm    sample
    .............uuccuaaauauaagucuAuuu...............................................................................   1        1     S05
    .............uuccuaaauauaagucuAuuu...............................................................................   1        1     S01
    .............uuccuaaauauaagucuAuuua..............................................................................   1        1     S04
    ................cuaaauauaagucuuuuAagagu..........................................................................   1        1     S04
    ................................................aaaauaagugaaucuaca...............................................   1        0     S06
    ................................................aaaauaagugaaucuacac..............................................   1        0     S06
    ......................................................................gaaaggacuuauauuuaggaacg....................   1        0     S05
    .......................................................................aaaggacuuauauuuaggaaUg....................   1        1     S03
    .......................................................................aaaggacuuauauuuaggaacgg...................   2        0     S05
    .......................................................................aaaggacuuauauuuaggaacgga..................   1        0     S04
    .......................................................................aaaggacuuauauuuaggaaUgga..................   1        1     S04
    .......................................................................aaaggacuuauauuuaggaaUgga..................   1        1     S02
    .......................................................................aaaggacuuauauuuaggaacgga..................   1        0     S05
    ........................................................................aaggacuuauauuuagga.......................   1        0     S06
    ........................................................................aaggacuuauauuuaggaacggag.................   2        0     S05
    .........................................................................aggacuuauauuuaggaacgg...................   1        0     S03
    .........................................................................aggacuuauauuuaggaacggag.................   1        0     S02
    .........................................................................aggacuuauauuuaggaacggagg................   1        0     S02
    .........................................................................aggacuuauauuuaggaacggagg................   1        0     S04
    .........................................................................aggacuuauauuuaggaacggagg................   1        0     S01
    .........................................................................aggacuuauauuuaggaacggagg................   1        0     S06
    .........................................................................aggacuuauauuuaggaacggagg................   2        0     S05
    ..........................................................................ggacuuauauuuaggaacg....................   1        0     S01
    ..........................................................................ggacuuauauuuaggaacggaggg...............   1        0     S04
    .................................................................................uauuuaggaacggagggggga...........   1        0     S04
```

Provisional ID	:	ta_iwgsc_2al_v1_6363060_994006
Score total	:	2332.1
Score for star read(s)	:	3.9
Score for read counts	:	2323.9
Score for mfe	:	2.7
Score for randfold	:	1.6
Score for cons. seed	:	
Total read count	:	4570
Mature read count	:	5139
Loop read count	:	0
Star read count	:	6

novel-m0661-5p

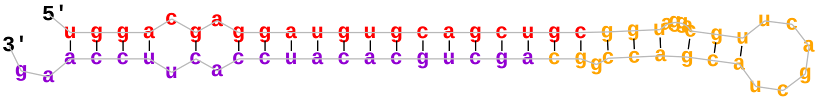

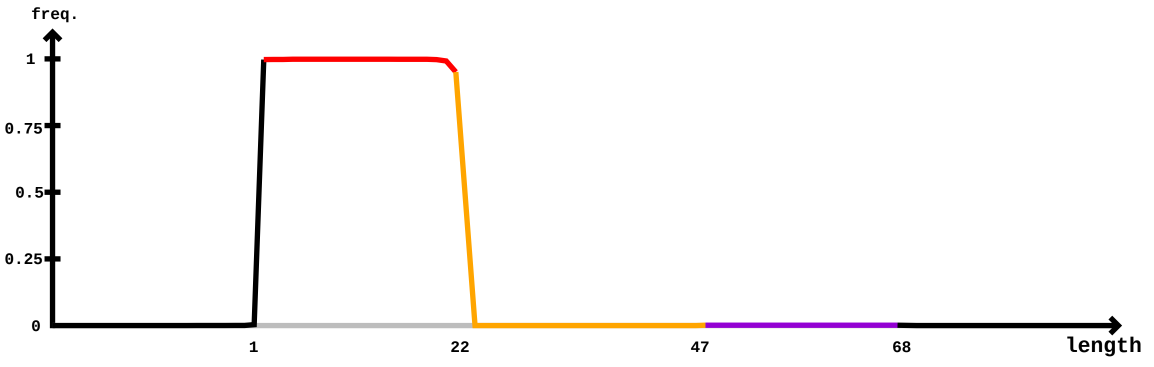

Mature Star

```
5'- guacgguccugguuagaguuuggacgaggaugugcagcugcgguaggacguucagcuacgaccggcagcugcacauccacuuccaagcgcuagcuaggaucgucaacaa  -3'   obs
    guacgguccugguuagaguuuggacgaggaugugcagcugcgguaggacguucagcuacgaccggcagcugcacauccacuuccaagcgcuagcuaggaucgucaacaa         exp
    ..((((((((((((((.(((((((.(.(((((((((((((((((....(((......)))))).)))))))))))))).).))))))).))))))))))))))......   reads   mm   sample
    .............uagaguuuggacgaggaugug...........................................................................   1       0    S05
    .................guuuggacgaggaugugcagc.......................................................................   1       0    S04
    ...................uuggacgaggaugugcagcu......................................................................   1       0    S01
    ...................uuggacgaggaugugcagcu......................................................................   1       0    S04
    ...................uuggacgaggaugugcagcug.....................................................................   3       0    S05
    ...................uuggacgaggaugugcagcug.....................................................................   2       0    S01
    ...................uuggacgaggaugugcagcug.....................................................................   6       0    S04
    ...................uuggacgaggaugugcagcugc....................................................................   1       0    S02
    ....................uggacgaggaugugcagc.......................................................................   3       0    S05
    ....................uggacgaggaugugcagc.......................................................................   2       0    S02
    ....................uggacgaggaugugcagcu......................................................................   5       0    S02
    ....................uggacgaggaugugcagcu......................................................................   3       0    S04
    ....................uggacgaggaugugcagcu......................................................................   7       0    S01
    ....................uggacgaggaugugcagcu......................................................................   4       0    S03
    ....................uggacgaggaugugcagcu......................................................................   2       0    S05
    ....................uggacgaggaugugcagcug.....................................................................   47      0    S02
    ....................uggacgaggaugugcagcug.....................................................................   33      0    S04
    ....................uggacgaggaugugcagcug.....................................................................   20      0    S01
    ....................uggacgaggaugugcagcug.....................................................................   9       0    S06
    ....................uggacgaggaugugcagcug.....................................................................   20      0    S05
    ....................uggacgaggaugugcagcug.....................................................................   49      0    S03
    ....................uggacgaggaugugcagcugc....................................................................   121     0    S06
    ....................uggacgaggaugugcagcugc....................................................................   892     0    S04
    ....................uggacgaggaugugcagcugc....................................................................   634     0    S05
    ....................uggacgaggaugugcagcugc....................................................................   842     0    S01
    ....................uggacgaggaugugcagcugc....................................................................   382     0    S03
    ....................uggacgaggaugugcagcugc....................................................................   1469    0    S02
    .....................ggacgaggaugugcagcugc....................................................................   1       0    S02
    .....................ggacgaggaugugcagcugc....................................................................   1       0    S01
    .......................acgaggaugugcagcugc....................................................................   1       0    S01
    .......................acgaggaugugcagcugc....................................................................   2       0    S03
    .......................acgaggaugugcagcugc....................................................................   1       0    S02
    ..................................................................agcugcacauccacuuccaag......................   2       0    S02
```

Mature Star

```
guacgguccugguuagaguuuggacgaggaugugcagcugcgguaggacguucagcuacgaccggcagcugcacauccacuuccaagcgcuagcuaggaucgucaacaa
.................................................................agcugcacauccacuuccaag......................	3	0	S03
.................................................................agcugcacauccacuuccaag......................	1	0	S01
```

Provisional ID	:	ta_iwgsc_2bl_v1_8076048_1541104
Score total	:	938.3
Score for star read(s)	:	3.9
Score for read counts	:	930.6
Score for mfe	:	2.3
Score for randfold	:	1.6
Score for cons. seed	:	
Total read count	:	1837
Mature read count	:	2215
Loop read count	:	0
Star read count	:	104

novel-m0704-5p

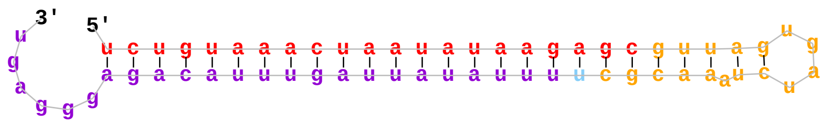

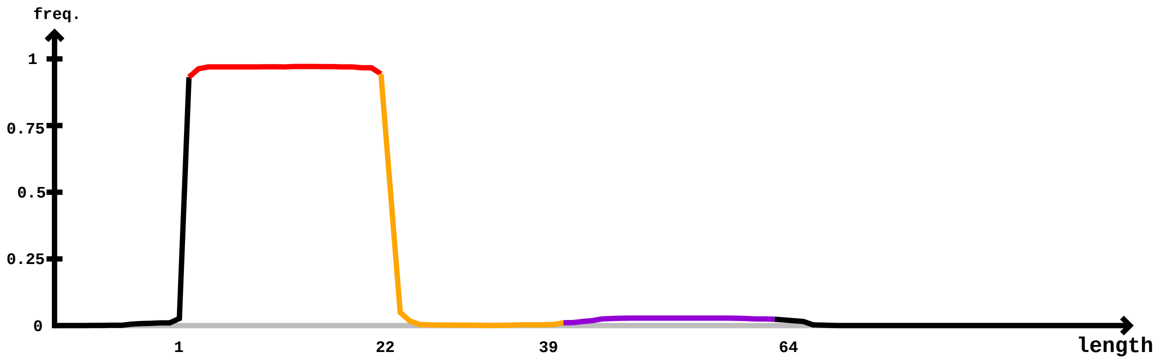

Mature **Star**

```
5'- auacguacucccucuguaaacuaauauaagagcguuagugaucuaaacgcuuuuauauuaguuuacagagggaguacauuaauaagcaaaggaaagagauauagguagg  -3'    obs
    auacguacucccucuguaaacuaauauaagagcguuagugaucuaaacgcuuuuauauuaguuuacagagggaguacauuaauaagcaaaggaaagagauauagguagg          exp
    ....(((((((((((((((((((((((((((((((((....)).)))))))))))))))))))))))))))))))..................................   reads    mm    sample
    ..acguacucccucuguaaacuaaua...................................................................................   1        0     S05
    ....guacucccucuguaaacu.......................................................................................   1        0     S01
    ......aGucccucuguaaacuaauauaag...............................................................................   1        1     S04
    ......acucAcucuguaaacuaauauaag...............................................................................   2        1     S05
    ......acucccucuguaCacuaauauaag...............................................................................   1        1     S06
    ......acuGccucuguaaacuaauauaag...............................................................................   1        1     S05
    ......acucAcucuguaaacuaauauaag...............................................................................   1        1     S02
    ......acucccucuguaaacuaauauaagag.............................................................................   1        0     S03
    ......acucccucuguaaacuaauauaagagc............................................................................   1        0     S05
    .......cucccucuguaaacuaauauaGa...............................................................................   1        1     S05
    .......cucccucuguaaacuaauauaagag.............................................................................   2        0     S01
    .......cucccucuguaaacuaauauaagagc............................................................................   1        0     S01
    ........uGccucuguaaacuaauaua.................................................................................   1        1     S05
    ........ucccucuguaaacuaauauaagag.............................................................................   1        0     S01
    .........cccAcuguaaacuaauauaagag.............................................................................   1        1     S05
    .........cccucuguaaacuaauauaagagc............................................................................   1        0     S04
    .........cccucuguaaacuaauauaagagc............................................................................   1        0     S02
    ...........cucuguaaacuaauauaagagc............................................................................   1        0     S04
    ...........cucuguaaacuaauauaagagcg...........................................................................   3        0     S04
    ...........cucuguaaacuaaCauaagagcg...........................................................................   1        1     S06
    ...........cucuguaaacuaauauaagagcg...........................................................................   3        0     S02
    ...........cucuguaaacuaauauaagagcg...........................................................................   3        0     S06
    ...........cucuguaaacuaauauaagagcg...........................................................................   1        0     S01
    ...........cucuguaaacuaauauaagagcgu..........................................................................   6        0     S01
    ...........cucuguaaacuaauauaagagcgu..........................................................................   5        0     S04
    ...........cucuguaaacuaauauaagagcgu..........................................................................   5        0     S02
    ...........cucuguaaacuaauauaagagcgu..........................................................................   1        0     S03
    ...........cucuguaaacuaauauaagagcgu..........................................................................   2        0     S06
    ............ucuguaaacuaauauaagag.............................................................................   4        0     S05
    ............ucuguaaacuaauauaagag.............................................................................   6        0     S03
    ............ucuguaaacuaauauaagag.............................................................................   4        0     S04
    ............ucuguaaacuaauauaagag.............................................................................   8        0     S02
    ............ucuguaaacuaauauaagag.............................................................................   9        0     S06
```

```
                Mature                    Star
auacguacucccucuguaaacuaauauaagagcguuagugaucuaaacgcuuuuauauuaguuuacagagggaguacauuaauaagcaaaaggaaagagauauagguagg
............ucuguaaacuaauauaagag..............................................................................   6     0   S01
............ucuguaaacuGauauaagagc.............................................................................   1     1   S06
............ucuguaaacuGauauaagagc.............................................................................   2     1   S04
............ucuguaaacuaauauaagagc.............................................................................   375   0   S05
............ucuguaaacuaaCauaagagc.............................................................................   1     1   S04
............ucuguaCacuaauauaagagc.............................................................................   1     1   S04
............ucuguaaacuaauaCaagagc.............................................................................   1     1   S02
............ucuguaaacuaauauaagagc.............................................................................   189   0   S06
............ucuguaaacuaauauaagagc.............................................................................   286   0   S01
............ucuguaaacuaaCauaagagc.............................................................................   2     1   S03
............ucuguaaacuaGuauaagagc.............................................................................   1     1   S01
............ucuguaaacuaauauaagagc.............................................................................   107   0   S03
............ucuguaaacuaauauaagagc.............................................................................   384   0   S04
............ucuguaaacuaauauaagagc.............................................................................   248   0   S02
............ucuguaaacuaauauaagagcg............................................................................   2     0   S06
............ucuguaaacuGauauaagagcg............................................................................   1     1   S01
............ucGguaaacuaauauaagagcg............................................................................   2     1   S02
............ucGguaaacuaauauaagagcg............................................................................   1     1   S05
............ucuguaaacuaauauaagagcg............................................................................   4     0   S01
............ucuguaaacuaauauaagagcg............................................................................   3     0   S02
............ucuguaaacuaauauaagagcg............................................................................   4     0   S05
............ucuguaaacuaauauaagagcg............................................................................   1     0   S04
............ucuguaaacuaauauaagagcg............................................................................   2     0   S03
............ucuguaaacuaaCauaagagcg............................................................................   1     1   S04
............ucuguaaacuaauauaagagcgu...........................................................................   4     0   S02
............ucuguaaacuaauauaagagcgu...........................................................................   10    0   S05
............uGuguaaacuaauauaagagcgu...........................................................................   1     1   S04
............ucuguaaacuaauauaagagcgu...........................................................................   3     0   S06
............ucuguaaacuaauauaagagcgu...........................................................................   4     0   S01
............ucuguaaacuaauauaagagcgu...........................................................................   9     0   S04
............ucuguaaacuaauauaagagcguu..........................................................................   5     0   S01
............ucuguaaacuaauauaagagcguA..........................................................................   1     1   S06
............ucuguaaacuaauauaagagcguu..........................................................................   3     0   S04
............ucuguaaacuaauauaagagcguu..........................................................................   6     0   S05
............uGuguaaacuaauauaagagcguu..........................................................................   1     1   S05
............ucuguaaacuaauauaagagcgAu..........................................................................   1     1   S05
............ucuguaaacuaauauaagagcguu..........................................................................   6     0   S02
............ucuguaaacuaauauaagagcguua.........................................................................   1     0   S02
............ucuguaaacuaauauaagagcguua.........................................................................   2     0   S04
............ucuguaaacuaauauaagagcguuaU........................................................................   1     1   S04
.............cuguaaacuaauauaagag..............................................................................   1     0   S01
.............cuguaaacuaauauaagag..............................................................................   1     0   S04
.............cuguaaacuaauauaagagc.............................................................................   1     0   S04
.............cuguaaacuaauauaagagc.............................................................................   2     0   S01
.............cuguaaacuaauauaagagcg............................................................................   8     0   S02
.............cuguaaacuaauauaagagcg............................................................................   7     0   S06
.............cuguaaacuaauauaagagcg............................................................................   12    0   S01
.............cuguaaacuaauauaagagcg............................................................................   9     0   S05
.............cuguaaacuaauauaagagcg............................................................................   9     0   S04
.............cuguaaacuaauauaagagcg............................................................................   5     0   S03
.............cuguaaacuaauauaagagcgu...........................................................................   2     0   S06
.............cuguaaacuaauauaagagcgu...........................................................................   1     0   S02
.............cuguaaacuaauauaagagcgu...........................................................................   1     0   S05
.............cuguaaacuaauauaagagcguu..........................................................................   1     0   S03
..............uguaaacuaauauaagagc.............................................................................   1     0   S01
..............uguaaacuaauauaagagc.............................................................................   1     0   S05
..............uguaaacuaauauaagagc.............................................................................   1     0   S04
..............uguaaacuaauauaagagcg............................................................................   1     0   S06
..............uguaaacuaauauaagagcg............................................................................   1     0   S05
..............uguaaacuaauauaagagcg............................................................................   1     0   S02
..............uguaaacuaauauaagagcgu...........................................................................   2     0   S06
..............uguaaacuaauauaagagcgu...........................................................................   2     0   S02
..............uguaaacuaauauaagagcgu...........................................................................   2     0   S01
..............uguaaacuaauauaagagcguA..........................................................................   1     1   S05
....................cuaauauaagagcguuagug......................................................................   1     0   S06
.......................auauaagagcguuagugau....................................................................   1     0   S06
.......................auauaagagcguuagugauc...................................................................   1     0   S01
.......................auauaagagcguuagugaucuaaac..............................................................   1     0   S06
.............................................aacgcuuuuauauuaguuuacaga.........................................   1     0   S04
..............................................acgcuuuuauauuaguuuacagag........................................   1     0   S06
```

Mature **Star**

```
auacguacucccucuguaaacuaauauaagagcguuagugaucuaaacgcuuuuauauuaguuuacagagggaguacauuaauaagcaaaaggaaagagauauagguagg
..............................................acgcuuuuauauuaguuuacagag........................................  1  0  S04
...............................................cgGuuuuauauuaguuuacagagg.......................................  1  1  S05
...............................................cgcuuuuauauuaguuuacagagg.......................................  1  0  S05
.................................................cuuuuauauuaguuuacagaggga.....................................  1  0  S04
..................................................uuuuauauuaguuuacagagg.......................................  1  0  S04
..................................................uuuuauauuaguuuacagagggag....................................  1  0  S01
...................................................uuuauauuaguuuacagagggag....................................  1  0  S06
...................................................uuuauauuaguuuacagagggagu...................................  2  0  S05
...................................................uuuauauuaAuuuacagagggagu...................................  1  1  S05
...................................................uuuauauuaguuuacagagggagu...................................  5  0  S04
...................................................uuuauauuaguuuacagagggagu...................................  1  0  S02
...................................................uuuauauuaguuuacagagggagu...................................  1  0  S01
...................................................uuuauauuaguuuacagagggagu...................................  1  0  S06
....................................................uuauauuaguuuacagagCga.....................................  1  1  S02
.....................................................uauauuaguuuacagagggaguac.................................  3  0  S04
.....................................................uauauuaguuuacagagggaguac.................................  1  0  S03
.....................................................uauauuaguuuacagagggaguac.................................  1  0  S01
.....................................................uauauuaguuuacagagggaguac.................................  3  0  S05
......................................................auauuaguuuacagagggagG...................................  1  1  S01
......................................................auauuaguuuacagagggaguac.................................  1  0  S04
......................................................auauuaguuuacagagggaguac.................................  1  0  S03
......................................................auauuaguuuacagagggaguac.................................  1  0  S06
......................................................auauuaguuuacagagggaguac.................................  1  0  S05
......................................................auauuaguuuacagagggaguaca................................  1  0  S05
.......................................................uauuaguuuacagagCgagua..................................  1  1  S05
.......................................................uauuaguuuacagagggagGa..................................  1  1  S02
.......................................................uauuaguuuacagagggaguac.................................  1  0  S02
.......................................................uauuaguuuacagagggaguac.................................  1  0  S04
.......................................................uauuaguuuacagagggaguac.................................  4  0  S05
.......................................................uauuaguuuacagagCgaguac.................................  1  1  S05
.......................................................uauuaguuuacagagggaguac.................................  1  0  S06
.......................................................uauuaguuuacagagggaguaca................................  1  0  S04
.......................................................uauuaguuuacagagggaguacau...............................  1  0  S01
........................................................auuaguuuacagagggaguac.................................  2  0  S04
........................................................auuaguuuacagagggaguac.................................  1  0  S01
.........................................................uuaguuuacagagggaguacau...............................  1  0  S04
.........................................................uuaguuuacagagAgaguacauu..............................  1  1  S04
..........................................................uaguuuacagagggaguac.................................  1  0  S04
```

Provisional ID	:	ta_iwgsc_2bs_v1_5128371_1609182
Score total	:	2.3
Score for star read(s)	:	-1.3
Score for read counts	:	0
Score for mfe	:	2
Score for randfold	:	1.6
Score for cons. seed	:	
Total read count	:	1025
Mature read count	:	990
Loop read count	:	0
Star read count	:	52

novel-m0467-5p

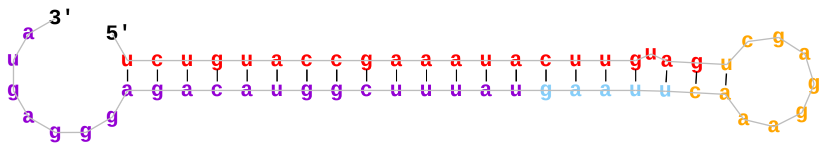

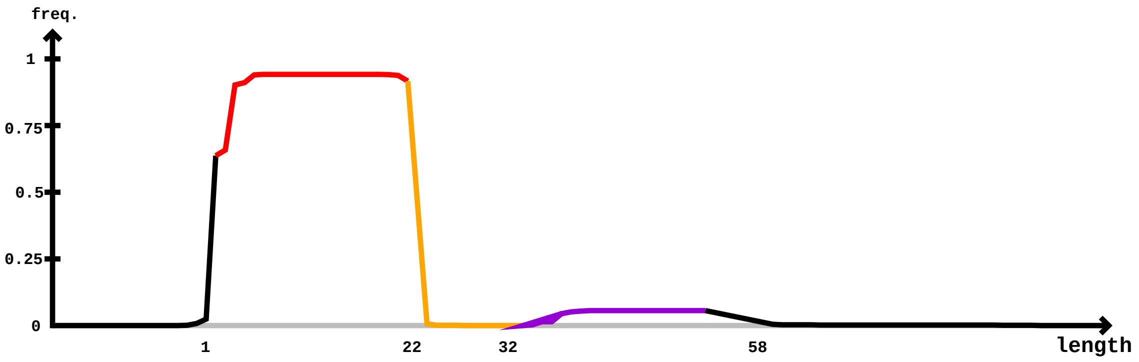

Mature Star

5'- gucacuagcacccccucuguaccgaaauacuuguagucgaggaaacuuaaguauuucgguacagagggaguaguagccaucacuaaucuuauucaugaacgugaaaac	-3'	obs		
gucacuagcacccccucuguaccgaaauacuuguagucgaggaaacuuaaguauuucgguacagagggaguaguagccaucacuaaucuuauucaugaacgugaaaac		exp		
....(((..((.(((((((((((((((((((((.(((.......))))))))))))))))))))))))).))..)))...((((...((........))..))))....	reads	mm	sample	
............cccucuguaccgaaauacuug...	1	0	S05	
.............ccucuguaccgaaauacuugua...	3	0	S05	
.............ccucuguaccgaaauacuuguag..	1	0	S03	
.............ccucuguaccgaaauacuuguag..	1	0	S01	
.............ccucuguaccgaaauacuuguagu...	1	0	S05	
.............ccucuguaccgaaauacuuguagu...	1	0	S01	
..............cucuguaccgaaauacuugu..	1	0	S03	
..............cucuguaccgaaauacuugua...	1	0	S03	
..............cucuguaccgaaauacuugua...	2	0	S02	
..............cucuguaccgaaauacuugua...	2	0	S04	
..............cucuguaccgaaauacuugua...	4	0	S06	
..............cucuguaccgaaauacuugua...	2	0	S01	
..............cucuguaccgaaauacuugua...	3	0	S05	
..............cucuguaccgaaauacuuguag..	1	0	S04	
..............cucuguaccgaaauacuuguagu...	1	0	S04	
...............ucuguaccgaaauacuugu..	1	0	S05	
...............ucuguaccgaaauacuugu..	1	0	S06	
...............ucuguaccgaaauacuugua...	2	0	S02	
...............ucuguaccgaaauacuugua...	3	0	S06	
...............ucuguaccgaaauacuuguag..	90	0	S06	
...............ucuguaccgaaauacuuguag..	40	0	S03	
...............ucuguaccgaaauacuuguag..	56	0	S05	
...............ucuguaccgaaauacuuguag..	29	0	S01	
...............ucuguaccgaaauacuuguag..	31	0	S04	
...............ucuguaccgaaauacuuguag..	34	0	S02	
...............ucuguaccgaaauacuuguagu...	45	0	S02	
...............ucuguaccgaaauacuuguagu...	34	0	S03	
...............ucuguaccgaaauacuuguagu...	60	0	S06	
...............ucuguaccgaaauacuuguagu...	89	0	S05	
...............ucuguaccgaaauacuuguagu...	70	0	S01	
...............ucuguaccgaaauacuuguagu...	48	0	S04	
................cuguaccgaaauacuuguag..	1	0	S04	
................cuguaccgaaauacuuguag..	3	0	S06	

Mature **Star**

```
gucacuagcacccccucuguaccgaaauacuuguagucgaggaaacuuaaguauuucgguacagagggaguaguagccaucacuaaucuuauucaugaacgugaaaac
................cuguaccgaaauacuuguag........................................................................ 1 0 S05
................cuguaccgaaauacuuguag........................................................................ 2 0 S03
................cuguaccgaaauacuuguagu....................................................................... 5 0 S03
................cuguaccgaaauacuuguagu....................................................................... 5 0 S05
................cuguaccgaaauacuuguagu....................................................................... 2 0 S04
................cuguaccgaaauacuuguagu....................................................................... 3 0 S06
.................uguaccgaaauacuuguag........................................................................ 12 0 S01
.................uguaccgaaauacuuguag........................................................................ 28 0 S06
.................uguaccgaaauacuuguag........................................................................ 9 0 S02
.................uguaccgaaauacuuguag........................................................................ 7 0 S05
.................uguaccgaaauacuuguag........................................................................ 8 0 S03
.................uguaccgaaauacuuguag........................................................................ 9 0 S04
.................uguaccgaaauacuuguagu....................................................................... 13 0 S03
.................uguaccgaaauacuuguagu....................................................................... 22 0 S05
.................uguaccgaaauacuuguagu....................................................................... 35 0 S01
.................uguaccgaaauacuuguagu....................................................................... 54 0 S06
.................uguaccgaaauacuuguagu....................................................................... 13 0 S04
.................uguaccgaaauacuuguagu....................................................................... 37 0 S02
.................uguaccgaaauacuuguaguc...................................................................... 2 0 S02
.................uguaccgaaauacuuguaguc...................................................................... 1 0 S03
.................uguaccgaaauacuuguaguc...................................................................... 2 0 S05
..................guaccgaaauacuuguag........................................................................ 4 0 S06
..................guaccgaaauacuuguagu....................................................................... 1 0 S05
..................guaccgaaauacuuguagu....................................................................... 1 0 S01
..................guaccgaaauacuuguagu....................................................................... 2 0 S06
..................guaccgaaauacuuguagu....................................................................... 1 0 S03
...................uaccgaaauacuuguagu....................................................................... 10 0 S06
...................uaccgaaauacuuguagu....................................................................... 4 0 S04
...................uaccgaaauacuuguagu....................................................................... 5 0 S01
...................uaccgaaauacuuguagu....................................................................... 5 0 S05
...................uaccgaaauacuuguagu....................................................................... 4 0 S03
...................uaccgaaauacuuguagu....................................................................... 2 0 S02
....................accgaaauacuuguaguc...................................................................... 1 0 S02
....................accgaaauacuuguagucgag................................................................... 1 0 S06
................................................aaguauuucgguacagaggga....................................... 1 0 S04
................................................aaguauuucgguacagaggga....................................... 1 0 S05
................................................aaguauuucgguacagaggga....................................... 1 0 S02
.................................................aguauuucgguacagaggga....................................... 1 0 S06
.................................................aguauuucgguacagaggga....................................... 1 0 S02
.................................................aguauuucgguacagaggga....................................... 2 0 S03
.................................................aguauuucgguacagagggag...................................... 2 0 S06
.................................................aguauuucgguacagagggag...................................... 1 0 S05
.................................................aguauuucgguacagagggagu..................................... 1 0 S06
.................................................aguauuucgguacagagggagu..................................... 1 0 S02
.................................................aguauuucgguacagagggagu..................................... 1 0 S05
.................................................aguauuucgguacagagggagu..................................... 1 0 S01
.................................................aguauuucgguacagagggaguagu.................................. 1 0 S03
...................................................uauuucgguacagagggag...................................... 1 0 S02
...................................................uauuucgguacagagggagu..................................... 1 0 S03
...................................................uauuucgguacagagggagu..................................... 1 0 S02
...................................................uauuucgguacagagggagu..................................... 1 0 S05
...................................................uauuucgguacagagggagu..................................... 1 0 S06
...................................................uauuucgguacagagggagua.................................... 6 0 S02
...................................................uauuucgguacagagggagua.................................... 5 0 S05
...................................................uauuucgguacagagggagua.................................... 3 0 S03
...................................................uauuucgguacagagggagua.................................... 4 0 S04
...................................................uauuucgguacagagggagua.................................... 3 0 S06
...................................................uauuucgguacagagggagua.................................... 5 0 S01
....................................................auuucgguacagagggagu..................................... 2 0 S05
....................................................auuucgguacagagggagua.................................... 2 0 S05
....................................................auuucgguacagagggagua.................................... 1 0 S03
....................................................auuucgguacagagggagua.................................... 1 0 S06
....................................................auuucgguacagagggaguag................................... 1 0 S05
.....................................................uuucgguacagagggagua.................................... 2 0 S06
.....................................................uuucgguacagagggaguagu.................................. 1 0 S02
......................................................uucgguacagagggaguaguagcc.............................. 1 0 S05
......................................................uucgguacagagggaguaguagcc.............................. 1 0 S06
.........................................................................uagccaucacuaaucuuauucaug........... 1 0 S06
..............................................................................aucacuaaucuuauucaugaacg....... 1 0 S06
```

Provisional ID	:	ta_iwgsc_2bs_v1_5189854_1669566
Score total	:	2.5
Score for star read(s)	:	-1.3
Score for read counts	:	0
Score for mfe	:	2.2
Score for randfold	:	1.6
Score for cons. seed	:	
Total read count	:	133
Mature read count	:	152
Loop read count	:	0
Star read count	:	64

novel-m0842-3p

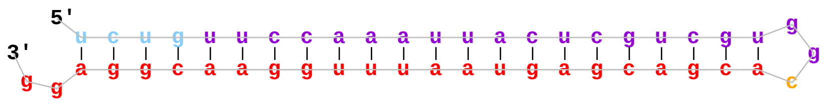

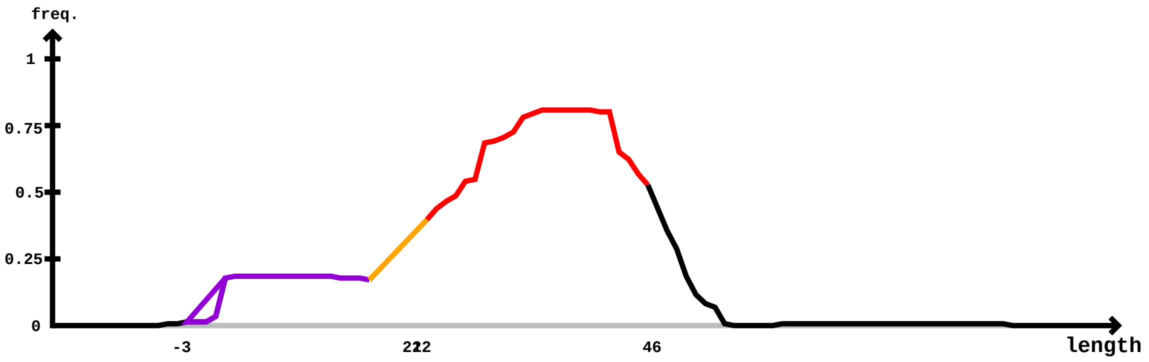

Star Mature

```
5'- uaacguacugccucuguuccaaauuacucgucguggcacgacgaguaauuuggaacggagggaguaucaauuaacuaucaugucuaauucuuaagcgcuacaguuuuau  -3'  obs
    uaacguacugccucuguuccaaauuacucgucguggcacgacgaguaauuuggaacggagggaguaucaauuaacuaucaugucuaauucuuaagcgcuacaguuuuau       exp
    ....(((((.((((((((((((((((((((((((...))))))))))))))))))))))))).))))).(((((((......)).)))))...((((......))))...  reads  mm  sample
    ..........ccucuguuccaaauuacu.................................................................................  1  0  S04
    ............ucuguuccaaauuacucgu..............................................................................  1  0  S06
    ...............guuccaaauuacucgucgug..........................................................................  1  0  S02
    ...............guuccaaauuacucgucgugg.........................................................................  1  0  S05
    ...............guuccaaauuacucgucgugg.........................................................................  1  0  S03
    ................uuccaaauuacucgucgug..........................................................................  1  0  S06
    ................uuccaaauuacucgucgug..........................................................................  1  0  S05
    ................uuccaaauuacucgucgugg.........................................................................  1  0  S01
    ................uuccaaauuacucgucgugg.........................................................................  6  0  S05
    ................uuccaaauuacucgucgugg.........................................................................  2  0  S04
    ................uuccaaauuacucgucgugg.........................................................................  1  0  S02
    ................uuccaaauuacucgucguggc........................................................................  1  0  S01
    ................uuccaaauuacucgucguggc........................................................................  2  0  S02
    ................uuccaaauuacucgucguggc........................................................................  5  0  S05
    ................uuccaaauuacucgucguggc........................................................................  1  0  S04
    .................uccaaauuacucgucgug..........................................................................  1  0  S03
    ....................................cacgacgaguaauuuggaacg....................................................  1  0  S04
    ....................................cacgacgaguaauuuggaacgga..................................................  2  0  S02
    ....................................cacgacgaguaauuuggaacggag.................................................  2  0  S06
    ....................................cacgacgaguaauuuggaacggag.................................................  2  0  S04
    .....................................acgacgaguaauuuggaa......................................................  1  0  S05
    .....................................acgacgaguaauuuggaacg....................................................  3  0  S03
    .....................................acgacgaguaauuuggaacg....................................................  5  0  S06
    .....................................acgacgaguaauuuggaacg....................................................  1  0  S04
    .....................................acgacgaguaauuuggaacg....................................................  4  0  S05
    .....................................acgacgaguaauuuggaacg....................................................  5  0  S02
    .....................................acgacgaguaauuuggaacg....................................................  2  0  S01
    .....................................acgacgaguaauuuggaacgg...................................................  1  0  S05
    .....................................acgacgaguaauuuggaacgg...................................................  1  0  S01
    .....................................acgacgaguaauuuggaacgg...................................................  1  0  S02
    .....................................acgacgaguaauuuggaacgga..................................................  1  0  S03
    .....................................acgacgaguaauuuggaacgga..................................................  1  0  S04
    .....................................acgacgaguaauuuggaacggag.................................................  1  0  S05
```

Star **Mature**

```
uaacguacugccucuguuccaaauuacucgucgugg cacgacgaguaauuuggaacggagggaguaucaauuaacuaucaugucuaauucuuaagcgcuacaguuuuau
.....................................acgacgaguaauuuggaacggag.................................................   1    0   S03
.....................................acgacgaguaauuuggaacggagg................................................   2    0   S06
.....................................acgacgaguaauuuggaacggagg................................................   11   0   S04
.....................................acgacgaguaauuuggaacggagg................................................   8    0   S05
.....................................acgacgaguaauuuggaacggagg................................................   2    0   S02
......................................cgacgaguaauuuggaacg....................................................   1    0   S03
......................................cgacgaguaauuuggaacgg...................................................   1    0   S04
......................................cgacgaguaauuuggaacgga..................................................   1    0   S05
......................................cgacgaguaauuuggaacgga..................................................   1    0   S06
......................................cgacgaguaauuuggaacgga..................................................   2    0   S02
.......................................gacgaguaauuuggaacggaggga..............................................   4    0   S05
........................................acgaguaauuuggaacggaggg...............................................   1    0   S02
........................................acgaguaauuuggaacggagggag.............................................   2    0   S04
.........................................cgaguaauuuggaacggagggagu............................................   3    0   S02
.........................................cgaguaauuuggaacggagggagu............................................   1    0   S05
.........................................cgaguaauuuggaacggagggagu............................................   2    0   S04
.........................................cgaguaauuuggaacggagggagu............................................   1    0   S01
.........................................cgaguaauuuggaacggagggagua...........................................   1    0   S01
..........................................gaguaauuuggaacggagggagua...........................................   1    0   S04
...........................................aguaauuuggaacggaggg...............................................   1    0   S06
...........................................aguaauuuggaacggaggga..............................................   2    0   S01
...........................................aguaauuuggaacggaggga..............................................   1    0   S06
...........................................aguaauuuggaacggaggga..............................................   2    0   S05
...........................................aguaauuuggaacggagggag.............................................   6    0   S05
...........................................aguaauuuggaacggagggag.............................................   1    0   S06
...........................................aguaauuuggaacggagggag.............................................   1    0   S03
...........................................aguaauuuggaacggagggag.............................................   2    0   S01
...........................................aguaauuuggaacggagggag.............................................   1    0   S02
...........................................aguaauuuggaacggagggag.............................................   2    0   S04
...........................................aguaauuuggaacggagggagua...........................................   1    0   S04
............................................guaauuuggaacggaggga..............................................   1    0   S03
.............................................uaauuuggaacggagggagu............................................   1    0   S06
.............................................uaauuuggaacggagggagu............................................   1    0   S02
..............................................aauuuggaacggagggagu............................................   1    0   S06
..............................................aauuuggaacggagggaguauc.........................................   1    0   S06
..............................................aauuuggaacggagggaguauc.........................................   1    0   S02
...............................................auuuggaacggagggagua...........................................   2    0   S05
...............................................auuuggaacggagggaguau..........................................   1    0   S02
...............................................auuuggaacggagggaguauc.........................................   2    0   S03
...............................................auuuggaacggagggaguauc.........................................   1    0   S06
...............................................auuuggaacggagggaguauc.........................................   2    0   S01
................................................uuuggaacggagggaguauc.........................................   1    0   S02
................................................uuuggaacggagggaguauca........................................   1    0   S03
.................................................uuggaacggagggaguau..........................................   1    0   S04
.................................................uuAgaacggagggaguauc.........................................   1    1   S02
..........................................................................cuaucaugucuaauucuuaagcgc...........   1    0   S02
```

Provisional ID	:	ta_iwgsc_5dl_v1_4590987_5407719
Score total	:	4090.1
Score for star read(s)	:	3.9
Score for read counts	:	4082.8
Score for mfe	:	1.8
Score for randfold	:	1.6
Score for cons. seed	:	
Total read count	:	8020
Mature read count	:	8098
Loop read count	:	0
Star read count	:	1

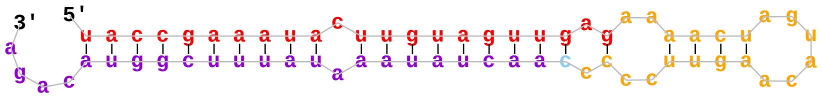

novel-m621-5p

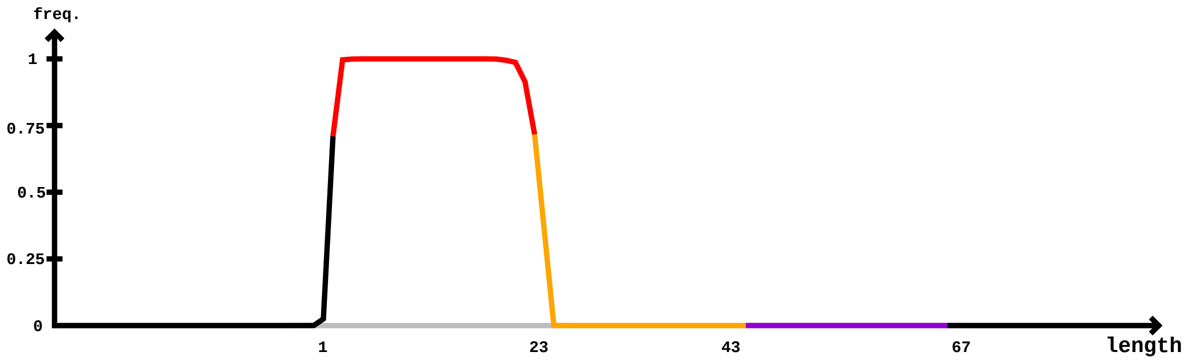

Mature   Star

```
5'- uguucuuuuucguauauacucccuccguaccgaaauacuuguaguugagaaaacuaguacaaguucccccaacuauaaaauauuucgguacagaggugguauaagacaaucagc  -3'   obs
    uguucuuuuucguauauacucccuccguaccgaaauacuuguaguugagaaaacuaguacaaguucccccaacuauaaaauauuucgguacagaggugguauaagacaaucagc         exp
    ..........((.((((((.((((.(((((((((((.((((((((.(..((((......))))..).))))))))).))))))))))).)))).))))))..)).......        reads   mm   sample
    .......................uccguaccgaaauacuuguaguu...................................................................    1       0    S05
    .........................cguaccgaaauacuuguag.....................................................................    1       0    S06
    ..........................guaccgaaauacuuguag.....................................................................    4       0    S06
    ..........................guaccgaaauacuuguagu....................................................................    2       0    S06
    ..........................guaccgaaauacuuguagu....................................................................    1       0    S01
    ..........................guaccgaaauacuuguagu....................................................................    1       0    S05
    ..........................guaccgaaauacuuguagu....................................................................    1       0    S03
    ..........................guaccgaaauacuuguaguu...................................................................    1       0    S02
    ..........................guaccgaaauacuuguaguu...................................................................    2       0    S04
    ..........................guaccgaaauacuuguaguu...................................................................    1       0    S06
    ..........................guaccgaaauacuuguaguu...................................................................    1       0    S05
    ..........................guaccgaaauacuuguaguug..................................................................    7       0    S04
    ..........................guaccgaaauacuuguaguug..................................................................    4       0    S02
    ..........................guaccgaaauacuuguaguug..................................................................    9       0    S03
    ..........................guaccgaaauacuuguaguug..................................................................    17      0    S06
    ..........................guaccgaaauacuuguaguug..................................................................    7       0    S05
    ..........................guaccgaaauacuuguaguug..................................................................    9       0    S01
    ..........................guaccgaaauacuuguaguuga.................................................................    9       0    S02
    ..........................guaccgaaauacuuguaguuga.................................................................    16      0    S06
    ..........................guaccgaaauacuuguaguuga.................................................................    7       0    S05
    ..........................guaccgaaauacuuguaguuga.................................................................    3       0    S03
    ..........................guaccgaaauacuuguaguuga.................................................................    8       0    S01
    ..........................guaccgaaauacuuguaguugU.................................................................    1       1    S03
    ..........................guaccgaaauacuuguaguuga.................................................................    7       0    S04
    ..........................guaccgaaauacuuguaguugag................................................................    9       0    S01
    ..........................guaccgaaauacuuguaguugag................................................................    3       0    S04
    ..........................guaccgaaauacuuguaguugag................................................................    10      0    S05
    ..........................guaccgaaauacuuguaguugag................................................................    11      0    S03
    ..........................guaccgaaauacuuguaguugag................................................................    35      0    S06
    ..........................guaccgaaauacuuguaguugag................................................................    11      0    S02
    ..........................guaccgaaauacuuguaguugaga...............................................................    1       0    S06
    ..........................guaccgaaauacuuguaguugaga...............................................................    1       0    S05
    ...........................uaccgaaauacuuguagu....................................................................    10      0    S06
```

```
                         Mature                                     Star
uguucuuuucguauauacuccuccguaccgaaauacuuguaguugagaaaacuaguacaaguucccccaacuauaaauauuucgguacagaggugguauaagacaaucagc
.........................uaccgaaauacuuguagu....................................................................   4     0   S03
.........................uaccgaaauacuuguagu....................................................................   4     0   S04
.........................uaccgaaauacuuguagu....................................................................   5     0   S01
.........................uaccgaaauacuuguagu....................................................................   5     0   S05
.........................uaccgaaauacuuguagu....................................................................   2     0   S02
.........................uaccgaaauacuuguaguu...................................................................   1     0   S03
.........................uaccgaaauacuuguaguu...................................................................   6     0   S06
.........................uaccgaaauacuuguaguu...................................................................   6     0   S02
.........................uaccgaaauacuuguaguu...................................................................   7     0   S05
.........................uaccgaaauacuuguaguu...................................................................   11    0   S01
.........................uaccgaaauacuuguaguu...................................................................   2     0   S04
.........................uaccgaaauacuuguaguug..................................................................   65    0   S02
.........................uaccgaaauacuuguaguug..................................................................   61    0   S01
.........................uaccgaaauacuuguaguug..................................................................   121   0   S06
.........................uaccgaaauacuuguaguug..................................................................   37    0   S04
.........................uaccgaaauacuuguaguug..................................................................   92    0   S03
.........................uaccgaaauacuuguaguug..................................................................   49    0   S05
.........................uaccgaaauacuuguaguuAa.................................................................   1     1   S02
.........................uaccgaaauacuuguaguuga.................................................................   240   0   S05
.........................uaccgaaauacuuguaguuga.................................................................   248   0   S01
.........................uaccgaaauacuuAuaguuga.................................................................   1     1   S04
.........................uaccgaaauacuuguaguuga.................................................................   134   0   S03
.........................uaccgaaauacuuguaguuga.................................................................   215   0   S02
.........................uaccgaaauacuuguaguugU.................................................................   4     1   S05
.........................uaccgaaauacuuguaguuga.................................................................   145   0   S04
.........................uaccgaaauacuuguaguuAa.................................................................   1     1   S06
.........................uaccgaaauacuuguaguuAa.................................................................   2     1   S01
.........................uaccgaaauacuuguaguugU.................................................................   2     1   S01
.........................uaccgaaauacuuguaguugU.................................................................   3     1   S02
.........................uaccgaaauacuuguaguuga.................................................................   314   0   S06
.........................uaccgaaauacuuguaguuAag................................................................   2     1   S04
.........................uaccgaaauacuuguaguugag................................................................   996   0   S06
.........................uaccgaaauacuuAuaguugag................................................................   1     1   S02
.........................uaccgaaauacuuguaguugag................................................................   491   0   S01
.........................uaccgaaauacuuguaguugag................................................................   629   0   S02
.........................uaccgaaauacuuguaguuAag................................................................   3     1   S05
.........................uaccgaaauacuuAuaguugag................................................................   1     1   S06
.........................uaccgaaauacuuguaguugag................................................................   778   0   S05
.........................uaccgaaauacuuguaguugag................................................................   447   0   S04
.........................uaccgaaauacuuguaguuAag................................................................   1     1   S01
.........................uaccgaaauacuuguaguugag................................................................   340   0   S03
.........................uaccgaaauacuuguaguugaga...............................................................   2     0   S05
.........................uaccgaaauacuuguaguugaga...............................................................   4     0   S04
.........................uaccgaaauacuuguaguugaga...............................................................   1     0   S01
.........................uaccgaaauacuuguaguugaga...............................................................   2     0   S06
..........................accgaaauacuuguaguu...................................................................   2     0   S02
..........................accgaaauacuuguaguu...................................................................   2     0   S04
..........................accgaaauacuuguaguu...................................................................   7     0   S03
..........................accgaaauacuuguaguu...................................................................   6     0   S06
..........................accgaaauacuuguaguu...................................................................   5     0   S01
..........................accgaaauacuuguaguu...................................................................   4     0   S05
..........................accgaaauacuuguaguug..................................................................   3     0   S04
..........................accgaaauacuuguaguug..................................................................   5     0   S05
..........................accgaaauacuuguaguug..................................................................   42    0   S06
..........................accgaaauacuuguaguug..................................................................   4     0   S01
..........................accgaaauacuuguaguug..................................................................   29    0   S02
..........................accgaaauacuuguaguug..................................................................   16    0   S03
..........................accgaaauacuuguaguuga.................................................................   36    0   S02
..........................accgaaauacuuguaguuga.................................................................   21    0   S03
..........................accgaaauacuuguaguuga.................................................................   95    0   S06
..........................accgaaauacuuguaguuga.................................................................   13    0   S04
..........................accgaaauacuuguaguuga.................................................................   13    0   S05
..........................accgaaauacuuguaguugU.................................................................   1     1   S03
..........................accgaaauacuuguaguugU.................................................................   1     1   S02
..........................accgaaauacuuguaguuga.................................................................   44    0   S01
..........................accgaaauacuuguaguugU.................................................................   1     1   S01
..........................accgaaauacuuguaguugag................................................................   130   0   S04
..........................accgaaauacuuAuaguugag................................................................   1     1   S06
..........................accgaaauacuuguaguuAag................................................................   1     1   S06
..........................accgaaauacuuguaguugag................................................................   264   0   S01
```

Mature Star

```
uguucuuuucguauauacucccuccguaccgaaauacuuguaguugagaaaacuaguacaaguucccccaacuauaaauauuucgguacagaggugguauaagacaaucagc
...........................accgaaauacuuguaguugag................................................................ 733 0 S06
...........................accgaaauacuuguaguuAag................................................................ 1 1 S02
...........................accgaaauacuuguaguugag................................................................ 138 0 S03
...........................accgaaauacuuguaguugag................................................................ 331 0 S02
...........................accgaaauacuuguaguugag................................................................ 344 0 S05
...........................accgaaauacuuguaguugaga............................................................... 1 0 S01
...........................accgaaauacuuguaguugaga............................................................... 1 0 S03
...........................accgaaauacuuguaguugagaaa............................................................. 1 0 S06
............................ccgaaauacuuguaguug.................................................................. 1 0 S06
............................ccgaaauacuuguaguug.................................................................. 1 0 S05
............................ccgaaauacuuguaguug.................................................................. 1 0 S02
............................ccgaaauacuuguaguugag................................................................ 1 0 S05
............................ccgaaauacuuguaguugag................................................................ 2 0 S04
............................ccgaaauacuuguaguugag................................................................ 4 0 S01
............................ccgaaauacuuguaguugag................................................................ 5 0 S06
............................ccgaaauacuuguaguugag................................................................ 3 0 S03
............................ccgaaauacuuguaguugag................................................................ 4 0 S02
.............................cgaaauacuuguaguuga................................................................. 1 0 S06
.............................cgaaauacuuguaguugag................................................................ 1 0 S03
.............................cgaaauacuuguaguugag................................................................ 1 0 S01
.............................cgaaauacuuguaguugag................................................................ 1 0 S06
.............................cgaaauacuuguaguugaAaa.............................................................. 1 1 S02
.....................................................................aacuauaaauauuucgguacaga.................... 1 0 S01
```

Provisional ID	: ta_iwgsc_2dl_v1_1240258_1791638		novel-m0621-5p
Score total	:	2.6	
Score for star read(s)	:	-1.3	
Score for read counts	:	0	
Score for mfe	:	2.3	
Score for randfold	:	1.6	
Score for cons. seed	:		
Total read count	:	9153	
Mature read count	:	8087	
Loop read count	:	0	
Star read count	:	0	

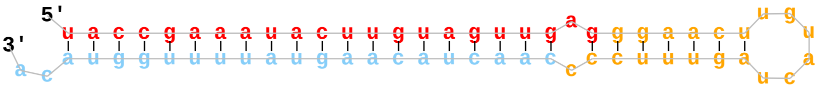

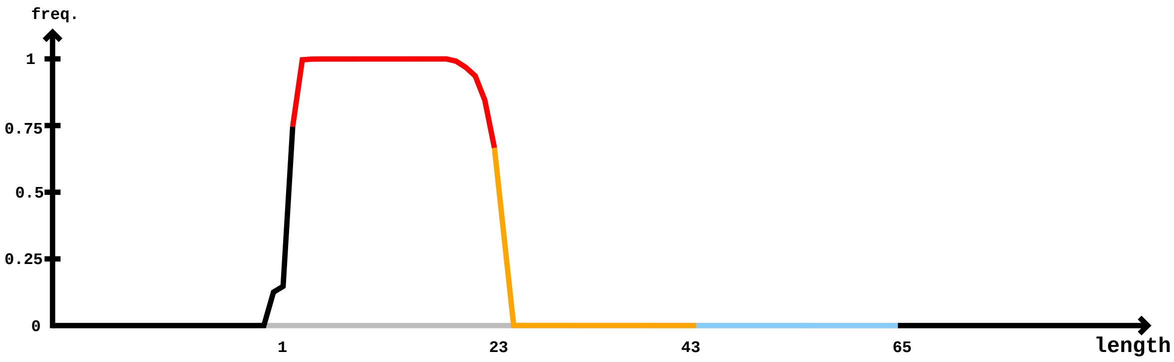

Mature **Star**

5'- caugaaauaauguacucacuauguaccgaaauacuuguaguugagggaacuuguacuaguuuccccaacuacaaguauuuugguacagagggaguaguggacaugaaccuca -3'	exp		
((((........(((((.((.((((((((((((((((((((((.(((((((......))))))).)))))))))))))))))))))).)).))))).....))))).......	reads	mm	sample
....................auguaccgaaauacuuguaguu..	1	0	S01
.....................uguaccgaaauacuuguag..	28	0	S06
.....................uguaccgaaauacuuguag..	12	0	S01
.....................uguaccgaaauacuuguag..	9	0	S04
.....................uguaccgaaauacuuguag..	9	0	S02
.....................uguaccgaaauacuuguag..	7	0	S05
.....................uguaccgaaauacuuguag..	8	0	S03
.....................uguaccgaaauacuuguagu...	54	0	S06
.....................uguaccgaaauacuuguagu...	13	0	S04
.....................uguaccgaaauacuuguagu...	22	0	S05
.....................uguaccgaaauacuuguagu...	35	0	S01
.....................uguaccgaaauacuuguagu...	13	0	S03
.....................uguaccgaaauacuuguagu...	37	0	S02
.....................uguaccgaaauacuuguaguu..	48	0	S02
.....................uguaccgaaauacuuguaguu..	44	0	S06
.....................uguaccgaaauacuuguaguu..	28	0	S04
.....................uguaccgaaauacuuguaguu..	41	0	S05
.....................uguaccgaaauacuuguaguu..	46	0	S01
.....................uguaccgaaauacuuguaguu..	26	0	S03
.....................uguaccgaaauacuuguaguug...	25	0	S03
.....................uguaccgaaauacuuguaguug...	42	0	S02
.....................uguaccgaaauacuuguaguug...	56	0	S01
.....................uguaccgaaauacuuguaguug...	35	0	S04
.....................uguaccgaaauacuuguaguug...	49	0	S06
.....................uguaccgaaauacuuguaguug...	48	0	S05
.....................uguaccgaaauacuuguaguuga..	10	0	S02
.....................uguaccgaaauacuuguaguuga..	3	0	S04
.....................uguaccgaaauacuuguaguuga..	2	0	S03
.....................uguaccgaaauacuuguaguuga..	10	0	S05
.....................uguaccgaaauacuuguaguugU..	1	1	S06
.....................uguaccgaaauacuuguaguugU..	1	1	S05
.....................uguaccgaaauacuuguaguuga..	7	0	S01
.....................uguaccgaaauacuuguaguuga..	16	0	S06
.....................uguaccgaaauacuuguaguugag...	42	0	S01

```
                       Mature                                     Star
caugaaauaauguacucacuauguaccgaaauacuuguaguugagggaacuuguacuaguuccccaacuacaaguauuuugguacagagggaguaguggacaugaaccuca
.....................uguaccgaaauacuuguaguugag.................................................................. 76 0 S06
.....................uguaccgaaauacuuguaguugag.................................................................. 70 0 S04
.....................uguaccgaaauacuuguaguugag.................................................................. 19 0 S03
.....................uguaccgaaauacuuguaguugag.................................................................. 87 0 S05
.....................uguaccgaaauacuuguaguugag.................................................................. 69 0 S02
......................guaccgaaauacuuguag....................................................................... 4 0 S06
......................guaccgaaauacuuguagu...................................................................... 1 0 S03
......................guaccgaaauacuuguagu...................................................................... 2 0 S06
......................guaccgaaauacuuguagu...................................................................... 1 0 S01
......................guaccgaaauacuuguagu...................................................................... 1 0 S05
......................guaccgaaauacuuguaguu..................................................................... 1 0 S02
......................guaccgaaauacuuguaguu..................................................................... 1 0 S06
......................guaccgaaauacuuguaguu..................................................................... 2 0 S04
......................guaccgaaauacuuguaguu..................................................................... 1 0 S05
......................guaccgaaauacuuguaguug.................................................................... 7 0 S05
......................guaccgaaauacuuguaguug.................................................................... 9 0 S03
......................guaccgaaauacuuguaguug.................................................................... 17 0 S06
......................guaccgaaauacuuguaguug.................................................................... 9 0 S01
......................guaccgaaauacuuguaguug.................................................................... 4 0 S02
......................guaccgaaauacuuguaguug.................................................................... 7 0 S04
......................guaccgaaauacuuguaguuga................................................................... 3 0 S03
......................guaccgaaauacuuguaguuga................................................................... 8 0 S01
......................guaccgaaauacuuguaguuga................................................................... 9 0 S02
......................guaccgaaauacuuguaguuga................................................................... 7 0 S04
......................guaccgaaauacuuguaguugU................................................................... 1 1 S03
......................guaccgaaauacuuguaguuga................................................................... 7 0 S05
......................guaccgaaauacuuguaguuga................................................................... 16 0 S06
......................guaccgaaauacuuguaguugag.................................................................. 3 0 S04
......................guaccgaaauacuuguaguugag.................................................................. 35 0 S06
......................guaccgaaauacuuguaguugag.................................................................. 11 0 S03
......................guaccgaaauacuuguaguugag.................................................................. 11 0 S02
......................guaccgaaauacuuguaguugag.................................................................. 10 0 S05
......................guaccgaaauacuuguaguugag.................................................................. 9 0 S01
.......................uaccgaaauacuuguagu...................................................................... 2 0 S02
.......................uaccgaaauacuuguagu...................................................................... 4 0 S04
.......................uaccgaaauacuuguagu...................................................................... 10 0 S06
.......................uaccgaaauacuuguagu...................................................................... 5 0 S01
.......................uaccgaaauacuuguagu...................................................................... 5 0 S05
.......................uaccgaaauacuuguagu...................................................................... 4 0 S03
.......................uaccgaaauacuuguaguu..................................................................... 7 0 S05
.......................uaccgaaauacuuguaguu..................................................................... 11 0 S01
.......................uaccgaaauacuuguaguu..................................................................... 1 0 S03
.......................uaccgaaauacuuguaguu..................................................................... 6 0 S06
.......................uaccgaaauacuuguaguu..................................................................... 6 0 S02
.......................uaccgaaauacuuguaguu..................................................................... 2 0 S04
.......................uaccgaaauacuuguaguug.................................................................... 37 0 S04
.......................uaccgaaauacuuguaguug.................................................................... 61 0 S01
.......................uaccgaaauacuuguaguug.................................................................... 92 0 S03
.......................uaccgaaauacuuguaguug.................................................................... 65 0 S02
.......................uaccgaaauacuuguaguug.................................................................... 121 0 S06
.......................uaccgaaauacuuguaguug.................................................................... 49 0 S05
.......................uaccgaaauacuuguaguuAa................................................................... 1 1 S06
.......................uaccgaaauacuuguaguuga................................................................... 314 0 S06
.......................uaccgaaauacuuguaguugU................................................................... 2 1 S01
.......................uaccgaaauacuuguaguuga................................................................... 240 0 S05
.......................uaccgaaauacuuguaguuga................................................................... 248 0 S01
.......................uaccgaaauacuuguaguugU................................................................... 3 1 S02
.......................uaccgaaauacuuguaguugU................................................................... 4 1 S05
.......................uaccgaaauacuuAuaguuga................................................................... 1 1 S04
.......................uaccgaaauacuuguaguuga................................................................... 215 0 S02
.......................uaccgaaauacuuguaguuAa................................................................... 2 1 S01
.......................uaccgaaauacuuguaguuga................................................................... 134 0 S03
.......................uaccgaaauacuuguaguuga................................................................... 145 0 S04
.......................uaccgaaauacuuguaguuAa................................................................... 1 1 S02
.......................uaccgaaauacuuAuaguugag.................................................................. 1 1 S06
.......................uaccgaaauacuuguaguuAag.................................................................. 3 1 S05
.......................uaccgaaauacuuguaguugag.................................................................. 778 0 S05
.......................uaccgaaauacuuguaguugag.................................................................. 996 0 S06
.......................uaccgaaauacuuguaguuAag.................................................................. 2 1 S04
.......................uaccgaaauacuuguaguuAag.................................................................. 1 1 S01
```

Mature Star

caugaaauaauguacucacuaug**uaccgaaauacuuguaguugag**ggaacuuguacuaguuuccc**caacuacaaguauuuugguaca**gagggaguaguggacaugaaccuca			
.......................uaccgaaauacuuguaguugag...	629	0	S02
.......................uaccgaaauacuuguaguugag...	491	0	S01
.......................uaccgaaauacuuAuaguugag...	1	1	S02
.......................uaccgaaauacuuguaguugag...	340	0	S03
.......................uaccgaaauacuuguaguugag...	447	0	S04
........................accgaaauacuuguaguu..	7	0	S03
........................accgaaauacuuguaguu..	6	0	S06
........................accgaaauacuuguaguu..	4	0	S05
........................accgaaauacuuguaguu..	2	0	S04
........................accgaaauacuuguaguu..	5	0	S01
........................accgaaauacuuguaguu..	2	0	S02
........................accgaaauacuuguaguug...	5	0	S05
........................accgaaauacuuguaguug...	29	0	S02
........................accgaaauacuuguaguug...	16	0	S03
........................accgaaauacuuguaguug...	42	0	S06
........................accgaaauacuuguaguug...	4	0	S01
........................accgaaauacuuguaguug...	3	0	S04
........................accgaaauacuuguaguugU..	1	1	S02
........................accgaaauacuuguaguugU..	1	1	S01
........................accgaaauacuuguaguuga..	13	0	S05
........................accgaaauacuuguaguuga..	95	0	S06
........................accgaaauacuuguaguuga..	13	0	S04
........................accgaaauacuuguaguuga..	36	0	S02
........................accgaaauacuuguaguuga..	21	0	S03
........................accgaaauacuuguaguugU..	1	1	S03
........................accgaaauacuuguaguuga..	44	0	S01
........................accgaaauacuuguaguugag...	130	0	S04
........................accgaaauacuuAuaguugag...	1	1	S06
........................accgaaauacuuguaguugag...	138	0	S03
........................accgaaauacuuguaguugag...	264	0	S01
........................accgaaauacuuguaguugag...	733	0	S06
........................accgaaauacuuguaguuAag...	1	1	S06
........................accgaaauacuuguaguugag...	344	0	S05
........................accgaaauacuuguaguuAag...	1	1	S02
........................accgaaauacuuguaguugag...	331	0	S02
........................accgaaauacuuguaguugagg..	1	0	S02
.........................ccgaaauacuuguaguug...	1	0	S05
.........................ccgaaauacuuguaguug...	1	0	S02
.........................ccgaaauacuuguaguug...	1	0	S06
.........................ccgaaauacuuguaguugag...	4	0	S02
.........................ccgaaauacuuguaguugag...	3	0	S03
.........................ccgaaauacuuguaguugag...	1	0	S05
.........................ccgaaauacuuguaguugag...	5	0	S06
.........................ccgaaauacuuguaguugag...	4	0	S01
.........................ccgaaauacuuguaguugag...	2	0	S04
.........................ccgaaauacuuguaguugagg..	1	0	S05
..........................cgaaauacuuguaguuga..	1	0	S06
..........................cgaaauacuuguaguugag...	1	0	S01
..........................cgaaauacuuguaguugag...	1	0	S06
..........................cgaaauacuuguaguugag...	1	0	S03
..............................auacuuguaguugagggaacuug...	1	0	S04
....................................guaguugagggaacuuguacuag...	1	0	S05
....uugagggaacuuguacuaguu...	1	0	S05
....uuuccccaacuacaaguU...................................	1	1	S04

Provisional ID : ta_iwgsc_5bl_v1_10795548_4773614

Score total : 127.8

Score for star read(s) : 3.9

Score for read counts : 119.4

Score for mfe : 2.8

Score for randfold : 1.6

Score for cons. seed :

Total read count : 246

Mature read count : 181

Loop read count : 1

Star read count : 30

novel-m0251-3p

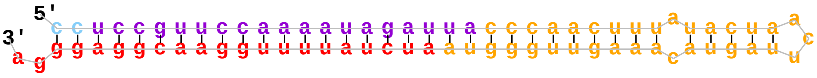

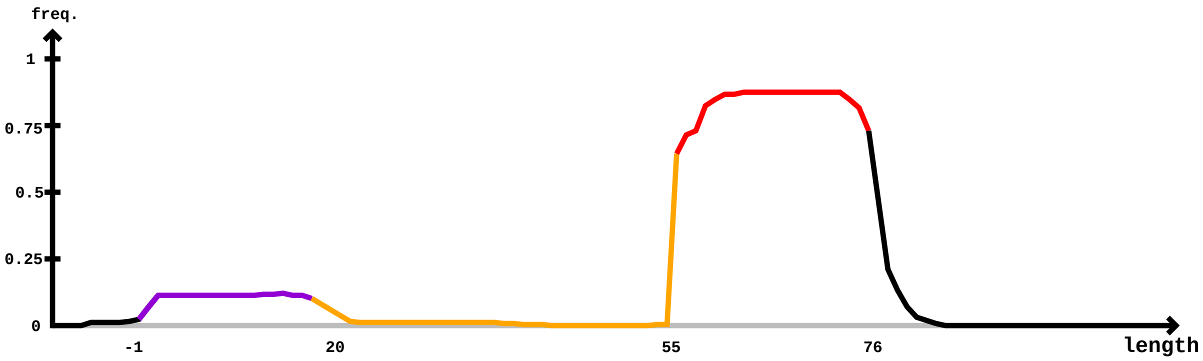

Star Mature

```
5'- uauacucccuccguuccaaaauagauuacccaacuuuauacuaacuuaguacaaaguuggguaaucuauuuuggaacggagggaguacgugcugauuuagauaugagaagcaugc  -3'   obs
    uauacucccuccguuccaaaauagauuacccaacuuuauacuaacuuaguacaaaguuggguaaucuauuuuggaacggagggaguacgugcugauuuagauaugagaagcaugc         exp
    ..(((((((((((((((((((((((((((((((((((.(((((...))))).)))))))))))))))))))))))))))))))))))((((((..((((....)))).)))))).  reads  mm  sample
    ..uacucccuccguuccaaaaua............................................................................................  1  0  S05
    ..uacucccuccguuccaaaaua............................................................................................  1  0  S02
    ..uacucccuccguuccaaaauaga.........................................................................................  1  0  S02
    ......cccuccguuccaaaauagau.........................................................................................  1  0  S02
    .......ccuccguuccaaaauaga..........................................................................................  1  0  S04
    .......ccuccguuccaaaauaga..........................................................................................  1  0  S01
    ........cuccguuccaaaauagau.........................................................................................  2  0  S02
    ........cuccguuccaaaauagau.........................................................................................  1  0  S03
    ........cuccguuccaaaauagauu........................................................................................  1  0  S01
    ........cuccguuccaaaauagauu........................................................................................  1  0  S04
    ........cuccguuccaaaauagauuU.......................................................................................  1  1  S03
    ........cuccguuccaaaauagauuU.......................................................................................  2  1  S05
    ........cuccguuccaaaauagauuac......................................................................................  1  0  S02
    ........cuccguuccaaaauagauuac......................................................................................  2  0  S05
    ........cuccguuccaaaauagauuac......................................................................................  1  0  S01
    .........uccguuccaaaauagauu........................................................................................  1  0  S01
    .........uccguuccaaaauagauu........................................................................................  1  0  S03
    .........uccguuccaaaauagauuU.......................................................................................  3  1  S01
    .........uccguuccaaaauagauuU.......................................................................................  4  1  S05
    .........uccguuccaaaauagauuU.......................................................................................  1  1  S04
    .........uccguuccaaaauagauuacc.....................................................................................  1  0  S05
    ....................auagauuacccaacuuuauacuaac......................................................................  1  0  S05
    ......................agauuacccaacuuuauacuaacuu....................................................................  1  0  S04
    ...........................acccaacuuuauacuaacuuagu.................................................................  1  0  S02
    .............................................................uaaucuauuuuggaacggagg.................................  1  0  S06
    ...............................................................aucuauuuuggaacggag..................................  3  0  S05
    ...............................................................aucuauuuuggaacggag..................................  3  0  S06
    ...............................................................aucuauuuuggaacggag..................................  1  0  S04
    ...............................................................aucuauuuuggaacggagg.................................  2  0  S02
    ...............................................................aucuauuuuggaacggagg.................................  2  0  S04
    ...............................................................aucuauuuuggaacggagg.................................  2  0  S06
    ...............................................................aucuauuuuggaacggaggg................................  5  0  S02
    ...............................................................aucuauuuuggaacggaggg................................  3  0  S05
```

Star **Mature**

```
uauacucccuccguuccaaaauagauuacccaacuuuauacuaacuuaguacaaaguuggguaaucuauuuuggaacggagggaguacgugcugauuuagauaugagaagcaugc
...............................................................aucuauuuuggaacggaggg................................ 4 0 S06
...............................................................aucuauuuuggaacggaggg................................ 2 0 S01
...............................................................aucuauuuuggaacggaggg................................ 5 0 S03
...............................................................aucuauuuuggaacggaggg................................ 2 0 S04
...............................................................aucuauuuuggaacggaggga............................... 9 0 S01
...............................................................aucuauuuuggaacggaggga............................... 38 0 S05
...............................................................aucuauuuuggaacggaggga............................... 20 0 S06
...............................................................aucuauuuuggaacggaggga............................... 25 0 S04
...............................................................aucuauuuuggaacggaggga............................... 5 0 S03
...............................................................aucuauuuuggaacggaggga............................... 26 0 S02
...............................................................aucuauuuuggaacggagggag.............................. 1 0 S04
...............................................................aucuauuuuggaacggagggagu............................. 1 0 S04
...............................................................aucuauuuuggaacggagggagu............................. 1 0 S05
...............................................................aucuauuuuggaacggagggagu............................. 1 0 S06
...............................................................aucuauuuuggaacggagggagu............................. 1 0 S03
...............................................................aucuauuuuggaacggagggagua............................ 1 0 S05
...............................................................aucuauuuuggaacggagggagua............................ 1 0 S04
................................................................ucuauuuuggaacggagg................................. 1 0 S04
................................................................ucuauuuuggaacggaggg................................ 1 0 S04
................................................................ucuauuuuggaacggaggga............................... 1 0 S02
................................................................ucuauuuuggaacggaggga............................... 1 0 S04
................................................................ucuauuuuggaacggagggag.............................. 2 0 S05
................................................................ucuauuuuggaacggagggag.............................. 3 0 S04
................................................................ucuauuuuggaacggagggag.............................. 1 0 S02
................................................................ucuauuuuggaacggagggag.............................. 1 0 S03
................................................................ucuauuuuggaacggagggagu............................. 2 0 S02
................................................................ucuauuuuggaacggagggagu............................. 1 0 S05
................................................................ucuauuuuggaacggagggagu............................. 1 0 S04
................................................................ucuauuuuggaacggagggUguac........................... 1 1 S02
................................................................ucuauuuuggaacggagggaguac........................... 1 0 S05
................................................................ucuauuuuggaacggagggagCac........................... 1 1 S04
.................................................................cuauuuuggaacggagggagu............................. 3 0 S01
.................................................................cuauuuuggaacggagggagua............................ 1 0 S01
..................................................................uauuuuggaacggagggagu............................. 1 0 S02
..................................................................uauuuuggaacggagggagu............................. 3 0 S05
..................................................................uauuuuggaacggagggagu............................. 1 0 S06
..................................................................uauuuuggaacggagggagu............................. 4 0 S04
..................................................................uauuuuggaacggagggagua............................ 3 0 S04
..................................................................uauuuuggaacggagggagua............................ 1 0 S03
..................................................................uauuuuggaacggagggagua............................ 1 0 S01
..................................................................uauuuuggaacggagggagua............................ 4 0 S02
..................................................................uauuuuggaacggagggagua............................ 1 0 S06
..................................................................uauuuuggaacggagggagua............................ 1 0 S05
..................................................................uauuuuggaacggagggaguac........................... 1 0 S04
..................................................................uauuuuggaacggagggaguacgu......................... 3 0 S04
...................................................................auuuuggaacggagggagua............................ 1 0 S03
...................................................................auuuuggaacggagggagua............................ 1 0 S01
...................................................................auuuuggaacggagggaguac........................... 1 0 S03
...................................................................auuuuggaacggagggaguac........................... 1 0 S01
...................................................................auuuuggaacggagggaguac........................... 1 0 S05
...................................................................auuuuggaacggagggaUuac........................... 1 1 S03
....................................................................uuuuCgaacggagggaguacg.......................... 2 1 S05
....................................................................uuuuggaacggagggagCacg.......................... 1 1 S05
....................................................................uuuugAaacggagggaguacgug........................ 1 1 S04
....................................................................uuuuggaacggagggaguacgug........................ 1 0 S04
......................................................................uuggaacggagggaguac........................... 2 0 S04
```

Provisional ID	:	ta_iwgsc_2dl_v1_9753892_1869976
Score total	:	2.2
Score for star read(s)	:	-1.3
Score for read counts	:	0
Score for mfe	:	1.9
Score for randfold	:	1.6
Score for cons. seed	:	
Total read count	:	152
Mature read count	:	671
Loop read count	:	0
Star read count	:	133

novel-m0360-3p

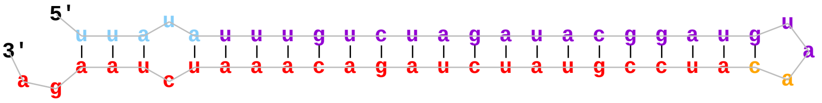

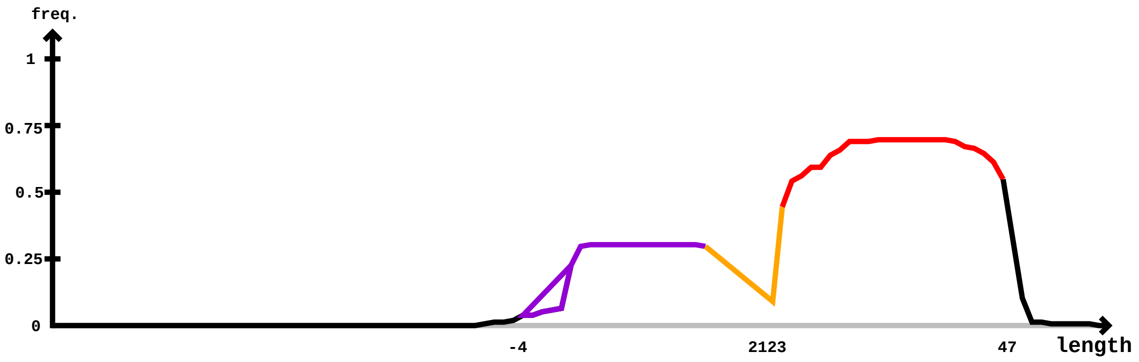

Star Mature

```
5'- cacccuuggucucuguugcauacuccuuccauccugaaaaguuuugucuuauauuugucuagauacggauguaacauccguaucuagacaaaucuaagacaaaauuuuc  -3'   obs
    cacccuuggucucuguugcauacuccuuccauccugaaaaguuuugucuuauauuugucuagauacggauguaacauccguaucuagacaaaucuaagacaaaauuuuc         exp
    ......(((.....((.....)).....)))....(((((.(((((((((.(((((((((((((((((((...))))))))))))))))))).))))))))).)))))   reads   mm   sample
    ............................................ugucuuauauuugucuagauacgg.........................................   1   0   S02
    .............................................gucuuauauuugucuagauacgg.........................................   1   0   S03
    ...............................................cuuauauuugucuagauacggaug......................................   1   0   S05
    ................................................uuauauuugucuagauacg..........................................   1   0   S02
    ................................................uuauauuugucuagauacgga........................................   1   0   S05
    ................................................uuauauuugucuagauacggaug......................................   1   0   S01
    ..................................................auauuugucuagauacgg.........................................   1   0   S04
    ..................................................auauuugucuagauacggaug......................................   1   0   S03
    ...................................................uauuugucuagauacgga........................................   1   0   S03
    ....................................................auuugucuagauacggaugua....................................   1   0   S05
    .....................................................uuugucuagauacggaug......................................   3   0   S05
    .....................................................uuugucuagauacggaug......................................   1   0   S04
    .....................................................uuugucuagauacggaug......................................   3   0   S01
    .....................................................uuugucuagauacggaugu.....................................   3   0   S01
    .....................................................uuugucuagauacggaugu.....................................   1   0   S03
    .....................................................uuugucuagauacggaugua....................................   3   0   S04
    .....................................................uuugucuagauacggaugua....................................   4   0   S02
    .....................................................uuugucuagauacggaugua....................................   1   0   S06
    .....................................................uuugucuagauacggaugua....................................   4   0   S01
    .....................................................uuugucuagauacggaugua....................................   1   0   S05
    .....................................................uuugucuagauacggaugua....................................   1   0   S03
    ......................................................uugucuagauacggaugu.....................................   1   0   S05
    ......................................................uugucuagauacggaugu.....................................   3   0   S04
    ......................................................uugucuagauacggauguC....................................   1   1   S04
    ......................................................uugucuagauacggaugua....................................   1   0   S01
    ......................................................uugucuagauacggauguC....................................   1   1   S05
    ......................................................uugucuagauacggaugua....................................   2   0   S04
    ......................................................uugucuagauacggaugua....................................   1   0   S05
    ......................................................uugucuagauacggauguaac..................................   1   0   S04
    .......................................................ugucuagauacggaugua....................................   1   0   S04
    .........................................................................acauccguaucuagacaaauc...............   1   0   S03
    .........................................................................acauccguaucuagacaaauc...............   1   0   S06
    .........................................................................acauccguaucuagacaaaucu..............   1   0   S05
```

Star **Mature**

cacccuuggucucuguugcauacuccuuccauccugaaaaguuugucuuauauuugucuagauacggauguaacauccguaucuagacaaaucuaagacaaaauuuuc

....acauccguaucuagacaaauAua.............	2	1	S02
....acauccguaucuagacaaaucuaa............	1	0	S05
....acauccguaucuagacaaaucuaa............	2	0	S02
....acauccguaucuagacaaaucuaa............	1	0	S04
....cauccguaucuagacaaau................	1	0	S05
....cauccguaucuagacaaaucua.............	1	0	S04
....cauccguaucuagacaaaucuaag...........	1	0	S05
....cauccguaucuagacaaaucuaag...........	1	0	S04
....auccguaucuagacaaaucuaag...........	2	0	S04
....auccguaucuagacaaaucuaag...........	1	0	S03
....auccguaucuagacaaaucuaag...........	4	0	S06
....auccguaucuagacaaaucuaag...........	1	0	S01
....auccguaucuagacaaaucuaaga..........	15	0	S04
....auccguaucuagacaaaucuaaga..........	1	0	S01
....auccguaucuagacaaaucuaaga..........	4	0	S03
....auccguaucuagacaaaucuaaga..........	17	0	S05
....auccguaucuagacaaaucuaaga..........	7	0	S06
....auccguaucuagacaaaucuaaga..........	4	0	S02
....uccguaucuagacaaauc...............	1	0	S05
....uccguaucuagacaaaucuaa............	1	0	S02
....uccguaucuagacaaaucuaaga..........	1	0	S04
....uccguaucuagacaaaucuaaga..........	1	0	S01
....uccguaucuagacaaaucuaagac.........	2	0	S05
....uccguaucuagacaaaucuaagac.........	2	0	S02
....uccguaucuagacaaaucuaagac.........	2	0	S04
....uccguaucuagacaaaucuaagac.........	1	0	S06
....uccguaucuagacaaaucuaagac.........	4	0	S03
....ccguaucuagacaaaucuaagac.........	1	0	S01
....ccguaucuagacaaaucuaagaca........	1	0	S02
....ccguaucuagacaaaucuaagaca........	1	0	S01
....cguaucuagacaaaucuaaga..........	1	0	S05
....cguaucuagacaaaucuaagac.........	1	0	S06
....cguaucuagacaaaucuaagaca........	3	0	S06
....uaucuagacaaaucuaagac.........	1	0	S05
....uaucuagacaaaucuaagac.........	1	0	S03
....uaucuagacaaaucuaagac.........	2	0	S02
....uaucuagacaaaucuaagaca........	1	0	S05
....uaucuagacaaaucuaagaca........	1	0	S04
....uaucuagacaaaucuaagaca........	1	0	S02
....aucuagacaaaucuaagaca........	1	0	S04
....aucuagacaaaucuaagaca........	2	0	S06
....ucuagacaaaucuaagac.........	1	0	S04
....ucuagacaaaucuaagaca........	1	0	S01
....ucuagacaaaucuaagaca........	1	0	S05
....ucuagacaaaucuaagacC........	1	1	S06
....ucuagacaaaucuaagacaaa......	1	0	S01
....agacaaaucuaagacaaaauuuu.	1	0	S06

Provisional ID	:	ta_iwgsc_2dl_v1_9842889_1927548
Score total	:	57.7
Score for star read(s)	:	3.9
Score for read counts	:	51.6
Score for mfe	:	0.5
Score for randfold	:	1.6
Score for cons. seed	:	
Total read count	:	113
Mature read count	:	159
Loop read count	:	0
Star read count	:	32

novel-m0292-5p

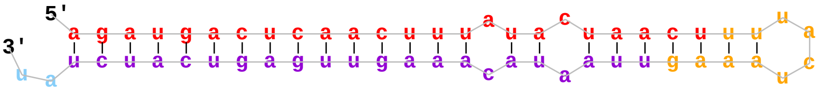

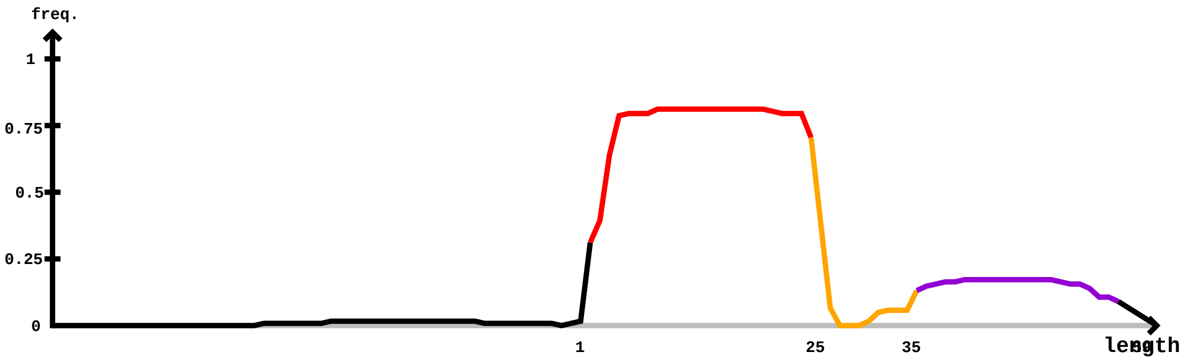

Mature Star

```
5'- cccaaagagaucaaccaccuuuuuauaacuacuagcuuacaugugcagccacaaagaugacucaacuuuauacuaacuuuuacuaaaguuaaauacaaaguugagucaucuauu  -3'   obs
    cccaaagagaucaaccaccuuuuuauaacuacuagcuuacaugugcagccacaaagaugacucaacuuuauacuaacuuuuacuaaaguuaaauacaaaguugagucaucuauu         exp
    ...((((((.........)))))).................((((....)))).(((((((((((((((.((.(((((((....))))))).)).)))))))))))))))...    reads   mm   sample
    ....................uuuuauaacuacuagcuuacaug.......................................................................    1       0    S02
    ...........................acuacuagcuuacaugugcagcca...............................................................    1       0    S02
    ....................................................aaagaugacucaacuuuauacuaacu....................................    1       0    S05
    .....................................................aagaugacucaacuuuauacu........................................    1       0    S06
    ......................................................agaugacucaacuuuauac.........................................    1       0    S03
    ......................................................agaugacucaacuuuauacuaac.....................................    1       0    S01
    ......................................................agaugacucaacuuuauacuaac.....................................    3       0    S05
    ......................................................agaugacucaacuuuauacuaac.....................................    1       0    S03
    ......................................................agaugacucaacuuuauacuaacu....................................    7       0    S06
    ......................................................agaugacucaacuuuauacuaacu....................................    4       0    S01
    ......................................................agaugacucaacuuuauacuaacu....................................    5       0    S05
    ......................................................agaugacucaacuuuauacuaacu....................................    1       0    S03
    ......................................................agaugacucaacuuuauacuaacu....................................    4       0    S02
    ......................................................agaugacucaacuuuauacuaacC....................................    1       1    S04
    ......................................................agaugacucaacuuuauacuaacu....................................    8       0    S04
    .......................................................gaugacucaacuuuauacuaac.....................................    1       0    S01
    .......................................................gaugacucaacuuuauacuaacu....................................    2       0    S03
    .......................................................gaugacucaacuuuauacuaacu....................................    1       0    S02
    .......................................................gaugacucaacuuuauacuaacu....................................    2       0    S05
    .......................................................gaugacucaacuuuauacuaacu....................................    2       0    S06
    .......................................................gaugacucaacuuuauacuaacuu...................................    1       0    S05
    .......................................................gaugacucaacuuuauacuaacuu...................................    1       0    S02
    ........................................................augacucaacuuuauacuaac.....................................    2       0    S01
    ........................................................augacucaacuuuauacuaac.....................................    1       0    S02
    ........................................................augacucaacuuuauacuaac.....................................    1       0    S06
    ........................................................augacucaacuuuauacuaacu....................................    1       0    S04
    ........................................................augacucaacuuuauacuaacu....................................    3       0    S02
    ........................................................augacucaacuuuauacuaacu....................................    1       0    S05
    ........................................................augacucaacuuuauacuaacuu...................................    3       0    S06
    ........................................................augacucaacuuuauacuaacuu...................................    4       0    S04
    ........................................................augacucaacuuuauacuaacuu...................................    1       0    S02
    ........................................................augacucaacuuuauacuaacuu...................................    4       0    S01
    ........................................................augacucaacuuuauacuaacuu...................................    3       0    S05
```

```
                                                     Mature                           Star
cccaaagagaucaaccaccuuuuuauaacuacuagcuuacaugugcagccacaaagaugacucaacuuuauacuaacuuuuacuaaaguuaauacaaaguugagucaucuauu
........................................................augacucaacuuuauacuGacuuu.................................  2  1  S04
........................................................augacucaacuuuauacuaacuuu.................................  1  0  S05
........................................................augacucaacuuuauacuaacuuu.................................  1  0  S02
........................................................augacucaacuuuauacuaacuuu.................................  1  0  S01
........................................................augacucaacuuuauacuaacuuu.................................  1  0  S04
.........................................................ugacucaacuuuauacuaac....................................  1  0  S04
.........................................................ugacucaacuuuauacuaacu...................................  3  0  S02
.........................................................ugacucaacuuuauacuaacu...................................  2  0  S03
.........................................................ugacucaacuuuauacuaacu...................................  2  0  S01
.........................................................ugacucaacuuuauacuaacu...................................  4  0  S06
.........................................................ugacucaacuuuauacuaacu...................................  3  0  S05
.........................................................ugacucaacuuuauacuaacuu..................................  1  0  S03
.........................................................ugacucaacuuuauacuaacuu..................................  1  0  S02
.........................................................ugacucaacuuuauacuaacuu..................................  1  0  S01
..........................................................gacucaacuuuauacuaacu...................................  1  0  S01
.............................................................ucaacuuuauacuaacuuu.................................  1  0  S04
.............................................................ucaacuuuauacuaacuuu.................................  1  0  S01
...................................................................................uaaaguuaauacaaaguuga..........  1  0  S01
...................................................................................uaaaguuaauacaaaguugag.........  1  0  S04
....................................................................................Uaaguuaauacaaaguugaguc.......  1  1  S04
....................................................................................aaaguuaauacaaaguugaguca......  1  0  S06
....................................................................................aaaguuaauacaaaguugaguca......  1  0  S05
....................................................................................aaaguuaauacaaaguugagucG......  1  1  S04
.....................................................................................aaguuaauacaaaguugagucauc....  1  0  S05
........................................................................................uuaauacaaaguugaguc.......  1  0  S06
........................................................................................uuaauacaaaguugagucC......  1  1  S05
........................................................................................uuaauacaaaguugagucaucu...  2  0  S05
........................................................................................uuaauacaaaguugagucaucu...  1  0  S01
........................................................................................uuaauacaaaguugagucaucu...  2  0  S04
........................................................................................uuaauacaaaguugagucaucuau.  2  0  S04
.........................................................................................uaauacaaaguugagucaucuau.  1  0  S04
.........................................................................................uaauacaaaguugagucaucuauu  1  0  S04
..........................................................................................aauacaaaguugagucauc....  1  0  S05
...........................................................................................auacaaaguugagucaucuauu  1  0  S02
.............................................................................................acaaaguugagucaucua..  1  0  S03
```

Provisional ID	:	ta_iwgsc_2dl_v1_9855501_1948578
Score total	:	2.2
Score for star read(s)	:	-1.3
Score for read counts	:	0
Score for mfe	:	1.9
Score for randfold	:	1.6
Score for cons. seed	:	
Total read count	:	77
Mature read count	:	90
Loop read count	:	0
Star read count	:	53

novel-m0757-5p

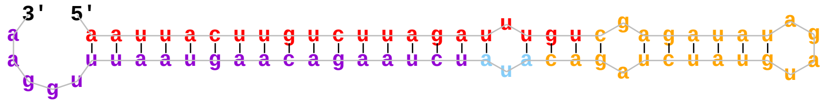

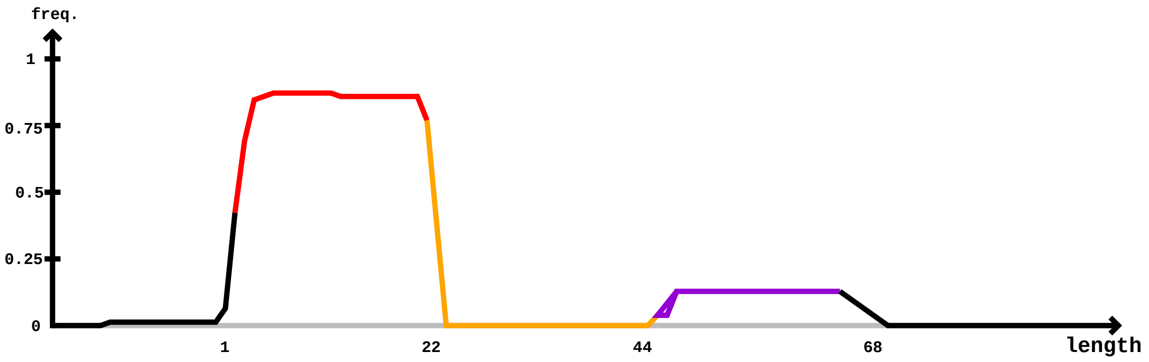

Mature **Star**

```
5'- auauuaucuccgucucaaauuacuugucuuagauuugucgagauauagauguaucuagacauaucuaagacaaguaauuuggaaccgaaggaguacuuuuuuagaugaa  -3'   obs
    auauuaucuccgucucaaauuacuugucuuagauuugucgagauauagauguaucuagacauaucuaagacaaguaauuuggaaccgaaggaguacuuuuuuagaugaa         exp
    .......((((.((((((((((((((((((((((.((((.((((((....)))))).)))).))))))))))))))))))))....)).))))................  reads  mm  sample
    ....uaucuccgucucaaauuacuuguc.................................................................................  1  0  S01
    ................aaauuacuugucuuagauuug........................................................................  1  0  S04
    ................aaauuacuugucuuagauuug........................................................................  2  0  S05
    ................aaauuacuugucuuagauuuguc......................................................................  1  0  S04
    .................aauuacuugucuuagauuug........................................................................  3  0  S03
    .................aauuacuugucuuagauuugu.......................................................................  7  0  S05
    .................aauuacuugucuuagauuugu.......................................................................  1  0  S01
    .................aauuacuugucuuagauuugu.......................................................................  5  0  S02
    .................aauuacuugucuuagauuugu.......................................................................  1  0  S06
    .................aauuacuugucuuagauuugu.......................................................................  7  0  S04
    .................aauuacuugucuuagauuugu.......................................................................  2  0  S03
    .................aauuacuugucuuagauuuguc......................................................................  1  0  S01
    .................aauuacuugucuuagauuuguc......................................................................  1  0  S03
    ..................auuacuugucuuagauuug........................................................................  1  0  S05
    ..................auuacuugucuuagauuugu.......................................................................  1  0  S06
    ..................auuacuugucuuagauuuguc......................................................................  3  0  S06
    ..................auuacuugucuuagauuuguc......................................................................  3  0  S04
    ..................auuacuugucuuagauuuguc......................................................................  4  0  S02
    ..................auuacuugucuuagauuuguc......................................................................  5  0  S01
    ..................auuacuugucuuagauuuguc......................................................................  2  0  S05
    ..................auuacuugucuuagauuuguc......................................................................  2  0  S03
    ...................uuacuugucuuagauuugu.......................................................................  1  0  S04
    ...................uuacuugucuuagauuugu.......................................................................  1  0  S05
    ...................uuacuugucuuagauuuguc......................................................................  3  0  S05
    ...................uuacuugucuuagauuuguc......................................................................  2  0  S04
    ...................uuacuugucuuagauuuguc......................................................................  2  0  S01
    ...................uuacuugucuuagauuuguc......................................................................  1  0  S06
    ...................uuacuugucuuagauuuguc......................................................................  2  0  S02
    ....................uacuugucuuagauuuguc......................................................................  1  0  S06
    .....................acuugucuuagauuuguc......................................................................  1  0  S04
    .............................................................uaucuaagacaaguaauuCg............................  1  1  S02
    .............................................................uaucuaagacaaguaauuugg...........................  1  0  S04
    .............................................................uaucuaagacaaguaauuugga..........................  1  0  S05
```

Mature Star

```
auauuaucuccgucucaaauuacuugucuuagauuugucgagauauagauguaucuagacauaucuaagacaaguaauuuggaaccgaaggaguacuuuuuuagaugaa
...............................................................ucuaagacaaguaauuugga..........................    1    0    S04
...............................................................ucuaagacaaguaauuugga..........................    1    0    S02
...............................................................ucuaagacaaguaauuuggaa.........................    2    0    S05
...............................................................ucuaagacaaguaauuuggaa.........................    2    0    S01
...............................................................ucuaagacaaguaauuuggaa.........................    1    0    S04
```

Provisional ID	:	ta_iwgsc_2dl_v1_9907757_2040834
Score total	:	2.3
Score for star read(s)	:	-1.3
Score for read counts	:	0
Score for mfe	:	2
Score for randfold	:	1.6
Score for cons. seed	:	
Total read count	:	197
Mature read count	:	131
Loop read count	:	0
Star read count	:	131

novel-m0354-3p

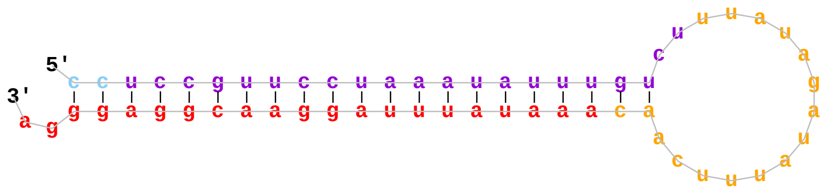

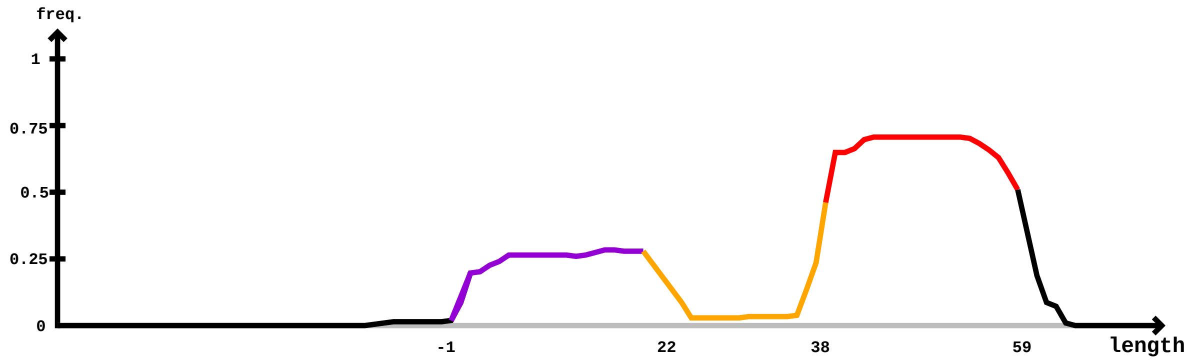

Star **Mature**

```
5'- uuaccauuuucuuuugagcgggaaggcaaaauauuacucccuccguuccuaaauauuugucuuuauagauauuucaacaaauauuuaggaacggagggaguaccaugaaugca  -3'   obs
    uuaccauuuucuuuugagcgggaaggcaaaauauuacucccuccguuccuaaauauuugucuuuauagauauuucaacaaauauuuaggaacggagggaguaccaugaaugca         exp
    ...((.(((......))).))....(((..((..(((((((((((((((((((((((((...............)))))))))))))))))))))))))..))...))).   reads   mm   sample
    ...............................uauuacucccuccguuccuUa.............................................................   1   1   S02
    ................................Uuuacucccuccguuccuaaauau.........................................................   1   1   S01
    .................................uuacucccuccguuccuaaauauu........................................................   1   0   S01
    .......................................ccuccguuccuaaauauuugu.....................................................   1   0   S05
    ........................................cuccguuccuaaauauuuguc....................................................   1   0   S01
    ........................................cuccguuccuaaauauuuguc....................................................   4   0   S06
    ........................................cuccguuccuaaauauuuguc....................................................   2   0   S05
    ........................................cuccguuccuaaauauuuguc....................................................   2   0   S04
    ........................................cuccguuccuaaauauuugCc....................................................   1   1   S05
    ........................................cuccguuccuaaauauuuguc....................................................   1   0   S03
    ........................................cuccguuccuaaauauuuguc....................................................   3   0   S02
    .........................................uccguuccuaaauauuuguc....................................................   1   0   S01
    .........................................uccguuccuaaauauuuguc....................................................   3   0   S05
    .........................................uccguuccuaaauauuugucu...................................................   2   0   S06
    .........................................uccguuccuaaauauuugucu...................................................   2   0   S04
    .........................................uccguuccuaaauauuugucu...................................................   3   0   S05
    .........................................uccguuccuaaauauuugucu...................................................   5   0   S01
    .........................................uccguuccuaaauauuugucu...................................................   4   0   S03
    .........................................uccguuccuaaauauuugucu...................................................   3   0   S02
    ..........................................ccguuccuaaauauuugucu...................................................   1   0   S01
    ...........................................cguuccuaaauauuugucu...................................................   1   0   S01
    ...........................................cguuccuaaauauuugucuuu.................................................   1   0   S02
    ...........................................cguuccuaaauauuugucuuu.................................................   1   0   S04
    ...........................................cguuccuaaauauuugucuuu.................................................   2   0   S01
    ............................................guuccuaaauauuugucuuu.................................................   1   0   S06
    ............................................guuccuaaauauuugucuuu.................................................   1   0   S05
    ............................................guuccuaaauauuugucuuu.................................................   1   0   S03
    .............................................uuccuaaauauuugucuuu.................................................   4   0   S01
    .............................................uuccuaaauauuugucuuu.................................................   1   0   S06
    .....................................................uauuugucuuuCuagauauuucaac...................................   1   1   S01
    ......................................................auuugucuuuCuagauauuucaac...................................   2   1   S06
    .......................................................uuugucuuuCuagauauuucaaca..................................   1   1   S04
    .......................................................uuugucuuuCuagauauuucaaca..................................   1   1   S03
```

Star **Mature**

uuaccauuuucuuuugagcgggaaggcaaaauauuacucccuccguuccuaaauauuugucuuuauagauauuucaacaaauauuuaggaacggagggaguaccaugaaugca			
....uugucuuuCuagauauuucaac...................................	1	1	S01
....auuucaacaaauauuuaggaacg....................	1	0	S05
....aacaaauauuuaggaacggagg................	1	0	S05
....acaaauauuuaggaacgg...................	1	0	S05
....acaaauauuuaggaacgg...................	2	0	S06
....acaaauauuuaggaacgg...................	1	0	S03
....acaaauauuuaggaacgga..................	1	0	S05
....acaaauauuuaggaacgga..................	1	0	S03
....acaaauauuuaggaacgga..................	1	0	S04
....acaaauauuuaggaacggag.................	1	0	S03
....acaaauauuuaggaacggag.................	1	0	S01
....acaaauauuuaggaacggagg................	3	0	S02
....acaaauauuuaggaacggagg................	2	0	S06
....acaaauauuuaggaacggagg................	2	0	S04
....acaaauauuuaggaacggagg................	1	0	S05
....acaaauauuuaggaacggaggg...............	1	0	S02
....acaaauauuuaggaacggagggag.............	2	0	S04
....caaauauuuaggaacgga..................	1	0	S03
....caaauauuuaggaacgga..................	1	0	S05
....caaauauuuaggaacggagg................	1	0	S06
....caaauauuuaggaacggaggg...............	2	0	S04
....caaauauuuaggaacggagggag.............	1	0	S03
....caaauauuuaggaacggagggagu............	4	0	S05
....caaauauuuaggaacggagggagu............	1	0	S04
....caaauauuuaggaacggagggagu............	4	0	S02
....caaauauuuaggaacggagggagu............	1	0	S03
....caaauauuuaggaacggagggagu............	3	0	S06
....caaauauuuaggaacggagggagu............	2	0	S01
....aaauauuuaggaacggag.................	3	0	S06
....aaauauuuaggaacggag.................	1	0	S01
....aaauauuuaggaacggagg................	1	0	S01
....aaauauuuaggaacggaggg...............	1	0	S03
....aaauauuuaggaacggaggg...............	5	0	S06
....aaauauuuaggaacggaggg...............	2	0	S02
....aaauauuuaggaacggaggga..............	10	0	S06
....aaauauuuaggaacggaggga..............	5	0	S04
....aaauauuuaggaacggaggga..............	9	0	S05
....aaauauuuaggaacggaggga..............	2	0	S01
....aaauauuuaggaacggaggga..............	1	0	S03
....aaauauuuaggaacggaggga..............	2	0	S02
....aaauauuuaggaacggagggag.............	1	0	S06
....aaauauuuaggaacggagggagu............	1	0	S01
....aaauauuuaggaacggagggagu............	2	0	S02
....aaauauuuaggaacggagggagu............	2	0	S04
....aaauauuuaggaacggagggagua...........	1	0	S04
....aaauauuuaggaacgAagggaguac..........	1	1	S06
....aaauauuuaggaacgAagggaguac..........	1	1	S04
....aauauuuaggaacggagg................	1	0	S01
....aauauuuaggaacggaggg...............	1	0	S03
....aauauuuaggaacggaggg...............	1	0	S05
....aauauuuaggaacggaggga..............	3	0	S06
....aauauuuaggaacggaggga..............	2	0	S04
....aauauuuaggaacggaggga..............	1	0	S03
....aauauuuaggaacggaggga..............	1	0	S02
....aauauuuaggaacggaggga..............	3	0	S05
....aauauuuaggaacggagggag.............	5	0	S04
....aauauuuaggaacggagggag.............	8	0	S05
....aauauuuaggaacggagggag.............	2	0	S01
....aauauuuaggaacggagggag.............	6	0	S02
....aauauuuaggaacggagggag.............	1	0	S03
....aauauuuaggaacggagggag.............	2	0	S06
....aauauuuaggaacggagggagu............	1	0	S05
....aauauuuaggaacggagggagua...........	1	0	S04
....aauauuuaggaacggagggagua...........	1	0	S03
....aauauuuaggaacggagggaguac..........	1	0	S03
....uauuuaggaacggagggaguac..........	1	0	S05
....uauuuaggaacggagggaguac..........	1	0	S03
....uUuuuaggaacggagggaguac..........	1	1	S04
....auuuaggaacggagggaguac..........	2	0	S05
....auuuaggaacggagggaguac..........	1	0	S01

Star Mature

```
uuaccauuuucuuuugagcgggaaggcaaaauauuacucccuccguuccuaaauauuugucuuuauagauauuucaacaaauauuuaggaacggagggaguaccaugaaugca
..................................................................................auuuaggaacggagggaguac..........     1     0     S02
..................................................................................auuuaggaacggagggaguac..........     1     0     S06
..................................................................................auuuaggaacggagggaguac..........     2     0     S04
...................................................................................uuuaggaacggagggaguacc.........     2     0     S04
```

Provisional ID	:	ta_iwgsc_2dl_v1_9909850_2061427
Score total	:	2.4
Score for star read(s)	:	-1.3
Score for read counts	:	0
Score for mfe	:	2.1
Score for randfold	:	1.6
Score for cons. seed	:	
Total read count	:	199
Mature read count	:	127
Loop read count	:	0
Star read count	:	121

novel-m0100-5p

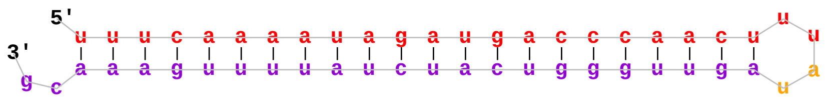

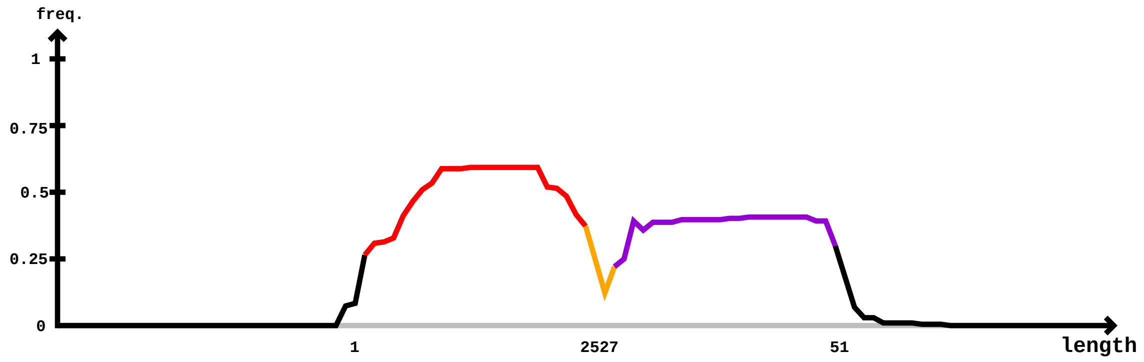

Mature Star

```
5'- acaacaucuacgccaccacggaucccuacguuucaaaauagaugacccaacuuuauaguugggucaucuauuuugaaacggagguaauacuugcuaggcgguucugau   -3'   obs
    acaacaucuacgccaccacggaucccuacguuucaaaauagaugacccaacuuuauaguugggucaucuauuuugaaacggagguaauacuugcuaggcgguucugau         exp
    ....((....((((..((.(....(((.((((((((((((((((((((((((....)))))))))))))))))))))))).))).....).))...))))....))..   reads   mm   sample
    ............................cguuucaaaauagaugaccca...........................................................   2   0   S06
    ............................cguuucaaaauagaugaccca...........................................................   2   0   S02
    ............................cguuucaaaauagaugaccca...........................................................   5   0   S04
    ............................cguuucaaaauagaugaccca...........................................................   2   0   S01
    ............................cguuucaaaauagaugaccca...........................................................   2   0   S05
    ............................cguuucaaaauagaugacccaacu........................................................   2   0   S06
    .............................guuucaaaauagaugacccaacu........................................................   1   0   S04
    .............................guuucaaaauagaugacccaacuu.......................................................   1   0   S06
    ..............................uuucaaaauagaugaccca...........................................................   1   0   S06
    ..............................uuucaaaauagaugacccaa..........................................................   1   0   S03
    ..............................uuucaaaauagaugacccaCc.........................................................   1   1   S03
    ..............................uuucaaaauagaugacccaac.........................................................   1   0   S06
    ..............................uuucaaaauagaugacccaac.........................................................   1   0   S05
    ..............................uuucaaaauagaugacccaac.........................................................   2   0   S04
    ..............................uuucaaaauagaugacccaacu........................................................   1   0   S05
    ..............................uuucaaaauagaugacccaacu........................................................   1   0   S02
    ..............................uuucaaaauagaugacccaacu........................................................   2   0   S04
    ..............................uuucaaaauagaugacccaacuu.......................................................   1   0   S06
    ..............................uuucaaaauagaugacccaacuu.......................................................   1   0   S05
    ..............................uuucaaaauagaugacccaacuu.......................................................   1   0   S04
    ..............................uuucaaaauagaugacccaacuuu......................................................   2   0   S06
    ..............................uuucaaaauagaugacccaacuuu......................................................   2   0   S03
    ..............................uuucaaaauagaugacccaacuuu......................................................   5   0   S02
    ..............................uuucaaaauagaugacccaacuuu......................................................   2   0   S01
    ..............................uuucaaaauagaugacccaacuuu......................................................   3   0   S05
    ..............................uuucaaaauagaugacccaacuuu......................................................   8   0   S04
    ..............................uuucaaaauagaugacccaacuAu......................................................   1   1   S04
    ...............................uucaaaauagaugaccca...........................................................   1   0   S04
    ...............................uucaaaauagaugacccaac.........................................................   1   0   S01
    ...............................uucaaaauagaugacccaacu........................................................   2   0   S01
    ...............................uucaaaauagaugacccaacu........................................................   1   0   S02
    ...............................uucaaaauagaugacccaacu........................................................   2   0   S05
    ...............................uucaaaauagaugacccaacuuu......................................................   1   0   S04
```

Mature Star

```
acaacaucuacgccaccacggaucccuacguuucaaaauagaugacccaacuuuauaguugggucaucuauuuugaaacggagguaauacuugcuaggcgguucugau
...............................uucaaaauagaugacccaacuuu......................................................  1  0  S05
................................ucaaaauagaugacccaacuuuau....................................................  1  0  S05
.................................caaaauagaugacccaacuuu......................................................  1  0  S05
.................................caaaauagaugacccaacuuu......................................................  1  0  S01
.................................caaaauagaugacccaacuuu......................................................  1  0  S06
..................................aaaauagaugacccaacu........................................................  1  0  S01
..................................aaaauagaugacccaacu........................................................  1  0  S06
..................................aaaauagaugacccaacuuu......................................................  3  0  S05
..................................aaaauagaugacccaacuuu......................................................  2  0  S01
..................................aaaauagaugacccaacuuu......................................................  3  0  S03
..................................aaaauagaugacccaacuuu......................................................  5  0  S06
..................................aaaauagaugacccaacuuuauagu.................................................  1  0  S04
..................................aaaauagaugacccaacuuuauagu.................................................  1  0  S06
...................................aaauagaugacccaacu........................................................  1  0  S02
...................................aaauagaugacccaacu........................................................  1  0  S04
...................................aaauagaugacccaacu........................................................  1  0  S05
...................................aaauagaugacccaacu........................................................  2  0  S06
...................................aaauagaugacccaacuuu......................................................  2  0  S03
...................................aaauagaugacccaacuuu......................................................  1  0  S02
...................................aaauagaugacccaacuuu......................................................  1  0  S04
...................................aaauagaugacccaacuuuauagu.................................................  2  0  S06
....................................aauagaugacccaacuuu......................................................  1  0  S01
....................................aauagaugacccaacuuu......................................................  1  0  S06
....................................aauagaugacccaacuuu......................................................  1  0  S02
....................................aauagaugacccaacuuu......................................................  2  0  S05
....................................aauagaugacccaacuuuaua...................................................  1  0  S02
....................................aauagaugacccaacuuuaua...................................................  1  0  S06
....................................aauagaugacccaacuuuauagu.................................................  2  0  S01
.....................................auagaugacccaacuuua.....................................................  1  0  S04
.....................................auagaugacccaacuuuaua...................................................  2  0  S05
.....................................auagaugacccaacuuuauagu.................................................  2  0  S05
......................................uagaugacccaacuuuauC...................................................  2  1  S05
......................................uagaugacccaacuuuauC...................................................  1  1  S01
......................................uagaugacccaacuuuauC...................................................  2  1  S02
......................................uagaugacccaacuuuaua...................................................  1  0  S04
......................................uagaugacccaacuuuauaA..................................................  1  1  S04
......................................uagaugacccaacuuuauagu.................................................  1  0  S02
......................................uagaugacccaacuuuaAagu.................................................  1  1  S05
......................................uagaugacccaacuuuauaUuu................................................  1  1  S06
......................................uagaugacccaacuuuauaUuu................................................  1  1  S05
.........................................augacccaacuuuauaUuu................................................  1  1  S03
........................................................aguugggucaucuauuuugaa...............................  1  0  S05
........................................................aguugggucaucuauuuugaaacg............................  4  0  S04
........................................................aguugggucaucuauuuugaaacg............................  2  0  S02
........................................................aguugggucaucuauuuugaaacg............................  4  0  S06
........................................................aguugggucaucuauuuugaaacg............................  2  0  S01
........................................................aguugggucaucuauuuugaaacg............................  6  0  S05
........................................................aCuugggucaucuauuuugaaacg............................  1  1  S06
........................................................aguugggucauAuauuuugaaacg............................  1  1  S06
.........................................................guugggucaucuauuuugaaacg............................  2  0  S06
.........................................................guugggucaucuauuuugaaacg............................  2  0  S05
.........................................................guugggucaucuauuuugaaacgg...........................  1  0  S03
.........................................................guugggucaucuauuuugaaacgg...........................  2  0  S01
.........................................................guugggucaucuauuuugaaacgg...........................  5  0  S05
.........................................................guugggucaucuauuuugaaacgg...........................  2  0  S06
.........................................................guugggucaucuauuuugaaacgg...........................  2  0  S04
..........................................................uugggucaucuauuuugaa...............................  1  0  S05
..........................................................uugggucaucuauuuugaaac.............................  1  0  S03
..........................................................uugggucaucuauuuugaaac.............................  1  0  S01
..........................................................uugggucaucuauuuugaaac.............................  7  0  S06
..........................................................uugggucaucuauuuugaaac.............................  3  0  S04
..........................................................uugggucaucuauuuugaaac.............................  3  0  S05
..........................................................uugggucaucuauuuugaaac.............................  3  0  S02
..........................................................uugggucaucuauuuugaaacg............................  1  0  S02
..........................................................uugggucaucuauuuugaaacg............................  2  0  S01
..........................................................uugggucaucuauuuugaaacgga..........................  4  0  S04
..........................................................uugggucaucuauuuugaaacgga..........................  1  0  S02
..........................................................uugggucaucuauuuugaaacgga..........................  3  0  S05
...........................................................ugggucaucuauuuugaa...............................  1  0  S03
...........................................................ugggucaucuauuuugaaac.............................  1  0  S05
```

Mature **Star**

```
acaacaucuacgccaccacggaucccuacguuucaaaauagaugacccaacuuuauaguugggucaucuauuuugaaacggagguaauacuugcuaggcgguucugau
...........................................................ugggucaucuauuuugaaacgg...........................	1	0	S06
............................................................gggucaucuauuuugaaacg............................	1	0	S01
............................................................gggucaucuauuuugaaacgg...........................	1	0	S04
............................................................gggucaucuauuuugaaacgg...........................	1	0	S03
............................................................gggucaucuauuuugaaacgg...........................	1	0	S02
............................................................gggucaucuauuuugaaacgg...........................	2	0	S01
............................................................gggucaucuauuuugaaacggagg........................	2	0	S05
............................................................gggucaucuauuuugaaacggagg........................	1	0	S06
...............................................................ucaucuauuuugaaacgg...........................	1	0	S03
...............................................................ucaucuauuuugaaacggagg........................	1	0	S05
....................................................................uauuuugaaacggaggGaauacu.................	1	1	S05
.......................................................................uuugaaacggagguaau....................	1	0	S01
```

Provisional ID	: ta_iwgsc_2ds_v1_5324744_2112339
Score total	: 200.7
Score for star read(s)	: 3.9
Score for read counts	: 192.8
Score for mfe	: 2.3
Score for randfold	: 1.6
Score for cons. seed	:
Total read count	: 390
Mature read count	: 309
Loop read count	: 0
Star read count	: 3

novel-m0148-5p

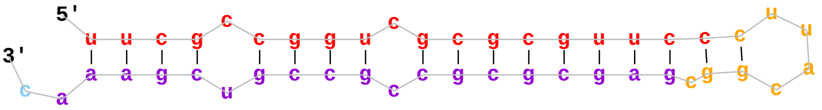

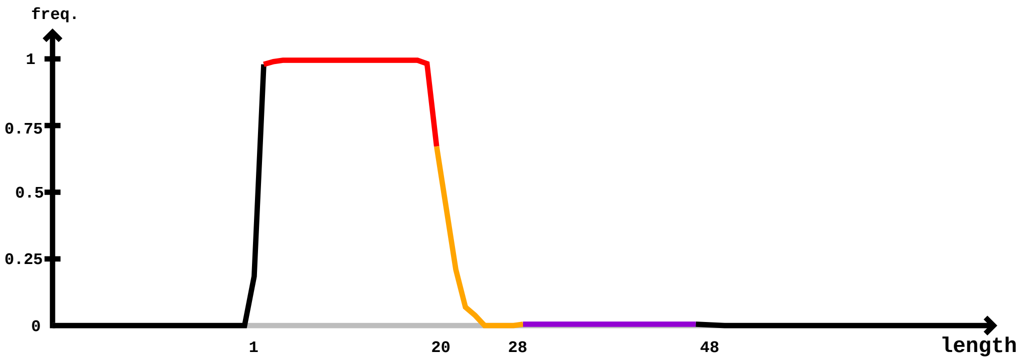

Mature **Star**

5'-	guggcgccguugccauuccuuucgccggucgcgcguucccuuacggcgagcgcgccgccgucgaaacguuuggccgcggugcccuuugccccucuc	-3' obs		
	guggcgccguugccauuccuuucgccggucgcgcguucccuuacggcgagcgcgccgccgucgaaacguuuggccgcggugcccuuugccccucuc	exp		
	..((((((((.((((..(.(((((.((((.((((((((((....)).)))))))).)))).))))).)..)))).))))))))).............	reads	mm	sample
	...................uuucgccggucgcgcguu...	3	0	S05
	...................uuucgccggucgcgcguu...	1	0	S06
	...................uuucgccggucgcgcguu...	1	0	S03
	...................uuucgccggucgcgcguuc..	2	0	S03
	...................uuucgccggucgcgcguucc...	2	0	S01
	...................uuucgccggucgcgcguucc...	5	0	S03
	...................uuucgccggucgcgcguucc...	2	0	S05
	...................uuucgccggucgcgcguucc...	2	0	S06
	...................uuucgccggucgcgcguucc...	2	0	S04
	...................uuucgccggucgcgcguuccc..	5	0	S02
	...................uuucgccggucgcgcguuccc..	2	0	S03
	...................uuucgccggucgcgcguuccc..	1	0	S05
	...................uuucgccggucgcgcguucccu...	8	0	S06
	...................uuucgccggucgcgcguucccu...	1	0	S01
	...................uuucgccggucgcgcguucccu...	2	0	S02
	...................uuucgccggucgcgcguucccu...	3	0	S03
	...................uuucgccggucgcgcguucccu...	2	0	S04
	...................uuucgccggucgcgcguucccu...	6	0	S05
	...................uuucgccggucgcgcguucccuu..	1	0	S02
	...................uuucgccggucgcgcguucccuu..	6	0	S05
	...................uuucgccggucgcgcguucccuua...	2	0	S04
	...................uuucgccggucgcgcguucccuua...	2	0	S06
	...................uuucgccggucgcgcguucccuua...	6	0	S05
	...................uuucgccggucgcgcguucccuua...	1	0	S01
	...................uuucgccggucgcgcguucccuua...	1	0	S02
	...................uuucgccggucgcgcguucccuua...	3	0	S03
	....................uucgccggucgcgcguuc..	20	0	S03
	....................uucgccggucgcgcguuc..	9	0	S02
	....................uucgccggucgcgcguuc..	39	0	S05
	....................uucgccggucgcgcguuc..	21	0	S04
	....................uucgccggucgcgcguuc..	20	0	S06
	....................uucgccggucgcgcguuc..	10	0	S01
	....................uucgccggucgcgcguucc...	3	0	S04

```
                    Mature                    Star
guggcgccguugccauuccuuucgccggucgcgcguucccuuacggcgagcgcgccgccgucgaaacguuuggccgcggugcccuuugccccucuc
....................uucgccggucgcgcguucc.........................................................   43   0   S05
....................uucgccggucgcgcguucc.........................................................   9    0   S02
....................uucgccggucgcgcguucc.........................................................   5    0   S01
....................uucgccggucgcgcguucc.........................................................   27   0   S06
....................uucgccggucgcgcguucc.........................................................   5    0   S03
....................uucgccggucgcgcguuccc........................................................   7    0   S02
....................uucgccggucgcgcguuccc........................................................   7    0   S03
....................uucgccggucgcgcguuccc........................................................   4    0   S01
....................uucgccggucgcgcguuccc........................................................   24   0   S05
....................uucgccggucgcgcguuccc........................................................   13   0   S06
....................uucgccggucgcgcguuccc........................................................   10   0   S04
....................uucgccggucgcgcguucccu.......................................................   9    0   S05
....................uucgccggucgcgcguucccu.......................................................   1    0   S01
....................uucgccggucgcgcguucccu.......................................................   4    0   S04
....................uucgccggucgcgcguucccu.......................................................   13   0   S06
....................uucgccggucgcgcguucccu.......................................................   4    0   S02
....................uucgccggucgcgcguucccuu......................................................   1    0   S01
....................uucgccggucgcgcguucccuu......................................................   1    0   S04
....................uucgccggucgcgcguucccuu......................................................   1    0   S05
.....................ucgccggucgcgcguuccc........................................................   1    0   S01
.....................ucgccggucgcgcguucccu.......................................................   1    0   S06
.....................ucgccggucgcgcguucccuu......................................................   1    0   S02
.....................ucgccggucgcgcguucccuu......................................................   1    0   S05
......................cgccggucgcgcguuccc........................................................   1    0   S04
......................cgccggucgcgcguucccu.......................................................   1    0   S01
...............................................gagcgcgccgccgucgaaa..............................   2    0   S01
```

Provisional ID	:	ta_iwgsc_2ds_v1_5338269_2133933
Score total	:	155.5
Score for star read(s)	:	3.9
Score for read counts	:	148
Score for mfe	:	2.1
Score for randfold	:	1.6
Score for cons. seed	:	
Total read count	:	302
Mature read count	:	97
Loop read count	:	0
Star read count	:	338

novel-0238-3p

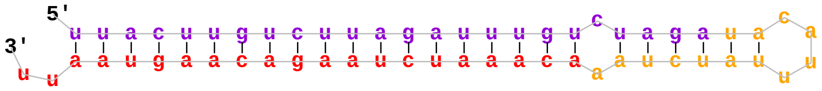

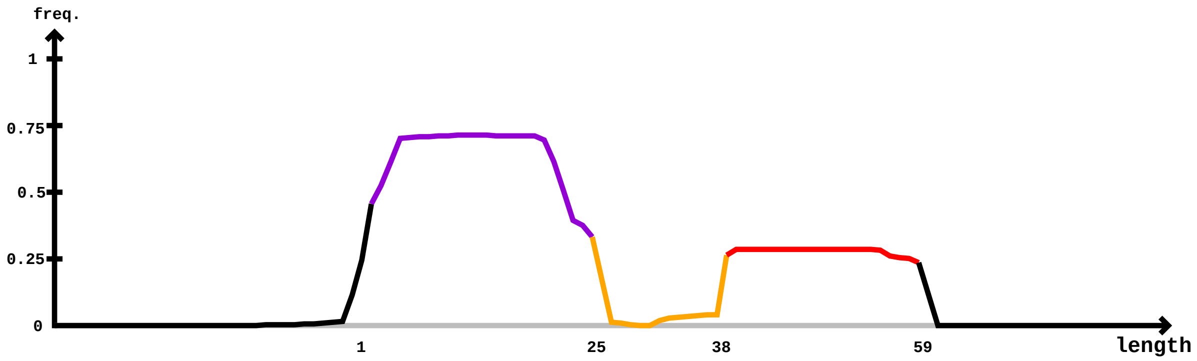

Star Mature

```
5'- auaauacgcuaguauaguccucuguccugaauuacuugucuuagauuugucuagauacauuuaucuaaacaaaucuaagacaaguaauuuaauuuuaggacgcagguaauauua   -3'   obs
    auaauacgcuaguauaguccucuguccugaauuacuugucuuagauuugucuagauacauuuaucuaaacaaaucuaagacaaguaauuuaauuuuaggacgcagguaauauua         exp
    ...((((....))))...(((.((((((((((((((((((((((((((((.((((((....)))))).))))))))))))))))))))......)))))))).)))........    reads   mm   sample
    ....................ucuguccugaauuacuugucuuag......................................................................    1       0    S05
    ........................uccugaauuacuugucuuagauuuguc...............................................................    1       0    S03
    ..........................cugaauuacuugucuuagauuug.................................................................    1       0    S04
    ...........................ugaauuacuugucuuagauuuguc...............................................................    1       0    S01
    ............................gaauuacuugucuuagauuugucu..............................................................    1       0    S06
    .............................aauuacuugucuuagauuug.................................................................    3       0    S03
    .............................aauuacuugucuuagauuugu................................................................    5       0    S02
    .............................aauuacuugucuuagauuugu................................................................    7       0    S05
    .............................aauuacuugucuuagauuugu................................................................    2       0    S03
    .............................aauuacuugucuuagauuugu................................................................    1       0    S01
    .............................aauuacuugucuuagauuugu................................................................    1       0    S06
    .............................aauuacuugucuuagauuugu................................................................    7       0    S04
    .............................aauuacuugucuuagauuuguc...............................................................    1       0    S03
    .............................aauuacuugucuuagauuuguc...............................................................    1       0    S01
    .............................aauuacuugucuuagauuugucu..............................................................    1       0    S01
    .............................aauuacuugucuuagauuugucu..............................................................    1       0    S04
    .............................aauuacuugucuuagauuugucua.............................................................    1       0    S04
    .............................aauuacuugucuuagauuugucua.............................................................    1       0    S02
    ..............................auuacuugucuuagauuug.................................................................    1       0    S05
    ..............................auuacuugucuuagauuugu................................................................    1       0    S06
    ..............................auuacuugucuuagauuuguc...............................................................    3       0    S04
    ..............................auuacuugucuuagauuuguc...............................................................    3       0    S06
    ..............................auuacuugucuuagauuuguc...............................................................    2       0    S03
    ..............................auuacuugucuuagauuuguc...............................................................    4       0    S02
    ..............................auuacuugucuuagauuuguc...............................................................    2       0    S05
    ..............................auuacuugucuuagauuuguc...............................................................    5       0    S01
    ..............................auuacuugucuuagauuugucu..............................................................    1       0    S05
    ..............................auuacuugucuuagauuugucuag............................................................    1       0    S03
    ..............................auuacuugucuuagauuugucuag............................................................    5       0    S04
    ..............................auuacuugucuuagauuugucuag............................................................    1       0    S06
    ..............................auuacuugucuuagauuugucuag............................................................    2       0    S01
    ..............................auuacuugucuuagauuugucuag............................................................    3       0    S05
    ..............................auuacuugucuuagauuugucuaga...........................................................    2       0    S04
```

Star **Mature**

```
auaauacgcuaguauaguccucuguccugaauuacuugucuuagauuugucuagauacauuuaucuaaacaaaucuaagacaaguaauuuaauuuuaggacgcagguaauauua
..............................auuacuugucuuagauuugucuaga...........................................................  1  0  S01
..............................auuacuugucuuagauuugucuaga...........................................................  1  0  S02
..............................auuacuugucuuagauuugucuaga...........................................................  1  0  S05
..............................auuacuugucuuagauuugucuaga...........................................................  3  0  S06
...............................uuacuugucuuagauuugu................................................................  1  0  S05
...............................uuacuugucuuagauuugu................................................................  1  0  S04
...............................uuacuugucuuagauuuguc...............................................................  2  0  S04
...............................uuacuugucuuagauuuguc...............................................................  2  0  S02
...............................uuacuugucuuagauuuguc...............................................................  2  0  S01
...............................uuacuugucuuagauuuguc...............................................................  1  0  S06
...............................uuacuugucuuagauuuguc...............................................................  3  0  S05
...............................uuacuugucuuagauuugucu..............................................................  6  0  S05
...............................uuacuugucuuagauuugucu..............................................................  4  0  S01
...............................uuacuugucuuagauuugucu..............................................................  3  0  S04
...............................uuacuugucuuagauuugucu..............................................................  2  0  S06
...............................uuacuugucuuagauuugucu..............................................................  4  0  S03
...............................uuacuugucuuagauuugucu..............................................................  2  0  S02
...............................uuacuugucuuagauuugucuU.............................................................  1  1  S06
...............................uuacuugucuuagauuugucuU.............................................................  1  1  S04
...............................uuacuugucuuagauuugucuaga...........................................................  3  0  S01
...............................uuacuugucuuagauuugucuaga...........................................................  3  0  S02
...............................uuacuugucuuagauuugucuaga...........................................................  2  0  S06
...............................uuacuugucuuUgauuugucuaga...........................................................  1  1  S03
...............................uuacuugucuuagauuugucuaga...........................................................  2  0  S03
...............................uuacuugucuuagauuugucuaga...........................................................  13  0  S04
...............................uuacuugucuuagauuugucuaga...........................................................  9  0  S05
................................uacuugucuuagauuuguc...............................................................  1  0  S06
................................uacuugucuuagauuugucu..............................................................  1  0  S03
................................uacuugucuuagauuugucu..............................................................  2  0  S04
................................uacuugucuuagauuugucu..............................................................  4  0  S01
................................uacuugucuuagauuugucu..............................................................  1  0  S02
................................uacuugucuuagauuugucua.............................................................  1  0  S01
................................uacuugucuuagauuugucua.............................................................  1  0  S05
................................uacuugucuuagauuugucuaga...........................................................  4  0  S04
................................uacuugucuuagauuugucuaga...........................................................  2  0  S06
................................uacuugucuuagauuugucuaga...........................................................  1  0  S01
................................uacuugucuuagauuugucuaga...........................................................  2  0  S05
................................uacuugucuuagauuugucuaga...........................................................  2  0  S02
.................................acuugucuuagauuuguc...............................................................  1  0  S04
.................................acuugucuuagauuugucu..............................................................  1  0  S04
.................................acuugucuuagauuugucu..............................................................  1  0  S05
.................................acuugucuuagauuugucuag............................................................  1  0  S04
.................................acuugucuuagauuugucuaga...........................................................  5  0  S01
.................................acuugucuuagauuugucuaga...........................................................  8  0  S05
.................................acuugucuuagauuugucuaga...........................................................  5  0  S06
.................................acuugucuuagauuugucuaga...........................................................  2  0  S04
.................................acuugucuuagauuugucuaga...........................................................  1  0  S02
.................................acuugucuuagauuugucuaga...........................................................  2  0  S03
.................................acuugucuuagauuugucuagaua.........................................................  1  0  S04
..................................cuugucuuagauuugucu..............................................................  1  0  S04
..................................cuugucuuagauuugucuag............................................................  1  0  S01
..................................cuugucuuagauuugucuaga...........................................................  7  0  S04
..................................cuugucuuagauuugucuaga...........................................................  2  0  S01
..................................cuugucuuagauuugucuaga...........................................................  6  0  S05
..................................cuugucuuagauuugucuaga...........................................................  4  0  S03
..................................cuugucuuagauuugucuaga...........................................................  4  0  S06
..................................cuugucuuagauuugucuaga...........................................................  4  0  S02
...................................uugucuuagauuugucuaga...........................................................  1  0  S03
....................................ugucuuagauuugucuagauac........................................................  1  0  S02
......................................ucuuagauuugucuagauaca.......................................................  1  0  S05
........................................uuagauuugucuagauac........................................................  1  0  S04
.............................................................uaucuaaacaaaucuaagacaag..............................  1  0  S03
.............................................................uaucuaaacaaaucuaagacaagA.............................  1  1  S06
.............................................................uaucuaaacaaaucuaagacaagA.............................  1  1  S04
.............................................................uaucuaaacaaaucuaagacaagA.............................  1  1  S01
.............................................................uaucuaaacaaaucuaagacaagu.............................  1  0  S01
.............................................................uaucuaaacaaaucuaagacaagA.............................  1  1  S03
..............................................................aucuaaacaaaucuaagacaagA.............................  1  1  S04
..............................................................aucuaaacaaaucuaagacaagua............................  1  0  S02
..............................................................aucuaaacaaaucuaagacaagua............................  1  0  S04
```

```
                              Star                                Mature
auaauacgcuaguauaguccucuguccugaauuacuugucuuagauuugucuagauacauuuaucuaaacaaaucuaagacaaguaauuuaauuuuaggacgcagguaauauua
...............................................................ucuaaacaaaucuaagacaagA.............................	1	1	S02
................................................................cuaaacaaaucuaagacaaguaauu.........................	1	0	S05
.................................................................uaaacaaaucuaagacaaguaauu.........................	1	0	S01
..................................................................aaaAaaaucuaagacaaguaauu.........................	1	1	S04
....................................................................acaaaucuaagacaaguaa...........................	1	0	S01
....................................................................acaaaucuaagacaaguaau..........................	1	0	S03
....................................................................acaaaucuaagacaaguaau..........................	2	0	S01
....................................................................acaaaucuaagacaaguaau..........................	1	0	S05
....................................................................acaaaucuaagacaaguaauu.........................	4	0	S06
....................................................................acaaaucuaagacaaguaauu.........................	17	0	S05
....................................................................acaaaucuaagacaaguaauu.........................	12	0	S04
....................................................................acaaaucuaagacaaguaauu.........................	21	0	S01
....................................................................acaaaucuaagacaaguaauu.........................	11	0	S02
....................................................................acaaaucuaagacaaguaauu.........................	2	0	S03
.....................................................................caaaucuaagacaaguaau..........................	1	0	S02
.....................................................................caaaucuaagacaaguaauu.........................	3	0	S01
.....................................................................caaaucuaagacaaguaauu.........................	1	0	S04
.....................................................................caaaucuaagacaaguaauuu........................	1	0	S01
.....................................................................caaaucuaagacaaguaauuu........................	1	0	S05
```

Provisional ID	:	ta_iwgsc_3as_v1_3357776_2501062
Score total	:	112.5
Score for star read(s)	:	3.9
Score for read counts	:	105.2
Score for mfe	:	1.8
Score for randfold	:	1.6
Score for cons. seed	:	
Total read count	:	218
Mature read count	:	484
Loop read count	:	0
Star read count	:	30

novel-m0861-5p

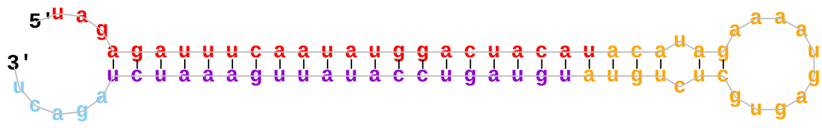

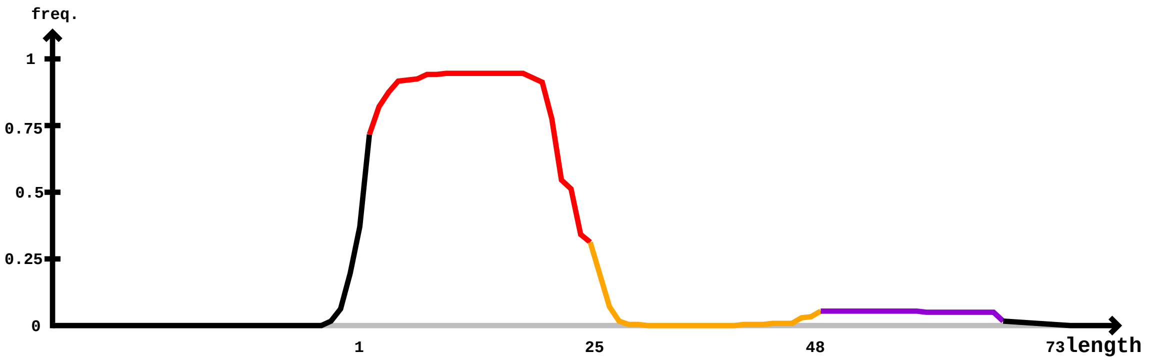

Mature   Star

```
5'- uccguuguacacauauauauauaugucuuuuuagagauuucaauauggacuacauacauagaaaaugagugcucuguauguaguccauauugaaaucuagacuuguauu   -3'   obs
    uccguuguacacauauauauauaugucuuuuuagagauuucaauauggacuacauacauagaaaaugagugcucuguauguaguccauauugaaaucuagacuuguauu         exp
    ..................(((((.((((......((((((((((((((((((((((((.((..........)).))))))))))))))))))))))))))))).))))).   reads   mm   sample
    ...........................uuuuuagagauuucaauaugg.............................................................   2   0   S04
    ...........................uuuuuagagauuucaauauggac...........................................................   1   0   S04
    ...........................uuuuuagagauuCcaauauggac...........................................................   1   1   S04
    ............................uuuuagagauuucaauaugg.............................................................   1   0   S02
    ............................uuuuagagauuucaauaugga............................................................   1   0   S04
    ............................uuuuagagauuCcaauauggac...........................................................   2   1   S04
    ............................uuuuagagauuucaauauggac...........................................................   1   0   S02
    ............................uuuuagagauuCcaauauggac...........................................................   2   1   S05
    ............................uuuuagagauuCcaauauggac...........................................................   1   1   S01
    ............................uuuuagagauuucaauauggacu..........................................................   1   0   S04
    ............................uuuuagagauuucaauauggacu..........................................................   1   0   S05
    ............................uuuuagagauuucaauauggacua.........................................................   1   0   S02
    .............................uuuagagauuucaauaugg.............................................................   1   0   S04
    .............................uuuagagauuucaauaugga............................................................   1   0   S02
    .............................uuuagagauuucaauauggac...........................................................   2   0   S06
    .............................uuuagagauuucaauauggac...........................................................   1   0   S05
    .............................uuuagagauuCcaauauggac...........................................................   1   1   S06
    .............................uuuagagauuCcaauauggac...........................................................   2   1   S02
    .............................uuuagagauuCcaauauggac...........................................................   1   1   S01
    .............................uuuagagauuCcaauauggac...........................................................   1   1   S04
    .............................uuuagagauuucaauauggacu..........................................................   2   0   S05
    .............................uuuagagauuCcaauauggacu..........................................................   1   1   S05
    .............................uuuagagauuucaauauggacu..........................................................   1   0   S02
    .............................uuuagagauuucaauauggacua.........................................................   1   0   S04
    .............................uuuagagauuucaauauggacua.........................................................   1   0   S05
    .............................uuuagagauuCcaauauggacuac........................................................   2   1   S01
    .............................uuuagagauuCcaauauggacuac........................................................   1   1   S05
    .............................uuuagagauuucaauauggacuac........................................................   1   0   S02
    .............................uuuagagauuCcaauauggacuac........................................................   2   1   S04
    .............................uuuagagauuucaauauggacuac........................................................   5   0   S05
    .............................uuuagagauuucaauauggacuac........................................................   5   0   S04
    ..............................uuagagauuucaauauggaU...........................................................   1   1   S04
    ..............................uuagagauuCcaauauggac...........................................................   2   1   S04
```

Mature **Star**

uccguuguacacauauauauauaugucuuuuuagagauuucaauauggacuacauacauagaaaaugagugcucuguauguaguccauauugaaaucuagacuuguauu			
..............................uuagagauuucaauauggac...	1	0	S02
..............................uuagagauuCcaauauggac...	1	1	S05
..............................uuagagauuCcaauauggac...	2	1	S01
..............................uuagagauuucaauauggac...	2	0	S05
..............................uuagagauuCcaauauggacu..	1	1	S06
..............................uuagagauuucaauauggacu..	5	0	S02
..............................uuagagauuucaauauggacu..	4	0	S05
..............................uuagagauuucaauauggacu..	10	0	S04
..............................uuagagauuCcaauauggacu..	2	1	S04
..............................uuagagauuCcaauauggacu..	2	1	S01
..............................uuagagauuCcaauauggacu..	1	1	S02
..............................uuagagauuucaauauggacu..	4	0	S01
..............................uuagagauuucaauauggacuac..	3	0	S04
..............................uuagagauuucaauauggacuaca...	1	0	S04
...............................uagagauuucaauaugga..	1	0	S04
...............................uagagauuucaauaugga..	1	0	S03
...............................uagagauuucaauauggaU...	1	1	S04
...............................uagagauuucaauauggaU...	1	1	S02
...............................uagagauuucaauauggaU...	3	1	S05
...............................uagagauuucaauauggac...	2	0	S01
...............................uagagauuucaauauggaU...	1	1	S01
...............................uagagauuucaauauggacu..	9	0	S05
...............................uagagauuucaauauggacC..	1	1	S04
...............................uagagauuucaauauggacu..	4	0	S04
...............................uagagauuucaauauggacu..	1	0	S02
...............................uagagauuucaauauggacu..	5	0	S01
...............................uagagauuucaauauggacua...	2	0	S04
...............................uagagauuucaauauggacua...	1	0	S05
...............................uagagauuucaauauggacuac..	1	0	S05
...............................uagagauuucaauauggacuaca...	1	0	S01
...............................uagagauuucaauauggacuaca...	1	0	S05
...............................uagagauuucaauauggacuaca...	1	0	S04
...............................uagagauuucaauauggacuacau..	1	0	S06
...............................uagagauuucaauauggacuaUau..	4	1	S04
...............................uagagauuucaauauggacuacau..	1	0	S02
...............................uagagauuucaauauggacuaUau..	2	1	S01
...............................uagagauuucaauauggacuacau..	18	0	S05
...............................uagagauuucaauauggacuaUau..	2	1	S05
...............................uagagauuucaauauggacuacau..	7	0	S01
...............................uagagauuucaauauggacuacau..	10	0	S04
...............................uagagauuCcaauauggacuacauac..	1	1	S04
................................agagauuucaauauggacua...	1	0	S04
................................agagauuucaauauggacua...	1	0	S03
................................agagauuucaauauggacuac..	4	0	S04
................................agagauuucaauauggacuac..	6	0	S06
................................agagauuucaauauggacuac..	2	0	S02
................................agagauuucaauauggacuac..	3	0	S01
................................agagauuucaauauggacuac..	5	0	S05
................................agagauuucaauauggacuacaua...	1	0	S05
................................agagauuucaauauggacuacaua...	1	0	S01
................................agagauuucaauauggacuacaua...	1	0	S04
.................................gagauuucaauauggacuac..	1	0	S04
.................................gagauuucaauauggacuaca...	1	0	S04
.................................gagauuucaauauggacuaUa...	1	1	S04
.................................gagauuucaauauggacuaUauac..	2	1	S05
.................................gagauuucaauauggacuacauac..	3	0	S04
.................................gagauuCcaauauggacuacauac..	3	1	S04
.................................gagauuucaauauggacuacauac..	2	0	S02
..................................agauuucaauauggacuaca...	1	0	S02
..................................agauuucaauauggacuacau..	2	0	S01
..................................agauuucaauauggacuacau..	1	0	S02
..................................agauuucaauauggacuaUau..	1	1	S05
..................................agauuucaauauggacuacau..	3	0	S06
..................................agauuucaauauggacuacau..	1	0	S03
..................................agauuucaauauggacuacau..	1	0	S04
...................................gauuucaauauggacuacaua...	1	0	S02
....................................auuCcaauauggacuacauac..	1	1	S04
.....................................uuCcaauauggacuacauac..	1	1	S02
.....................................uuucaauauggacuacauacU...	1	1	S02
.....................................uuucaauauggacuacauaca...	1	0	S02

Mature / Star			
uccguuguacacauauauauauaugucuuuuuagagauuucaauauggacuacauacauagaaaaugagugcucuguauguaguccauauugaaaucuagacuuguauu			
.....................................uuucaauauggacuacauacU...	1	1	S04
....ucaauauggacuacauacaua...	1	0	S05
....gcucuguauguaguccaua....................	1	0	S03
....cuguauguaguccauauugaaauc............	1	0	S06
....uauguaguccauauugaaauc............	1	0	S03
....uauguaguccauauugaaauc............	3	0	S01
....uauguaguccauauugaaauc............	1	0	S02
....auguaguccauauugaaaucu...........	1	0	S01
....uguaguccauauugaaauc............	1	0	S04
....uguaguccauauugaaauc............	1	0	S01
....uguaguccauauugaaaucu...........	1	0	S02
....uguaguccauauugaaaucu...........	1	0	S05
....uguaguccauauugaaaucu...........	1	0	S04

Provisional ID	:	ta_iwgsc_3as_v1_3420147_2532878
Score total	:	72.1
Score for star read(s)	:	3.9
Score for read counts	:	64.4
Score for mfe	:	2.2
Score for randfold	:	1.6
Score for cons. seed	:	
Total read count	:	138
Mature read count	:	176
Loop read count	:	0
Star read count	:	107

novel-0612-5p

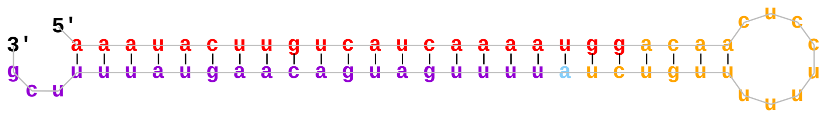

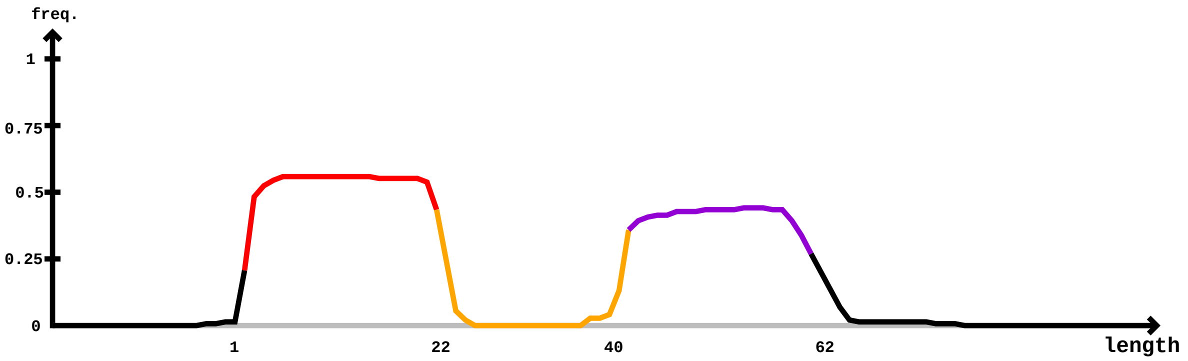

Mature **Star**

```
5'- cauacucccucccucuggaaauacuugucaucaaaauggacaacuccuuuuuugucuauuuugaugacaaguauuuucggacgaaaggaguauuucuuuuuuuuggaaaaacu -3'   obs
    cauacucccucccucuggaaauacuugucaucaaaauggacaacuccuuuuuugucuauuuugaugacaaguauuuucggacgaaaggaguauuucuuuuuuuuggaaaaacu        exp
    ..((((((...(.((((((((((((((((((((((((((((((........)))))))))))))))))))))))))))))).)...))))))(((((.......)))))....   reads   mm   sample
    ..............cuggaaauacuugucauc.................................................................................   1   0   S05
    ................ggaaauacuugucaucaaaaug...........................................................................   1   0   S03
    ..................aaauacuugucaucaaaaug...........................................................................   1   0   S01
    ..................aaauacuugucaucaaaaug...........................................................................   2   0   S04
    ..................aaauacuugucaucaaaaug...........................................................................   1   0   S06
    ..................aaauacuugucaucaaaaug...........................................................................   1   0   S03
    ..................aaauacuugucaucaaaaug...........................................................................   3   0   S02
    ..................aaauacuugucaucaaaaugg..........................................................................   1   0   S04
    ..................aaauacuugucaucaaaaugg..........................................................................   5   0   S03
    ..................aaauacuugucaucaaaaugg..........................................................................   4   0   S06
    ..................aaauacuugucaucaaaaugg..........................................................................   6   0   S01
    ..................aaauacuugucaucaaaaugg..........................................................................   2   0   S05
    ..................aaauacuugucaucaaaaugga.........................................................................   1   0   S02
    ..................aaauacuugucaucaaaaugga.........................................................................   1   0   S05
    ...................aauacuugucaucaaaau............................................................................   2   0   S04
    ...................aauacuugucaucaaaaug...........................................................................   1   0   S02
    ...................aauacuugucaucaaaaug...........................................................................   1   0   S06
    ...................aauacuugucaucaaaaug...........................................................................   1   0   S05
    ...................aauacuugucaucaaaaug...........................................................................   1   0   S01
    ...................aauacuugucaucaaaaug...........................................................................   1   0   S03
    ...................aauacuugucaucaaaaugg..........................................................................   2   0   S05
    ...................aauacuugucaucaaaaugg..........................................................................   1   0   S04
    ...................aauacuugucaucaaaaugg..........................................................................   3   0   S01
    ...................aauacuugucaucaaaaugg..........................................................................   6   0   S06
    ...................aauacuugucaucaaaaugg..........................................................................   3   0   S02
    ...................aauacuugucaucaaaaugg..........................................................................   3   0   S03
    ...................aauacuugucaucaaaaugga.........................................................................   2   0   S05
    ...................aauacuugucaucaaaaugga.........................................................................   2   0   S06
    ...................aauacuugucaucaaaaugga.........................................................................   2   0   S02
    ...................aauacuugucaucaaaaugga.........................................................................   2   0   S04
    ...................aauacuugucaucaaaaugga.........................................................................   3   0   S03
    ...................aauacuugucaucaaaaugga.........................................................................   1   0   S01
    ...................aauacuugucaucaaaauggac........................................................................   1   0   S05
```

Mature **Star**

```
cauacucccucccucuggaaauacuugucaucaaaauggacaacuccuuuuuugucuauuuugaugacaaguauuuucggacgaaaggaguauuucuuuuuuuuggaaaaacu
...................aauacuugucaucaaaauggaca.......................................................................   1   0   S02
...................aauacuugucaucaaaauggacU.......................................................................   1   1   S06
....................auacuugucaucaaaaug...........................................................................   1   0   S06
....................auacuugucaucaaaaugga.........................................................................   1   0   S04
....................auacuugucaucaaaaugga.........................................................................   1   0   S01
....................auacuugucaucaaaauggac........................................................................   1   0   S02
....................auacuugucaucaaaauggac........................................................................   2   0   S04
.....................uacuugucaucaaaaugg..........................................................................   1   0   S04
.....................uacuugucaucaaaaugga.........................................................................   1   0   S06
.....................uacuugucaucaaaauggacU.......................................................................   1   1   S02
......................acuugucaucaaaaugga.........................................................................   1   0   S03
......................acuugucaucaaaauggac........................................................................   1   0   S05
......................................................ucuauuuugaugacaagua........................................   1   0   S06
......................................................ucuauuuugaugacaaguauu......................................   1   0   S01
......................................................ucuauuuugaugacaaguauu......................................   2   0   S04
........................................................uauuuugaugacaaguauu......................................   1   0   S04
........................................................uauuuugaugacaaguauu......................................   1   0   S05
.........................................................auuuugaugacaaguauu......................................   1   0   S04
.........................................................auuuugaugacaaguauuuu....................................   1   0   S06
.........................................................auuuugaugacaaguauuuuc...................................   1   0   S05
.........................................................auuuugaugacaaguauuuucgg.................................   1   0   S04
.........................................................auuuugaugacaaguauuuucgg.................................   3   0   S06
.........................................................auuuugaugacaaguauuuucgg.................................   1   0   S01
.........................................................auuuugaugacaaguauuuucgg.................................   1   0   S02
.........................................................auuuugaugacaaguauuuucgg.................................   1   0   S05
.........................................................auuuugaugacaaguauuuucgga................................   1   0   S05
.........................................................auuuugaGgacaaguauuuucgga................................   1   1   S04
.........................................................auuuugaugacaaguauuuucggaA...............................   1   1   S04
..........................................................uuuugaugacaaguauuu.....................................   1   0   S04
..........................................................uuuugaugacaaguauuu.....................................   3   0   S06
..........................................................uuuugaugacaaguauuu.....................................   1   0   S05
..........................................................uuuugaugacaaguauuu.....................................   3   0   S01
..........................................................uuuugaugacaaguauuuu....................................   1   0   S06
..........................................................uuuugaugacaaguauuuu....................................   1   0   S01
..........................................................uuuugaugacaaguauuuu....................................   2   0   S04
..........................................................uuuugaugacaaguauuuu....................................   4   0   S05
..........................................................uuuugaugacaaguauuuuc...................................   2   0   S04
..........................................................uuuugaugacaaguauuuuc...................................   1   0   S01
..........................................................uuuugaugacaaguauuuucg..................................   3   0   S05
..........................................................uuuugaugacaaguauuuucg..................................   2   0   S02
..........................................................uuuugaugacaaguauuuucg..................................   1   0   S01
..........................................................uuuugaugacaaguauuuucg..................................   5   0   S04
..........................................................uuuugaugacaaguauuuucgg.................................   1   0   S04
..........................................................uuuugaugacaaguauuuucgg.................................   1   0   S05
..........................................................uuuugaugacaaguauuuucgga................................   1   0   S05
...........................................................uuugaugacaaguauuuu....................................   1   0   S03
...........................................................uuugaugacaaguauuuucg..................................   1   0   S01
...........................................................uuugaugacaaguauuuucgg.................................   1   0   S03
...........................................................uuugaugacaaguauuuucgg.................................   1   0   S01
...........................................................uuugaugacaaguauuuucgga................................   1   0   S04
............................................................uugaugacaaguauuuucgg.................................   1   0   S05
............................................................uugaugacaaguauuuucgg.................................   1   0   S04
.............................................................ugaugacaaguauuuucgga................................   1   0   S05
...............................................................augacaaguauuuucgga................................   2   0   S01
..................................................................acaaguauuuucggacgaaaggag.......................   1   0   S05
......................................................................guauuuucggacgaaaggaguau....................   1   0   S04
```

Provisional ID	:	ta_iwgsc_3b_v1_10416116_2597336
Score total	:	116.7
Score for star read(s)	:	3.9
Score for read counts	:	108.7
Score for mfe	:	2.5
Score for randfold	:	1.6
Score for cons. seed	:	
Total read count	:	225
Mature read count	:	291
Loop read count	:	0
Star read count	:	357

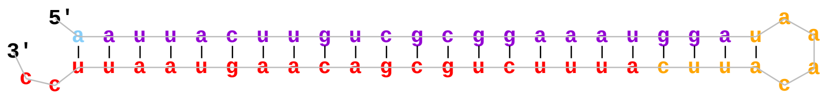

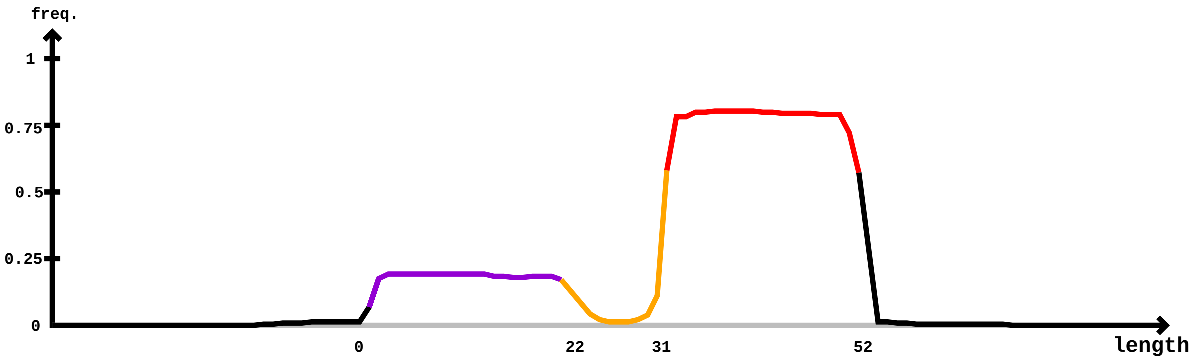

Star Mature

```
5'- uugaccagacacacauacucccucaguccggaauuacuugucgcggaaauggauaaacauucauuucugcgacaaguaauuccagaugaagggaguacuuuuguuaguuugaua   -3'   obs
    uugaccagacacacauacucccucaguccggaauuacuugucgcggaaauggauaaacauucauuucugcgacaaguaauuccagaugaagggaguacuuuuguuaguuugaua         exp
    .....(((((..((((((((((..(((.((((((((((((((((((((((((((....))))))))))))))))))))))))).)))..))))))))....)))..)))))...   reads   mm   sample
    ....................ccucaguccggaauuacuugucgc......................................................................   1   0   S03
    ......................ucaguccggaauuacuugucgcgg....................................................................   1   0   S05
    .........................guccggaauuacuugucgc......................................................................   1   0   S04
    ...............................aauuacuugucgcggaaaug...............................................................   1   0   S02
    ...............................aauuacuugucgcggaaaugg..............................................................   2   0   S06
    ...............................aauuacuugucgcggaaaugg..............................................................   1   0   S04
    ...............................aauuacuugucgcggaaaugg..............................................................   7   0   S05
    ...............................aauuacuugucgcggaaauggaua...........................................................   1   0   S06
    ...............................aauuacuugucgcggaaauggaua...........................................................   1   0   S04
    ................................auuacuugucgcggaaaug...............................................................   1   0   S03
    ................................auuacuugucgcggaaaug...............................................................   1   0   S06
    ................................auuacuugucgcggaaaugg..............................................................   2   0   S03
    ................................auuacuugucgcggaaaugg..............................................................   1   0   S01
    ................................auuacuugucgcggaaaugg..............................................................   2   0   S02
    ................................auuacuugucgcggaaaugga.............................................................   3   0   S03
    ................................auuacuugucgcggaaaugga.............................................................   3   0   S05
    ................................auuacuugucgcggaaaugga.............................................................   1   0   S02
    ................................auuacuugucgcggaaaugga.............................................................   3   0   S01
    ................................auuacuugucgcggaaaugga.............................................................   1   0   S04
    ................................auuacuugucgcggaaaugga.............................................................   2   0   S06
    ................................auuacuugucgcggaaauggaua...........................................................   1   0   S05
    ................................auuacuugucgcggaaauggaua...........................................................   1   0   S06
    ................................auuacuugucgcggaaauggauGa..........................................................   1   1   S05
    ................................auuacuugucgcggaaauggauaa..........................................................   1   0   S05
    ................................auuacuugucgcggaaauggauGa..........................................................   1   1   S04
    .................................uuacuugucgcggaaaugg..............................................................   1   0   S03
    .................................uuacuugucgcggaaauggau............................................................   1   0   S02
    .................................uuacuugucgcggaaauggaua...........................................................   1   0   S04
    .................................uuacuugucgcggaaauggauGa..........................................................   1   1   S03
    ................................................auggauaaacauucauuucugcga..........................................   1   0   S04
    ........................................................acauucauuucugcgaca........................................   1   0   S01
    ........................................................acauucauuucugcgacaaguaa...................................   1   0   S03
    ...........................................................uucauuucugcgacaaguaauucc...............................   1   0   S04
```

Star Mature			
uugaccagacacacauacucccucaguccggaauuacuugucgcggaaauggauaaacauucauuucugcgacaaguaauuccagaugaagggaguacuuuuguuaguuugaua			
....uucauuucugcgacaaguaauucc...............................	1	0	S05
....ucauuucugcgacaagua....................................	1	0	S03
....ucauuucugcgacaaguaauu.................................	1	0	S01
....ucauuucugcgacaaguaauuc................................	1	0	S05
....ucauuucugcgacaaguaauucca..............................	1	0	S03
....cauuucugcgacaaguaauuc................................	2	0	S06
....cauuucugcgacaaguaauuc................................	3	0	S02
....cauuucugcgacaaguaauuc................................	6	0	S01
....cauuucugcgacaaguaauuc................................	1	0	S03
....cauuucugcgacaaguaauuc................................	3	0	S04
....cauuucugcgacaaguaauuc................................	1	0	S05
....cauuucugcgacaaguaauucca..............................	1	0	S05
....auuucugcgacaaguaauu.................................	1	0	S04
....auuucugcgacaaguaauu.................................	1	0	S06
....auuucugcgacaaguaauuc................................	1	0	S04
....auuucugcgacaaguaauucc...............................	22	0	S02
....auuucugcgacaaguaauucc...............................	13	0	S03
....auuucugcgacaaguaauucc...............................	18	0	S05
....auuucugcgacaaguaauucc...............................	13	0	S04
....auuucugcgacaaguaauucc...............................	7	0	S06
....auuucugcgacaaguaauucc...............................	33	0	S01
....auuucCgcgacaaguaauuccaga............................	1	1	S04
....uuucugcgacaaguaauu.................................	1	0	S01
....uuucugcgacaaguaauu.................................	1	0	S03
....uuucugcgacaaguaauu.................................	2	0	S02
....uuucugcgacaaguaauu.................................	6	0	S05
....uuucugcgacaaguaauu.................................	3	0	S04
....uuucugcgacaaguaauuc................................	3	0	S04
....uuucugcgacaaguaauuc................................	2	0	S02
....uuucugcgacaaguaauuc................................	8	0	S01
....uuucugcgacaaguaauuc................................	4	0	S05
....uuucugcgacaaguaauucc...............................	5	0	S05
....uuucugcgacaaguaauucc...............................	1	0	S02
....uuucugcgacaaguaauucc...............................	3	0	S04
....uuucugcgacaaguaauucc...............................	4	0	S01
....uuucugcgacaaguaauucc...............................	2	0	S06
....uuucugcgacaaguaauucca..............................	2	0	S05
....ucugcgacaaguaauucc...............................	1	0	S03
....ucugcgacaaguaauucc...............................	1	0	S01
....ucugcgacaaguaauucc...............................	2	0	S05
....ugcgacaaguaauuccagaug..........................	1	0	S04
....uuccGgaugaagggaguac................	1	1	S05

Provisional ID	:	ta_iwgsc_3b_v1_10416116_2597350
Score total	:	2.6
Score for star read(s)	:	-1.3
Score for read counts	:	0
Score for mfe	:	2.3
Score for randfold	:	1.6
Score for cons. seed	:	
Total read count	:	169
Mature read count	:	196
Loop read count	:	0
Star read count	:	257

novel-m0776-3p

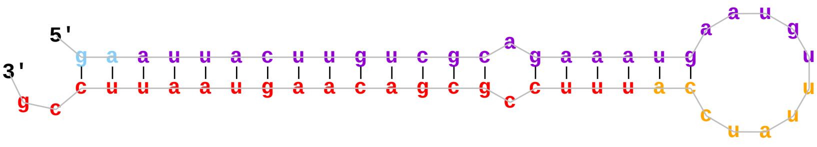

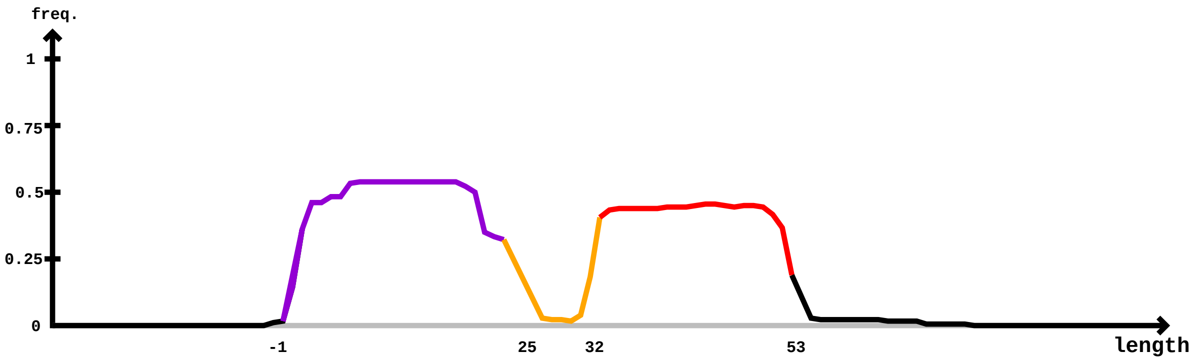

Star Mature

5'- acaaaaguacucccuucaucuggaauuacuugucgcagaaaugaauguuuauccauuuccgcgacaaguaauuccggacugagggaguaugugugucuggucaacgaacuuguc -3'	obs		
acaaaaguacucccuucaucuggaauuacuugucgcagaaaugaauguuuauccauuuccgcgacaaguaauuccggacugagggaguaugugugucuggucaacgaacuuguc	exp		
(((...((((((((((..(((((((((((((((((.((((((.........)))))).)))))))))))))))))..))))))))))...)))....................	reads	mm	sample
.....................ggaauuacuugucgcagaaa...	1	0	S06
.....................Agaauuacuugucgcagaaauga..	1	1	S05
......................gaauuacuugucgcagaaa...	1	0	S05
.......................aauuacuugucgcagaaa...	1	0	S04
.......................aauuacuugucgcagaaUu..	2	1	S05
.......................aauuacuugucgcagaaaug...	2	0	S02
.......................aauuacuugucgcagaaaug...	3	0	S03
.......................aauuacuugucgcagaaaug...	2	0	S05
.......................aauuacuugucgcagaaaCg...	1	1	S06
.......................aauuacuugucgcagaaaug...	3	0	S06
.......................aauuacuugucgcagaaaug...	5	0	S01
.......................aauuacuugucgcagaaaugaau..	1	0	S06
.......................aauuacuugucgcagaaaugaau..	1	0	S04
.......................aauuacuugucgcagaaaugaau..	1	0	S05
.......................aauuacuugucgcagaaaugaaug...	1	0	S04
........................auuacuugucgcagaaau..	1	0	S03
........................auuacuugucgcagaaau..	1	0	S01
........................auuacuugucgcagaaaug...	1	0	S06
........................auuacuugucgcagaaaug...	4	0	S01
........................auuacuugucgcagaaaug...	2	0	S05
........................auuacuugucgcagaaaug...	1	0	S03
........................auuacuugucgcagaaaugaa...	1	0	S02
........................auuacuugucgcagaaaugaau..	1	0	S03
........................auuacuugucgcagaaaugaaug...	2	0	S03
........................auuacuugucgcagaaaugaaug...	1	0	S06
........................auuacuugucgcagaaaugaaugu..	6	0	S04
........................auuacuugucgcagaaaugaaugu..	1	0	S03
........................auuacuugucgcagaaaugaaugu..	10	0	S05
........................auuacuugucgcagaaaugaaugu..	5	0	S02
........................auuacuugucgcagaaaugaaugu..	2	0	S06
.........................uuacuugucgcagaaaug...	1	0	S04
.........................uuacuugucgcagaaaug...	2	0	S01
.........................uuacuugucgcagaaauga..	2	0	S04

Star Mature

```
acaaaaguacucccuucaucuggaauuacuugucgcagaaaugaauguuuauccauuuccgcgacaaguaauuccggacugagggaguaugugugucuggucaacgaacuuguc
.........................uuacuugucgcagaaauga......................................................................   1    0    S05
.........................uuacuugucgcagaaaugaa.....................................................................   1    0    S05
.........................uuacuugucgcUgaaaugaau....................................................................   1    1    S05
.........................uuacuugucgcagaaaugaau....................................................................   1    0    S01
.........................uuacuugucgcagaaaugaaug...................................................................   1    0    S06
.........................uuacuugucgcagaaaugaaugA..................................................................   1    1    S04
.........................uuacuugucgcagaaaugaaugu..................................................................   1    0    S02
.........................uuacuugucgcagaaaugaaugu..................................................................   1    0    S06
.........................uuacuugucgcagaaaugaaugu..................................................................   1    0    S04
.........................uuacuugucgcagaaaugaaugu..................................................................   3    0    S05
.........................uuacuugucgcagaaaugaauguu.................................................................   1    0    S02
...........................acuugucgcagaaaugaaug...................................................................   2    0    S05
...........................acuugucgcagaaaugaaugu..................................................................   2    0    S04
.............................uugucgcagaaaugaaug...................................................................   1    0    S05
.............................uugucgcagaaaugaaugu..................................................................   3    0    S04
.............................uugucgcagaaaugaaugu..................................................................   1    0    S01
.............................uugucgcagaaaugaaugu..................................................................   1    0    S05
.............................uugucgcagaaaugaaugu..................................................................   1    0    S06
.............................uugucgcagaaaugaauguu.................................................................   1    0    S04
.............................uugucgcagaaaugaauguuuau..............................................................   1    0    S05
..............................ugucgcagaaaugaauguuu................................................................   1    0    S06
............................................auguuuauccauuuccgcgacaag..............................................   1    0    S05
................................................uuauccauuuccgcgacaagu.............................................   1    0    S05
................................................uuauccauuuccgcgacaaguaau..........................................   1    0    S05
.....................................................cauuuccgcgacaaguaauu.........................................   1    0    S01
.....................................................cauuuccgcgacaaguaauuc........................................   1    0    S04
.....................................................cauuuccgcgacaaguaauuc........................................   1    0    S01
.....................................................cauuuccgcgacaaguaauuccg......................................   1    0    S04
......................................................auuuccgcgacaaguaauucc.......................................   2    0    S03
......................................................auuuccgcgacaaguaauucc.......................................   2    0    S05
......................................................auuuccgcgacaaguaauucc.......................................   2    0    S01
......................................................auuuccgcgacaaguaauucc.......................................   10   0    S02
......................................................auuuccgcgacaaguaauucc.......................................   6    0    S06
......................................................auuuccgcgacaaguaauucc.......................................   2    0    S04
......................................................auuuccgcgacaaguaauuccg......................................   1    0    S04
......................................................auuuccgcgacaaguaauuccAga....................................   1    1    S04
.......................................................uuuccgcgacaaguaauu.........................................   1    0    S05
.......................................................uuuccgcgacaaguaauu.........................................   2    0    S04
.......................................................uuuccgcgacaaguaauu.........................................   1    0    S01
.......................................................uuuccgcgacaaguaauuc........................................   1    0    S02
.......................................................uuuccgcgacaaguaauuc........................................   5    0    S01
.......................................................uuuccgcgacaaguaauuc........................................   1    0    S05
.......................................................uuuccgcgacaaguaauucc.......................................   1    0    S05
.......................................................uuuccgcgacaaguaauucc.......................................   3    0    S04
.......................................................uuuccgcgacaaguaauucc.......................................   1    0    S03
.......................................................uuuccgcgacaaguaauucc.......................................   1    0    S01
.......................................................uuuccgcgacaaguaauuccg......................................   1    0    S01
.......................................................uuuccgcgacaaguaauuccg......................................   7    0    S04
.......................................................uuuccgcgacaaguaauuccg......................................   2    0    S02
.......................................................uuuccgcgacaaguaauuccg......................................   10   0    S05
.......................................................uuuccgcgacaaguaauuccg......................................   2    0    S06
.......................................................uuuccgcgacaaguaauuccg......................................   1    0    S03
........................................................uuccgcgacaaguaauucc.......................................   1    0    S05
........................................................uuccgcgacaaguaauucc.......................................   1    0    S02
........................................................uuccgcgacaaguaauuccg......................................   1    0    S04
........................................................uuccgcgacaaguaauuccg......................................   1    0    S05
........................................................uuccgcgacaaguaauuccg......................................   1    0    S06
.........................................................uccgcgacaaguaauuccg......................................   1    0    S06
..............................................................gacaaguaauuccggacAgaggg.............................   1    1    S05
.................................................................aaguaauuccggacugagggagua.........................   1    0    S05
..................................................................aguaauuccggacugagggagua.........................   1    0    S02
......................................................................auuccggacugagggaguaugugu....................   1    0    S05
```

Provisional ID	:	ta_iwgsc_3b_v1_10468405_2642660
Score total	:	2.8
Score for star read(s)	:	-1.3
Score for read counts	:	0
Score for mfe	:	2.5
Score for randfold	:	1.6
Score for cons. seed	:	
Total read count	:	1004
Mature read count	:	990
Loop read count	:	0
Star read count	:	21

novel-m0467-5p

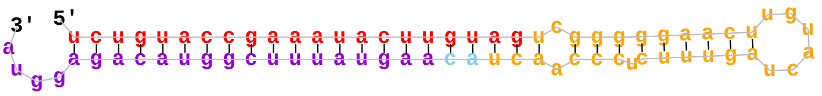

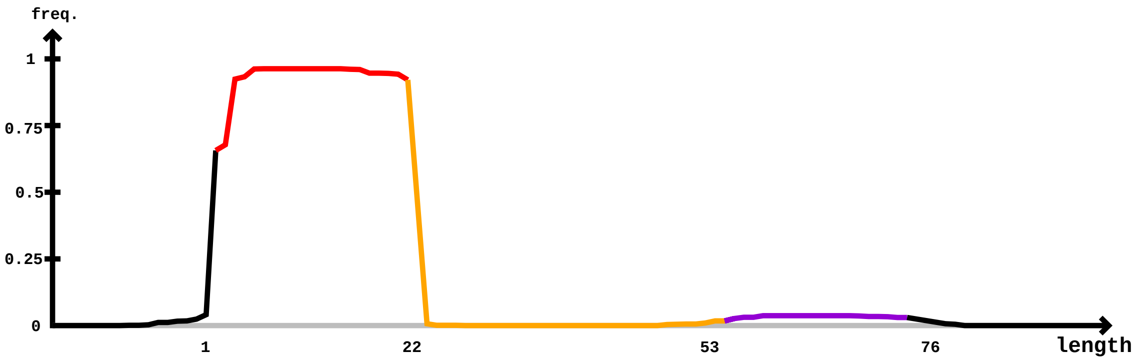

Mature **Star**

```
5'- cauguaaguacucccucuguaccgaaauacuuguagucgggggaacuuguacuaguuucucccaacuacaaguauuucgguacagagguaguaguuagcuagauuugu  -3'  obs
    cauguaaguacucccucuguaccgaaauacuuguagucgggggaacuuguacuaguuucucccaacuacaaguauuucgguacagagguaguaguuagcuagauuugu       exp
    ....(((.((((.((((((((((((((((((((((((.(((((((((......)))))).))).)))))))))))))))))))))))).)))).)))...........  reads  mm  sample
    ......aguacucccucuguaccgaaaua...............................................................................  1  0  S05
    ........uacucccucuguaccgaaaua...............................................................................  1  0  S04
    ........uacucccucuguaccgaaauac..............................................................................  1  0  S01
    .........acucccucuguaccgaaauacu.............................................................................  1  0  S06
    .........acucccucuguaccgaaauacu.............................................................................  1  0  S03
    .........acucccucuguaccgaaauacu.............................................................................  2  0  S05
    .........acucccucuguaccgaaauacu.............................................................................  1  0  S01
    .........acucccucuguaccgaaauacu.............................................................................  1  0  S04
    .........acucccucuguaccgaaauacu.............................................................................  3  0  S02
    ...........ucccucuguaccgaaauacu.............................................................................  1  0  S03
    ...........ucccucuguaccgaaauacu.............................................................................  2  0  S05
    ...........ucccucuguaccgaaauacu.............................................................................  1  0  S06
    ...........ucccucuguaccgaaauacu.............................................................................  1  0  S04
    ............cccucuguaccgaaauacuug...........................................................................  1  0  S05
    .............ccucuguaccgaaauacuugua.........................................................................  3  0  S05
    .............ccucuguaccgaaauacuuguag........................................................................  1  0  S03
    .............ccucuguaccgaaauacuuguag........................................................................  1  0  S01
    .............ccucuguaccgaaauacuuguagu.......................................................................  1  0  S05
    .............ccucuguaccgaaauacuuguagu.......................................................................  1  0  S01
    ..............cucuguaccgaaauacuugu..........................................................................  1  0  S03
    ..............cucuguaccgaaauacuugua.........................................................................  3  0  S05
    ..............cucuguaccgaaauacuugua.........................................................................  1  0  S03
    ..............cucuguaccgaaauacuugua.........................................................................  4  0  S06
    ..............cucuguaccgaaauacuugua.........................................................................  2  0  S01
    ..............cucuguaccgaaauacuugua.........................................................................  2  0  S02
    ..............cucuguaccgaaauacuugua.........................................................................  2  0  S04
    ..............cucuguaccgaaauacuuguag........................................................................  1  0  S04
    ..............cucuguaccgaaauacuuguagu.......................................................................  1  0  S04
    ...............ucuguaccgaaauacuugu..........................................................................  1  0  S05
    ...............ucuguaccgaaauacuugu..........................................................................  1  0  S06
    ...............ucuguaccgaaauacuugua.........................................................................  2  0  S02
    ...............ucuguaccgaaauacuugua.........................................................................  3  0  S06
    ...............ucuguaccgaaauacuuguag........................................................................  90  0  S06
```

```
               Mature                                            Star
cauguaaguacucccucuguaccgaaauacuuguagucgggggaacuuguacuaguuucucccaacuacaaguauuucgguacagagguaguaguuagcuagauuugu
...............ucuguaccgaaauacuuguag........................................................................   34   0   S02
...............ucuguaccgaaauacuuguag........................................................................   29   0   S01
...............ucuguaccgaaauacuuguag........................................................................   40   0   S03
...............ucuguaccgaaauacuuguag........................................................................   56   0   S05
...............ucuguaccgaaauacuuguag........................................................................   31   0   S04
...............ucuguaccgaaauacuuguagu.......................................................................   34   0   S03
...............ucuguaccgaaauacuuguagu.......................................................................   45   0   S02
...............ucuguaccgaaauacuuguagu.......................................................................   48   0   S04
...............ucuguaccgaaauacuuguagu.......................................................................   60   0   S06
...............ucuguaccgaaauacuuguagu.......................................................................   89   0   S05
...............ucuguaccgaaauacuuguagu.......................................................................   70   0   S01
................cuguaccgaaauacuuguag........................................................................   2    0   S03
................cuguaccgaaauacuuguag........................................................................   3    0   S06
................cuguaccgaaauacuuguag........................................................................   1    0   S05
................cuguaccgaaauacuuguag........................................................................   1    0   S04
................cuguaccgaaauacuuguagu.......................................................................   2    0   S04
................cuguaccgaaauacuuguagu.......................................................................   3    0   S06
................cuguaccgaaauacuuguagu.......................................................................   5    0   S03
................cuguaccgaaauacuuguagu.......................................................................   5    0   S05
.................uguaccgaaauacuuguag........................................................................   8    0   S03
.................uguaccgaaauacuuguag........................................................................   28   0   S06
.................uguaccgaaauacuuguag........................................................................   9    0   S02
.................uguaccgaaauacuuguag........................................................................   12   0   S01
.................uguaccgaaauacuuguag........................................................................   9    0   S04
.................uguaccgaaauacuuguag........................................................................   7    0   S05
.................uguaccgaaauacuuguagu.......................................................................   13   0   S03
.................uguaccgaaauacuuguagu.......................................................................   13   0   S04
.................uguaccgaaauacuuguagu.......................................................................   22   0   S05
.................uguaccgaaauacuuguagu.......................................................................   37   0   S02
.................uguaccgaaauacuuguagu.......................................................................   54   0   S06
.................uguaccgaaauacuuguagu.......................................................................   35   0   S01
.................uguaccgaaauacuuguaguc......................................................................   2    0   S05
.................uguaccgaaauacuuguaguc......................................................................   2    0   S02
.................uguaccgaaauacuuguaguc......................................................................   1    0   S03
.................uguaccgaaauacuuguagCcggg...................................................................   1    1   S01
..................guaccgaaauacuuguag........................................................................   4    0   S06
..................guaccgaaauacuuguagu.......................................................................   1    0   S01
..................guaccgaaauacuuguagu.......................................................................   1    0   S03
..................guaccgaaauacuuguagu.......................................................................   2    0   S06
..................guaccgaaauacuuguagu.......................................................................   1    0   S05
...................uaccgaaauacuuguagu.......................................................................   2    0   S02
...................uaccgaaauacuuguagu.......................................................................   10   0   S06
...................uaccgaaauacuuguagu.......................................................................   4    0   S03
...................uaccgaaauacuuguagu.......................................................................   5    0   S05
...................uaccgaaauacuuguagu.......................................................................   5    0   S01
...................uaccgaaauacuuguagu.......................................................................   4    0   S04
....................accgaaauacuuguaguc......................................................................   1    0   S02
..............................................................caacuacaaguauuucggua..........................   1    0   S06
..............................................................caacuacaaguauuucgguac.........................   2    0   S06
..............................................................caacuacaaguauuucgguacaga......................   1    0   S03
...............................................................aacuacaaguauuucgguacag.......................   1    0   S06
................................................................acuacaaguauuucgguacaga......................   1    0   S02
..................................................................uacaaguauuucgguacaga......................   1    0   S06
..................................................................uacaaguauuucgguacagagg....................   1    0   S02
..................................................................uacaaguauuucgguacagagg....................   1    0   S05
..................................................................uacaaguauuucgguacagagg....................   1    0   S06
...................................................................acaaguauuucgguacagagg....................   1    0   S02
...................................................................acaaguauuucgguacagagg....................   2    0   S05
...................................................................acaaguauuucgguacagagg....................   1    0   S01
...................................................................acaaguauuucgguacagaggu...................   1    0   S05
...................................................................acaaguauuucgguacagaggu...................   1    0   S03
...................................................................acaaguauuucgguacagaggua..................   1    0   S04
...................................................................acaaguauuucgguacagagguagua...............   1    0   S01
.....................................................................aaguauuucgguacagaggua..................   4    0   S02
.....................................................................aaguauuucgguacagaggua..................   1    0   S05
.....................................................................aaguauuucgguacagaggua..................   2    0   S01
.....................................................................aaguauuucgguacagaggua..................   1    0   S03
.....................................................................aaguauuucgguacagaggua..................   1    0   S04
......................................................................aguauuucgguacagaggua..................   2    0   S01
......................................................................aguauuucgguacagagguag.................   2    0   S01
```

Mature **Star**

cauguaaguacucccucuguaccgaaauacuuguagucgggggaacuuguacuaguuucucccaacuacaaguauuucgguacagagguaguaguuagcuagauuugu			
....aguauuucgguacagagguag.................	1	0	S05
....uauuucgguacagagguagu...............	1	0	S02
....uauuucgguacagagguagu...............	1	0	S06
....uauuucgguacagagguagua..............	1	0	S06
....uauuucgguacagagguagua..............	1	0	S04
....uauuucgguacagagguagua..............	2	0	S02

Provisional ID	:	ta_iwgsc_3dl_v1_6911113_3129623
Score total	:	2.7
Score for star read(s)	:	-1.3
Score for read counts	:	0
Score for mfe	:	2.4
Score for randfold	:	1.6
Score for cons. seed	:	
Total read count	:	359
Mature read count	:	462
Loop read count	:	0
Star read count	:	138

novel-m0883-3p

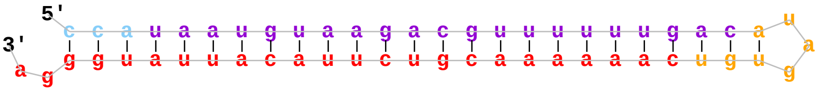

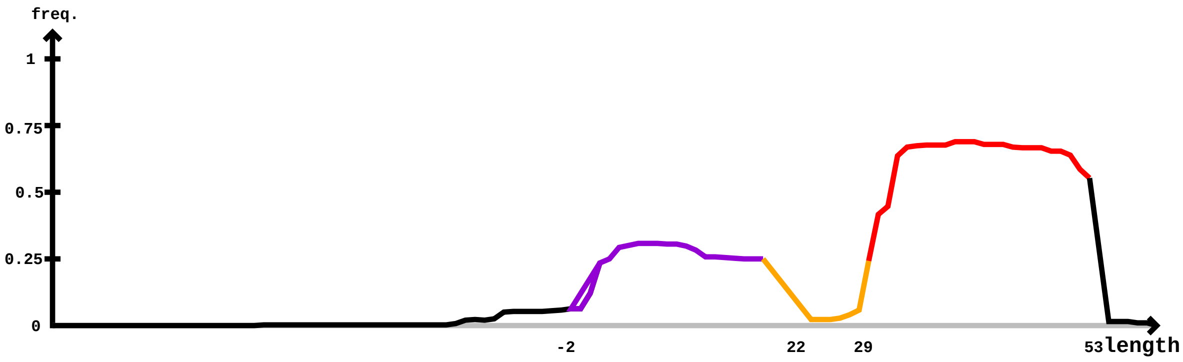

Star Mature

```
5'- auaugagaugauaaaguuacacucgauacguauaauuaguacucccuccgucccauaauguaagacguuuuuugacauagugucaaaaaacgucuuacauuaugggacggagg  -3'   obs
    auaugagaugauaaaguuacacucgauacguauaauuaguacucccuccgucccauaauguaagacguuuuuugacauagugucaaaaaacgucuuacauuaugggacggagg         exp
    ...((((.((.........))))))....((((.....))))..(((((((((((((((((((((((((((((((((...)))))))))))))))))))))))))))))))))   reads   mm   sample
    ....................acucgauacguauaauuaguacu......................................................................   1       0    S05
    ........................................acucccuccgucccauaaugua...................................................   1       0    S02
    ........................................acucccuccgucccauaauguaaga................................................   1       0    S02
    .........................................cucccuccgucccauaauguaag.................................................   1       0    S03
    .........................................cucccuccgucccauaauguaag.................................................   1       0    S01
    .........................................cucccuccgucccauaauguaag.................................................   1       0    S06
    .........................................cucccuccgucccauaauguaaga................................................   1       0    S05
    .........................................cucccuccgucccauaauguaaga................................................   1       0    S06
    ..........................................ucccuccgucccauaauguaagac...............................................   1       0    S06
    ............................................ccuccgucccauaauguaagac...............................................   1       0    S03
    ............................................ccuccgucccauaauguaagacgu.............................................   1       0    S05
    .............................................cuccgucccauaauguaaga................................................   1       0    S04
    .............................................cuccgucccauaauguaaga................................................   1       0    S02
    .............................................cuccgucccauaauguaagac...............................................   1       0    S01
    .............................................cuccgucccauaauguaagac...............................................   4       0    S02
    .............................................cuccgucccauaauguaagac...............................................   1       0    S06
    .............................................cuccgucccauaauguaagac...............................................   2       0    S04
    ..............................................uccgucccauaauguaaga................................................   1       0    S01
    ..................................................ucccauaauguaagacguu............................................   1       0    S04
    ...................................................cccauaauguaagacguu............................................   1       0    S06
    ....................................................ccauaauguaagacguuuuuugac.....................................   1       0    S06
    ....................................................ccauaauguaagacguuuuuugaca....................................   1       0    S05
    ......................................................auaauguaagacguuuuuuga......................................   1       0    S05
    ......................................................auaauguaagacguuuuuuga......................................   1       0    S01
    ......................................................auaauguaagacguuuuuugac.....................................   1       0    S05
    ......................................................auaauguaagacguuuuuugac.....................................   1       0    S04
    ......................................................auaauguaagacguuuuuugaca....................................   2       0    S02
    ......................................................auaauguaagacguuuuuugaca....................................   5       0    S04
    ......................................................auaauguaagacguuuuuugaca....................................   4       0    S01
    ......................................................auaauguaagacguuuuuugaca....................................   3       0    S05
    ......................................................auaauguaagacgAuuuuugaca....................................   1       1    S02
    ......................................................auaauguaagacguuuuuugaca....................................   4       0    S06
    .......................................................uaauguaagacguuuuuug.......................................   1       0    S02
```

	Star / Mature			
	auaugagaugauaaaguuacacucgauacguauaauuaguacucccuccgucccca**uaauguaagacguuuuuugac**auagugu**caaaaaacgucuuacauuauggga**cggagg			
	....uaauguaagacguuuuuug.......................................	3	0	S04
	....uaauguaagacguuuuuuga......................................	1	0	S04
	....uaauguaagacguuuuuuga......................................	1	0	S06
	....uaauguaagacguuuuuuga......................................	2	0	S05
	....uaauguaagacguuuuuuga......................................	1	0	S01
	....uaauguaagacguuuuuugCc.....................................	1	1	S02
	....uaauguaagacguuuuuugCc.....................................	1	1	S05
	....uaauguaagacguuuuuugac.....................................	9	0	S04
	....uaauguaagacguuuuuugac.....................................	4	0	S02
	....uaauguaagacguuuuuugac.....................................	8	0	S05
	....uaauguaagacguuuuuugac.....................................	1	0	S03
	....uaauguaagacguuuuuugCc.....................................	1	1	S06
	....uaauguaagacguuuuuugac.....................................	2	0	S01
	....uaauguaagacguuuuuugaca....................................	3	0	S04
	....uaauguaagacguuuuuugaca....................................	2	0	S05
	....uaauguaagacguuuuuugaca....................................	1	0	S06
	....uaauguaagacguuuuuugaca....................................	3	0	S01
	....aauguaagacguuuuuuga......................................	1	0	S02
	....aauguaagacguuuuuugac.....................................	1	0	S06
	....aauguaagacguuuuuugac.....................................	1	0	S01
	....aauguaagacguuuuuugaca....................................	1	0	S01
	....aauguaagacguuuuuugaca....................................	2	0	S02
	....auguaagacguuuuuuga......................................	1	0	S02
	....auguaagacguuuuuuga......................................	1	0	S05
	....auguaagacguuuuuugac.....................................	1	0	S03
	....auguaagacguuuuuugaca....................................	5	0	S02
	....auguaagacguuuuuugaca....................................	1	0	S06
	....auguaagacguuuuuugaca....................................	4	0	S01
	....auguaagacguuuuuugaca....................................	2	0	S04
	....auguaagacguuuuuugaca....................................	1	0	S03
	....auguaagacgCuuuuugacau...................................	1	1	S04
	....uguaagacguuuuuugac.....................................	1	0	S02
	....uguaagacguuuuuugac.....................................	2	0	S05
	....guaagacguuuuuugaca....................................	1	0	S01
	....guaagacguuuuuugaca....................................	1	0	S05
	....guaagacguuuuuugaca....................................	1	0	S04
	....uagugucaaaaaacgucu..................	1	0	S05
	....uagugucaaaaaacgucu..................	2	0	S04
	....uagugucaaaaaacgucu..................	1	0	S06
	....uagugucaaaaaacgucuuac...............	3	0	S04
	....uagugucaaaaaacgucuuac...............	1	0	S05
	....agugucaaaaaacgucuuaca..............	1	0	S05
	....ugucaaaaaacgucuuacauuaug.........	1	0	S06
	....ugucaaaaaacgucuuacauuaug.........	1	0	S01
	....gucaaaaaacgucuuacauuaugg........	2	0	S03
	....gucaaaaaacgucuuacauuaugg........	1	0	S06
	....gucaaaaaacgucuuacauuaugg........	2	0	S05
	....ucaaaaaacgucuuacauuaugg........	2	0	S05
	....ucaaaaaacgucuuacauuauggg.......	2	0	S05
	....ucaaaaaacgucuuacauuauggg.......	1	0	S02
	....ucaaaaaacgucuuacauuauggg.......	2	0	S04
	....caaaaaacgucuuacauua...........	1	0	S04
	....caaaaaacgucuuacauuaugg........	2	0	S03
	....caaaaaacgucuuacauuaugg........	1	0	S06
	....caaaaaacgucuuacauuauggg.......	1	0	S06
	....caaaaaacgucuuacauuauggg.......	2	0	S02
	....caaaaaacgucuuacauuauggga......	9	0	S02
	....caaaaaacgucuuacauuauggga......	19	0	S05
	....caaaaaacgucuuacauuauggga......	12	0	S04
	....caaaaaacgucuuacauuauggga......	3	0	S01
	....caaaaaacgucuuacauuauggga......	7	0	S03
	....caaaaaacgucuuaAauuauggga......	1	1	S05
	....caaaaaacgucuuacauuauggga......	15	0	S06
	....aaaaaacgucuuacauua...........	3	0	S04
	....aaaaaacgucuuacauua...........	1	0	S03
	....aaaaaacgucuuacauuaug.........	1	0	S02
	....aaaaaacgucuuacauuaug.........	1	0	S04
	....aaaaaacgucuuacauuaugg........	1	0	S01
	....aaaaaacgucuuacauuaugg........	1	0	S03
	....aaaaaacgucuuacauuaugg........	1	0	S02

Star Mature

```
auaugagaugauaaaguuacacucgauacguauaauuaguacucccuccgucccauaauguaagacguuuuuugacauagugucaaaaaacgucuuacauuaugggacggagg
....................................................................................aaaaaacgucuuacauuaugg........  1  0  S04
....................................................................................aaaaaacgucuuacauuaugg........  3  0  S05
....................................................................................aaaaaacgucuuacauuaugg........  2  0  S06
....................................................................................aaaaaacgucuuacauuauggg.......  1  0  S05
....................................................................................aaaaaacgucuuacauuauggga......  13  0  S04
....................................................................................aaaaaacgucuuacauuauggga......  12  0  S05
....................................................................................aaaaaacgucuuacauuauggga......  8  0  S02
....................................................................................aaaaaacgucuuacauuauggga......  3  0  S03
....................................................................................aaaaaacgucuuacauuauggga......  14  0  S06
....................................................................................aaaaaacgucuuacauuauggga......  3  0  S01
.....................................................................................aaaaacgucuuacauuaugg........  1  0  S06
.....................................................................................aaaaacgucuuacauuauggg.......  1  0  S04
.....................................................................................aaaaacgucuuacauuauggga......  2  0  S06
.....................................................................................aaaaacgucuuacauuauggga......  2  0  S04
.....................................................................................aaaaacgucuuacauuauggga......  3  0  S05
.....................................................................................aaaaacgucuuacauuauggga......  3  0  S02
......................................................................................aaaacgucuuacauuaug.........  2  0  S02
......................................................................................aaaacgucuuacauuauggg.......  2  0  S04
......................................................................................aaaacgucuuacauuauggg.......  1  0  S05
......................................................................................aaaacgucuuacauuauggga......  15  0  S02
......................................................................................aaaacgucuuacauuauggga......  8  0  S04
......................................................................................aaaacgucuuacauuauggga......  12  0  S01
......................................................................................aaaacgucuuacauuauggga......  17  0  S05
......................................................................................aaaacgucuuacauuauggga......  7  0  S03
......................................................................................aaaacgucuuacauuauggga......  11  0  S06
.......................................................................................aaacgucuuacauuaugg........  1  0  S06
.......................................................................................aaacgucuuacauuauggga......  3  0  S01
.......................................................................................aaacgucuuacauuauggga......  3  0  S05
.......................................................................................aaacgucuuacauuauggga......  2  0  S06
.......................................................................................aaacgucuuacauuauggga......  3  0  S03
.......................................................................................aaacgucuuacauuaugggaAgga..  1  1  S05
........................................................................................aacgucuuacauuauggga......  2  0  S06
.........................................................................................acgucuuacauuauggga......  1  0  S02
............................................................................................ucuuacauuaugggacgga..  1  0  S03
............................................................................................ucuuacauuaugggacggagg  1  0  S04
............................................................................................ucuuacauuaugggacggagg  2  0  S03
............................................................................................ucuuacauuaugggacggagg  1  0  S05
```

Provisional ID	:	ta_iwgsc_3b_v1_10517750_2687625
Score total	:	2.5
Score for star read(s)	:	-1.3
Score for read counts	:	0
Score for mfe	:	2.2
Score for randfold	:	1.6
Score for cons. seed	:	
Total read count	:	600
Mature read count	:	480
Loop read count	:	0
Star read count	:	162

novel-m0729-5p

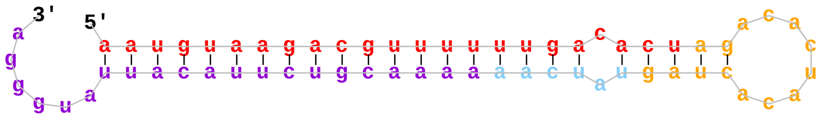

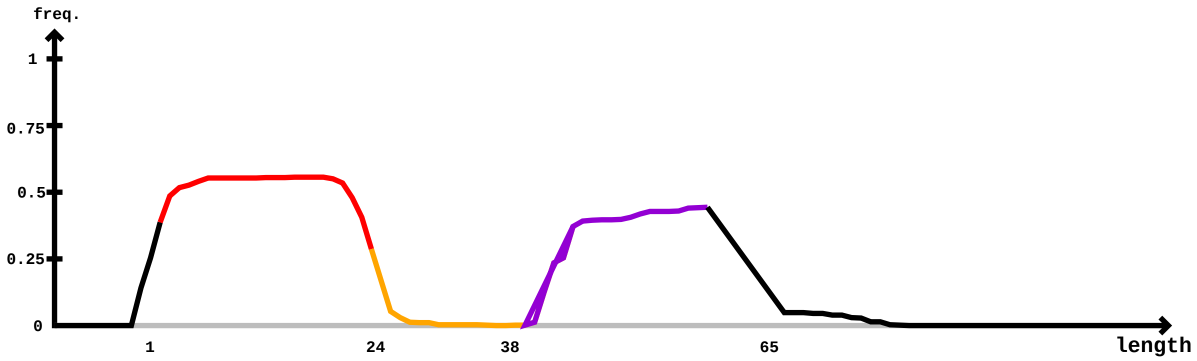

Mature   Star

```
5'- cagucucauaauguaagacguuuuuugacacuagacacuacacuaguaucaaaaaacgucuuacauuaugggacggagggaguacuagguugcaccaugcuucucgcguuuag  -3'  obs
    cagucucauaauguaagacguuuuuugacacuagacacuacacuaguaucaaaaaacgucuuacauuaugggacggagggaguacuagguugcaccaugcuucucgcguuuag       exp
    ..((((((((((((((((((((((((((.(((((........))))).)))))))))))))))))))))))))(..(((((((...(((...))).)))))))..).......  reads  mm  sample
    .......auaauguaagacguuuuuuga.....................................................................................  1  0  S05
    .......auaauguaagacguuuuuuga.....................................................................................  1  0  S01
    .......auaauguaagacguuuuuugac....................................................................................  1  0  S04
    .......auaauguaagacguuuuuugac....................................................................................  1  0  S05
    .......auaauguaagacguuuuuugaca...................................................................................  2  0  S02
    .......auaauguaagacguuuuuugaca...................................................................................  4  0  S06
    .......auaauguaagacgAuuuuugaca...................................................................................  1  1  S02
    .......auaauguaagacguuuuuugaca...................................................................................  4  0  S01
    .......auaauguaagacguuuuuugaca...................................................................................  5  0  S04
    .......auaauguaagacguuuuuugaca...................................................................................  3  0  S05
    .......auaauguaagacguuuuuugacac..................................................................................  19  0  S05
    .......auaauguaagacguuuuuugaGac..................................................................................  1  1  S06
    .......auaauguaagacguuuuuugacac..................................................................................  7  0  S06
    .......auaauguaagacguuuuuugacac..................................................................................  12  0  S04
    .......auaauguaagacguuuuuugacac..................................................................................  3  0  S03
    .......auaauguaagacguuuuuugacac..................................................................................  6  0  S02
    .......auGauguaagacguuuuuugacac..................................................................................  1  1  S04
    .......auaauUuaagacguuuuuugacac..................................................................................  1  1  S05
    .......auaauguaagacguuuuuugacac..................................................................................  7  0  S01
    .......auGauguaagacguuuuuugacacu.................................................................................  1  1  S06
    .......auaauguaagGcguuuuuugacacu.................................................................................  1  1  S01
    .......auaauguaagacguuuuuugacacu.................................................................................  4  0  S04
    .......auaauguaagacguuuuuugacacu.................................................................................  2  0  S05
    .......auaauguaagacguuuuuugacacu.................................................................................  2  0  S02
    ........uaauguaagacguuuuuug......................................................................................  1  0  S02
    ........uaauguaagacguuuuuug......................................................................................  3  0  S04
    ........uaauguaagacguuuuuuga.....................................................................................  1  0  S01
    ........uaauguaagacguuuuuuga.....................................................................................  1  0  S04
    ........uaauguaagacguuuuuuga.....................................................................................  1  0  S06
    ........uaauguaagacguuuuuuga.....................................................................................  2  0  S05
    ........uaauguaagacguuuuuugac....................................................................................  1  0  S03
    ........uaauguaagacguuuuuugCc....................................................................................  1  1  S02
    ........uaauguaagacguuuuuugac....................................................................................  2  0  S01
```

Mature **Star**

```
cagucucauaauguaagacguuuuuugacacuagacacuacacuaguaucaaaaaacgucuuacauuaugggacggagggaguacuagguugcaccaugcuucucgcgcuuuag
........uaauguaagacguuuuuugac.....................................................................................  8  0  S05
........uaauguaagacguuuuuugac.....................................................................................  4  0  S02
........uaauguaagacguuuuuugCc.....................................................................................  1  1  S06
........uaauguaagacguuuuuugCc.....................................................................................  1  1  S05
........uaauguaagacguuuuuugac.....................................................................................  9  0  S04
........uaauguaagacguuuuuugaca....................................................................................  1  0  S06
........uaauguaagacguuuuuugaca....................................................................................  2  0  S05
........uaauguaagacguuuuuugaca....................................................................................  3  0  S04
........uaauguaagacguuuuuugaca....................................................................................  3  0  S01
........uaauguaagacguuuuuugacac...................................................................................  2  0  S04
........uaauguaagacguuuuuugacacu..................................................................................  4  0  S05
........uaauguaagacguuuuuugacacu..................................................................................  6  0  S01
........uaauguaagacguuuuuugacacu..................................................................................  11  0  S04
........uaauguaagacguuuuuugacacu..................................................................................  4  0  S02
.........aauguaagacguuuuuuga......................................................................................  1  0  S02
.........aauguaagacguuuuuugac.....................................................................................  1  0  S01
.........aauguaagacguuuuuugac.....................................................................................  1  0  S06
.........aauguaagacguuuuuugaca....................................................................................  1  0  S01
.........aauguaagacguuuuuugaca....................................................................................  2  0  S02
.........aauguaagacguuuuuugacac...................................................................................  2  0  S05
.........aauguaagacguuuuuugacac...................................................................................  1  0  S02
.........aauguaagacguuuuuugacac...................................................................................  1  0  S06
.........aauguaagacguuuuuugacacu..................................................................................  4  0  S03
.........aauguaagacguuuuuugacacu..................................................................................  9  0  S05
.........aauguaagacguuuuuugacacu..................................................................................  2  0  S06
.........aauguaagacguuuuuugacacu..................................................................................  10  0  S02
.........aauguaagacguuuuuugacacu..................................................................................  4  0  S01
.........aauguaagacguuuuuugacacu..................................................................................  22  0  S04
.........aauguaagacguuuuuugacacua.................................................................................  5  0  S06
.........aauguaagacgAuuuuugacacua.................................................................................  1  1  S04
.........aauguaagacguuuuuugacacua.................................................................................  6  0  S05
.........aauguaagacguuuuuugacacua.................................................................................  2  0  S01
.........aauguaagacguuuuuugacacua.................................................................................  3  0  S02
.........aauguaagacguuuuuugacacua.................................................................................  7  0  S04
..........auguaagacguuuuuuga......................................................................................  1  0  S05
..........auguaagacguuuuuuga......................................................................................  1  0  S02
..........auguaagacguuuuuugac.....................................................................................  1  0  S03
..........auguaagacguuuuuugaca....................................................................................  4  0  S01
..........auguaagacguuuuuugaca....................................................................................  1  0  S03
..........auguaagacguuuuuugaca....................................................................................  2  0  S04
..........auguaagacguuuuuugaca....................................................................................  5  0  S02
..........auguaagacguuuuuugaca....................................................................................  1  0  S06
..........auguaagacguuuuuugCcac...................................................................................  1  1  S04
..........auguaagacguuuuuugacac...................................................................................  1  0  S04
..........auguaagacguuuuuugCcac...................................................................................  1  1  S02
..........auguaagacguuuuuugacac...................................................................................  2  0  S02
..........auguaagacguuuuuugacac...................................................................................  2  0  S06
..........auguaagacguuuuuugacac...................................................................................  2  0  S05
..........auguaagacguuuuuugacac...................................................................................  1  0  S03
..........auguaagacguuuuuugacacu..................................................................................  3  0  S02
..........auguaagacguuuuuugacacu..................................................................................  1  0  S06
..........auguaagacguuuuuugacacu..................................................................................  2  0  S03
..........auguaagacguuuuuugacacu..................................................................................  4  0  S01
..........auguaagacguuuuuugacacu..................................................................................  1  0  S04
..........auguaagacguuuuuugacacu..................................................................................  4  0  S05
..........auguaagacguuuuuugacacua.................................................................................  3  0  S01
..........auguaagacguuuuuugacacua.................................................................................  3  0  S04
..........auguaagacguuuuuugacacua.................................................................................  2  0  S05
..........auguaagacguuuuuugacacua.................................................................................  2  0  S06
..........auguaagacguuuuuugacacuag................................................................................  4  0  S04
..........auguaagacguuuuuugacacuag................................................................................  4  0  S05
..........auguaagacguuuuuugacacuag................................................................................  1  0  S01
..........auguaagacguuuuuugacacuaga...............................................................................  1  0  S05
..........auguaagacguuuuuugacacuagC...............................................................................  1  1  S04
..........auguaagacguuuuuugacacuagC...............................................................................  1  1  S05
...........uguaagacguuuuuugac.....................................................................................  1  0  S02
...........uguaagacguuuuuugac.....................................................................................  2  0  S05
...........uguaagacguuuuuugacacu..................................................................................  2  0  S01
...........uguaagacguuuuuugacacu..................................................................................  1  0  S05
...........uguaagacguuuuuugacacu..................................................................................  2  0  S02
```

Mature **Star**

```
cagucucauaauguaagacguuuuuugacacuagacacuacacuaguaucaaaaaacgucuuacauuaugggacggagggaguacuagguugcaccaugcuucucgcguuuag
...........uguaagacguuuuuugacacu.................................................................................  3  0  S06
...........uguaagacguuuuuugacacu.................................................................................  1  0  S04
...........uguaagacguuuuuugacacua................................................................................  1  0  S05
...........uguaagacguuuuuugacacuag...............................................................................  1  0  S02
...........uguaagacguuuuuugacacuag...............................................................................  1  0  S06
...........uguaagacguuuuuugacacuagC..............................................................................  1  1  S05
...........uguaagacguuuuuugacacuaga..............................................................................  1  0  S02
...........uguaagacguuuuuugacacuaga..............................................................................  1  0  S04
...........uguaagacguuuuuugacacuaga..............................................................................  1  0  S05
...........uguaagacguuuuuugacacuagC..............................................................................  1  1  S06
.............guaagacguuuuuugaca..................................................................................  1  0  S01
.............guaagacguuuuuugaca..................................................................................  1  0  S04
.............guaagacguuuuuugaca..................................................................................  1  0  S05
.............guaagacguuuuuugacacuag..............................................................................  1  0  S05
.............guaagacguuuuuugacacuag..............................................................................  1  0  S03
.............guaagacguuuuuugacacuagac............................................................................  1  0  S04
..............uaagacguuuuuugacac.................................................................................  1  0  S03
..............uaagacguuuuuugacac.................................................................................  1  0  S05
..............uaagacguuuuuugacac.................................................................................  1  0  S02
..............uaagacguuuuuugacacu................................................................................  2  0  S05
..............uaagacguuuuuugacacua...............................................................................  1  0  S05
..............uaagacguuuuuugacacuag..............................................................................  1  0  S01
..............uaagacguuuuuugacacuaga.............................................................................  1  0  S05
..............uaagacguuuuuugacacuaga.............................................................................  1  0  S06
...............aagacguuuuuugacacua...............................................................................  1  0  S05
...............aagacguuuuuugacacuag..............................................................................  1  0  S04
...............aagacguuuuuugacacuagC.............................................................................  1  1  S02
...............aagacguuuuuugacacuagacac..........................................................................  1  0  S06
...............aagacguuuuuugacacuagacac..........................................................................  2  0  S05
...............aagacguuuuuugacacuagacac..........................................................................  1  0  S01
...............aagacguuuuuugacacuagacac..........................................................................  1  0  S04
....................uuuuuugacacuagacacuacacu.....................................................................  1  0  S04
........................uugacacuagacacuacac......................................................................  1  0  S06
..............................................uaucaaaaaacgucuuacauu..............................................  1  0  S04
................................................ucaaaaaacgucuuacauuaugg..........................................  2  0  S05
................................................ucaaaaaacgucuuacauuauggg.........................................  1  0  S02
................................................ucaaaaaacgucuuacauuauggg.........................................  2  0  S04
................................................ucaaaaaacgucuuacauuauggg.........................................  2  0  S05
.................................................caaaaaacgucuuacauua.............................................  1  0  S04
.................................................caaaaaacgucuuacauuaugg..........................................  2  0  S03
.................................................caaaaaacgucuuacauuaugg..........................................  1  0  S06
.................................................caaaaaacgucuuacauuauggg.........................................  1  0  S06
.................................................caaaaaacgucuuacauuauggg.........................................  2  0  S02
.................................................caaaaaacgucuuacauuauggga........................................  15  0  S06
.................................................caaaaaacgucuuaAauuauggga........................................  1  1  S05
.................................................caaaaaacgucuuacauuauggga........................................  9  0  S02
.................................................caaaaaacgucuuacauuauggga........................................  19  0  S05
.................................................caaaaaacgucuuacauuauggga........................................  7  0  S03
.................................................caaaaaacgucuuacauuauggga........................................  3  0  S01
.................................................caaaaaacgucuuacauuauggga........................................  12  0  S04
..................................................aaaaaacgucuuacauua.............................................  1  0  S03
..................................................aaaaaacgucuuacauua.............................................  3  0  S04
..................................................aaaaaacgucuuacauuaug...........................................  1  0  S02
..................................................aaaaaacgucuuacauuaug...........................................  1  0  S04
..................................................aaaaaacgucuuacauuaugg..........................................  1  0  S04
..................................................aaaaaacgucuuacauuaugg..........................................  2  0  S06
..................................................aaaaaacgucuuacauuaugg..........................................  3  0  S05
..................................................aaaaaacgucuuacauuaugg..........................................  1  0  S03
..................................................aaaaaacgucuuacauuaugg..........................................  1  0  S01
..................................................aaaaaacgucuuacauuaugg..........................................  1  0  S02
..................................................aaaaaacgucuuacauuauggg.........................................  1  0  S05
..................................................aaaaaacgucuuacauuauggga........................................  14  0  S06
..................................................aaaaaacgucuuacauuauggga........................................  13  0  S04
..................................................aaaaaacgucuuacauuauggga........................................  8  0  S02
..................................................aaaaaacgucuuacauuauggga........................................  12  0  S05
..................................................aaaaaacgucuuacauuauggga........................................  3  0  S03
..................................................aaaaaacgucuuacauuauggga........................................  3  0  S01
...................................................aaaaacgucuuacauuaugg..........................................  1  0  S06
...................................................aaaaacgucuuacauuauggg.........................................  1  0  S04
...................................................aaaaacgucuuacauuauggga........................................  3  0  S05
```

```
         Mature                                  Star
cagucucauaauguaagacguuuuuugacacuagacacuacacuaguaucaaaaaacgucuuacauuaugggacggagggaguacuagguugcaccaugcuucucgcgcuuuag
...................................................aaaaacgucuuacauuauggga.........................................  2  0  S06
...................................................aaaaacgucuuacauuauggga.........................................  3  0  S02
...................................................aaaaacgucuuacauuauggga.........................................  2  0  S04
....................................................aaaacgucuuacauuaug............................................  2  0  S02
....................................................aaaacgucuuacauuauggg..........................................  2  0  S04
....................................................aaaacgucuuacauuauggg..........................................  1  0  S05
....................................................aaaacgucuuacauuauggga.........................................  7  0  S03
....................................................aaaacgucuuacauuauggga.........................................  12  0  S01
....................................................aaaacgucuuacauuauggga.........................................  8  0  S04
....................................................aaaacgucuuacauuauggga.........................................  17  0  S05
....................................................aaaacgucuuacauuauggga.........................................  15  0  S02
....................................................aaaacgucuuacauuauggga.........................................  11  0  S06
.....................................................aaacgucuuacauuaugg............................................  1  0  S06
.....................................................aaacgucuuacauuauggga.........................................  3  0  S01
.....................................................aaacgucuuacauuauggga.........................................  2  0  S06
.....................................................aaacgucuuacauuauggga.........................................  3  0  S03
.....................................................aaacgucuuacauuauggga.........................................  3  0  S05
.....................................................aaacgucuuacauuaugggaAgga.....................................  1  1  S05
......................................................aacgucuuacauuauggga.........................................  2  0  S06
.......................................................acgucuuacauuauggga.........................................  1  0  S02
.........................................................gucuuacauuaugggacggaggga.................................  1  0  S04
..........................................................ucuuacauuaugggacgga.....................................  1  0  S03
..........................................................ucuuacauuaugggacggagg...................................  1  0  S04
..........................................................ucuuacauuaugggacggagg...................................  1  0  S05
..........................................................ucuuacauuaugggacggagg...................................  2  0  S03
...........................................................cuuacauuaugggacggagggag................................  1  0  S04
...........................................................cuuacauuaugggacggagggagu...............................  1  0  S06
...........................................................cuuacauuaugggacggagggagu...............................  1  0  S02
...........................................................cuuacauuaugggacggagggagu...............................  1  0  S05
...........................................................cuuacauuaugggacggagggagu...............................  4  0  S04
............................................................uuacauuaugggacggaggga.................................  2  0  S02
............................................................uuacauuaugggacggaggga.................................  2  0  S04
............................................................uuacauuaugggacggaggga.................................  1  0  S05
............................................................uuacauuaugggacggagggagu...............................  1  0  S02
...............................................................cauuaugggacggagggagu...............................  1  0  S04
................................................................auuaugggacggagggaguac.............................  1  0  S01
................................................................auuaugggacggagggaguac.............................  2  0  S02
................................................................auuaugggacggagggaguac.............................  2  0  S04
................................................................auuaugggacggagggaguac.............................  2  0  S06
.................................................................uuaugggacggagggaguacu............................  1  0  S04
..................................................................uaugggacggagggaguacua...........................  1  0  S04
```

Provisional ID	:	ta_iwgsc_3b_v1_7382794_3001503
Score total	:	180.5
Score for star read(s)	:	3.9
Score for read counts	:	173
Score for mfe	:	2
Score for randfold	:	1.6
Score for cons. seed	:	
Total read count	:	351
Mature read count	:	351
Loop read count	:	2
Star read count	:	32

novel-m0343-5p

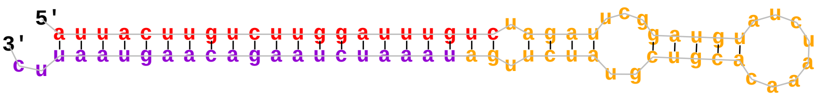

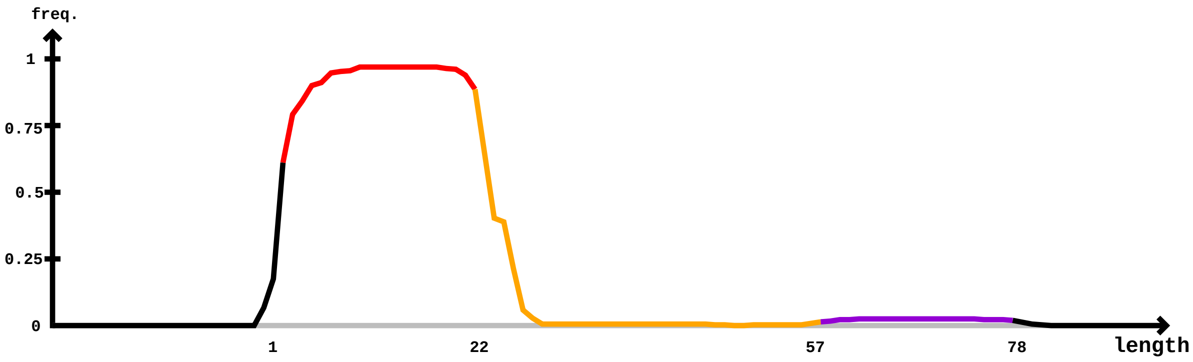

Mature **Star**

```
5'- acaccuaaaaaacaugucccaaauuacuugucuuggauuugucuagauucggauguaucuaaacacgucguaucuugauaaaucuaagacaaguaauucaggacagagagagua   -3'   obs
    acaccuaaaaaacaugucccaaauuacuugucuuggauuugucuagauucggauguaucuaaacacgucguaucuugauaaaucuaagacaaguaauucaggacagagagagua         exp
    ..............(((((..((((((((((((((((((((((.((((...(((((........)))))..)))).))))))))))))))))))))))..)))))..........   reads   mm   sample
    ....................aaauuacuugucuuggauuug.........................................................................   3   0   S05
    ....................aaauuacuugucuuggauuug.........................................................................   1   0   S06
    ....................aaauuacuugucuuggauuuguc.......................................................................   3   0   S05
    ....................aaauuacuugucuuggauuuguc.......................................................................   2   0   S04
    ....................aaauuacuugucuuggauuugucu......................................................................   5   0   S04
    ....................aaauuacuugucuuggauuugucu......................................................................   3   0   S02
    ....................aaauuacuugucuuggauuugucu......................................................................   4   0   S06
    ....................aaauuacuugucuuggauuugucu......................................................................   1   0   S05
    ....................aaauuacuugucuuggauuugucu......................................................................   1   0   S01
    ....................aaauuacuugucuuggauuugucu......................................................................   1   0   S03
    .....................aauuacuugucuuggauu...........................................................................   1   0   S01
    .....................aauuacuugucuuggauu...........................................................................   1   0   S02
    .....................aauuacuugucuuggauuu..........................................................................   1   0   S04
    .....................aauuacuugucuuggauuug.........................................................................   1   0   S04
    .....................aauuacuugucuuggauuug.........................................................................   1   0   S01
    .....................aauuacuugucuuggauuug.........................................................................   1   0   S06
    .....................aauuacuugucuuggauuugu........................................................................   1   0   S04
    .....................aauuacuugucuuggauuugu........................................................................   3   0   S01
    .....................aauuacuugucuuggauuugu........................................................................   2   0   S02
    .....................aauuacuugucuuggauuugu........................................................................   4   0   S03
    .....................aauuacuugucuuggauuuguc.......................................................................   4   0   S01
    .....................aauuacuugucuuggauuuguc.......................................................................   1   0   S02
    .....................aauuacuugucuuggauuugucu......................................................................   1   0   S06
    .....................aauuacuugucuuggauuugucu......................................................................   1   0   S03
    .....................aauuacuugucuuggauuugucu......................................................................   6   0   S04
    .....................aauuacuugucuuggauuugucu......................................................................   1   0   S01
    .....................aauuacuugucuuggauuugucu......................................................................   4   0   S05
    .....................aauuacuugucuuggauuugucua.....................................................................   1   0   S05
    .....................aauuacuugucuuggauuugucua.....................................................................   2   0   S03
    .....................aauuacuugucuuggauuugucua.....................................................................   1   0   S06
    .....................aauuacuugucuuggauuugucuag....................................................................   1   0   S04
    ......................auuacuugucuuggauuug.........................................................................   1   0   S03
    ......................auuacuugucuuggauuugu........................................................................   1   0   S05
```

```
                      Mature                                                  Star
acaccuaaaaaacaugucccaaauuacuugucuuggauuugucuagauucggauguaucuaaacacgucguaucuugauaaaucuaagacaaguaauucaggacagagagagua
......................auuacuugucuuggauuugu........................................................................    2    0    S02
......................auuacuugucuuggauuugu........................................................................    1    0    S04
......................auuacuugucuuggauuugu........................................................................    1    0    S03
......................auuacuugucuuggauuugu........................................................................    1    0    S01
......................auuacuugucuuggauuuguc.......................................................................    14   0    S05
......................auuacuugucuuggauuuguc.......................................................................    16   0    S01
......................auuacuugucuuggauuuguc.......................................................................    13   0    S04
......................auuacuugucuuggauuuguc.......................................................................    11   0    S03
......................auuacuugucuuggauuuguc.......................................................................    24   0    S02
......................auuacuugucuuggauuuguc.......................................................................    12   0    S06
......................auuacuugucuuggauuugucu......................................................................    2    0    S05
......................auuacuugucuuggauuugucu......................................................................    1    0    S03
......................auuacuugucuuggauuugucu......................................................................    3    0    S04
......................auuacuugucuuggauuugucu......................................................................    2    0    S02
......................auuacuugucuuggauuugucua.....................................................................    1    0    S02
......................auuacuugucuuggauuugucuag....................................................................    5    0    S01
......................auuacuugucuuggauuugucuag....................................................................    5    0    S03
......................auuacuugucuuggauuugucuag....................................................................    2    0    S02
......................auuacuugucuuggauuugucuag....................................................................    15   0    S04
......................auuacuugucuuggauuugucuag....................................................................    13   0    S05
......................auuacuugucuuggauuugucuag....................................................................    6    0    S06
......................auuacuugucuuggauuugucuaga...................................................................    2    0    S05
......................auuacuugucuuggauuugucuaga...................................................................    1    0    S01
......................auuacuugucuuggauuugucuaga...................................................................    2    0    S06
.......................uuacuugucuuggauuugu........................................................................    1    0    S01
.......................uuacuugucuuggauuugu........................................................................    1    0    S05
.......................uuacuugucuuggauuuguc.......................................................................    3    0    S04
.......................uuacuugucuuggauuuguc.......................................................................    1    0    S06
.......................uuacuugucuuggauuuguc.......................................................................    1    0    S05
.......................uuacuugucuuggauuugucu......................................................................    8    0    S01
.......................uuacuugucuuggauuugucu......................................................................    6    0    S05
.......................uuacuugucuuggauuugucu......................................................................    3    0    S03
.......................uuacuugucuuggauuugucu......................................................................    5    0    S06
.......................uuacuugucuuggauuugucu......................................................................    2    0    S02
.......................uuacuugucuuggauuugucu......................................................................    5    0    S04
.......................uuacuugucuuggauuugucuag....................................................................    2    0    S05
.......................uuacuugucuuggauuugucuag....................................................................    1    0    S02
.......................uuacuugucuuggauuugucuag....................................................................    5    0    S06
.......................uuacuugucuuggauuugucuaga...................................................................    4    0    S06
.......................uuacuugucuuggauuugucuaga...................................................................    5    0    S05
.......................uuacuugucuuggauuugucuaga...................................................................    8    0    S04
.......................uuacuugucuuggauuugucuaga...................................................................    2    0    S02
.......................uuacuugucuuUgauuugucuaga...................................................................    1    1    S03
.......................uuacuugucuuggauuugucuaga...................................................................    1    0    S01
........................uacuugucuuggauuugu........................................................................    1    0    S03
........................uacuugucuuggauuuguc.......................................................................    1    0    S05
........................uacuugucuuggauuuguc.......................................................................    1    0    S04
........................uacuugucuuggauuugucu......................................................................    1    0    S02
........................uacuugucuuggauuugucuag....................................................................    1    0    S02
........................uacuugucuuggauuugucuag....................................................................    2    0    S05
........................uacuugucuuggauuugucuag....................................................................    1    0    S03
........................uacuugucuuggauuugucuag....................................................................    1    0    S06
........................uacuugucuuggauuugucuaga...................................................................    1    0    S05
........................uacuugucuuggauuugucuaga...................................................................    1    0    S03
........................uacuugucuuggauuugucuaga...................................................................    2    0    S02
........................uacuugucuuggauuugucuaga...................................................................    1    0    S06
........................uacuugucuuggauuugucuagau..................................................................    3    0    S05
........................uacuugucuuggauuugucuagau..................................................................    1    0    S01
.........................acuugucuuggauuuguc.......................................................................    1    0    S06
.........................acuugucuuggauuugucu......................................................................    1    0    S05
.........................acuugucuuggauuugucuag....................................................................    1    0    S02
.........................acuugucuuggauuugucuaga...................................................................    2    0    S01
.........................acuugucuuggauuugucuaga...................................................................    3    0    S05
.........................acuugucuuggauuugucuaga...................................................................    2    0    S04
.........................acuugucuuggauuugucuaga...................................................................    1    0    S03
.........................acuugucuuggauuugucuaga...................................................................    4    0    S06
.........................acuugucuuggauuugucuagau..................................................................    2    0    S04
.........................acuugucuuggauuugucuagau..................................................................    2    0    S06
.........................acuugucuuggauuugucuagau..................................................................    1    0    S02
.........................acuugucuuggauuugucuagau..................................................................    1    0    S05
```

```
                      Mature                                        Star
acaccuaaaaaacaugucccaaauuacuugucuuggauuugucuagauucggauguaucuaaacacgucguaucuugauaaaucuaagacaaguaauucaggacagagagagua
..........................cuugucuuggauuugucuag....................................................................   1   0   S03
..........................cuugucuuggauuugucuag....................................................................   1   0   S06
..........................cuugucuuggauuugucuaga...................................................................   1   0   S02
..........................cuugucuuggauuugucuaga...................................................................   1   0   S06
...........................uugucuuggauuugucua.....................................................................   1   0   S02
...........................uugucuuggauuugucuaga...................................................................   1   0   S04
...........................uugucuuggauuugucuaga...................................................................   3   0   S01
...........................uugucuuggauuugucuaga...................................................................   4   0   S05
...........................uugucuuggauuugucuagauu.................................................................   3   0   S05
...........................uugucuuggauuugucuagauu.................................................................   1   0   S01
............................ugucuuggauuugucuaga...................................................................   1   0   S04
............................ugucuuggauuugucuaga...................................................................   1   0   S05
.............................gucuuggauuugucuaga...................................................................   1   0   S04
..............................ucuuggauuugucuagau..................................................................   1   0   S04
..............................ucuuggauuugucuagauu.................................................................   1   0   S03
..............................ucuuggauuugucuagauu.................................................................   2   0   S04
..............................ucuuggauuugucuagauu.................................................................   1   0   S01
............................................agauucggauguaucuaaacacg...............................................   1   0   S05
.............................................gauucggauguaucuaaacacguc.............................................   1   0   S05
.......................................................................aucuugaCaaaucuaagacaagua...................   1   1   S03
.............................................................................auaaaucuaagacaaguaauu................   1   0   S02
.............................................................................auaaaucuaagacaaguaauuc...............   1   0   S04
..............................................................................uaaaucuaagacaaguaauuc...............   1   0   S02
..............................................................................uaaaucuaagacaaguaauuc...............   1   0   S01
...............................................................................aaaucCaagacaaguaauucagg............   1   1   S06
................................................................................aaucuaagacaaguaauuca..............   1   0   S05
................................................................................aaucuaagacaaguaauucag.............   1   0   S01
..................................................................................ucuaagacaaguaauuca..............   1   0   S04
```
