# Supplementary figures and images for: Identification and Comparative Analysis of microRNA in Wheat (Triticum aestivum L.) Callus Derived from Mature and Immature Embryos during In vitro Culture
Source: Front Plant Sci. 2016 Aug 30;7:1302. doi: 10.3389/fpls.2016.01302 (PMC5003897; doi:10.3389/fpls.2016.01302)

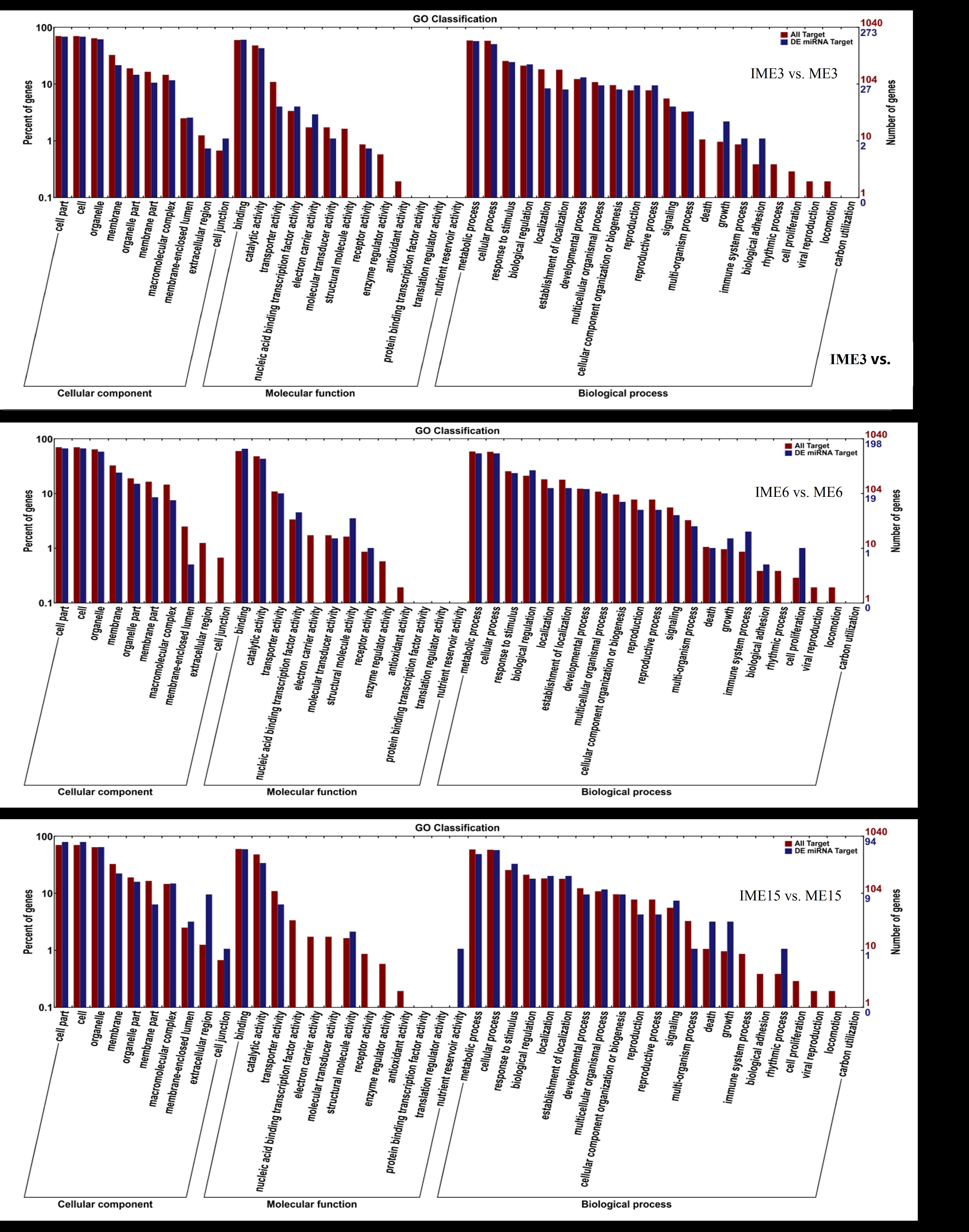

Supplement: Image S3 — Gene ontology of predicted targets for all differentially expressed miRNAs. Categorization of miRNA-target genes was performed according to cellular components, molecular functions, and biological processes. [file Image3.jpeg]
